# Supplementary material for: Real-time in situ magnetization reprogramming for soft robotics
Source: Nature. 2025 Aug 4;645(8080):375–84. doi: 10.1038/s41586-025-09459-0 (PMC12422961; doi:10.1038/s41586-025-09459-0)
Supplement: Supplementary file 1 — This file contains Supplementary Notes 1–11, Supplementary Figs. 1–96 and legends for the Supplementary Videos. [file 41586_2025_9459_MOESM1_ESM.pdf]

nature portfolio

https://doi.org/10.1038/s41586-025-09459-0

Supplementary information

Real-time in situ magnetization reprogramming for soft robotics

In the format provided by the
authors and unedited

Supplementary Information for

Real-time in-situ magnetization reprogramming for soft robotics

Xianqiang Bao *et al.*
Corresponding author: Metin Sitti, msitti@ku.edu.tr

The PDF file includes:

Supplementary Notes

Supplementary Note 1- Background on Magnetization Reprogramming and Its Application **2**

Supplementary Note 2- Mechanical Analysis of Soft Tube Deformation **8**

Supplementary Note 3- Experimental Analysis of Soft Tube Deformation **14**

Supplementary Note 4- Error Analysis of Magnetic Unit Position Control **18**

Supplementary Note 5- Catheter-Blood Vessel Contact Analysis **22**

Supplementary Note 6- Experimental Analysis of Tube Manipulation **26**

Supplementary Note 7- Feasibility of 3D Manipulations without Altering External Magnetic Fields **32**

Supplementary Note 8- Force-Torque Space Analysis **41**

Supplementary Note 9- Design and Analysis of Tube Manipulation Schemes **52**

Supplementary Note 10- Design of Magnetization Profile **102**

Supplementary Note 11- Analysis of Multi-Magnetic Field Interference **117**

Supplementary Note 12- Detailed Procedures and Discussion for Coordinated Multi-Instrument Operation Experiments **123**

Supplementary Figs. 1 to 96 .... **126**

Legends for Supplementary Videos 1 to 21 .... **222**

Supplementary references **229**

Other Supplementary Information for this manuscript includes the following:

Supplementary Videos 1 to 21

Supplementary Note 1:

Background on Magnetization Reprogramming and Its Application

To conserve space in the main text, we outline the relevant research background in this supplementary note. We begin with an overview of the current state of magnetization reprogramming, and then provide a detailed discussion of the background for each of the four applications introduced in this study: contact-free object navigation, reprogrammable cilia arrays, coordinated multi-instrument operation, and shape-adaptable soft grippers.

1. Magnetization reprogramming

In magnetic soft robots, morphing typically involves altering an external magnetic field. Nevertheless, the morphing range attainable with this method can be limited. Generally, a specific magnetization profile can generate only a single or a specific range of morphing modes, while diverse tasks and multiple functions can demand more drastically different morphing sequences. Even for some morphing modes that can be generated by certain fixed magnetization profiles, relying entirely on external magnetic fields would lead to complex control schemes, bulky and intricate magnetic field generation systems, and challenges in actuating specific regions of soft robots (*1*, *2*). Therefore, it is essential to be able to reprogram the magnetization profile of soft robots during their applications requiring diverse tasks or multiple functions (*3*, *4*). Additionally, many operational tasks demand a high degree of continuity, which places increased demand on reprogramming speed and in-situ operation. The inability to conduct real-time, in-situ magnetization reprogramming adversely impacts the operational continuity of magnetically controlled robots and imposes significant constraints on their applications.

Existing studies have explored reprogramming techniques, such as phase change of the base materials (*5–8*) and Curie temperature-based reorientation of magnetic domains (*3*). Although these approaches facilitate reprogramming, they limit real-time operations, as methods like laser heating or oven heating require sufficient time for

both heating and cooling. Besides, the depth of heating and selection of heating points impose specific requirements on the wall thickness and shape of the soft robot; for example, laser heating is only suitable for two-dimensional structures and thin-walled designs (*3*). Furthermore, the deployment of heating technology is constrained by the operating workspace, consequently limiting in-situ operability to some extent. Another approach uses a viscous liquid to localize magnetic nanoparticles, avoiding heating-cooling cycles but offering only two magnetization profiles and fixed magnetic field orientations (*9*). Since this approach requires additional equipment to drive the slow motion of nanoparticles in a highly viscous medium, it is incapable of achieving real-time, in-situ magnetization reprogramming. There are also template-based reprogramming methods (*10*, *11*), which require templates for positioning, still limiting both the real-time capability and in-situ operability of reprogramming.

2. Four different application scenarios

2.1. Contact-free object navigation

Minimally invasive surgery (MIS) is now widely utilized due to its benefits in reducing tissue trauma, decreasing postoperative recovery time, and lowering the risk of complications (*12–14*). Common forms of MIS include vascular interventional surgery (*15–20*), laparoscopic surgery (*21–23*), endoscopic surgery (*24*, *25*), and ophthalmic procedures (*26–28*). In MIS, minimally invasive instruments are externally manipulated through incisions or natural orifices to reach the target site for diagnosis or treatment (*14*). One prevalent operating technique, as depicted in Fig. 2a(i), involves navigating these instruments around obstacles, demonstrating the flexibility of MIS. To facilitate this, control over the instrument’s form is essential, commonly achieved through pneumatic systems (*29*), hydraulic systems (*30*), magnetic systems(*31*, *32*), and tendon/cable mechanisms (*21*, *33*). However, during the movement of common surgical instruments, unavoidable contact with avoidance objects may occur, leading to varying degrees of tissue damage. This is detrimental to patient recovery and may even cause complications, particularly when the avoidance tissue is already damaged (*14*, *19*, *34*) (Fig. 2a(i)). Specifically, for vascular

intervention surgeries (Fig. 2a(ii)), the seriousness of vascular complications makes such contact particularly noteworthy. Even in successful surgeries, contact between instruments and blood vessels is inevitable. Such damage is particularly concerning for older patients whose vascular physical properties are compromised (e.g., reduced elasticity of vascular walls), not only increasing their surgical risks but also limiting their options for diagnosis or treatment through vascular intervention. In vascular interventional surgeries, the parts of the catheter that collide or contact with the vascular wall are divided into the head and body. Damage to the vascular wall by the catheter head primarily results from direct impact. The primary source of damage to the vascular wall from the catheter body is friction. This friction is less impactful in blood vessels with smaller curvatures but cannot be ignored in blood vessels with larger curvatures or at bifurcations. We conducted a detailed analysis and discussion on this issue (Supplementary Note 5, Supplementary Fig. 30). Even by the most conservative estimates, the friction caused by this contact is 62.5 times greater than that caused by the weight of the catheter. Efforts must be made to eliminate this friction and ensure the catheter is in an optimal contact condition (Supplementary Fig. 30C).

Regarding the contact of the catheter head with the vascular wall, numerous studies have been conducted, mainly focusing on measuring the operating force at the proximal or distal end of the catheter and combining it with safe operation mechanisms and algorithms (*19*, *35*) to reduce vessel damage. The most common approach to mitigate the catheter body's contact with the vessel involves surface treatments, such as hydrogel skin (*32*) or commercial resin coatings (*36*), to reduce friction between the catheter and the vessel. However, these methods do not fundamentally eliminate such contacts. The study in literature (*37*) introduces an advanced method of manipulating magnetization combinations to alter its shape near obstacles, enabling the catheter to traverse them, a significant improvement over previous research. However, due to the limitation that its magnetization profile cannot freely adjust to positional changes, the bent part of the catheter is influenced by its forward or backward movements, and thus lacks independent motion. As depicted in

Fig. 2b(i), the bent section moves along with the overall motion, which still fails to address the issue fundamentally. In addition, a technique that combines magnets with low-melting-point alloys has been developed (*38*, *39*). This approach uses magnetic forces to deform the tube and leverages the alloy's solidification and melting processes to maintain or release the tube's shape. The technique shows the potential for reducing vascular damage. However, heating and cooling within a sealed chamber is time-consuming, which precludes real-time operation and raises concerns about excessive heat accumulation.

2.2. Reprogrammable cilia array

The ubiquitous cilia in nature play a paramount role in various creatures, including human beings, due to their essential functionalities such as cerebrospinal fluid transportation (*40*), mucus clearance (*41*), egg cell delivery (*42*), self-propulsion (*43*), food capture (*44*), as well as self-cleaning (*45*). Inspirations were leveraged to create artificial cilia for versatile on-chip integrated microfluidic pumps and mixers, controlled particle and droplet manipulators, self-cleaning and antifouling surfaces, environmental information sensors, and soft robots(*46–48*). Although dramatic progress has been made in the field of artificial cilia (*49–53*), there is still a long way to go for the real application of artificial cilia in areas including microfluidics, chemical engineering, biological engineering, biomedical science, and robotics. The primary reason is the lack of in-situ reprogrammability of existing artificial cilia targeting desired reconfigurable fluid and particle/cell manipulation capabilities as well as reprogrammable locomotion capacities that are tunable on the fly using a simple and efficient strategy. For example, programmable magnetic artificial cilia could only generate specific flow patterns using predefined cilia coordination or phase shift (*53–55*). Moreover, electrically actuated artificial cilia are either cost-intensive or require a cumbersome sophisticated actuation platform inhibiting their actual application despite their ability to induce reconfigurable flow patterns (*49*, *50*).

2.3. Coordinated multi-instrument operation

In medical diagnosis and treatment, multiple medical instruments are commonly needed to work together to achieve the desired outcome. For example, catheters and guidewires are used together in vascular interventional surgeries (*17*, *20*), and multiple robotic arms or grippers are employed in laparoscopic procedures (*56*). Currently, various actuation mechanisms have been investigated and implemented in medical procedures (*21*, *29*, *30*, *32*, *57*). In magnetic actuation, the locomotion or shape control of instruments is achieved through the gradient force or torque exerted by an external magnetic field. Nevertheless, due to the pervasive nature of magnetic fields, which affect magnetic objects within their range non-selectively, independently controlling multiple targets using the same magnetic field poses a considerable challenge (*58*). A typical minimally invasive surgery scenario is illustrated in Fig. 4a, where the diagnostic or treatment target is obscured by a tissue. This necessitates the use of instrument A to appropriately reposition the obstructive tissue, ensuring sufficient workspace for instrument B to either diagnose or treat the target tissue. Additionally, instrument C is often required to enhance visibility (by obtaining operational images) within the body or to assist during the diagnostic and treatment processes. The conventional method of single magnetic field control is inadequate for such operations; the use of multiple magnetic fields, though posing significant challenges, also raises questions about the feasibility of completing such tasks. Therefore, magnetic actuation methods are often limited to manipulating a single instrument, restricting their use in scenarios requiring multiple instruments.

2.4. Shape adaptable soft grippers

Compared to traditional grippers, soft grippers exhibit unique advantages in interacting with objects due to their excellent compliance and adaptability. Their common actuation mechanisms include cable-driven actuation (*59–61*), pneumatic-driven actuation (*62–64*), dielectric elastomer actuators (*65–67*), and shape memory alloys (*68–70*). In contrast to these actuation methods, magnetically-driven actuation offers notable advantages in reducing size and simplifying components. The manipulation can be easily accomplished when utilizing magnetic fields to control

soft grippers; however, it is generally limited to basic gripping actions. The actuation of soft grippers depends on their magnetization profiles and external magnetic fields, posing challenges for scenarios requiring adaptive gripping conforming to the shape of the object. Achieving specific shapes in soft grippers through external magnetic fields results in a limited number of shapes, satisfying only highly specialized needs. The optimal approach involves modifying the magnetization profile of the soft gripper itself to achieve the necessary deformations for effective gripping—this method is termed "real-time in-situ magnetization reprogramming," as proposed in this study.

Supplementary Note 2:

Mechanical Analysis of Soft Tube Deformation

To analyze the deformation of soft tubes in magnetic fields, this study employs the pseudo-rigid body method. In this method, the soft tube is modeled as a series of rigid rods connected by hinges. These segments remain undeformed under external forces, while the hinges undergo rotational motion. The elastic potential energy resulting from the deformation of the soft tube is assumed to be stored in these deforming hinges. As the soft tube deforms, the angles between the rigid rods change. By subdividing these segments, a curve composed of segments at varying angles can be constructed, thereby approximating the deformation of the soft tube. This study applies the energy method, specifically the principle of minimum potential energy, to approximate the deformation of the soft tube.

To simplify and accurately analyze the deformation of soft tubes, this study opts for suspending the tubes. It's noteworthy that similar force analysis processes apply to other placement methods, requiring only adjustments in the direction of the applied forces or the addition of corresponding forces. When a magnetic field is applied to the soft tube, it is subjected to both gravity and the magnetic field. The total potential energy of the system is composed of magnetic potential energy, elastic potential energy, and gravitational potential energy. The total potential energy is expressed as:

$$V = U_m + U_g + U_e \tag{S2-1}$$

where U_m represents the magnetic potential energy of the system;

U_g means the gravitational potential energy of the system;

U_e represents the elastic potential energy of the system.

1. Magnetic potential energy

In this study, the soft tube is modeled as a structure comprised of n rigid rods connected by $n-1$ hinges (as shown in Supplementary Fig. 57), with m of these rods possessing magnetic properties. Within a magnetic field, these magnetized rigid rods

experience magnetic forces, and the total magnetic potential energy of these *m* magnetic rods can be represented as:

$$U_m = -\sum_{j=1}^{m} V_{mj}$$

(S2-2)

Here, V_{mj} represents the magnetic potential energy of a single rod, which can be calculated using the following formula:

$$V_{mj} = \mathbf{m_j} \cdot \mathbf{B_j} \tag{S2-3}$$

In this context, $\mathbf{m_j}$ denotes the magnetic moment of the jth rod; $\mathbf{B_j}$ represents the magnetic field strength experienced by the jth rod. The magnetic moment of the jth rod can be obtained by

$$\mathbf{m_j} = m_j \mathbf{r_j} \tag{S2-4}$$

where m_j represents the magnitude of the magnetic moment of the jth rod and $\mathbf{r_j}$ is the unit direction vector of the jth rod.

As shown in Supplementary Fig. 57, this study defines the horizontal rightward direction as the positive *x*-axis and the vertical downward direction as the positive *y*-axis. Assume that the angle between the ith rod and the positive direction of the *x*-axis is α_i, and the angle between the $i+1^{th}$ rod and the ith rod is θ_i. Since the deformation of the soft tube is considered to occur within the same plane, it follows that:

$$\theta_i = \alpha_{i+1} - \alpha_i \tag{S2-5}$$

In the computation process, the angle α_1 of the first rod with the *x*-axis can be set to an initial value (for instance, 90 degrees when the soft tube is vertically suspended), then:

$$\alpha_i = \alpha_1 + \sum_{n=1}^{i-1} \theta_n$$

(S2-6)

The unit direction vector of each rod can be represented as:

$$\mathbf{r}_{\boldsymbol{i}} = (\cos\alpha_i\,, \sin\alpha_i) \tag{S2-7}$$

Therefore, the total magnetic potential energy of the system can be expressed as:

$$U_m = -\sum_{j=1}^{m} m_j \mathbf{r}_{\boldsymbol{j}} \cdot \mathbf{B}_{\boldsymbol{j}}$$

(S2-8)

2. Elastic potential energy

Since the soft tube is modeled as a combination of rigid rods and hinges, the elastic potential energy of the system is stored only in the hinges. The elastic potential energy in a hinge depends on the angle between adjacent rods, and the energy of a single hinge can be expressed as:

$$V_e = \frac{1}{2} K\theta_i^2 \tag{S2-9}$$

where *K* represents the equivalent rotational stiffness of the hinge and its magnitude is given by:

$$K = \frac{E_i I_i}{L_R}$$

(S2-10)

Here, E_i represents the modulus of elasticity of the ith rod;
I_i is the moment of inertia of the cross-section of the ith rod;
L_R is the length of the ith rod.

Since the soft tube is evenly divided into *n* rigid rods, L_R can be obtained by:

$$L_R = \frac{L}{n}$$

(S2-11)

Thus, the total elastic potential energy of the system can be calculated by:

$$U_e = \frac{2n}{L}\sum_{i=1}^{n} E_i I_i \theta_i^2$$

(S2-12)

3. Gravitational potential energy

To facilitate calculations, this study assigns corresponding vectors to each rod. The initial vector of the ith rod is denoted as $\mathbf{p}_i^i$, and upon the deformation of the soft tube under the influence of external forces, the final vector of the rod becomes $\mathbf{p}_i^f$. Assuming that the rigid rods in the pseudo-rigid body model have a uniformly distributed mass, the center of gravity of each rod coincides with its geometric center. Therefore, the vector representing the change in the center of gravity position of the ith rod is expressed as:

$$\mathbf{\Delta p}_i = \frac{1}{2}(\mathbf{p}_i^f - \mathbf{p}_i^i) \quad \text{(S2-13)}$$

Since the initial position of the soft tube is vertically suspended, the initial position vectors of all the rods are:

$$\mathbf{p}_i^i = (0, L_R) \quad \text{(S2-14)}$$

The final vector of all rods are

$$\mathbf{p}_i^f = L_R \mathbf{r}_i \quad \text{(S2-15)}$$

Assuming the mass of the ith rod is m_i^m, the gravitational potential energy of the system can be represented as:

$$U_g = -\frac{1}{2}\sum_{i=1}^{n} m_i^m (\mathbf{p}_i^f - \mathbf{p}_i^i)\cdot \mathbf{g}$$

(S2-16)

where **g** is the acceleration due to gravity.

Through the aforementioned analysis, we have derived the magnetic potential energy, elastic potential energy, and gravitational potential energy. According to Equation (1) and utilizing the principle of minimum potential energy, we can determine the static equilibrium configuration by finding the local minima of the potential energy. This will allow us to obtain the angles between the rods, denoted as θ_i. The potential energy equation can be solved using the fmincon function in MATLAB (R2022a, The MathWorks, Inc., USA), which is a solver for finding the minimum of a constrained nonlinear multivariable function.

4. Parameter calculation

To measure the deformation of the soft tube, we select two parameters: the terminal bending angle and the minimum radius of curvature in the bending area (Supplementary Fig. 29). If there are multiple bending areas, then there will be multiple minimum radii of curvature.

Based on the solved data θ_i, the terminal bending angle of the soft tube (i.e., the nth rod) can be obtained through the following calculation:

$$\alpha_n = \alpha_1 + \sum_{i=1}^{n} \theta_i$$

(S2-17)

To obtain the minimum radius of curvature, first, using the solved data θ_i, the initial angle α_1 of the first rod, and the length of the rod L_R, calculate the coordinates of both ends of the rod as well as the midpoint of the rod. Let the coordinates of the end of the ith rod be $(x_i,\ y_i)$, which can be obtained through the following calculation:

$$\begin{cases} x_i = x_{i-1} + L_R \cos \alpha_i \\ y_i = y_{i-1} + L_R \sin \alpha_i \\ \alpha_i = \alpha_1 + \sum_{n=1}^{i-1} \theta_n \end{cases}$$

(S2-18)

Let the geometric midpoint coordinates of the ith rod be ($x_{i,mid}$, $y_{i,mid}$), which can be obtained through the following calculation:

$$\begin{cases} x_{i,mid} = x_{i-1} + \frac{1}{2} L_R \cos \alpha_i \\ y_{i,mid} = y_{i-1} + \frac{1}{2} L_R \sin \alpha_i \end{cases}$$

(S2-19)

Using the method of least squares, these discrete coordinates are fitted into a curve. By dividing the fitted curve into segments and fitting these segments into circles, the radius R of the circles can be obtained. The radius of these circles represents the radius of curvature (the same method used to calculate experimental data in Supplementary Fig. 29). By finding the minimum value within a bending area, the minimum radius of curvature in that area is obtained:

$$R_{min} = \min (R) \qquad \text{(S2-20)}$$

Supplementary Note 3:

Experimental Analysis of Soft Tube Deformation

The deformation of the soft tubes is influenced by various factors, including the magnetic moments of the magnetic units, the number of magnetic units, the magnetic field, the elastic modulus, and the moment of inertia. The theoretical calculations for soft tube deformation are detailed in Supplementary Note 2, where theoretical values are compared with actual experimental data (Supplementary Fig. 27). To more vividly illustrate the influence of various parameters on soft tube deformation, we conducted experiments and presented the effects from an experimental perspective. This section provides more intuitive references and specific design data for soft tube design.

The forces or torques acting on a soft tube in a magnetic field are determined by the magnetic moment and the magnetic field. For instance, the torque on the tube can be calculated as follows:

$$\boldsymbol{\tau} = \mathbf{m} \times \mathbf{B} \tag{S3-1}$$

where $\mathbf{m}$ represents the magnetic moment, and $\mathbf{B}$ denotes the magnetic field. The specific magnitude of the torque

$$|\boldsymbol{\tau}| = |\mathbf{m}||\mathbf{B}|\sin\theta \tag{S3-2}$$

where θ is the angle between the magnetic moment vector and the magnetic field vector. We can understand that when the directions of the magnetic moment and the magnetic field remain constant, the magnitudes of the magnetic moment and the magnetic field strength have equivalent effects on the torque produced; they influence the magnitude of the magnetic moment in the same way. Similarly, the forces experienced by a soft tube in a gradient magnetic field follow a comparable trend. Therefore, when considering the effects of the magnetic moment and magnetic field strength on tube deformation, since both contribute to the generation of force or torque and exhibit similar trends in their effects, it is sufficient to consider the impact of either the magnetic moment or the magnetic field strength alone. Considering the

advantages of creating and adjusting magnetic fields with precision, we use the same magnetic units in the manufacture of soft tubes, controlling variables by adjusting the magnitude of the magnetic field.

Based on the analysis of the effects of magnetic moment and magnetic field, we set five different magnetic field strengths at 30 mT, 60 mT, 90 mT, 120 mT, and 150 mT. For the distribution of magnetic units, we used the most common configuration of magnetic units discussed in this paper (two magnetic units), setting the distance between magnetic units at 6mm, 12mm, 18mm, and 24mm. To assess the impact of the elastic modulus, different materials were used to fabricate the soft tubes. We utilized Polydimethylsiloxane (PDMS) (Sylgard 184, Dow Corning, US) as the manufacturing material and varied the base-to-curing agent mass ratio at 3:1, 5:1, 10:1, and 15:1 to achieve tubes with different elastic moduli. For the influence of the moment of inertia, as the tubes involved in this study possess similar cross-sectional structures and the parameters do not vary significantly, the impact on deformation is relatively minor. Therefore, we chose not to conduct experiments on the effect of the moment of inertia on deformation for the time being. Following the analyses above, we fabricated different soft tubes using Configuration C from 1D magnetization reprogramming, as outlined in Supplementary Figs. 2 and 9; specific parameters can be found in Supplementary Fig. 58. In the experiments, the soft tubes were placed flat on a plate, and a magnetic field was applied in a direction parallel to the plate and perpendicular to the tube's axis, as illustrated in Supplementary Fig. 58C.

In experiments on magnetic field effects, we used tubes made from PDMS with a base-to-curing agent mass ratio of 10:1 and selected magnetic unit distances of 12mm, 18mm, and 24mm. We applied uniform magnetic fields of five different strengths: 30 mT, 60 mT, 90 mT, 120 mT, and 150 mT. For each parameter configuration, the experiment was repeated three times, and the deformation of the soft tube was recorded for each trial. We also used the radius of curvature and terminal bending angle (definitions provided in Supplementary Fig. 29) to describe the deformation of the soft tube, with specific data shown in Supplementary Fig. 59. We observed that as the magnetic field strength increases, the radius of curvature of the soft tube with the

same magnetic unit distance gradually decreases, while the terminal bending angle progressively increases. This indicates that the deformation of the soft tube increases with the intensity of the magnetic field. Soft tubes with different magnetic unit distances also exhibit the same trend.

In experiments on magnetic unit distance effects, we also employed soft tubes made from PDMS with a base-to-curing agent mass ratio of 10:1, featuring magnetic unit distances of 6mm, 12mm, 18mm, and 24mm. We observed the deformation of these soft tubes under magnetic field strengths of 60 mT, 90 mT, and 120 mT, with each parameter configuration being tested three times. The radius of curvature and terminal bending angle of the soft tube are documented in Supplementary Fig. 60. Our findings indicate that under a consistent magnetic field, the radius of curvature of the soft tube increases with an increase in magnetic unit distance, as does the terminal bending angle. This suggests that soft tubes with larger magnetic unit distances are more likely to exhibit larger arc-shaped bends. A similar trend is observed across different magnetic field strengths.

In experiments on elastic modulus effects, we set the magnetic unit distance at 12mm and employed soft tubes fabricated from PDMS with varying base-to-curing agent mass ratios (3:1, 5:1, 10:1, 15:1). The deformation of these soft tubes was observed under magnetic fields of 60 mT, 90 mT, and 120 mT, with each experimental setup repeated three times. Supplementary Fig. 61 shows the radius of curvature and terminal bending angle of the soft tubes. The results indicate that under the same magnetic field strength, soft tubes with a higher base-to-curing agent mass ratio exhibit a smaller radius of curvature and a larger terminal bending angle. This trend is consistently observed across different magnetic field strengths. Additionally, to determine the elastic modulus of soft tubes made from PDMS with different base-to-curing agent mass ratios, we conducted tensile tests using a Universal Testing System (Model 5942, INSTRON, USA), as shown in Supplementary Fig. 70B. The corresponding values for the elastic modulus were obtained and are presented in Supplementary Fig. 62. The measured data indicate that higher base-to-curing agent mass ratios in PDMS result in a lower elastic modulus. Consequently, in the

experiments on elastic modulus effects, the degree of soft tube bending increases as the elastic modulus of the manufacturing material increases.

We employed experimental methods to investigate the factors influencing the deformation of soft tubes, analyzing how various factors affect the radius of curvature and terminal bending angle. This analysis provides insights for the design of soft tubes. Additionally, the specific experimental data obtained can be directly used as parameters in the design process.

Supplementary Note 4:

Error Analysis of Magnetic Unit Position Control

In this study, we control the position of magnetic units through the relative movement of multiple tubes. Given the flexibility of the tubes, the magnetic units are subjected to magnetic forces or torques when exposed to an external magnetic field. The tubes connected to the magnetic units undergo deformation under the influence of these forces/torques, leading to a deviation of the magnetic units from their predetermined positions. Axially, the position of the magnetic units is controlled by the inner tubes; therefore, axial extension or compression of the inner tubes affects the axial positioning of the magnetic units, which constitutes the source of axial control error. The outer tube forms the main structure of the soft robot, with the magnetic unit being one of its integral parts, capable of driving deformations in the soft robot, resulting in its extension and compression. These deformations are inherent working characteristics of the soft robot and are not considered as errors in the position control of the magnetic unit. Therefore, radially, the elastic deformation of the outer tube does not affect the positional accuracy of the magnetic unit. On the other hand, in this study, due to the gap between the inner and outer tubes, the magnetic unit cannot seamlessly fit into the outer tube, causing a certain degree of rotation. Thus, in this research, the positional control error of the magnetic unit is characterized by axial position error and angular error.

When calculating the axial position error, the magnetic unit may be subjected to the effects of a gradient magnetic field, a uniform magnetic field, or a combination of both. When a gradient magnetic field is applied, the force exerted on the magnetic unit by the gradient magnetic field can be expressed as

$$\mathbf{F}_{i}^{G} = \nabla(\mathbf{m}_{i} \cdot \mathbf{B}_{i}) \tag{S4-1}$$

where $\mathbf{m}_i$ represents the magnetic moment of magnetic unit *i*; $\mathbf{B}_i$ denotes the magnetic field experienced by magnetic unit *i*.

When a uniform magnetic field is applied, the torque exerted on the magnetic unit by the uniform magnetic field is

$$\mathbf{T}_i^U = \mathbf{m}_i \times \mathbf{B}_i \tag{S4-2}$$

Assuming a uniform distribution of magnetization in the magnetic unit, the force exerted on the tube connected to it can be derived as follows:

$$F_i^U = \frac{T_i^U}{L_m/2} \tag{S4-3}$$

Here, L_m denotes the axial length of the magnetic unit.

When the magnetic unit is subjected to the force $\mathbf{F}_i^G$ from a gradient magnetic field, it induces complex deformations in the inner tube, including bending, stretching, and even twisting. To obtain the maximum axial position error and simplify the calculation, we approximate the complex deformations caused by $\mathbf{F}_i^G$ on the inner tube as either stretching or compressing under axial force. Considering $\mathbf{F}_i^G$ to act solely in stretching or compressing is an extreme case possible in practical situations, where the amount of stretch or compression will be maximal. It will be feasible to calculate the maximum axial position error using this extreme case scenario.

Similarly, since the outer tube surrounding the magnetic unit exerts a reactive force on the magnetic unit, a portion of the force F_i^U derived from the torque experienced by the magnetic unit will be counteracted by this reactive force. In other words, the force acting on the inner tube will be less than F_i^U. However, for the sake of simplifying calculations and computing the maximum error, we neglect the external reactive force of the outer tube and apply the entire force F_i^U on the inner tube. This simplification is also viable for calculating the maximum axial position error.

In various applications, each inner tube may have multiple magnetic units attached, with the possibility of differing magnetization directions, leading to scenarios where the forces exerted on the inner tube may counteract each other. To simplify the calculation process and determine the maximum error, we assume that

the forces from multiple magnetic units are concentrated on a single magnetic unit for calculation purposes. Therefore, the maximum axial force can be calculated as follows:

$$F_{max} = \sum_{i=1}^{n} (F_i^G + F_i^U)$$

(S4-4)

where n represents the number of magnetic units.

Under axial force, whether stretching or compressing, the maximum deformation of the inner tube (i.e., the maximum axial position error of the magnetic unit) can be expressed as:

$$\varepsilon_a = \frac{F_{max} L_{in}}{E_{in} A_{in}}$$

(S4-5)

where L_{in} represents the length of the inner tube; E_{in} denotes the elastic modulus of the inner tube; A_{in} refers to the cross-sectional area of the inner tube.

In the calculation of angle errors controlled by magnetic units, due to the existence of gaps, the magnetic units do not maintain perfect axial alignment with the outer tube. To calculate the maximum angle error, as shown in Supplementary Fig. 63, we assume the magnetic units are placed in extreme positions. Similar to the analysis for calculating axial position errors, due to the elasticity of the outer tube, the magnetic units compress the outer tube, causing a bulge at the contact point. However, this phenomenon is a unique working characteristic of soft robots, and not a result of control errors of the magnetic units. Therefore, this scenario is not considered an angle error of magnetic unit control. Based on the geometric relationships depicted in the figure, the following formula can be derived:

$$L_m \sin \varphi_e + D_m \cos \varphi_e = D_{out} \quad \text{(S4-6)}$$

where L_m represents the axial length of the magnetic unit; φ_e is the angle between the axial direction of the magnetic unit and the outer tube, which is the maximum angle

error; D_m denotes the outer diameter of the magnetic unit; D_{out} refers to the inner diameter of the outer tube.

Therefore, the maximum angle error controlled by the magnetic unit can be expressed as:

$$\varphi_e = \arcsin\frac{D_{out}}{\sqrt{{L_m}^2 + {D_m}^2}} - \arcsin\frac{D_m}{\sqrt{{L_m}^2 + {D_m}^2}}$$

(S4-7)

Taking the parameters used in most experiments of this study as examples, specifically D_{out}=1.7 mm, D_m=1.4 mm, and L_m=5.6 mm, the calculations based on the given formula yielded a maximum angle error of 3.09°. Such a level of angle error is completely acceptable within soft robotics.

Supplementary Note 5:

Catheter-Blood Vessel Contact Analysis

In vascular interventional procedures, catheters/guidewires are used to navigate through blood vessels to reach target locations for diagnosis or treatment. A catheter typically consists of a catheter tip and a catheter body. The main cause of damage inflicted by the catheter tip on blood vessels is contact, leading to potential abrasions or even perforations of the vessel wall. Numerous studies have been conducted to address this challenge. However, comparatively less attention has been paid to the damage caused by the catheter body to blood vessels.

The damage inflicted by the catheter body on vessel walls is most likely to occur at sites of vessel curvature or branching. During catheter manipulation, movements involve both advancement and retraction, with the primary actions being pushing and pulling the catheter. At sites of vessel curvature, the corresponding state during catheter pushing is depicted in Supplementary Fig. 30A(i). When pushing the catheter, friction occurs between the catheter body and the vessel wall. Consequently, the resultant force exerted on the vessel wall is:

$$F_{f-push} = \mu F_{n1} \tag{S5-1}$$

where μ denotes the friction coefficient between the catheter body and the blood vessel, and F_{n1} represents the normal force exerted by the catheter body on the blood vessel. The normal force exerted by the catheter body on the blood vessel, i.e., the component of catheter thrust, can be expressed as

$$F_{n1} = F_{push} \cos\theta \tag{S5-2}$$

where F_{push} represents the thrust of the catheter, and θ denotes the angle between the direction of catheter movement at the point of contact with the blood vessel wall and the normal direction. Thus, the force exerted by the catheter body on the blood vessel wall during catheter propulsion can be derived as:

$$F_{f-push} = \mu F_{push} \cos\theta. \tag{S5-3}$$

Similarly, during the process of pulling the catheter, as illustrated in Supplementary Fig. 30A(ii), the force exerted by the catheter body on the blood vessel wall can be calculated using the following formula:

$$F_{f-pull} = \mu F_{n2} \tag{S5-4}$$

$$F_{n2} = F_{pull} \cos\theta \tag{S5-5}$$

$$F_{f-pull} = \mu F_{pull} \cos\theta \tag{S5-6}$$

where F_{f-pull} is the pulling force exerted by the catheter body on the blood veseel, F_{n2} represents the normal force exerted by the catheter body on the blood vessel during catheter pulling, and F_{pull} denotes the pulling force applied to the catheter.

For the two scenarios described above regarding catheter pushing and pulling (Supplementary Fig. 30A), the cross-sectional view at the point of contact is illustrated in Supplementary Fig. 30B, where the forces between the catheter body and the blood vessel are independent of gravity. However, there is another scenario depicted in Supplementary Fig. 30C, where the catheter precisely matches the morphology of the blood vessel, meaning that, when viewed along the planes intersecting the main vessel axis and branch vessel axes, there is no contact between the catheter and the vessel wall. In this case, as shown in Supplementary Fig. 30C(ii) for the C-C cross-section, the frictional force resulting from the contact between the catheter body and the vessel wall during pushing or pulling is eliminated, leaving only the frictional force generated by gravity, the magnitude of which is

$$F_{f-g} = \mu G \tag{S5-7}$$

where G represents the gravitational force acting on the portion of the catheter in contact with the blood vessel wall. Due to multiple points of contact between the catheter and the vessel wall, let N denote the number of contact points. Therefore, the gravitational force on the catheter at these points is given by

$$G = mg/N \tag{S5-8}$$

where m represents the total mass of the catheter. Therefore, the force exerted by the catheter body on the blood vessel wall at that point is given by

$$F_{f-g} = \mu mg/N. \quad \text{(S5-9)}$$

Due to the flexible nature of the catheter, when it is pushed, it bends (forming waves) inside the blood vessel, as illustrated in Supplementary Fig. 30A(i). If the pushing force increases, the catheter will bend more inside the vessel, and a portion of the work done by the external pushing force will be converted into the catheter's strain potential energy, unable to be entirely transmitted to the vessel wall. However, when the catheter is pulled, as depicted in Supplementary Fig. 30A(ii), it does not form waves inside the vessel. Therefore, the work done by the pulling force is not converted into the catheter's strain potential energy but is instead transmitted to the vessel wall. Therefore, assuming equal magnitudes of pushing and pulling forces on the catheter at locations where the blood vessel bends, the friction generated by the pulling force exceeds that generated by the pushing force.

We are now comparing the effects of pushing the catheter and the force caused by gravity on the blood vessel wall. According to (*71*), the minimum operating force is 0.29N, while the maximum operating force can reach up to 40N under extreme conditions. Based on (*72*, *20*, *17*, *19*), the typical operating force ranges from 2 to 3.4N. As a conservative estimate, we choose the minimum operating force of 2N as the pushing force. During the process of pushing the catheter forward, as shown in Supplementary Fig. 30A(i), the angle between the catheter and the blood vessel wall axis roughly equals the angle of the blood vessel branch. Assuming the angle of the blood vessel branch is 30°, then θ is 60°. Assuming the friction coefficient between the catheter and the blood vessel wall is 0.2, the force exerted by the catheter on the blood vessel wall is $F_{f-push} = 0.2 \times 2 \times \cos 60\,°$=0.2N.

Using a commonly used 7 French catheter (WAIN-FBK-7S, WASAHI INTECC CO., JP) as an example, with a length of 900mm and a weight of approximately 4.9g. When the catheter is fully adhered to the blood vessel, there will be numerous contact points between the catheter and the vessel. To provide a conservative estimate, let's

consider an extreme scenario where we assume the catheter only has three contact points with the vessel: the catheter tip, tail, and a midpoint, leaving approximately 450mm of the catheter suspended on each side, resulting in N=3. Thus, the force exerted by the catheter on the blood vessel wall is $F_{f-g} = 0.2 \times 0.0049 \times 9.8/3$ $=3.2\times10^{-3}$N。

Based on the above analysis, even under our conservative estimate using the most extreme scenario, the friction induced by the pushing force is 62.5 times greater than that induced by gravity. In other words, the frictional force caused by gravity will be significantly smaller than that caused by pulling and pushing. Furthermore, as the catheter moves within the blood vessel, the vessel wall must counteract its weight, rendering gravity's effect unavoidable. Therefore, among the forces exerted by the catheter on the vessel, the scenario depicted in Supplementary Fig. 30C represents the ideal condition. In this scenario, the force exerted by the catheter on the vessel wall is minimized. From the perspective of cross-sections along the main vessel axis and branch vessel axes (Supplementary Fig. 30C), there is no contact between the catheter and the vessel walls on either side.

Supplementary Note 6:

Experimental Analysis of Tube Manipulation

The real-time in-situ magnetization reprogramming proposed in this study is achieved by dynamically altering the relative positions of magnetic units, a process that relies on the relative motion between tubes. Here, we present an experimental analysis of tube manipulation, which provides empirical data to support the design and improvement discussions in Supplementary Note 9 and serves as a reference for specific design implementations when considered alongside Supplementary Note 9. Since the manipulations in 1D, 2D, and 3D magnetization reprogramming are similar, we focus our analysis solely on tube manipulation in the 1D case.

1. Experimental Setup

Achieving relative motion between tubes requires overcoming the relative motion resistance, $F_{m-total}$ (for detailed analysis, please refer to Supplementary Note 9). The factors affecting $F_{m-total}$ include the tube curvature, the number of bends, the clearance between the tubes, the tube material, the mode of relative motion between the tubes, and the lubrication method. Therefore, we designed six experiments to quantify the impact of these factors on $F_{m-total}$: **Experiment I:** Effect of the radius of curvature; **Experiment II:** Impact of the number of bends; **Experiment III:** Influence of the clearance between tubes; **Experiment IV:** Effect of the tube material's elastic modulus; **Experiment V:** Influence of tube motion mode; **Experiment VI:** Impact of the lubrication method.

In Experiment I, which assesses the impact of the radius of curvature on the relative motion resistance, we simplified the analysis due to the irregular nature of the deformation. We used a circular arc with a specific radius of curvature as an approximation. As shown in Supplementary Fig. 64A, we designed an experimental model featuring circular cavities with various pathways. These circular cavities have a diameter of 2.9mm. The model's cross-section consists of a semi-circular arc connected to two straight segments, simulating the deformation of the tube. When the

integrated tube (the inner rod is positioned within the outer tube) is placed into the model cavity, it assumes a specific shape, thereby simulating the deformations it would undergo in a magnetic field. To achieve various deformations, we established circular arcs with differing radii (Supplementary Fig. 64A), where "∞" indicates that the circular arc degenerates into a straight line. During the experiment, the integrated tube was placed within the cavity of the model. The outer tube was fixed to the model, which is attached to the ground. The inner rod was then pulled along points M, N, and P. We utilized a Universal Testing System (Model 5942, INSTRON, USA) to measure the forces (Supplementary Fig. 70B), with each experiment being repeated three times. The dimensional parameters of the outer and inner rods are identical to those of the tubes in Configuration A of the 1D magnetization reprogramming (Supplementary Fig. 3). Both the tubes and rods were fabricated using PDMS with a base-to-curing agent ratio of 20:1.

In Experiment II, which investigates the impact of the number of bends on relative motion resistance, we designed a model featuring multiple bends. This model includes circular cavities with various pathways, each having a diameter of 2.9mm. The model's cross-section consists of multiple semi-circular arcs connected to straight segments. Cross-sectional views and specific dimensional parameters are shown in Supplementary Fig. 64B. We set up experiments to test shapes with 1, 2, and 3 bends: for the single-bend experiment, the integrated tube was placed along the AFG curve into the model, with the outer tube fixed, and the inner rod was then pulled along points AFG; for the experiment with two bends, the integrated tube was placed along the BEFG curve, with the outer tube fixed, and the inner rod was then pulled along points BEFG; similarly, for the experiment with three bends, the integrated tube was placed along the CDEFG curve, with the outer tube secured, and the inner rod was then pulled along points CDEFG. The forces were measured using the Universal Testing System, with each experiment conducted in triplicate. The materials and dimensional parameters of the outer tubes and inner rods are also identical to those in Experiment I.

In Experiment III, which examines the impact of the clearance between the inner and outer tubes on the relative motion resistance, we used tubes with the same parameters as those in Experiment I. The inner diameter of the outer tube was set at 1.7mm, and we used inner rods with four different outer diameters: 1.4mm, 1.5mm, 1.6mm, and 1.7mm. The model used to fix the tubes was the same as in Experiment I, featuring a cavity with a circular arc radius of 20mm. The same operational procedures from Experiment I were employed and the force measurements for each operation were recorded.

In Experiment IV, to investigate the effect of different elastic moduli of the materials on the relative motion resistance, we kept the material of the outer tube constant and fabricated the inner rod using various materials. Specifically, we prepared rods using PDMS with base-to-curing agent ratios of 20:1, 15:1, 10:1, and 3:1, respectively. Additionally, we fabricated a rod by mixing PDMS (10:1) with magnetic powder at a mass ratio of 3:1. We employed the same model as in Experiment I, selecting a cavity with a curvature radius of 10 mm for the experiments. Using the same operational procedure as in the tube curvature influence experiment, we recorded the force values for each operation, and each experiment was repeated three times.

In Experiment V, we further employed the setup from Experiment I to assess the impact of different tube motion modes on the relative motion resistance. According to the analysis in Supplementary Note 9, the tube can achieve linear motion through pushing or pulling, which results in relative motion between the tubes. Additionally, rotating the tube while pushing or pulling it can reduce the relative motion resistance. Consequently, we designed two sets of experiments. The first set was identical to Experiment I, with the tube being pulled. In the second set, the tube was both pulled and rotated. The relative rotation between the tube and rod was induced by rotating the support frame that holds the tube, with a rotational speed set at 60°/s. We used the same model from Experiment I, selecting a cavity with a radius of curvature of 10 mm. The outer tube remained the same as in Experiment I, while the inner rod was fabricated using a PDMS and magnetic powder mixture as described in Experiment IV.

Each experimental condition was measured three times, and the force data were recorded.

In Experiment VI, to investigate the impact of different lubrication methods on the relative motion resistance, we tested four lubricants: talcum powder, 85% glycerol, 40% glycerol, and water. We used the same model as in Experiment I, specifically a cavity with a radius of curvature of 10 mm. The outer tube was identical to that in Experiment I, and the inner rod was fabricated using the PDMS and magnetic particle mixture described in Experiment IV. By applying the different lubricants between the tube and the rod, we measured the force using the same methodology as in Experiment I. Each experiment was repeated three times, and the data were recorded.

2. Experimental Results and Discussion

The data measured in Experiments I and II are presented in Supplementary Fig. 65A-65B. We observed that the relative motion resistance significantly decreases as the radius of curvature increases: at a radius of curvature of 2mm, the average force was 118.2 mN; at an infinite radius of curvature (i.e., when the tube is straight), the average force was only 0.9 mN. The relative motion resistance significantly increases with the number of bends: with one bend, the average force was 24.8 mN; with three bends, it significantly increased to 243.9 mN. Therefore, both the radius of curvature and the number of bends severely impact the manipulation over the tube. Simultaneously, we observed that at smaller radii of curvature, the forces required to push and pull the tube are relatively similar. However, when the radius of curvature decreases to 2mm, it becomes impossible to push the inner rod. Consequently, pulling operations are easier than pushing: with minimal deformation, the tube can be moved either by pulling or pushing to adjust its position; yet, as the radius of curvature decreases and the number of bends increases, pushing becomes challenging, whereas pulling remains feasible. This is because the critical buckling load of the tube is generally lower than its yield force. When the yield force exceeds the relative motion resistance, the tube can be manipulated by pulling; when the critical buckling load exceeds relative motion resistance, the tube can be manipulated by pushing. A detailed

analysis, including the mechanical conditions for tube manipulation (Equation (S9-16)), can be found in Supplementary Note 9.

The results from Experiment III are illustrated in Supplementary Fig. 65C. We observed a sudden decrease in relative motion resistance as the outer diameter of the inner rod was reduced: with an outer diameter of the inner rod of 1.7mm (the same as the inner diameter of the outer tube), the average force is 38.3 mN; when the outer diameter of the inner rod is reduced to 1.6mm, the average force abruptly decreases to 5.2 mN; with outer diameter of the inner rod smaller than 1.6mm, the average force does not significantly decrease further, but remains roughly equivalent to that observed with a 1.6mm outer diameter. Our analysis suggests that when the outer diameter of the inner rod matches the inner diameter of the outer tube, additional compressive forces arise, thus increasing friction; however, when the outer diameter of the inner rod is less than the inner diameter of the outer tube, the compressive forces between them do not change significantly, hence the relative motion resistance remains similar. Therefore, in design, reducing relative motion resistance does not necessitate significantly increasing the clearance between the inner and outer tubes, allowing more flexibility in other aspects of their design.

The data from Experiment IV, as shown in Supplementary Fig. 89A, indicate that the relative motion resistance increases with an increase in the elastic modulus of the material (the elastic moduli of these materials were measured in Supplementary Fig. 62). When using PDMS with a 20:1 base-to-curing agent ratio (elastic modulus of 0.33 MPa), the relative motion resistance was 21.3 mN; in contrast, when employing a PDMS-magnetic particle mixture (elastic modulus of 12.3 MPa), the relative motion resistance reached 83.6 mN. This observation is consistent with the analysis presented in Section 1 of Supplementary Note 9, which states that a higher elastic modulus results in greater relative motion resistance, due to the increased elastic potential energy stored in the rod during bending.

Supplementary Fig. 89B shows the results from Experiment V, and it indicates that by employing a simultaneous rotation and translation method, the relative motion resistance is significantly reduced from 83.6 mN to 27.4 mN. This reduction is

attributed to the conversion of static friction between the tubes into sliding friction, thereby lowering the friction coefficient. Additionally, the rotation weakens the adhesive effects between the tubes by continuously rearranging and refreshing the local contact surfaces, which in turn reduces adhesive friction.

The data from Experiment VI, as shown in Supplementary Fig. 89C, reveal that the relative motion resistance varies considerably with different lubricants. When using talcum powder, the relative motion resistance is 83.6 mN; with 40% glycerol, it increases to 93.75 mN; and with 85% glycerol, it reaches 163.75 mN. This suggests that higher-viscosity glycerol is less effective in lubricating the tubes. When water is used as the lubricant, the relative motion resistance is 131 mN, which is intermediate between the values obtained with talcum powder and glycerol. Given that talcum powder exhibits a relatively low friction coefficient, it was chosen as the lubricant in this study. Furthermore, in practical applications, surface treatments for the tubes, such as adding a superlubricity layer, could further reduce the relative motion resistance, thereby facilitating easier control of the tubes' relative motion.

The experimental results and discussion presented above offer valuable insights for the design and manipulation of the tubes, and these findings can be extended to the design and operation of 2D sheets and 3D structures.

Supplementary Note 7:

Feasibility of 3D Manipulations Without Altering External Magnetic Fields

To demonstrate the feasibility of achieving 3D manipulations of soft robots without altering external magnetic fields using our proposed method, we first considered 1D tubular objects as the carriers, and discussed the operational scenarios for tubes with a single magnetic unit and those with multiple magnetic units, respectively. Subsequently, we extended the analysis to 2D/3D (planar/spatial) objects. For clarity and ease of discussion, the analysis is divided into three subsections: tubes with a single magnetic unit, tubes with multiple magnetic units, and 2D/3D objects.

1. Tubes with a single magnetic unit

We positioned a tube with a length of L and a radius of R, equipped with a magnetic unit at one end, vertically along the z-axis of the coordinate system. The end without a magnetic unit was fixed at the origin O of the coordinate system, and a magnetic field $\mathbf{B}$ was applied. The magnetization of the magnetic unit can be expressed as

$$\mathbf{M} = M\mathbf{a} \quad \text{(S7-1)}$$

where M is the magnitude of the magnetization, and $\boldsymbol{a}$ is the unit direction vector of magnetization. Since $\mathbf{a}$ can be adjusted in direction on the unit sphere, it can be represented using spherical coordinates as

$$\mathbf{a} = (\sin\theta\cos\varphi\,, \sin\theta\sin\varphi\,, \cos\theta) \quad \text{(S7-2)}$$

where θ is the angle between the magnetization unit vector of the magnetic unit and the z-axis, and φ means the angle between the projection of the magnetization unit vector of the magnetic unit in the xoy plane and the x-axis.

Under the applied magnetic field, the magnetic unit experiences a force or torque, with the force density given by

$$\mathbf{f} = \mu_0(\mathbf{M} \cdot \nabla)\mathbf{B}(\mathbf{r}) \tag{S7-3}$$

where μ_0 is the permeability of free space,

and $\mathbf{B}(\mathbf{r})$ is the magnetic field strength at position $\mathbf{r}$. Assuming the volume of the magnetic unit is V, the force exerted on the magnetic unit is given by:

$$\mathbf{F}_V = \int_V \mathbf{f} \mathrm{d}V \tag{S7-4}$$

The magnetic moment of the magnetic unit can be obtained using the following formula:

$$\mathbf{m} = \int_V \mathbf{M} \, \mathrm{d}V \tag{S7-5}$$

The torque exerted on the magnetic unit can be expressed as

$$\boldsymbol{\tau}_\mathrm{V} = \mathbf{m} \times \mathbf{B} \tag{S7-6}$$

If the applied magnetic field is uniform and the magnetization of the magnetic unit is assumed to be uniformly distributed throughout its volume, then, since $\nabla\mathbf{B} = 0$ in a uniform magnetic field, the force exerted on the magnetic unit, according to Equations (S7-3) and (S7-4), is given by

$$\mathbf{f} = \mu_0(\mathbf{M} \cdot \nabla)\mathbf{B}(\mathbf{r}) = 0 \tag{S7-7}$$

$$\mathbf{F}_V = \int_V \mathbf{f} \mathrm{d}V = 0 \tag{S7-8}$$

This indicates that no net force acts on the magnetic unit in a uniform magnetic field. According to Equations (S7-1), (S7-5), and (S7-6), the torque exerted on the magnetic unit can be expressed as

$$\boldsymbol{\tau}_\mathrm{V} = MV(\mathbf{a} \times \mathbf{B}) \tag{S7-9}$$

If the applied magnetic field is a gradient field, the force exerted on the magnetic unit can be expressed as

$$\mathbf{F}_\mathrm{V} = \mu_0 \int_V (\mathbf{M} \cdot \nabla)\mathbf{B}(\mathbf{r})\mathrm{d}V \tag{S7-10}$$

Since $\nabla\mathbf{B} \neq 0$ in a gradient field, a net force acts on the magnetic unit. Furthermore, the exerted force induces a torque, which is given by

$$\boldsymbol{\tau}_\mathbf{V} = \int_V \mathbf{r} \times \mathbf{f}\, \mathrm{d}V \tag{S7-11}$$

Substituting the equation for force density, we obtained:

$$\boldsymbol{\tau}_\mathbf{V} = \mu_0 \int_V \mathbf{r} \times [(\mathbf{M} \cdot \nabla)\mathbf{B}(\mathbf{r})]\, \mathrm{d}V \tag{S7-12}$$

Under the influence of forces and torques, the tube undergoes deformation in the form of stretching, bending, and twisting. Since the effect of magnetic forces on stretching deformation is limited, bending deformation is more commonly utilized in the field of robotics for 1D tubes to achieve various functions and applications. Therefore, we will focus our analysis on bending deformation in the following discussion.

Assuming that one end of the tube is fixed while the other end is free, we can use the cantilever beam model to calculate its deformation under torque. The deflection of the tube under the applied torque is given by:

$$\frac{\mathrm{d}^2 w(r)}{\mathrm{d}z^2} = -\frac{\boldsymbol{\tau}(r)}{EI}$$

(S7-13)

$$w(r) = (w_x(r), w_y(r), w_z(r)) \tag{S7-14}$$

where, $w(r)$ represents the deflection at position r along the tube, where the magnetic unit is located,

$\boldsymbol{\tau}(r)$ denotes the torque applied at position r (i.e., on the magnetic unit),

E is the elastic modulus of the tube,

and I is the second moment of area (area moment of inertia) of the tube's cross-section.

Based on the above analysis, under a uniform magnetic field, substituting the torque expression in a uniform magnetic field into the deflection equation, the deflection of the tube is given by:

$$\frac{\mathrm{d}^2 w(\mathrm{r})}{\mathrm{d}z^2} = -\frac{MV(\mathbf{a} \times \mathbf{B})}{EI}$$

(S7-15)

Similarly, under a gradient magnetic field, the deflection of the tube due to the torque generated by the field gradient can be written as:

$$\frac{\mathrm{d}^2 w(\mathrm{r})}{\mathrm{d}z^2} = -\frac{\mu_0 M \int_V \mathbf{r} \times [(\mathbf{a} \cdot \nabla)\mathbf{B}(\mathbf{r})]\, \mathrm{d}V}{EI}$$

(S7-16)

Since one end of the tube is fixed, boundary conditions apply at the fixed end are:

$$w(0) = 0 \tag{S7-17}$$

$$\left.\frac{\mathrm{d}w}{\mathrm{d}z}\right|_{z=0} = 0$$

(S7-18)

Substituting this into the deflection equation allows us to obtain the deflection of the tube at the position of the magnetic unit. Given that the unit vector **a** is expressed as $\mathbf{a} = (\sin\theta\cos\varphi, \sin\theta\sin\varphi, \cos\theta)$, it is possible to achieve different magnetization directions by adjusting the angles θ and φ.

Furthermore, the proposed method allows for the adjustment of both the magnetization strength of each magnetic unit (by varying the effective volume V) and the position of the magnetic units. By substituting different orientations of **a** or different values of V into the deflection equations derived earlier, the deflection of the tube along the *x*, *y*, and *z* directions can be obtained under a constant magnetic field, whether in a uniform or gradient magnetic field. This enables controlled three-dimensional deformation of the tube. By incorporating variations in the positions of the magnetic units, a wider range of deformations can be achieved. This further demonstrates the feasibility of realizing 3D manipulation under a constant magnetic field.

2. Tubes with multiple magnetic units

We extend the number of magnetic units in the tube from one to *N*. While keeping the magnetic field constant, we will demonstrate the feasibility of 3D

manipulation. Similar to the previous analysis of a single magnetic unit, when the tube contains *N* magnetic units, the magnetization strength of the *i*-th magnetic unit is expressed as:

$$\mathbf{M}_i = M_i \mathbf{a}_i \tag{S7-19}$$

where M_i is the magnetization strength of the *i*-th magnetic unit,

and $\mathbf{a}_i$ means the unit direction vector of the *i*-th magnetic unit, which can be expressed in spherical coordinates as:

$$\mathbf{a}_i = (\sin\theta_i \cos\varphi_i, \sin\theta_i \sin\varphi_i, \cos\theta_i) \tag{S7-20}$$

where θ_i represents the angle between the magnetization unit vector of the *i*-th magnetic unit and the *z*-axis, and φ_i is the angle between the projection of the magnetization unit vector of the *i*-th magnetic unit in the *xoy* plane and the *x*-axis.

Under a uniform magnetic field, the torque experienced by the *i*-th magnetic unit is given by

$$\boldsymbol{\tau}_{Vi} = M_i V_i (\mathbf{a}_i \times \mathbf{B}) \tag{S7-21}$$

where **B** represents the strength of the uniform magnetic field. The total torque acting on the entire tube is then determined by:

$$\boldsymbol{\tau}_{\text{total}} = \sum_{1}^{N} M_i V_i (\mathbf{a}_i \times \mathbf{B}) \tag{S7-22}$$

Substituting this into the deflection equation, the deflection at the end of the tube is given by:

$$\frac{\mathrm{d}^2 w(r)}{\mathrm{d}z^2} = -\frac{1}{EI} \sum_{1}^{N} M_i V_i (\mathbf{a}_i \times \mathbf{B}) \tag{S7-23}$$

We can conclude that by adjusting the angles θ_i and φ_i of each magnetic unit, or by modifying the magnetization strength of each unit (i.e., by changing the effective volume), it is possible to achieve different deflections at the tube's end in various

directions without altering the applied magnetic field. This is analogous to the case of a single magnetic unit. Furthermore, the method proposed in this paper allows for modifications to the relative positions of individual magnetic units. Since the deformation of the tube depends not only on the magnitude of the net torque but also on the spatial distribution of the magnetic units, a broader range of deflections with varying magnitudes and directions can also be achieved.

We only demonstrated the case under a uniform magnetic field. Similar to the case of a single magnetic unit, in a gradient field, the total torque is also the superposition of individual torques. Since this conclusion holds for the gradient field as well, we did not provide further proof.

3. 2D/3D objects

We extended the above analysis of a 1D structure to 2D and 3D cases. We began by analyzing the 2D case, taking a sheet as a representative example. The sheet has a length L, a width B, and a thickness t. For simplification, we assumed the thickness is negligible, i.e.,

$$t \ll L, B \tag{S7-24}$$

Magnetic units are randomly distributed on the surface of the sheet. Similar to the deformation analysis of a tube with multiple magnetic units, the magnetization and unit direction vector of each magnetic unit are given by:

$$\mathbf{M}_i = M_i \mathbf{a}_i \tag{S7-25}$$

$$\mathbf{a}_i = (\sin\theta_i \cos\varphi_i, \sin\theta_i \sin\varphi_i, \cos\theta_i) \tag{S7-26}$$

where M_i represents the magnitude of the magnetization of the i-th magnetic unit, $\mathbf{a}_i$ means the magnetization unit vector of the i-th magnetic unit, θ_i represents the angle between the magnetization unit vector of the i-th magnetic unit and the z-axis, and φ_i is the angle between the projection of the magnetization unit vector of the i-th magnetic unit in the *xoy* plane and the x-axis.

The position of the i-th magnetic unit can be expressed as

$$\mathbf{r}_i = (x_i, y_i, z_i) \tag{S7-27}$$

Under a uniform magnetic field, the torque acting on the i-th magnetic unit is given by:

$$\boldsymbol{\tau}_{Vi} = M_i V_i(\mathbf{a}_i \times \mathbf{B}) \tag{S7-28}$$

In the Kirchhoff-Love thin plate theory, only the torque components perpendicular to the plate directly contribute to its bending deformation. Therefore, we defined the equivalent external load induced by the distribution of magnetic torques as:

$$q(\mathbf{r}) = \sum_{i=1}^{N} (\boldsymbol{\tau}_{Vi} \cdot \mathbf{t})\, \delta(\mathbf{r} - \mathbf{r}_i) \tag{S7-29}$$

where $\mathbf{t}$ is the unit vector in the thickness direction of the sheet,

$\delta(\mathbf{r} - \mathbf{r}_i)$ means the 2D Dirac delta function, representing the localized application of torque at position $\mathbf{r}_i$.

Substituting the magnetic torque of each unit into the above equation, we obtain:

$$q(\mathbf{r}) = \sum_{i=1}^{N} [M_i V_i(\mathbf{a}_i \times \mathbf{B}) \cdot \mathbf{t}]\, \delta(\mathbf{r} - \mathbf{r}_i) \tag{S7-30}$$

Neglecting the effects of gravity, external pressure, and other influences, and under the small deflection and linear elasticity assumptions of Kirchhoff-Love thin plate theory, the equilibrium equation of the sheet is given by:

$$D\nabla^4 w(\mathbf{r}) = q(\mathbf{r}) \tag{S7-31}$$

$$D = \frac{Et^3}{12(1 - \nu^2)} \tag{S7-32}$$

where D is the bending stiffness of the sheet,

$w(\mathbf{r})$ represents the deflection of the sheet in the thickness direction,

and ν is the Poisson’s ratio.

The equilibrium equation above can be transformed from the spatial domain to the frequency domain to facilitate the solution. First, applying the Fourier transform, solving the equation in the frequency domain, and then performing the inverse Fourier

transform, we obtain the solution in the spatial domain. The deflection corresponding to the i-th magnetic unit can be expressed as:

$$w_i(\mathbf{r}) = \frac{M_i V_i (\mathbf{a}_i \times \mathbf{B}) \cdot \mathbf{t}}{D} G(\mathbf{r}, \mathbf{r}_i)$$

(S7-33)

where $G(\mathbf{r}, \mathbf{r}_i)$ is the biharmonic Green's function, which can be obtained using the following equation:

$$G(\mathbf{r}, \mathbf{r}_i) = \frac{1}{8\pi} |\mathbf{r} - \mathbf{r}_i|^2 ln |\mathbf{r} - \mathbf{r}_i| \quad \text{(S7-34)}$$

When the sheet contains only a single magnetic unit, the deflection induced by this unit is given by Equation (S7-33). Since the unit magnetization direction is expressed as $\mathbf{a}_i = (\sin\theta_i \cos\varphi_i, \sin\theta_i \sin\varphi_i, \cos\theta_i)$, it is possible to achieve different magnetization orientations by adjusting the angles θ_i and φ_i. Additionally, the effective volume V_i of the magnetic unit can be modified to control the magnetization strength.

By substituting different unit magnetization directions $\mathbf{a}_i$ or different magnetization strengths (via varying V_i) into the deflection equation while keeping the magnetic field constant, different deflections can be obtained. This results in 3D deformation of the sheet, thereby demonstrating the feasibility of achieving 3D manipulation under a constant magnetic field. Furthermore, this method also allows for the adjustment of the position $\mathbf{r}_i$ of the magnetic unit. Changing the position of the magnetic unit affects the location where the torque is applied, leading to different deformation patterns.

When the sheet contains N magnetic units, the total torque can be obtained by summing the torques of all magnetic units. Substituting this total torque into the deflection equation, the deflection of the sheet is given by:

$$w(\mathbf{r}) = \frac{1}{D} \sum_{i=1}^{N} [M_i V_i (\mathbf{a}_i \times \mathbf{B}) \cdot \mathbf{t}]\, G(\mathbf{r}, \mathbf{r}_i)$$

(S7-35)

In the Equation (S7-35), similar to the analysis of a sheet with a single magnetic unit, a sheet with multiple magnetic units can also achieve 3D deflection variations by adjusting the parameters, all while keeping the magnetic field constant.

So far, we only demonstrated the case under a uniform magnetic field. Similar to the 1D tube with a single magnetic unit, the total torque is also the superposition of individual torques in a gradient field. Since this conclusion also holds for the gradient field, we did not provide further proof.

Similarly, the manipulation of a 2D sheet can be extended to 3D objects, and the same conclusion applies. One of the simplest examples is stacking two sheets in a non-parallel configuration, forming a 3D structure. Since each sheet can independently achieve 3D deformation under a constant external magnetic field, the combined 3D object can also exhibit 3D deformation under the same conditions. Therefore, we did not further discuss the case of 3D objects.

The above analysis demonstrates the feasibility of 3D operation without altering external magnetic fields. Furthermore, if the external magnetic field can be varied, the proposed method would enable even more complex 3D deformations. In Supplementary Note 8, we have shown that the force-torque space generated by the proposed method, when combined with a variable magnetic field, fully encompasses the force-torque space achievable by existing methods. This implies that existing methods are a subset of the method introduced in this study (as illustrated in Supplementary Fig. 71).

Supplementary Note 8:

Force-Torque Space Analysis

To compare the manipulation capabilities of the proposed method with those of existing method for soft robotics, we analyzed their respective force-torque spaces, thereby theoretically demonstrating their maneuverability. The existing method—wherein only the external magnetic field is altered while the robot's magnetization profile remains unchanged—is designated as **Method 1**. For the proposed approach that employs a static magnetic field (i.e., where no alteration of the magnetic field occurs during the operation), with only the robot's magnetization curve being modified, it is designated as **Method 2**. For the proposed approach that utilizes a tunable magnetic field (i.e., one that can be adjusted as required), it is referred to as **Method 3**, whereby both the external magnetic field and the robot's magnetization profile can be modified. To simplify the analysis, we initially examined the scenario under a uniform magnetic field, and subsequently extended the analysis to the case of a general magnetic field.

1. Spatial representation

We assume that the magnetic soft robot is situated in the 3D Euclidean space $\mathbb{R}^3$ and is composed of n magnetic units, where $n \geq 1$. Each magnetic unit possesses a magnetic moment, denoted by $\mathbf{m}_i$. When the robot is subjected to a uniform magnetic field, the ith magnetic unit experiences only a torque, given by

$$\boldsymbol{\tau}_i = \mathbf{m}_i \times \mathbf{B} \quad \text{(S8-1)}$$

where $\mathbf{B}$ represents the magnetic field. Since the magnetic robot comprises n magnetic units, its torques can be expressed as

$$(\boldsymbol{\tau}_1, \boldsymbol{\tau}_2, \dots, \boldsymbol{\tau}_n) \quad \text{(S8-2)}$$

Thus, the overall torque of the robot is distributed in the space $\mathbb{R}^{3n}$.

For Method 1, since the magnetization profile (i.e., $\mathbf{m}_i$) is maintained constant while the external magnetic field (i.e., $\mathbf{B} \in \mathbb{R}^3$) is varied freely, the torque

experienced by the robot is computed using equation (S8-1). Consequently, the set of torque distributions (torque space) is given by

$$\mathrm{S}_1 = \{(\boldsymbol{\tau}_1, \dots, \boldsymbol{\tau}_n) \mid \exists \mathbf{B} \in \mathbb{R}^3, \boldsymbol{\tau}_i = \mathbf{m}_i \times \mathbf{B}\ (\forall i)\} \tag{S8-3}$$

The torque distribution space S_1 contains only those torque combinations that can be generated by some magnetic field **B**, and it does not span the entire space $\mathbb{R}^{3n}$. We define a mapping

$$F\colon\ \mathbb{R}^3 \to \mathbb{R}^{3n}, F(\mathbf{B}) = (\mathbf{m}_1 \times \mathbf{B}, \dots, \mathbf{m}_n \times \mathbf{B}) \tag{S8-4}$$

Since $\mathbf{m}_1 \times \mathbf{B}$ is linear, the mapping F is also linear and its image is given by

$$\mathrm{S}_1 = \mathrm{Im}\ (F) \tag{S8-5}$$

Here, S_1 represents the set of all possible vectors that F can output. Because the magnetic field **B** has only three independent components, it follows that

$$\dim(\mathrm{Im}\ (F)) \leq 3 \tag{S8-6}$$

Thus, the torque space generated by Method 1 can realize all torque distributions within S_1 in the space $\mathbb{R}^{3n}$, but it is at most a 3D subspace. The specific dimension depends on the linear independence of the magnetic moments $\mathbf{m}_i$.

For Method 2, since the robot's magnetization profile are varied while the external magnetic field remains constant, the torques acting on the robot are computed using Equation (S8-1). Thus, the torque distribution set (torque space) is defined as

$$S_2 = \{(\boldsymbol{\tau}_1, \dots, \boldsymbol{\tau}_n) \mid \exists (\mathbf{m}'_1, \dots, \mathbf{m}'_n) \in (\mathbb{R}^3)^n,\ \boldsymbol{\tau}_i = \mathbf{m}'_i \times \mathbf{B}_0\ (\forall i)\} \tag{S8-7}$$

In this formulation, the robot's magnetic moment $\mathbf{m}_i$ can be arbitrarily adjusted in both magnitude and direction, and is denoted by $\mathbf{m}'_i$. The fixed, nonzero magnetic field is represented by $\mathbf{B}_0$, where $\mathbf{B}_0 \in \mathbb{R}^3 \setminus \{0\}$. Although any $\mathbf{m}'_i$ can be selected to achieve a desired torque combination, the nature of the cross product imposes constraints such that S_2 does not span the entire $\mathbb{R}^{3n}$ space.

Since the cross product of two vectors always yields a vector perpendicular to both, the torque experienced by each magnetic unit must be orthogonal to the magnetic field. This condition is expressed as

$$\boldsymbol{\tau}_i \cdot \mathbf{B_0} = (\mathbf{m}_i' \times \mathbf{B_0}) \cdot \mathbf{B_0} = 0 \tag{S8-8}$$

Thus, the torque space S_2 can be formulated as:

$$S_2 = \{(\boldsymbol{\tau}_1, \dots, \boldsymbol{\tau}_n) \mid \boldsymbol{\tau}_i \cdot \mathbf{B_0} = 0 \quad (\forall i)\} \tag{S8-9}$$

In 3D space, if the magnetic field $\mathbf{B_0}$ is fixed, then every torque $\boldsymbol{\tau}_i$ must lie within the 2D plane perpendicular to $\mathbf{B_0}$. In other words, we can independently adjust each magnetic moment $\mathbf{m}_i'$ to control the corresponding component of $\boldsymbol{\tau}_i$ within that plane. Consequently, each $\boldsymbol{\tau}_i$ has two independent degrees of freedom, and with n independent magnetic units, the robot can generate n independent torques. Thus, the overall torque space S_2 forms a $2n$-dimensional linear subspace, i.e.,

$$\dim(S_2) = 2n \tag{S8-10}$$

For Method 3, where both the robot's magnetic moments and the external magnetic field are variable, the torque acting on the robot is again computed using Equation (S8-1). Therefore, the torque distribution set (torque space) is defined as

$$S_3 = \{(\boldsymbol{\tau}_1, \dots, \boldsymbol{\tau}_n) \mid \exists \mathbf{B} \in \mathbb{R}^3, \exists (\mathbf{m}_1', \dots, \mathbf{m}_n'),\ \boldsymbol{\tau}_i = \mathbf{m}_i' \times \mathbf{B},\ \forall i\} \tag{S8-11}$$

Here, we can arbitrarily choose the magnetic field $\mathbf{B}$ and the magnetic moment $\mathbf{m}_i'$ to achieve any desired combination of torques. However, due to the inherent properties of the cross product, S_3 does not span the entire $\mathbb{R}^{3n}$ space. Since each $\mathbf{m}_i'$ can be independently selected, yet the resulting torque $\boldsymbol{\tau}_i$ is always orthogonal to $\mathbf{B}$, the torque for each magnetic unit is confined to a fixed 2D plane. Therefore, S_3 can be equivalently expressed as

$$S_3 = \{ (\boldsymbol{\tau}_1, \dots, \boldsymbol{\tau}_n) \mid \exists \mathbf{B} \neq 0,\ \boldsymbol{\tau}_i \cdot \mathbf{B} = 0 \quad (\forall i) \} \tag{S8-12}$$

Because the magnetic field $\mathbf{B}$ can be varied as needed, different 2D planes can be obtained by selecting different magnetic fields. For any two torque vectors $\boldsymbol{\tau}_m$ and $\boldsymbol{\tau}_n$

in S_3 ($\boldsymbol{\tau}_{\boldsymbol{m}} \in \mathrm{S}_3$, $\boldsymbol{\tau}_{\boldsymbol{n}} \in \mathrm{S}_3$), which are generated under magnetic fields $\mathbf{B_m}$ and $\mathbf{B_n}$ respectively, the fact that $\boldsymbol{\tau}_{\boldsymbol{m}}$ and $\boldsymbol{\tau}_{\boldsymbol{n}}$ lie on two distinct 2D planes implies that their linear combination will generally not lie in any single plane, i.e.,

$$\alpha\boldsymbol{\tau}_{\boldsymbol{m}} + \beta\boldsymbol{\tau}_{\boldsymbol{m}} \notin \mathrm{S}_3 \tag{S8-13}$$

Thus, S_3 is not a linear subspace but rather the union of all 2D planes corresponding to all possible orientations of the magnetic field **B**.

2. Spatial relationships

Regarding the relationships among the torque spaces generated by the three methods, we first compare Methods 1 and 2, and then examine their relationship with Method 3. For the torque space S_1 generated by Method 1, based on the analysis above, since the torques are produced under the same magnetic field **B**, S_1 is at most three-dimensional—thus representing a low-dimensional subspace in $\mathbb{R}^{3n}$. In contrast, the torque space S_2 generated by Method 2 is a $2n$-dimensional subspace. When $n \geq 2$, we have $\dim(S_2) \geq 4$, while $\dim(S_1) \leq 3$.

2.1 Relationship between the torque spaces generated by methods 1 and 2

For the intersection of the torque spaces corresponding to Methods 1 and 2, based on the analysis above, we define the following set:

$$S_1 \cap S_2 = \{ (\boldsymbol{\tau}_1, \ldots, \boldsymbol{\tau}_n) \mid \exists \mathbf{B},\ \boldsymbol{\tau}_i = \mathbf{m}_i \times \mathbf{B}, \boldsymbol{\tau}_i \cdot \mathbf{B_0} = 0,\ \forall i \} \tag{S8-14}$$

This set is equivalent to

$$S_1 \cap S_2 = \{ (\mathbf{m_1} \times \mathbf{B}, \ldots, \mathbf{m}_n \times \mathbf{B}) \mid \mathbf{B} \in \mathbb{R}^3, (\mathbf{m}_i \times \mathbf{B}) \cdot \mathbf{B_0} = 0, \forall i \} \tag{S8-15}$$

The intersection $S_1 \cap S_2$ comprises those torque distributions that can be generated by a magnetic field **B** while also being required to be orthogonal to $\mathbf{B_0}$. According to the properties of vector operations:

$$(\mathbf{m}_i \times \mathbf{B}) \cdot \mathbf{B_0} = (\mathbf{B_0} \times \mathbf{m}_i) \cdot \mathbf{B} \tag{S8-16}$$

Therefore, the magnetic field **B** must lie within span $(\mathbf{B_0} \times \mathbf{m}_i)$.

When $\dim(\text{span}(\mathbf{B_0} \times \mathbf{m}_i)) \leq 2$, the vector $\mathbf{B_0} \times \mathbf{m}_i$ spans at most a 2D plane. In such a 2D space, one can always choose a magnetic field $\mathbf{B}$ that is perpendicular to this plane, thereby satisfying $(\mathbf{B_0} \times \mathbf{m}_i) \cdot \mathbf{B} = 0$. Under this condition, the intersection $S_1 \cap S_2$ is nonempty, i.e., $S_1 \cap S_2 \neq \emptyset$.

In contrast, when $\dim(\text{span}(\mathbf{B_0} \times \mathbf{m}_i)) \geq 3$—for instance, when it equals 3—the vector $\mathbf{B_0} \times \mathbf{m}_i$ spans a 3D space. In that case, $\mathbf{B}$ would have to be orthogonal to every direction in this 3D space, which is only possible if $\mathbf{B} = 0$; hence, $S_1 \cap S_2 = \{0\}$.

From the analysis above, it follows that S_1 and S_2 have a nontrivial (nonzero) intersection under certain conditions. We now proceed to discuss their inclusion relationship. Let us first assume that $S_1 \subseteq S_2$, that is, every torque distribution generated by Method 1 can also be achieved by Method 2. The corresponding mathematical description is:

$$\forall\, \mathbf{B},\ \ (\mathbf{m_1} \times \mathbf{B}, \dots, \mathbf{m}_n \times \mathbf{B}) \in S_2 \tag{S8-17}$$

Since S_2 is the set of all torque distributions that are perpendicular to the fixed magnetic field $\mathbf{B_0}$, every $\mathbf{m}_i \times \mathbf{B}$ must be orthogonal to $\mathbf{B_0}$. Hence, we have:

$$(\mathbf{m}_i \times \mathbf{B}) \cdot \mathbf{B_0} = 0, \quad \forall i \tag{S8-18}$$

Based on the properties of vector operations, the above equation can be rewritten as:

$$(\mathbf{m}_i \times \mathbf{B}) \cdot \mathbf{B_0} = (\mathbf{B_0} \times \mathbf{m}_i) \cdot \mathbf{B} \tag{S8-19}$$

According to equations (S8-18) and (S8-19), in order for the condition to hold for all magnetic fields $\mathbf{B}$, it is necessary that:

$$\mathbf{B_0} \times \mathbf{m}_i = \mathbf{0} \tag{S8-20}$$

Thus, it follows that either $\mathbf{m}_i \parallel \mathbf{B_0}$ or $\mathbf{m}_i = \mathbf{0}$. Since a magnetic soft robot must be magnetic, not all $\mathbf{m}_i$ can be zero simultaneously. Based on the above analysis, for $S_1 \subseteq S_2$ to hold, it is required that:

$$\mathbf{m}_i \parallel \mathbf{B_0}, \quad \forall i \tag{S8-21}$$

In other words, $S_1 \subseteq S_2$ occurs if and only if all $\mathbf{m}_i$ are parallel to $\mathbf{B_0}$. If at least one $\mathbf{m}_i$ is not parallel to $\mathbf{B_0}$, then some torque distributions generated by Method 1 will not lie in S_2, i.e., $S_1 \nsubseteq S_2$.

Similarly, let us assume that $S_2 \subseteq S_1$, i.e., every torque distribution generated by Method 2 can also be achieved by Method 1. According to the spatial description and discussion in the previous section in this Note, Method 1 generates torque distributions by altering the external magnetic field, and its dimension is

$$\dim(S_1) \leq 3 \tag{S8-22}$$

whereas the torque space generated by Method 2 has dimension

$$\dim(S_2) = 2n \tag{S8-23}$$

In order for $S_2 \subseteq S_1$ to hold, it is necessary that

$$\dim(S_2) \leq \dim(S_1) \tag{S8-24}$$

i.e.,

$$2n \leq 3 \tag{S8-25}$$

Since n is a positive integer (representing the number of magnetic units), the inequality $2n \leq 3$ can only be satisfied when $n = 1$. Therefore, $S_2 \subseteq S_1$ holds only for $n = 1$. When $n \geq 2$, the dimension of S_2 exceeds that of S_1, which implies that $S_2 \nsubseteq S_1$.

Based on the above analysis of the intersection and inclusion relationships between S_1 and S_2, we conclude that $S_1 \cap S_2 \neq \emptyset$, $S_1 \nsubseteq S_2$, and $S_2 \nsubseteq S_1$. Thus, the relationship between S_1 and S_2 is that they partially overlap without one being a subset of the other.

2.2 Relationship between the torque spaces generated by method 3 and those generated by methods 1 and 2

According to the description of the torque spaces generated by the three methods in Section 1 in this Note, the torque space generated by Method 1 is

$$S_1 = \{(\boldsymbol{\tau}_1, \ldots, \boldsymbol{\tau}_n) \mid \exists \mathbf{B} \in \mathbb{R}^3, \boldsymbol{\tau}_i = \mathbf{m}_i \times \mathbf{B}\ (\forall i)\} \tag{S8-26}$$

The torque space generated by Method 2 is

$$S_2 = \{(\boldsymbol{\tau}_1, \ldots, \boldsymbol{\tau}_n) \mid \exists (\mathbf{m}_1', \ldots, \mathbf{m}_n') \in (\mathbb{R}^3)^n,\ \boldsymbol{\tau}_i = \mathbf{m}_i' \times \mathbf{B}_0\ (\forall i)\} \tag{S8-27}$$

The torque space generated by Method 2 can be expressed as

$$S_3 = \{(\boldsymbol{\tau}_1, \ldots, \boldsymbol{\tau}_n) \mid \exists \mathbf{B} \in \mathbb{R}^3, \exists (\mathbf{m}_1', \ldots, \mathbf{m}_n'),\ \boldsymbol{\tau}_i = \mathbf{m}_i' \times \mathbf{B},\ \forall i\} \tag{S8-28}$$

For the relationship between the torque spaces S_1 and S_3, we first consider and analyze S_3. In the set S_3 (see Equation (S8-28)), since the magnetic field $\mathbf{B}$ can take any value in $\mathbb{R}^3$, we choose it to be the same variable magnetic field as in S_1. Simultaneously, we set $\mathbf{m}_i' = \mathbf{m}_i$, and then, within S_3 we have

$$\boldsymbol{\tau}_i = \mathbf{m}_i' \times \mathbf{B} = \mathbf{m}_i \times \mathbf{B},\ \forall i \tag{S8-29}$$

This demonstrates that every torque distribution generated by Method 1 can also be produced by Method 3, i.e., $S_1 \subseteq S_3$。

Similarly, for the relationship between the torque spaces S_2 and S_3, we again consider and analyze S_3. In the set S_3, since the magnetic field $\mathbf{B}$ can assume any value in $\mathbb{R}^3$, we set it to the fixed magnetic field $\mathbf{B}_0$ used in S_2. Again, let $\mathbf{m}_i' = \mathbf{m}_i$ and then, within S_3 we obtain

$$\boldsymbol{\tau}_i = \mathbf{m}_i' \times \mathbf{B} = \mathbf{m}_i \times \mathbf{B}_0,\ \forall i \tag{S8-30}$$

This shows that every torque distribution generated by Method 2 can also be achieved by Method 3, i.e., $S_2 \subseteq S_3$.

In summary, the torque spaces generated by Methods 1 and 2 can both be produced by Method 3, i.e., $S_1 \subseteq S_3$, $S_2 \subseteq S_3$.When the variable magnetic field in S_3 is set to be the same as the variable magnetic field used in S_1, and the variable magnetic moments in S_3 are fixed to be identical to those in S_1, then S_3 degenerates into S_1. Similarly, when the variable magnetic field in S_3 is set to the fixed magnetic field used in S_2, and the variable magnetic moments in S_3 are chosen to be identical to

those in S_2, then S_3 degenerates into S_2. The relationship between them can be expressed as follows:

$$S_1 \subseteq S_3, \quad S_3|_{\mathbf{m}_i'=\mathbf{m}_i} = S_1 \tag{S8-31}$$

$$S_2 \subseteq S_3, \quad S_3|_{\mathbf{B}=\mathbf{B_0}} = S_2 \tag{S8-32}$$

3. Generalization

The analysis above examined the relationships among the force-torque spaces generated by the three methods under a uniform magnetic field. We now extend the discussion to the case of a general (non-uniform) magnetic field. Similarly, we assume that the robot is equipped with n magnetic units, where the i-th magnetic unit is located at $\mathbf{r}_i \in \mathbb{R}^3$ and possesses a magnetic moment $\mathbf{m}_i \in \mathbb{R}^3$. Because a non-uniform magnetic field is influenced not only by external control but also by spatial position, we define the magnetic field at a given point as $\mathbf{B}(\mathbf{r}_i, \Psi)$, where Ψ denotes the set of externally adjustable parameters that modulate the magnitude and direction of the magnetic field. Here, $\Psi \in \mathbb{R}^k$, with k representing the number of parameters for adjusting the external magnetic field (which may include, for example, current, the number of coil turns, etc.). Under a non-uniform magnetic field, the robot experiences both forces and torques. Therefore, the force acting on the i-th magnetic unit is given by:

$$\mathbf{F}_i = (\mathbf{m}_i \cdot \nabla)\mathbf{B}(\mathbf{r}_i, \Psi) \tag{S8-33}$$

$$\boldsymbol{\tau}_i = \mathbf{m}_i \times \mathbf{B}(\mathbf{r}_i, \Psi) \tag{S8-34}$$

Thus, the force-torque distribution for the i-th magnetic unit is given by

$$(\mathbf{F}_i, \boldsymbol{\tau}_i) \in \mathbb{R}^6 \tag{S8-35}$$

The overall force-torque distribution for a soft robot comprising n magnetic units is denoted by

$$(\mathbf{F_1}, \boldsymbol{\tau_1}, \dots, \mathbf{F}_n, \boldsymbol{\tau}_n) \in \mathbb{R}^{6n} \tag{S8-36}$$

For Method 1, where only the external magnetic field **B** is varied (i.e., by altering the parameter Ψ), and analogous to the analysis for a uniform magnetic field above, we obtain that the force-torque space generated by Method 1 is given by

$$S_1' = \{(\mathbf{F_1}, \boldsymbol{\tau}_1, \dots, \mathbf{F}_n, \boldsymbol{\tau}_n) | \exists \Psi \in \mathbb{R}^k, \mathbf{F}_i = (\mathbf{m}_i \cdot \nabla)\mathbf{B}(\mathbf{r}_i, \Psi), \boldsymbol{\tau}_i = \mathbf{m}_i \times \mathbf{B}(\mathbf{r}_i, \Psi),\ \forall i\} \tag{S8-37}$$

By contrast, in Method 2, the magnetic field **B** is held constant (i.e., $\mathbf{B}(\mathbf{r}_i, \Psi_0)$) while the magnetic moments $\mathbf{m}_i'$ of each magnetic unit are varied, yielding a force-torque space given by

$$S_2' = \left\{(\mathbf{F_1}, \boldsymbol{\tau}_1, \dots, \mathbf{F}_n, \boldsymbol{\tau}_n) \middle| \begin{array}{c} \exists (\mathbf{m}_1', \dots, \mathbf{m}_n') \in (\mathbb{R}^3)^n, \\ \mathbf{F}_i = (\mathbf{m}_i' \cdot \nabla)\mathbf{B}(\mathbf{r}_i, \Psi_0), \boldsymbol{\tau}_i = \mathbf{m}_i' \times \mathbf{B}(\mathbf{r}_i, \Psi_0),\ \forall i \end{array}\right\} \tag{S8-38}$$

Similarly, in Method 3, both the external magnetic field **B** and the magnetic moments $\mathbf{m}_i'$ are adjusted, which leads to a force-torque space represented as

$$S_3' = \left\{(\mathbf{F_1}, \boldsymbol{\tau}_1, \dots, \mathbf{F}_n, \boldsymbol{\tau}_n) \middle| \begin{array}{c} \exists \Psi \in \mathbb{R}^k,\ \exists (\mathbf{m}_1', \dots, \mathbf{m}_n') \in (\mathbb{R}^3)^n \\ \mathbf{F}_i = (\mathbf{m}_i' \cdot \nabla)\mathbf{B}(\mathbf{r}_i, \Psi), \boldsymbol{\tau}_i = \mathbf{m}_i' \times \mathbf{B}(\mathbf{r}_i, \Psi),\ \forall i \end{array}\right\} \tag{S8-39}$$

Based on Equations (S8-37) and (S8-38), the intersection of the force-torque spaces generated by Methods 1 and 2 must simultaneously satisfy the following conditions:

$$(\mathbf{m}_i \cdot \nabla)\mathbf{B}(\mathbf{r}_i, \Psi) = (\mathbf{m}_i' \cdot \nabla)\mathbf{B}(\mathbf{r}_i, \Psi_0) \tag{S8-40}$$

$$\mathbf{m}_i \times \mathbf{B}(\mathbf{r}_i, \Psi) = \mathbf{m}_i' \times \mathbf{B}(\mathbf{r}_i, \Psi_0) \tag{S8-41}$$

In the equations above, $\mathbf{m}_i$ is fixed, $\mathbf{m}_i'$ is adjustable, $\mathbf{B}(\mathbf{r}_i, \Psi)$ is variable, and $(\mathbf{r}_i, \Psi_0)$ is fixed.

Since $\mathbf{B}(\mathbf{r}_i, \Psi)$ and $\mathbf{B}(\mathbf{r}_i, \Psi_0)$ can represent any magnetic field, to prove whether S_1' and S_2' have a nonempty intersection, it suffices to show that for a particular choice of $\mathbf{B}(\mathbf{r}_i, \Psi)$ and $\mathbf{B}(\mathbf{r}_i, \Psi_0)$ the intersection $S_1' \cap S_2'$ is nonempty. Assume that both $\mathbf{B}(\mathbf{r}_i, \Psi)$ and $\mathbf{B}(\mathbf{r}_i, \Psi_0)$ are uniform magnetic fields. In this case, the two methods degenerate to the scenario discussed in Section 2.1 in this Note. As we proved in Section 2.1 of this Note that Methods 1 and 2 have a nonempty intersection under a uniform magnetic field, it follows that $S_1' \cap S_2' \neq \emptyset$.

Regarding the inclusion relationship between S_1' and S_2', we first assume that $S_1' \subseteq S_2'$. This assumption implies that every force and torque in S_1' has a corresponding force and torque in S_2'. In other words, the following conditions must be simultaneously satisfied:

$$\forall\Psi,\ \forall i,\ (\mathbf{m}_i \cdot \nabla)\mathbf{B}(\mathbf{r}_i, \Psi) \in S_2' \tag{S8-42}$$

$$\forall\,\Psi,\ \forall i,\ \mathbf{m}_i \times \mathbf{B}(\mathbf{r}_i, \Psi) \in S_2' \tag{S8-43}$$

If either of these conditions fails, then it necessarily follows that $S_1' \nsubseteq S_2'$. Because the magnetic field can be chosen arbitrarily, we set it as a uniform magnetic field. Under this assumption, the above requirement reduces to the sole condition given by Equation (S8-43). In Section 2.1 in this Note, we demonstrated that under a uniform magnetic field (i.e., when only considering Equation (S8-43)), the torque space generated by Method 1 is not a subset of that generated by Method 2. Therefore, even when considering only Equation (S8-43), it holds that $S_1' \nsubseteq S_2'$, and consequently, when both Equations (S8-42) and (S8-43) are taken into account, we must have $S_1' \nsubseteq S_2'$.

Similarly, using Section 2.1 in this Note as a special case, we can also prove that $S_2' \nsubseteq S_1'$. In summary, given that $S_1' \cap S_2' \neq \emptyset$, $S_1' \nsubseteq S_2'$, and $S_2' \nsubseteq S_1'$, we conclude that the relationship between S_1' and S_2' is characterized by partial overlap, with neither space being a subset of the other.

Regarding the relationship between S_1' and S_3', we prove as follows. In Method 1, $(\mathbf{F_1}, \boldsymbol{\tau}_1, \ldots, \mathbf{F}_n, \boldsymbol{\tau}_n) \in S_1'$, since S_1' is achieved by adjusting the external magnetic field, there exists a Ψ^* such that for all magnetic units,

$$\mathbf{F}_i = (\mathbf{m}_i \cdot \nabla)\mathbf{B}(\mathbf{r}_i, \Psi^*) \tag{S8-44}$$

$$\boldsymbol{\tau}_i = \mathbf{m}_i \times \mathbf{B}(\mathbf{r}_i, \Psi^*) \tag{S8-45}$$

In Method 3, since the magnetic field can be chosen arbitrarily, we select the same varying magnetic field as in Method 1, i.e., $\Psi = \Psi^*$; moreover, because the magnetic moments in Method 3 are also adjustable, we choose them to be identical to those in Method 1, i.e., $\mathbf{m}_i' = \mathbf{m}_i$. Under these selections, all forces and torques achievable by

Method 1 (i.e., Equations (S8-44) and (S8-45)) can also be produced by Method 3. The force-torque space S_1' obtained by Method 1 is simply a special case of the force-torque space S_3' produced by Method 3, and we conclude that $S_1' \subseteq S_3'$.

Similarly, Method 3 can be configured by choosing the same fixed magnetic field as used in Method 2, and by selecting the variable magnetic moments to be identical to those in Method 2. In this case, the force-torque space obtained by Method 2 is simply a special case of that generated by Method 3, i.e., $S_2' \subseteq S_3'$.

In summary, similar to the case of a uniform magnetic field, under a general magnetic field, the force-torque spaces generated by Methods 1 and 2 partially overlap and neither is contained within the other; moreover, the force-torque spaces produced by Methods 1 and 2 are both subsets of the force-torque space generated by Method 3; and under certain conditions, Method 3 can degenerate into either Method 1 or Method 2. Specifically, the details are as follows:

$$S_1' \subseteq S_3', \quad S_3'|_{\mathbf{m}_i'=\mathbf{m}_i} = S_1' \tag{S8-46}$$

$$S_2' \subseteq S_3', \quad S_3'|_{\mathbf{B}=\mathbf{B}_0} = S_2' \tag{S8-47}$$

The relationships among the force-torque spaces produced by the three methods are illustrated in an Euler diagram, as shown in Supplementary Fig. 71.

Supplementary Note 9:

Design and Analysis of Tube Manipulation Schemes

To facilitate the application of the proposed method across various scenarios, we will present detailed design, analysis, and optimization solutions in this Note. Since the deformation of the 1D soft tube forms the basis of this study—with deformations in 2D and 3D structures being extensions of the 1D soft tube—we focus here solely on the design of the 1D soft tube manipulation scheme. In the design of the 1D soft tube, configurations involving either a two-tube or a multi-tube nested arrangement are considered. We focus on the two-tube configuration for detailed analysis, while the multi-tube design is presented in Section 4.4 of this note.

For the manipulation of two nested soft tubes, the relative displacement between the two tubes is achieved by manipulating the terminal ends, causing the tubes to advance or retract. Although there are multiple methods to manipulate the tube, we simplify the operation by using grippers. As shown in Supplementary Fig. 74A, two grippers are employed to grasp Tube A and Tube B, respectively; they are then assembled and placed within a magnetic field to achieve the desired deformations. To clarify the discussion, we define the relevant concepts as follows. As depicted in Supplementary Fig. 74B, the effective area covered by the magnetic field is termed the workspace. Once the tubes are assembled and placed within the workspace, the zone where the tubes interact with the magnetic field and undergo deformation is defined as the Magnetic Interaction Zone, with the corresponding tube length denoted as the Magnetic Interaction Length (L_{mi}). The zone between this zone and the gripper is called the Feeding Zone, and the corresponding tube length is defined as the Feeding Length (L_f). Finally, the region where the gripper holds the tube and controls its motion is referred to as the Actuation Zone, with the spacing in this region defined as the Actuation Spacing (L_{as}).

We will individually analyze and discuss the three zones: the **Magnetic Interaction Zone**, the **Feeding Zone**, and the **Actuation Zone**. For each zone, we first conduct a mechanical analysis of the soft tube, then propose appropriate design

solutions and optimization strategies based on this analysis, and finally present a case study from our experiments as an example. After discussing these three zones, we focus on several special operations: **long-range operation**, **multi-bend deformation operation**, **high-curvature deformation operation**, and **multi-nested-tube operation** in Section 4.

1. Magnetic Interaction Zone

1.1 Mechanics Analysis

When operating the inner and outer soft tubes, the forces involved in the overlapping region (i.e., the interaction area in Supplementary Fig. 74C, as non-overlapping sections do not involve relative motion or interaction) can be categorized as follows. 1) With the outer tube stationary while pushing the inner tube (in the $+x$ direction), the outer tube experiences tensile force and the inner tube experiences compressive force. 2) With the outer tube stationary while pulling the inner tube (in the $-x$ direction), the outer tube experiences compressive force and the inner tube experiences tensile force. 3) With the inner tube stationary while pushing the outer tube (in the $+x$ direction), the inner tube experiences tensile force and the outer tube experiences compressive force. 4) With the inner tube stationary while pulling the outer tube (in the $-x$ direction), the inner tube experiences compressive force and the outer tube experiences tensile force. Since the inner and outer tubes are nested and essentially equivalent, these four cases can be reduced to an analysis of two scenarios: one tube remains fixed while the other moves, being subjected either to tensile or compressive forces.

Tensile and compressive forces are influenced by various factors, including tube bending, gravity, and friction. In addition, because the tube is located within the Magnetic Interaction Zone, its bending is also affected by magnetic forces/torques. Under the combined influence of these factors, the dynamic process of relative tube motion becomes exceedingly complex. Since the static bending profile can be calculated using the formulas provided in Supplementary Note 2, we simplify the dynamic process by modeling it as the inner tube moving along a fixed bending profile

maintained by the outer tube. Because this static profile is established under the combined effects of gravity, magnetic forces, and other external loads, it inherently accounts for these influences on the inner tube. Therefore, the magnetic forces and gravity acting on the bent segment do not require separate calculations.

Assuming that the tube undergoes only bending deformation without any torsion, and following a pseudo-rigid model, we assume that the tube is composed of n serial link segments. If the curvature of a certain segment is denoted as κ_i, then the bending moment corresponding to that segment is:

$$M_i = E_i I_i \kappa_i \tag{S9-1}$$

where E_i is the elastic modulus of that section; I_i is the area moment of inertia of that segment. Therefore, the bending moment of all the bent segments is:

$$M_{total} = \sum E_i I_i \kappa_i \tag{S9-2}$$

The force required to move the inner tube to form or eliminate a bend of the same shape as the outer tube is:

$$F_c = \frac{M_{total}}{L_e} \tag{S9-3}$$

where L_e is the equivalent lever arm.

The formation of bending is a result of the action of magnetic torque. In segments where there is no bending, there is an absence of magnetic torque. Consequently, in these non-bent segments, the only external force acting is gravity. The gravity acting on the tube primarily serves two functions: it generates resistance in the direction of the tube's end movement, and it also creates frictional force in the direction perpendicular to the tube. The force exerted by gravity in the direction of the tube's end movement is:

$$F_e^G = \sum m_i^m \mathbf{g} \cdot \mathbf{s}_e \tag{S9-4}$$

where m_i^m represents the mass per unit length of the tube; $\mathbf{g}$ is the acceleration due to gravity; $\mathbf{s}_e$ is the unit directional vector in the direction of the tube's end movement.

When the inner and outer tubes move relative to each other, frictional force is generated. The total friction force F^f, is given by the sum of the friction forces acting on each individual unit. The frictional force experienced by the ith segment is:

$$F_i^f = \mu_i N_i \tag{S9-5}$$

where μ_i is the coefficient of friction corresponding to the ith segment;
N_i means the normal force corresponding to the ith segment.
In the non-bent segments, the normal force is the component of gravity in the direction perpendicular to the movement; in the bent segments, this normal force arises from the compressive force due to the bending of the inner tube. Thus there is

$$N_{nb} = F_N^G \tag{S9-6}$$

$$N_b = F_N^C \tag{S9-7}$$

where N_{ub} and N_b represent the normal force in the non-bent segments and bent segments, respectively; F_N^G means the component of gravity; F_N^C is normal force arises from the compressive force due to the bending of the inner tube. The normal force generated by gravity is:

$$F_N^G = \sum m_i^m \mathbf{g} \cdot \mathbf{s}_i^N \tag{S9-8}$$

Here, $\mathbf{s}_i^N$ is the unit direction vector of the normal force for the ith segment. As elaborated in Supplementary Note 2, the angle of this segment relative to the $+x$ axis is α_i, and $\mathbf{s}_i^N$ can be calculated by

$$\mathbf{s}_i^N = (-\sin\alpha_i, \cos\alpha_i) \tag{S9-9}$$

The normal force exerted by the inner tube on the outer tube originates from the compression of the outer tube by the bent profile of the inner tube, and the formation of this bent profile depends on the bending moment experienced during bending. To simplify calculations, we approximate the normal force generated by the bending of the

inner tube as the force corresponding to the bending moment, denoted as F_c, and thus there is

$$F_N^C = F_c \tag{S9-10}$$

Therefore, the resultant force required for the inner tube to move within the outer tube is:

$$F_{m-total} = F_c + F_e^G + \sum F_i^f \tag{S9-11}$$

The tension rod exhibits stability and can operate as long as the axial tensile stress remains below its yield strength. Therefore, if the relative motion resistance between the tubes is less than the tube's yield force, pulling the tube is feasible, i.e.,

$$F_{m-total} < F_y \tag{S9-12}$$

where F_y represents the tube's yield force, which can be calculated using the following formula:

$$F_y = \sigma_y A \tag{S9-13}$$

Here, σ_y denotes the tube's yield strength, and A is its cross-sectional area.

When pushing the tube, since it is subject to compressive force, the instability of the compression rod must be considered. Specifically, once the applied compressive load exceeds the critical buckling load, the compression rod loses stability and is unable to transmit axial force. Furthermore, since the tube is located within the Magnetic Interaction Zone and undergoes bending deformation under magnetic forces, and given that the clearance between the inner and outer tubes is relatively small in this study, multiple contact points between the tubes are likely to form. For this situation, one simplified computational method is to calculate the critical buckling load of the tube segment between every two contact points (refer to Section 2.1 of this Note for the critical buckling load calculation of a straight rod), and then take the smallest of these calculated values as the critical buckling load F_{cr}, for that state, i.e.,

$$F_{cr} = \min\left(F_{cr}^i\right) \tag{S9-14}$$

where F_{cr}^{i} denotes the critical buckling load for the tube segment between the ith and $(i+1)^{th}$ contact points.

Due to the formation of multiple overlapping arcs of the inner tube within the outer tube, determining the number of contact points is challenging. Furthermore, once the tube buckles, additional contact points may emerge, leading to a subsequent equilibrium state. This situation is exceedingly complex, and the aforementioned calculation method can only provide a rough estimate. More precise calculations must be carried out using simulation software or determined experimentally. In simulation, a simplified approach similar to the calculation of the relative motion resistance $F_{m-total}$ for tube movement can be adopted, whereby the outer tube maintains a constant bending profile while the inner tube moves along that profile.

For experimental methods, different bending curvatures and numbers of bends can be designed based on the specific operation, taking into account other factors, and measurements can then be taken. For instance, as illustrated in Supplementary Figs. 65 and 89, we employed an experimental approach to assess the influence of various parameters—including radius of curvature, number of bends, clearance between tubes, material, motion mode, and lubrication method—on the relative motion resistance. This method not only provides design reference data but also enables the feasibility validation of the completed design.

Whether determined via experiments or simulations, the critical buckling load F_{cr}, can be obtained. In order to push the tube and achieve relative motion between the tubes, the following condition must be satisfied:

$$F_{m-total} < F_{cr} \tag{S9-15}$$

Considering the condition for achieving relative motion through pulling, the mechanical criterion for realizing relative tube motion within the Magnetic Interaction Zone—and thereby enabling real-time magnetic reprogramming—is given by:

$$\begin{cases} F_{cr} > F_{m-total} \\ F_y > F_{m-total} \end{cases} \tag{S9-16}$$

1.2 Design and Optimization Strategies

For operations within the Magnetic Interaction Zone, the tube's deformation will vary depending on the magnetic field strength. As the magnetic field increases, the bending of the tube intensifies, and the relative motion resistance between the tubes correspondingly increases. However, regardless of variations in the magnetic field or tube deformation, as long as the operational condition expressed in Equation (S9-16) is satisfied, relative motion between the tubes can be achieved.

If, based on operational requirements, the Magnetic Interaction Length needs to be further increased such that the relative motion resistance between the tubes $F_{m-total}$ exceeds the original critical buckling load F_{cr} and yield force F_y, the pushing and pulling operations will no longer be effective. In response, we propose the following solutions.

1.2.1 Reducing the Relative Motion Resistance $F_{m-total}$

Based on the analysis in Section 1.1 of this Note, under these conditions, the tube curvature cannot be altered (as the required curvature is necessary to fulfill specific operational demands). Therefore, reducing friction is the most effective strategy. The following discussion details methods for reducing friction between the tubes, along with the corresponding design and optimization strategies.

a) Utilization of low-friction materials

For soft materials, a variety of options are available. Under the constraint of meeting various performance requirements, selecting materials with a lower friction coefficient is preferable. For example, tubes fabricated from Ecoflex exhibit a slightly lower friction coefficient compared to those made from Polydimethylsiloxane (PDMS). However, while Ecoflex reduces friction, its lower elastic modulus relative to PDMS can affect other properties, such as deformation behavior and the critical buckling load. Therefore, a comprehensive evaluation is necessary. In this study, most tubes are fabricated from PDMS (Figs. 2-4, Supplementary Figs. 3-14), whereas some tubes are made from Ecoflex (Supplementary Fig. 15), and both the 2D and 3D structures are entirely fabricated using Ecoflex (Fig. 5, Supplementary Figs. 17-26).

b) Surface treatment of the tube

During or after fabrication, the tube surface can be chemically treated to reduce its friction coefficient. For instance, when fabricating tubes using silicone rubber, a surface tension diffuser (Slide STD-1, Kaupo, DE) can be added. This additive diffuses to the material's surface during the curing process, forming a thin, low-surface-energy film that effectively reduces friction. In this study, certain 2D sheets incorporated this surface tension diffuser to lower surface viscosity (Supplementary Figs. 17-24).

c) Enhancing lubrication conditions

Various lubricants can be introduced between the tubes to reduce friction. For example, incorporating talcum powder between the tubes can markedly lower friction; in addition, other lubrication methods, such as oil-based lubricants, may also be employed. In this study, talcum powder was added to all tubes to reduce the friction between them (Supplementary Figs. 3-14). We experimentally measured the relative motion resistance under different lubrication conditions. As illustrated in Supplementary Fig. 89C, the average relative motion resistance was 163.8 mN with 85% glycerol, 131 mN with water, and decreased to 83.6 mN when talcum powder was used. Additional analysis is provided in Supplementary Note 6.

d) Altering the tube's motion mode

When pushing or pulling the tubes, introducing a rotational component—rotating while simultaneously pushing or pulling—can modify the frictional interaction between the tubes. It changes the nature of friction between the tubes from static to kinetic, with the coefficient of kinetic friction generally being much lower than that of static friction. Additionally, rotation weakens the adhesive effects between the tubes by continuously reordering and refreshing the local contact surfaces, thereby reducing adhesive friction. The direct consequence of this operational modification is a significant reduction in the frictional force between the tubes. For instance, in this study, for scenarios involving relatively long tubes or tubes with pronounced curvature, a method combining rotation with pulling/pushing was adopted (Figs. 2b(iii), 2c, and 2e).To assess the impact of motion modes, we measured the relative motion resistance under different operational conditions. As illustrated in Supplementary Fig. 89B, when the tube's motion mode

changed from simple pulling to pulling combined with rotation, its relative motion resistance significantly decreased from 83.6 mN to 27.4 mN. Additional analysis is provided in Supplementary Note 6. Accordingly, for some applications in this study, the rotational motion control of the tube was integrated into the design of the control system, as illustrated in Supplementary Fig. 87.

1.2.2 Increasing the Critical Buckling Load F_{cr}

Although the classical formula for the critical buckling load of a straight rod cannot yield precise values, more complex calculations involving multiple contact points and simulations are based on similar principles. Thus, the parameters involved in the calculation of the critical buckling load for a straight rod at least reflect the positive or negative correlations of each parameter with the steady-state behavior. According to the analysis of the critical load in Section 2.1 of this Note (Equation (S9-28)), the critical buckling load can be increased by raising the tube's elastic modulus, increasing its area moment of inertia, or reducing its length. The following analysis examines these three aspects in detail.

a) Increasing the elastic modulus

The critical buckling load F_{cr} is directly proportional to the tube's elastic modulus. Increasing the elastic modulus can effectively raise the critical buckling load. However, the elastic modulus also directly influences the tube's deformation characteristics. Assuming that the tube bends under the influence of a magnetic field, and neglecting other factors, the energy density for a segment of arc length s is given by

$$u_{ela}(s) = \frac{1}{2}EI\kappa(s)^2 \tag{S9-17}$$

where $\kappa(s)$ is the curvature corresponding to the segment of arc length s. Let the angle between the tangent of this segment and a reference direction be $\theta(s)$, and then the curvature can be expressed as

$$\kappa(s) = \frac{\mathrm{d}\theta}{\mathrm{d}s} \tag{S9-18}$$

Therefore, the total bending energy of the tube is given by

$$U_{ela} = \frac{1}{2}EI\int\left(\frac{\mathrm{d}\theta}{\mathrm{d}s}\right)^2 \mathrm{d}s$$

(S9-19)

The magnetic potential energy associated with the tube is

$$U_{mag} = -mB\int cos\theta(s)\,\mathrm{d}s$$

(S9-20)

Neglecting other influencing factors for the time being, the total energy of the system is

$$U_{sys} = \frac{1}{2}EI\int\left(\frac{\mathrm{d}\theta}{\mathrm{d}s}\right)^2 \mathrm{d}s - mB\int\cos\theta(s)\,\mathrm{d}s$$

(S9-21)

In equilibrium, the system attains an extreme value, and the above expression can be reformulated as follows:

$$\frac{EI}{mB} = -\frac{\mathrm{d}^2\theta}{\sin\theta(s)\mathrm{d}s^2}$$

(S9-22)

When increasing the elastic modulus to enhance the critical buckling load, the tube's deformation becomes smaller than the originally prescribed deformation. To maintain the target deformation, and given that the right-hand side of Equation (S9-22) remains constant according to the previous analysis, it is necessary to increase either the magnetic moment m or the magnetic field strength B to preserve the tube's deformation. In other words, an increased elastic modulus E enhances the tube's resistance to bending, requiring the magnetic field to supply additional energy to induce the desired bending. Therefore, if the approach is to increase the magnetic moment, one must design the tube using materials with higher magnetization or incorporate permanent magnets (see Supplementary Note 10 for a detailed design method for magnetization profiles). Alternatively, if the strategy is to increase the magnetic field, the associated cost of enhancing the field must be considered. Within a certain range, increasing the magnetic field is the simplest and most direct method, as it is both straightforward and rapid, without necessitating changes to the existing design.

However, once the magnetic field strength reaches a certain threshold, the required field may exceed the capabilities of the current magnetic field generating apparatus, rendering this approach impractical.

Furthermore, we have experimentally verified the effects of both the elastic modulus and the magnetic field on tube bending (see Supplementary Note 3, Supplementary Figs. 58-61 for further details).

b) Increasing the area moment of inertia

Similar to the elastic modulus, the area moment of inertia is directly proportional to the critical buckling load F_{cr}. Increasing the area moment of inertia can effectively raise F_{cr}. This approach shares the same design concept as increasing the elastic modulus; by augmenting the area moment of inertia, the tube's resistance to bending is enhanced, which in turn necessitates an increase in the magnetic moment or magnetic field to compensate (see Equation (S9-22)). For a detailed discussion, please refer to the analysis on increasing the elastic modulus above. However, unlike the elastic modulus, enhancing the critical buckling load by increasing the area moment of inertia also poses dimensional design challenges. The area moment of inertia is directly related to the tube's dimensions and is given by

$$I = \frac{\pi}{4}(R^4 - r^4) \tag{S9-23}$$

where R is the outer radius of the tube and r is the inner radius of the tube. Thus, an increase in the area moment of inertia can only be achieved by either enlarging the outer diameter or reducing the inner diameter. Nevertheless, the outer and inner diameters are critical to the overall dimensions of the tube and the fit between multiple nested tubes. As a result, the possibility of adjusting the diameters is subject to various factors such as specific operational requirements and inter-tube compatibility. Unlike the elastic modulus, which can be varied more freely, modifications to the tube's dimensions must take all design parameters into careful consideration.

c) Reducing tube length

The square of the tube's length is inversely proportional to the critical buckling load. Consequently, shortening the tube yields a more pronounced increase in the

critical buckling load than merely increasing the elastic modulus or the area moment of inertia. However, because the tube's length is directly tied to specific operational requirements, there is no corresponding compensation strategy akin to those available for the elastic modulus and the area moment of inertia. Thus, reducing the tube length cannot serve as an effective post-design optimization measure; it can only be applied during the design phase, aiming to minimize the tube length while still meeting the operational criteria.

1.2.3 Increasing the Yield Force F_y

To increase the yield force, according to Equation (S9-13), one can either enhance the yield strength or augment the tube's cross-sectional area. For yield strength, selecting materials with higher yield strengths is a viable approach. With regard to increasing the cross-sectional area, this inherently involves adjustments to the tube's dimensions. The cross-sectional area is calculated by:

$$A = \pi(R^2 - r^2) \tag{S9-24}$$

where R denotes the outer radius of the tube and r the inner radius of the tube. According to this equation, increasing the outer radius or decreasing the inner radius will yield a larger cross-sectional area. However, as with the area moment of inertia, changes in dimensions affect the overall tube size and the compatibility among nested tubes. Therefore, practical operational requirements and all pertinent design parameters must be carefully considered to effectively achieve an increase in the cross-sectional area.

1.3 Case Study Analysis

To more clearly demonstrate the design solutions and optimization strategies discussed above, we present a case study that integrates the designs and applications involved in this research.

For the most common tubes used in this study, the outer tube has an outer diameter of 2.6 mm and an inner diameter of 1.7 mm, and is fabricated from PDMS with a base-

to-curing agent mass ratio of 20:1. The inner tube is replaced by a rod with a diameter of 1.4 mm, made from PDMS with a base-to-curing agent mass ratio of 10:1. Regarding the calculation of the yield Force, since both tubes are made of PDMS—and as an elastomer, PDMS does not exhibit a clearly defined "yield point" as metals do (it shows nonlinear elastic behavior at large strains and often lacks a conventional yield)—we adopt the approach commonly used in other studies. Specifically, we use the ultimate tensile strength rather than the traditional yield strength for the calculation. That is,

$$\sigma_y = \sigma_u \tag{S9-25}$$

where σ_y denotes the yield strength and σ_u denotes the ultimate tensile strength. Based on our measurements of the elastic modulus for materials with different base-to-curing agent mass ratios, the approximate ultimate tensile strength of PDMS is about 0.4 MPa for a 20:1 ratio and about 3.2 MPa for a 10:1 ratio. Consequently, the yield force can be calculated using Equations (S9-13) and (S9-25). Substituting the data into these formulas results in a yield force of 1220 mN for the outer tube and 4930 mN for the inner rod. Supplementary Fig. 65 displays the average relative motion resistance between the two tubes under various conditions and these values are generally below 60 mN, with the maximum reaching 244 mN when multiple bends occur. Since the yield force provided by the rod far exceeds this relative motion resistance, effective relative motion between the tubes can be achieved by pulling.

For the critical buckling load, it can be calculated by using Equation (S9-28). The critical buckling load depends on the tube's effective length under load, which decreases as the effective length increases. In Supplementary Fig. 65A, we measured the average friction force between the two tubes in a straight configuration (0.9 mN). Based on this value, we back-calculate the maximum operational length that can be achieved under this friction force. Substituting the data into Equation (S9-28) yields a maximum operational length of 179 mm for the tube and 162.5 mm for the rod. To ensure conservative estimation, a safety factor of 2 is applied. Under these conditions, the maximum operational length is reduced to 127 mm for the tube and 115 mm for the rod. This length is sufficient for the vast majority of operations. A similar calculation

method can be applied for bending operations. For designs requiring a longer operational length, specific design solutions and optimization strategies are presented in Section 4.1 of this Note.

Next, we will discuss each of the three improvement measures analyzed in Section 1.2 of this Note.

For the strategy of reducing the relative motion resistance $F_{m-total}$, some designs have employed both enhanced lubrication conditions and an altered tube motion approach. In demonstrations of relative motion for 1D tubes as well as 2D or 3D objects, we applied talcum powder to the surfaces of the components to reduce friction during relative motion. Additionally, in certain operations, we combined rotational movement with translation to facilitate the pushing or pulling of the tube. For example, in the "Contact-free object navigation" section—where two bends are used to overcome an obstacle (Fig. 2b(iii))—we implemented a method involving rotational advancement and rotational retraction, which significantly reduced friction and eased the operation. In contrast, in other applications where friction is relatively minor (e.g., "Reprogrammable cilia array" section), direct pushing and pulling are sufficient to complete the operation quickly, thereby eliminating the need for rotational motion.

In the measures for increasing the critical buckling load F_{cr}, some designs employ strategies such as increasing the elastic modulus and reducing the tube length. For instance, in the "Reprogrammable cilia array" section, the relative motion resistance $F_{m-total}$ is low. Consequently, using an inner rod fabricated from PDMS with a base-to-curing agent mass ratio of 10:1 (with an elastic modulus of 1 MPa) is sufficient to achieve the desired operation. In contrast, in the "Contact-free object navigation" section, multiple bends in 3D space are used to overcome obstacles (see Fig. 2h, Supplementary Figs. 80-83 and Supplementary video 17) and the increased relative motion resistance $F_{m-total}$ necessitates a higher critical buckling load F_{cr} to ensure successful operation. In this case, a composite material consisting of PDMS and magnetic powder, with an effective elastic modulus of 12.3 MPa, was employed. The more than tenfold increase in the elastic modulus leads to a corresponding increase in

F_{cr} by a similar factor. In designing the specific application, the effective tube length was minimized as much as possible while still meeting the operational requirements.

Regarding measures to increase the yield force F_y, some schemes substitute the original material with one that has a higher yield strength. In the "Contact-free object navigation" section, for example, to achieve multiple bends for overcoming obstacles in 3D space, we replaced pure PDMS with a composite of PDMS and magnetic particle to fabricate the inner rod (Supplementary Fig. 80). Our experiments indicate that the ultimate tensile strength of the PDMS/magnetic particle composite (with a mass ratio of PDMS to magnetic particle of 1:3) is approximately 4.3 MPa, compared to about 3.2 MPa for PDMS with a base-to-curing agent mass ratio of 10:1. By substituting the ultimate tensile strength for the yield strength in the calculations (i.e., $\sigma_y = \sigma_u$), and incorporating the data into Equation (S9-13), we computed that the yield force of the composite is 7.1 N, while that of pure PDMS is 5.28 N—a 35% increase in the yield force.

2. Feeding Zone

2.1 Mechanics Analysis

In the Feeding Zone, the tube is no longer influenced by the magnetic field. However, it is subjected to tensile forces, compressive forces, and torques during movement, as well as forces or torques transmitted from the tube in the Magnetic Interaction Zone. In this region, the tube either has a straight configuration or exhibits bending. Two types of motion or deformation occur in the Feeding Zone: relative motion between the tubes and bending under load.

For the relative motion between tubes, when the two nested tubes are subjected to tensile or compressive forces, it is essential to ensure that relative movement can be achieved. The tube in the Feeding Zone serves as a connector between the Magnetic Interaction Zone and the Actuation Zone, transmitting forces from the Actuation Zone to the tube in the Magnetic Interaction Zone. The force acting on the tube at the interface between the Feeding Zone and the Magnetic Interaction Zone is identical to the force it transmits to the Magnetic Interaction Zone (i.e., the force at point A at the junction of

the two zones as shown in Supplementary Fig. 75A, neglecting acceleration effects), namely,

$$F_{f-total} = F_{m-total} \tag{S9-26}$$

Therefore, the force acting on the tube in the Feeding Zone can be determined using the same calculations as for the tube in the Magnetic Interaction Zone described in Section 1.1 of this Note. Similarly, due to the stability of the tension rod, to achieve relative motion in the Feeding Zone through pulling, the following condition must be satisfied:

$$F_{f-total} < F_y \tag{S9-27}$$

For obtaining relative motion between the tubes through pushing, a similar analysis as in Section 1.1 of this Note is performed, by simplifying the situation to one in which one tube is fixed while the other moves. Whether the outer tube is under compression or the inner tube is under compression, the scenario can be equivalently represented by the situation shown in Supplementary Fig. 75B, where both ends of the rod are subjected to compressive forces, resulting in deflection. Based on the working conditions of the two tubes, we assume fixed boundary conditions for the compression rod at both ends. According to classical Euler buckling theory, the critical buckling load is given by:

$$F_{cr} = \frac{4\pi^2 EI}{L_{fl}^2} \tag{S9-28}$$

where E is the elastic modulus of the soft tube, I is the area moment of inertia of the soft tube, and L_{fl} is the feeding length of the soft tube.

When the axial load applied at both ends of the soft tube exceeds the critical buckling load, the soft tube will buckle. According to the Euler-Bernoulli beam theory, we have:

$$EI\frac{\mathrm{d}^4 w}{\mathrm{d}x^4} = F\frac{\mathrm{d}^2 w}{\mathrm{d}x^2} \tag{S9-29}$$

where w represents the lateral deflection and F denotes the applied axial load. Therefore, the buckling equation of the tube is given by

$$\frac{\mathrm{d}^4 w}{\mathrm{d}x^4} - \frac{F}{EI}\frac{\mathrm{d}^2 w}{\mathrm{d}x^2} = 0$$

(S9-30)

By solving the characteristic equation of this differential equation, the general solution is obtained as

$$w(x) = A + Bx + C\cos(kx) + D\sin(kx) \quad \text{(S9-31)}$$

Since the tube is assumed to be fixed at both ends, both the displacements and rotations at its ends are zero. The specific boundary conditions are:

$$w(0) = 0, \ w(L_{fl}) = 0 \quad \text{(S9-32)}$$

$$w'(0) = 0, \ w'(L_{fl}) = 0 \quad \text{(S9-33)}$$

Based on these boundary conditions, the deflection curve of the tube is given by

$$w(x) = A\left[1 - \cos\left(\frac{2\pi x}{L_{fl}}\right)\right] \quad \text{(S9-34)}$$

Since the maximum deflection occurs at the midpoint of the tube, the maximum deflection is given by

$$w_{max} = w\left(\frac{L_{fl}}{2}\right) = 2A \quad \text{(S9-35)}$$

Here, the constant A is determined by the initial imperfections, the degree of overload, and the material properties. For example, for roughly processed rod elements, a typical estimate is $A = 0.01L_{fl}$. In this study, as a conservative estimate, we assume zero deflection, meaning that we treat the critical buckling load as the maximum compressive force the tube can withstand while remaining stable—that is, the maximum pushing force it can provide. Therefore, as long as the relative motion resistance between the tubes is less than the critical buckling load, the tube can be effectively pushed, namely,

$$F_{f-total} < F_{cr} \tag{S9-36}$$

In summary, the mechanical criterion for achieving relative motion between the tubes in the Feeding Zone is

$$\begin{cases} F_{cr} > F_{f-total} \\ F_y > F_{f-total} \end{cases} \tag{S9-37}$$

In the Feeding Zone, the bending of the tube is induced by a force or torque transmitted from the tube in the Magnetic Interaction Zone. To calculate the deformation under this loading, we can adopt a simplified cantilever beam model (Supplementary Fig. 75C) to evaluate the bending deformation experienced by the two nested tubes under the applied force and moment.

As shown in Supplementary Fig. 75C, the cantilever beam comprises two tubes, with both a force F and a torque M applied simultaneously at the free end. Owing to the superposition principle inherent in linear elasticity, the deformations induced by the force and the torque can be analyzed separately. First, the deflection due to the force is calculated, followed by the deflection resulting from the torque, and finally, the two deflections are superimposed.

The displacement of the tube under the applied force can be decomposed into x- and y-components. Since axial deformation along the x direction is negligible for small deformations, we only consider the deformation along the y direction. Therefore, the y-component of the force $\mathbf{F}$ is given by:

$$F_y = \mathbf{F} \cdot \boldsymbol{e_y} \tag{S9-38}$$

where $\boldsymbol{e_y}$ is the unit vector in the y-direction. The torque distribution along the beam under the force is given by

$$M_F(x) = F_y(L_f - x) \tag{S9-39}$$

where L_f represents the length of the tube in the Feeding Zone. The fundamental bending equation for the cantilever beam is:

$$E_e I_e \frac{\mathrm{d}^2 y(x)}{dx^2} = M(x)$$

(S9-40)

where E_e is the equivalent elastic modulus of the two tubes and I_e is the equivalent area moment of inertia. If the tubes possess identical elastic moduli, then the equivalent elastic modulus is equal to the original modulus, and the equivalent area moment of inertia can be determined by the following equation:

$$I_e = \sum I_i \tag{S9-41}$$

where I_i represents the area moment of inertia of the ith tube. If the tubes have different elastic moduli, then we can calculate the equivalent area moment of inertia by combining the individual area moments of inertia as follows:

$$E_e I_e = \sum E_i I_i$$

(S9-42)

where E_i is the elastic modulus of the ith tube. By substituting the moment distribution derived from the force into the cantilever beam bending equation and applying the beam's boundary conditions, integration yields the displacement at the free end as follows:

$$\delta_F = \frac{L_f^3}{3E_e I_e} \mathbf{F} \cdot \boldsymbol{e}_y$$

(S9-43)

Similarly, the displacement at the free end produced by the torque is given by

$$\delta_M = \frac{L_f^2}{2E_e I_e} M$$

(S9-44)

Consequently, the total displacement of the tube end along the *y*-direction is

$$\delta_{total} = \delta_F + \delta_M \tag{S9-45}$$

$$\delta_{total} = \frac{L_f^3}{3E_e I_e} \mathbf{F} \cdot \boldsymbol{e}_y + \frac{L_f^2}{2E_e I_e} M$$

(S9-46)

After deformation under load, the tube's end undergoes a rotation corresponding to an angle of

$$\theta(x) = \frac{dy}{dx} \tag{S9-47}$$

By integrating the fundamental bending equation for a cantilever beam and applying the boundary conditions, the rotation at the free end of the tube due to the applied force is given by:

$$\theta_F = \theta(L) = \frac{L_f^2}{2E_e I_e} \mathbf{F} \cdot \boldsymbol{e_y}$$

(S9-48)

Similarly, the rotation produced at the free end of the tube by the applied torque is given by

$$\theta_M = \frac{L_f}{E_e I_e} M$$

(S9-49)

Therefore, the total rotation at the free end of the tube resulting from both the applied force and torque is

$$\theta_{total} = \theta_F + \theta_M \tag{S9-50}$$

$$\theta_{total} = \frac{L_f^2}{2E_e I_e} \mathbf{F} \cdot \boldsymbol{e_y} + \frac{L_f}{E_e I_e} M$$

(S9-51)

2.2 Design and Optimization Strategies

In the Feeding Zone, the tube serves as a connection between the Magnetic Interaction Zone and the Actuation Zone. To enable effective control of the tube within the Magnetic Interaction Zone, it is essential to ensure that relative motion can occur between the tubes in the Feeding Zone. According to the analysis above, two conditions must be met, as expressed in Equation (S9-37). If the relative motion resistance $F_{f-total}$, becomes too high such that Equation (S9-37) is no longer satisfied, and then

manipulation of the tube in the Feeding Zone cannot be accomplished. Consequently, the following solutions or optimization strategies are proposed.

To meet the conditions outlined in the equation, three main considerations can be addressed: reducing the relative motion resistance $F_{f-total}$, increasing the critical buckling load F_{cr}, and increasing the yield force F_y. These improvement strategies are essentially the same as those discussed in Section 1.2 of this Note, so they will not be repeated here. The primary distinction lies in the approach of shortening the tube to increase F_{cr}. Shortening the tube has a more pronounced effect on enhancing the critical buckling load than increasing either the elastic modulus or the area moment of inertia. However, because the tube's length is directly tied to specific operational requirements, there is no corresponding compensation strategy for length as there is for elastic modulus and area moment of inertia. Consequently, in Section 1.2 of this Note, reducing the tube length was not considered an effective post-design optimization measure but rather a design guideline to be addressed during the initial design phase. In contrast, within the Feeding Zone the tube length is not directly related to the specific operational requirements. Under appropriate conditions, reducing the tube length in the Feeding Zone can effectively increase F_{cr}. In addition to the aforementioned methods for increasing F_{cr}, we also proposed a segmented design approach (stiffness segmentation), which will be discussed in detail in Section 4.1 of this Note.

Since the tube in the Feeding Zone is subjected to forces and torques transmitted from the tube in the Magnetic Interaction Zone, it will experience a displacement in the direction perpendicular to its axis. The magnitude of this displacement and the corresponding rotation can be calculated using Equations (S9-46) and (S9-51). This displacement may cumulatively affect the operation of the tube in the Magnetic Interaction Zone. If the cumulative effect is negligible or does not interfere with the operation, the displacement can be ignored. However, if the displacement significantly impacts the performance of the tube in the Magnetic Interaction Zone, measures must be taken to reduce it. Based on the mechanical analysis above, optimization can be approached from three aspects: increasing the equivalent elastic modulus E_e, increasing the equivalent area moment of inertia I_e, and reducing the applied force F

and torque M. The strategies for increasing the elastic modulus and the area moment of inertia have already been discussed in Section 1.2 of this Note, so those analyses need not be repeated here. In contrast, the force and torque acting on the tube are determined by the operation of the tube in the Magnetic Interaction Zone and cannot be arbitrarily reduced. Therefore, enhancing the elastic modulus and the area moment of inertia is an effective method for mitigating displacement and rotational errors. Additionally, the segmented design method proposed in Section 4.1 of this Note can also effectively reduce or even eliminate the observed displacement and rotational errors.

We have discussed methods to reduce or even eliminate the displacement at the free end of the tube in the Feeding Zone, thereby mitigating its impact on the operation of the tube in the Magnetic Interaction Zone. An alternative approach is to incorporate this displacement into the design of the operation for the tube in the Magnetic Interaction Zone. In other words, when designing the operation for the tube in the Magnetic Interaction Zone, this displacement is fully considered, and a compensation strategy is employed to account for it. This compensation method is relatively effective and practical, although it does increase the complexity of the design process.

2.3 Case Study Analysis

Below, we combine a specific application from our study to analyze and discuss the tube in the Feeding Zone, focusing on the feasibility of the operation, the effective operating distance, and the displacement error at the tube's free end. For example, in the "Coordinated multi-instrument operation" section, the outer tube is fabricated from PDMS with a base-to-curing agent mass ratio of 20:1, having an elastic modulus of 0.33 MPa (Supplementary Fig. 62), an outer diameter of 2.6 mm, and an inner diameter of 1.7 mm. For the inner tube, a rod is used as a substitute; it is made from PDMS with a base-to-curing agent mass ratio of 10:1, with an elastic modulus of 1 MPa (Supplementary Fig. 62) and a diameter of 1.4 mm. Suppose that, in the Magnetic Interaction Zone, the tube is required to achieve a radius of curvature of 15 mm. Under these conditions, our experiments measured a relative motion resistance between the

tubes of 15.2 mN (Supplementary Fig. 65). By setting this 15.2 mN as the critical buckling load in Equation (S9-28), the calculated maximum operating lengths are 39.7 mm for the outer tube and 22.1 mm for the inner rod. Therefore, the outer tube and the inner rod can be arbitrarily pushed over ranges of 39.7 mm and 22.1 mm, respectively. For the yield force calculation, as discussed in Section 1.3 of this Note, we substitute the ultimate tensile strength for the conventional yield strength (see Equation (S9-25)). Our experimental results indicate that PDMS with a base-to-curing agent mass ratio of 20:1 has an ultimate tensile strength of approximately 0.4 MPa, whereas PDMS with a ratio of 10:1 exhibits an ultimate tensile strength of about 3.2 MPa. Using Equation (S9-13), the yield force for the outer tube is calculated to be 1.22 N, and that for the inner rod is 4.93 N. Since both the outer tube and the inner rod possess yield forces that far exceed the relative motion resistance of 15.2 mN, pulling operations can be readily performed.

Regarding the operational errors induced by the tube in the Feeding Zone, we still analyze the tube used in the "Coordinated multi-instrument operation" section as an example. For the radial displacement error, only the error due to the operating force is considered. To calculate the maximum error, a conservative approach is adopted: the maximum achievable operating length is used as the tube length, and the maximum relative motion resistance is taken as the operating force. Generally, the tube transmits the operating force axially, so the angle between the force and the tube's axis is minimal; for conservative calculation, this angle is set to 3°. Substituting these parameters along with the elastic modulus and geometric properties into Equations (S9-41), (S9-42) and (S9-46), we calculate a lateral deflection at the tube's free end of 3.59 mm and an angular displacement of 14°. In addition, using the axial deformation formula $L = {FL}/{EA}$ yields an axial deformation of 0.132 mm, which is much smaller than the lateral deflection. This lateral error and angular displacement are directly superimposed on the tube in the Magnetic Interaction Zone.

To address this issue, two approaches can be adopted. The first approach is to incorporate this error into the design and operation of the tube in the Magnetic

Interaction Zone by employing a compensation strategy to mitigate or eliminate the error originating from the Feeding Zone. The second approach is to reduce these errors using the methods described in Section 2.2 of this Note. In this study, we adopted the second approach. In conjunction with the solution presented in Section 4.1 of this Note, we replaced the rod in the Feeding Zone with a 1 mm diameter copper rod. Pure copper has an elastic modulus of approximately 115 GPa and a yield strength of around 150 MPa. After substituting these data and recalculating, the lateral deflection at the tube's free end is determined to be 5.05×10^{-4}mm, the angular displacement is 0.002°, and the axial deformation is 3.71×10^{-6}mm. These errors are negligible. Moreover, substituting these values into Equations (S9-28) and (S9-13) yields a critical buckling load of 2228 N and a yield force of 117.8 N, both of which far exceed the relative motion resistance of the tube (15.2 mN). Therefore, pushing and pulling can effectively be used to achieve relative motion between the tubes.

3. Actuation Zone

3.1 Mechanics Analysis

In the Actuation Zone, the two tubes are gripped separately by the grippers to perform the operation (as shown in Supplementary Fig. 76A). Only the inner tube undergoes tensile and compressive deformations. When pulling the tube, similar to the analysis in Section 1.1 of this Note, it is sufficient that the tube's yield force exceeds the resistance to relative motion between the tubes. In the Actuation Zone, there is no nesting of multiple tubes; therefore, this relative motion resistance is equal to the relative motion resistance in the Feeding Zone $F_{f-total}$, and must satisfy

$$F_{f-total} < F_y \tag{S9-52}$$

For pushing the tube, as analyzed in Section 2.1 of this Note, the issue of compression rod stability arises. Consequently, it is similarly required that the critical buckling load exceeds the relative motion resistance, i.e.,

$$F_{f-total} < F_{cr} \tag{S9-53}$$

In summary, similar to the Feeding Zone, the mechanical criteria for achieving tube manipulation in the Actuation Zone are:

$$\begin{cases} F_{cr} > F_{f-total} \\ F_y > F_{f-total} \end{cases} \tag{S9-54}$$

3.2 Design and Optimization Strategies

In the Actuation Zone, only the tensile and compressive deformations of a single tube are involved. If the tube's operation does not satisfy Equation (S9-54), effective manipulation cannot be achieved. The specific design solutions and improvement strategies are identical to those in the Feeding Zone (Section 3 of this Note) and are therefore not repeated here. In addition, the segmented design method proposed in Section 4.1 of this Note can be applied to the design of the tube in the Actuation Zone. The detailed design is as follows.

We adopt a segmented design method in which the inner tube is no longer designed as a single, homogeneous element using identical materials or parameters. Instead, a material with a higher elastic modulus and higher yield strength is used to fabricate the tube in the Actuation Zone, while the tubes in the Feeding Zone and Magnetic Interaction Zone continue to use the original material. These two types of tubes are then connected, as shown in Supplementary Fig. 76B. The use of a high-modulus material transforms the tube from a soft tube into a semi-rigid or rigid tube. As a result, in the Magnetic Interaction Zone where deformation is required, the original soft tube is still employed to ensure the desired deformation and functionality; whereas in the Actuation Zone, the semi-rigid or rigid tube substantially increases the yield force F_y and the critical buckling load F_{cr}, thereby ensuring that Equation (S9-54) is satisfied and effective tube manipulation is achieved. For the quantitative improvements and corresponding operational performance, please refer to the case study in Section 3.3 of this Note.

3.3 Case Study Analysis

In this study, this method discussed in Section 3.2 of this Note was used to optimize the tube in the Actuation Zone (with the inner tube replaced by a rod) in both

“Contact-free object navigation” and “Coordinated multi-instrument operation.” Two different materials were employed for the improvement, and both yielded excellent results. Consequently, this design approach was adopted in both “Contact-free object navigation” and Coordinated multi-instrument operation.”

The first material is a composite of PDMS and magnetic particles. In this composite, PDMS is prepared with a base-to-curing agent mass ratio of 10:1, and the mass ratio of magnetic particles to PDMS is 3:1, resulting in an elastic modulus of 12.3 MPa (Supplementary Fig. 62). In the Actuation Zone, the actuator spacing for the tube is set to 10 mm, and the rod substituting the tube has a diameter of 1.4 mm. By substituting these parameters into the critical buckling load equation (Equation (S9-28)), the critical buckling load is calculated to be 920 mN. In contrast, using pure PDMS with a 10:1 mass ratio (elastic modulus of 1 MPa) yields a critical buckling load of only 75 mN. Thus, the segmented design dramatically increases the critical buckling load compared to the original design. For the yield force calculation, as discussed in Section 1.3 of this Note, the “yield point” for elastomers is less well-defined than for metals due to their nonlinear elastic behavior under large strains; therefore, we use the ultimate tensile strength in our calculations. Experimentally, the ultimate tensile strength of the first material (the PDMS and magnetic particle composite) was approximately 4.3 MPa, compared to about 3.2 MPa for PDMS with a 10:1 mass ratio. Substituting these values into Equation (S9-13) results in a yield force of 7.1 N for the composite, whereas pure PDMS provides a yield force of 5.28 N. Furthermore, experimental measurements of the relative motion resistance between the tubes under various curvature radii (Supplementary Fig. 65) show that operating a tube in the Magnetic Interaction Zone with a radius of curvature of 5 mm requires a force of 43 mN, while a radius of curvature of 2 mm demands 118 mN. Therefore, the segmented design method enables both pushing and pulling operations for tubes with a curvature radius of 2 mm, whereas the original design would allow only pulling.

The second material is pure copper. We replaced the previous soft rod with a copper rod having a diameter of 1 mm. Pure copper exhibits an elastic modulus of approximately 115 GPa and a yield strength of around 150 MPa. Substituting these

values into Equations (S9-28) and (S9-13) yields a critical buckling load of 2228 N and a yield force of 117.8 N, respectively—values that far exceed the necessary requirements. Therefore, using a copper rod instead of a PDMS rod will enable smooth execution of the relative motion operation between the tubes.

4. Other Critical Considerations

In the preceding three sections, we analyzed and discussed the three zones—the Magnetic Interaction Zone, the Feeding Zone, and the Actuation Zone—and provided comprehensive design solutions and improvement strategies. However, during practical applications, several specific operational requirements arise. Although these requirements could be addressed within the scope of the aforementioned discussions and solutions, a more detailed analysis is necessary to effectively tackle these particular issues. Accordingly, we will separately analyze and discuss the **long-range operation**, the **multi-bend deformation operation**, the **high-curvature deformation operation** and the **multi-nested-tube operation**, and propose corresponding solutions.

4.1 Long-Range Operation

We divided the operational region of the tube into three zones: the Magnetic Interaction Zone, the Feeding Zone, and the Actuation Zone. Only the Magnetic Interaction Zone and the Feeding Zone contribute to the tube's effective operational length. Therefore, the Effective Operating Distance (D_{eo}) of the tube is defined as the sum of the tube lengths within these two zones, i.e.,

$$D_{eo} = L_{mi} + L_f \tag{S9-55}$$

where L_{mi} represents the Magnetic Interaction Length (in the Magnetic Interaction Zone) and L_f is the Feeding Length (in the Feeding Zone). In all the demonstration cases and the four applications presented in this study—whether involving 1D tube or 2D/3D objects derived from the 1D tube—the Effective Operating Distance remains within 100 mm. With increasing tube length, additional design considerations emerge.

Herein, we propose design strategies tailored for long-distance operations (100 mm or longer).

Based on the analysis in the previous sections of this Note, to achieve the relative motion of the tube, the relative motion resistance $F_{m-total}$, the critical buckling load F_{cr}, and the yield force F_y must satisfy Equation (S9-16). However, as the operating length increases, the relative motion resistance $F_{m-total}$ increases while the critical buckling load F_{cr} decreases, making it increasingly challenging or even impossible to meet this condition. To address this issue, we propose a **segmented design method** that enables long-distance operations. Moreover, this segmented design method can also effectively resolve the challenges encountered in short-distance operations.

4.1.1 Segmented Design Method

The segmented design method involves dividing the tube into several smaller segments, each with distinct physical or geometric parameters, and then assembling these segments to form a complete tube. As a result, the tube exhibits different physical properties in each segment. In this study, based on the analysis in the previous sections, the two key parameters affecting the relative motion resistance $F_{m-total}$, the critical buckling load F_{cr}, and the yield force F_y are the bending stiffness and yield strength. Therefore, we assign different stiffness and yield strengths to the tube in the Magnetic Interaction Zone, the Feeding Zone, and the Actuation Zone. The segmented stiffness for the different regions is as follows:

$$E_sI_s = \begin{cases} E_1I_1, & \text{Magnetic interaction zone} \\ E_2I_2, & \text{Feeding zone} \\ E_3I_3, & \text{Actuation zone} \end{cases} \tag{S9-56}$$

where E_sI_s represents the segmented stiffness, and E_iI_i (i =1, 2, 3) denotes the stiffness of each region. Similarly, the segmented yield strengths for the different regions are given as follows

$$\sigma_{ys} = \begin{cases} \sigma_{y1}, & \text{Magnetic interaction zone} \\ \sigma_{y2}, & \text{Feeding zone} \\ \sigma_{y3}, & \text{Actuation zone} \end{cases} \tag{S9-57}$$

Here, σ_{ys} represents the segmented yield strength, and σ_{yi} (i = 1, 2, 3) denotes the yield strength in each region. Since the relative motion resistance between the tubes increases from the Magnetic Interaction Zone to the Feeding Zone and the Actuation Zone, the segmented stiffness and segmented yield strength can be set as follows:

$$E_1 I_1 < E_2 I_2 < E_3 I_3 \tag{S9-58}$$

$$\sigma_{y1} < \sigma_{y2} < \sigma_{y3} \tag{S9-59}$$

Based on the above equations, three materials with increasing stiffness and yield strength can be selected to manufacture the tube segments in the Magnetic Interaction Zone, the Feeding Zone, and the Actuation Zone, respectively. The stiffness EI is the product of the elastic modulus E and the moment of inertia I of the cross-section. Since altering the moment of inertia I would require changes in dimensions that not only increase manufacturing difficulty but also affect the fit between the tubes, modifying E is the simplest and most direct method to change the tube's stiffness. Therefore, the segmented design of stiffness is equivalent to a segmented design of the elastic modulus, namely:

$$E_s = \begin{cases} E_1, & \text{Magnetic interaction zone} \\ E_2, & \text{Feeding zone} \\ E_3, & \text{Actuation zone} \end{cases} \tag{S9-60}$$

Because the relative motion resistance changes with the tube's position, being zero at the end of the Magnetic Interaction Zone and reaching its maximum at the gripper in the Actuation Zone, we can improve the design by dividing the tube into more segments instead of into only three regions. The detailed segmentation is as follows:

$$E(x) = \begin{cases} E_1, & x \in [0, L_1) \\ E_2, & x \in [L_1, L_2) \\ \vdots & \vdots \\ E_n, & x \in [L_{n-1}, L_n] \end{cases} \tag{S9-61}$$

$$E_1 < E_2 < \cdots < E_n \tag{S9-62}$$

$$0 < L_1 < L_2 < \cdots < L_n = L \tag{S9-63}$$

where x denotes the axial distance along the tube, with the starting point at the end of the tube in the Magnetic Interaction Zone, and L represents the total length of the tube. Naturally, if conditions permit, we can adopt a design approach similar to that of "constant strength beams" to further increase the number of segments and achieve a smooth and gradual transition. Its elastic modulus can be expressed as:

$$E(x) = E_0 + kx \tag{S9-64}$$

$$E_0 > 0, \quad k > 0 \tag{S9-65}$$

Certainly, the tube's yield strength can be designed in the same manner.

By employing the segmented design method described above, the tube can be configured with varying critical buckling loads F_{cr} and yield forces F_y along its length, with these values increasing toward the Actuation Zone. Since the relative motion resistance $F_{m-total}$ between the tubes also increases as the location approaches the Actuation Zone, this design approach ensures that in each segment the critical buckling load F_{cr} and yield force F_y always exceed the relative motion resistance $F_{m-total}$, thereby enabling long-range operation.

4.1.2 Design and Optimization Strategies

According to the segmented design concept described above, we divide the tube into three segments (see Supplementary Fig. 77). For the outer tube, the corresponding elastic modulus and yield strength are E_i and σ_{yi} (i = 1, 2, 3), respectively, with their values increasing sequentially. For the inner tube, the corresponding elastic modulus and yield strength are E'_i and σ'_{yi} (i = 1, 2, 3), which also increase respectively. In the same region, the elastic modulus and yield strength of the inner and outer tubes must be designed according to the dimensions of the tube to ensure that they exhibit the same critical buckling load and yield force. Under appropriate conditions, for tubes located within the same zone, a straightforward approach can be adopted by setting them to have the same elastic modulus and yield strength, that is, by fabricating them using the same material.

In fact, there is no standard for dividing the tube into segments as long as the required performance criteria are met, although the gradual increase method described above (Equation (S9-64)) is optimal. For example, in the "Contact-free object navigation demonstration" section and "Coordinated multi-instrument operation" section, we adopted a two-segment design where the tube in the Actuation Zone was adjusted in terms of its elastic modulus and yield strength, while the tubes in the other zones continued to use the original material. We used two types of materials for the tube in the Actuation Zone: the first was a composite of PDMS and magnetic powder, and the second was pure copper. With the first material, the tube's critical buckling load increased from 75 mN to 920 mN, and the yield force rose from 5.28 N to 7.1 N. With the second material, the tube's critical buckling load increased from 75 mN to 2228 N, and the yield force increased from 5.28 N to 117.8 N. For a detailed analysis, please refer to Section 3.3 of this Note.

For long-range operation, according to the definition given in Equation (S9-55), the Effective Operating Distance consists of the Magnetic Interaction Length L_{mi} and the Feeding Length L_f. Since the Effective Operating Distance is determined by these two parameters, long-range operation can be categorized into three distinct modes based on their relative contributions: **long-range operation with a large Magnetic Interaction Length**, **long-range operation with a large Feeding Length**, and **long-range operation with a large Magnetic Interaction Length and large Feeding Length** (see Supplementary Fig. 78). The following sections provide a detailed explanation of these three modes.

(1) Long-range operation with a large Magnetic Interaction Length

When significant deformation or complex operations are required, it is necessary to increase the Magnetic Interaction Length. In such cases, the operation features an enlarged Magnetic Interaction Zone while the Feeding Zone remains relatively small, as shown in Supplementary Fig. 78A. Due to the relatively small size of the Feeding Zone, when it is negligible compared to the Magnetic Interaction Zone, the tube in the Feeding Zone and its corresponding Feeding Length can be temporarily disregarded. In the Actuation Zone, tubes are fabricated using materials with high stiffness and yield

strength (see Section 3 of this Note for details). Meanwhile, the tube within the enlarged Magnetic Interaction Zone must be modified to accommodate long-range operation.

The first method involves segmenting the tube within the Magnetic Interaction Zone. Since the tube in this zone has a relatively long length, the relative motion resistance $F_{m-total}$ increases with length. To satisfy the conditions for relative motion (Equation (S9-16)), it is necessary to both reduce the relative motion resistance $F_{m-total}$ and enhance the critical buckling load F_{cr} and yield force F_y. We can apply the approach outlined in Section 1.2 of this Note to reduce $F_{m-total}$. However, relying solely on this strategy may prove insufficient when the operating length increases substantially, as the mechanical conditions for relative motion might no longer be met. In contrast, increasing F_{cr} and F_y is more effective. Therefore, based on the method proposed in Section 4.1.1 of this Note, we can achieve improvements by enhancing the tube's stiffness and yield strength.

For example, in the demonstration "Long-range Operation with a Large Magnetic Interaction Length" (Supplementary Fig. 84, Supplementary Video 18), we employed two outer tubes and an inner rod. The outermost tube is positioned in the Magnetic Interaction Zone on the Actuation Zone side, while the secondary outer tube extends farther. The outermost tube was fabricated using PDMS with a base-to-curing agent mass ratio of 10:1, yielding an elastic modulus of 1 MPa; in contrast, the secondary outer tube was made using PDMS with a mass ratio of 15:1, resulting in an elastic modulus of 0.55 MPa. Since the outermost tube is nearer to the Actuation Zone, its relative motion resistance is higher. This design imparts a greater critical buckling load to the outermost tube, enabling it to overcome the relative motion resistance between the tubes. Additionally, this design also considers the need to achieve multiple bending deformations under a magnetic field. We present this case just as an example for reference.

The second method involves designing the tube in the Magnetic Interaction Zone as a whole using materials with high stiffness and high yield strength. This approach is relatively straightforward, as it directly employs the same material for the tube in the Magnetic Interaction Zone. For example, similarly, in the demonstration "Long-range

Operation with a Large Magnetic Interaction Length" (Supplementary Fig. 84, Supplementary Video 18), the innermost rod was fabricated using a composite of PDMS and magnetic particles. The PDMS was prepared with a base-to-curing agent mass ratio of 10:1, and the magnetic particle was mixed with PDMS at a mass ratio of 3:1, resulting in a composite elastic modulus of 12.3 MPa. In comparison, PDMS with a base-to-curing agent mass ratio of 10:1 has an elastic modulus of 1 MPa. This design increases the tube's critical buckling load by a factor of 11.3.

We fabricated the outermost outer tube (Tube A) and secondary outer tube (Tube B) using the first method, and the innermost rod using the second method. These components were then assembled and operated within a large magnetic field region. Tube B has a length of 315 mm, while the rod measures 438 mm. Accounting for 10 mm allocated to clamping both Tube B and the rod in the Actuation Zone, we achieved a Magnetic Interaction Length of 305 mm (see Supplementary Fig. 84, Supplementary Video 18). Including the tube length in the Feeding Zone, the total Effective Operating Distance can be expressed as

$$D_{eo} = L_f + 305mm \tag{S9-66}$$

The Magnetic Interaction Length of 305 mm demonstrated here is intended solely for experimental demonstration purposes. Naturally, with increased tube stiffness and yield strength, this length could be further extended. However, an increase in stiffness requires the tube to have a higher magnetization intensity or to be exposed to a stronger magnetic field to achieve the desired deformation. For a detailed analysis of the effect of the stiffness on the deformation, please refer to Section 1.2.2 of this Note.

(2) Long-range operation with a large Feeding Length

If the Magnetic Interaction Zone is located far from the Actuation Zone, it is necessary to increase the size of the Feeding Zone—that is, to extend the Feeding Length—to achieve long-range operation. In this case, the Feeding Zone is significantly larger than the Magnetic Interaction Zone (see Supplementary Fig. 78B). To ensure that the relative motion of tubes can be maintained within this substantially enlarged Feeding Zone, it is essential, as in the previous analysis, to reduce the relative motion

resistance $F_{f-total}$ and to increase the critical buckling load F_{cr} and the yield force F_y. The reduction in $F_{f-total}$ can be achieved using the method described in Section 1.2 of this Note, while the enhancement of F_{cr} and F_y can be accomplished by directly employing materials with high stiffness and high yield strength.

Since the tube in the Feeding Zone is not within the magnetic field and does not undergo deformation, it solely functions to control the tube in the Magnetic Interaction Zone. Therefore, the tube in the Feeding Zone can be fabricated using materials with arbitrarily high stiffness and yield strength. In theory, as long as the material's stiffness and yield strength are sufficiently high, the critical buckling load F_{cr} and yield force F_y will also be high, thereby allowing for an extended Feeding Length. This approach could theoretically enable operation over any distance.

For example, in the demonstration "Long-range operation with a large Feeding Length" (Supplementary Fig. 86, Supplementary Video 19), we replaced the tubes in the Feeding Zone with commercial catheters. The commercial catheter set (WAIN-FBK-6SD110, ASAHI INTECC Co. LTD., JP) contains two catheters: one with an outer diameter of 2.2 mm and an inner diameter of 1.8 mm, and the other with an outer diameter of 2.7 mm and an inner diameter of 2.28 mm; both catheters have a length of 1100 mm. After nesting these two catheters, they were connected with the soft tube we fabricated. The soft tube was placed in a magnetic field, and by manipulating the ends of the commercial catheters, we were able to control the soft tube (see Supplementary Video 19 for details). If the tube in the Magnetic Interaction Zone has a length L_{mi}, then the Effective Operating Distance of the tube in this experiment is

$$D_{eo} = L_{mi} + 1100mm \tag{S9-67}$$

In the video, the soft tube is shown to have a length of 140 mm, so the total Effective Operating Distance in the experiment is 1240 mm. The 1100 mm Feeding Length corresponds to the length of the commercial catheter used. As discussed above, by selecting materials with higher stiffness and yield strength, an even greater Feeding Length could be achieved.

(3) Long-range operation with a large Magnetic Interaction Length and large Feeding Length

For operations in which both the Magnetic Interaction Zone and the Feeding Zone are large, the approach is essentially a combination of the two aforementioned schemes. In each zone, the corresponding method can be applied, and the total Effective Operating Distance is the sum of the Magnetic Interaction Length and the Feeding Length for the respective zones. We will not elaborate further here.

4.1.3 Magnetic Field Design Scheme

When the Magnetic Interaction Zone occupies a large space, the requirements for the magnetic field generation device become more stringent. In this study, a Vibrating Sample Magnetometer (VSM) (EZ7, MicroSense, USA) was employed as the magnetic field generation device, which provides a uniform magnetic field region of approximately 45 mm × 45 mm × 45 mm. For operations involving a large Magnetic Interaction Zone, the conventional approach is to design a large-scale magnetic field generation device to obtain the required magnetic field. Although this method is straightforward, such large-scale devices significantly increase capital investment and can, to some extent, limit the application and dissemination of magnetically controlled technologies. To address this issue, we propose a **multi-magnetic field control method** that employs a spatial combination of multiple magnetic fields to replace a large-scale magnetic field device for controlling the tube.

As shown in Supplementary Fig. 79A, a large-scale magnetic field generation device is used to create the workspace required by the tubes. A schematic of the multi-magnetic field control method is presented in Supplementary Fig. 79B. Based on the tube's specific operational requirements, multiple small-scale magnetic fields are designed and arranged in the regions where deformation is needed; in regions where the tube remains undeformed, the magnetic field can be omitted. This approach employs the spatial distribution of these small-scale magnetic fields to control different sections of the tube. Acting in unison, these fields can achieve the desired tube deformation, effectively replacing the need for a large-scale magnetic field device.

For example, in Supplementary Fig. 84 and Supplementary Video 18, to demonstrate long-range operation, the Magnetic Interaction Length was extended to 305 mm. Fabricating a uniform magnetic field over such a large area to accommodate a tube of that length would be extremely challenging. Therefore, we employed a combination of two Halbach Arrays (see Supplementary Fig. 85), enabling tube operation over 305 mm: the tube undergoes 2D bending in the first magnetic field and achieves multiple 2D or 3D bending deformations in the second magnetic field.

However, when arranging multiple small-scale magnetic fields, mutual interference may occur, and the superposition of several fields in certain areas can generate undesired magnetic fields at specific spatial locations. To address these issues, we conducted a detailed analysis using simulation methods, as described in Supplementary Note 11, in order to provide concrete design guidance.

4.2 Multi-Bend Deformation Operation

In some applications, the tube must perform deformations with multiple bends. The conventional approach is to design an appropriate magnetization profile to achieve these multiple bends. By designing various magnetization profiles, we have demonstrated two planar bends as well as multiple spatial bends within a single magnetic field (Figs. 2c-2f, Supplementary Videos 4-5; Supplementary Figs. 80-83, Supplementary Video 17). However, as the number of bends increases, the complexity of the magnetization profile design also escalates. To reduce this design complexity and facilitate the possibility of incorporating more bending deformations, we propose a **multi-bend deformation design method**. The following sections will explain the principles behind this multi-bend deformation design method and analyze specific design cases.

4.2.1 Multi-Bend Deformation Design Method

Assume that the tube has a total length L and is bent into a certain arc. By dividing the tube along its arc into n segments, each segment s_i has a corresponding length L_i. For the ith segment, the magnetic moment is $\mathbf{m}_i$. Assuming that the tube is placed in a

magnetic field of strength $\mathbf{B}$, the curvature function for the ith segment can be expressed as:

$$\kappa_i(s) = \Gamma(\mathbf{m}_i, \mathbf{B}, \chi) \tag{S9-68}$$

Here, Γ represents a function that incorporates the effects of the magnetic field, magnetic moment, and the physical and geometric parameters of the tube, while χ denotes the tube's physical and geometric parameters. Integrating the curvature with respect to the arc length yields the bending angle for that tube segment, i.e.,

$$\alpha_i = \int_0^{L_i} \kappa_i(s) ds \tag{S9-69}$$

At the same time, we describe the rotation of the local coordinate system due to the bending by employing a rigid transformation. The local rigid transformation corresponding to the bending of the ith segment is given by:

$$R_i = \exp(\alpha_i \widehat{U}_i) \tag{S9-70}$$

where $\widehat{U}_i$ denotes the generator of rotation. Consequently, the final pose of the mth tube segment can be expressed as:

$$R_m = R_m R_{m-1} \cdots R_1 \tag{S9-71}$$

$$\alpha_m = \sum_{i=1}^{m} \alpha_i \tag{S9-72}$$

The final pose of the tube is determined by the rigid transformation R_m and the bending angle α_m, both of which depend on the magnetic field, magnetic moment, and the physical and geometric parameters of the tube (Equation (S9-68)). When a tube with specific physical and geometric parameters is subjected to a magnetic field $\mathbf{B}$, its deformation is dictated by the magnetization profile $\{\mathbf{m}_i\}_1^n$. For complex deformations, such as those involving multiple bends, the magnetization profile becomes correspondingly more intricate.

We propose a multi-bend deformation design method in which multiple bending deformations are decoupled for separate designs. In this approach, different magnetic fields are used to control distinct regions of the tube; each region corresponds to a specific magnetization profile and a particular magnetic field, thereby achieving a defined bending deformation. By combining these individual bending deformations, the desired multi-bending deformation can be realized. We denote the collection of magnetic moments $\mathbf{m}_{\boldsymbol{i}}$ as $\mathbf{m}(\boldsymbol{s})$. Under a single magnetic field, the corresponding curvature function of the tube is given by:

$$\kappa(s) = \Gamma(\mathbf{m}(\boldsymbol{s}), \mathbf{B}, \chi) \tag{S9-73}$$

When multiple magnetic fields are employed to control different regions, for clarity, we denote the collection of magnetic moments (i.e., the magnetization profile) of the tube under the jth magnetic field as:

$$\boldsymbol{m}(\boldsymbol{s})_{\boldsymbol{j}} = \{\mathbf{m}_{\boldsymbol{i}} \mid i = a, a+1, \cdots b\} \tag{S9-74}$$

where a and b denote the indices of the first and last magnetic moments, respectively, under the jth magnetic field. We partition the tube into regions and employ multiple magnetic fields, each corresponding to a specific magnetization profile. Consequently, the curvature function of the tube is given by:

$$\kappa(s) = \begin{cases} \Gamma(\mathbf{m}(\boldsymbol{s})_{\mathbf{1}}, \mathbf{B}_{\mathbf{1}}, \chi), & x \in [0, L_p) \\ \Gamma(\mathbf{m}(\boldsymbol{s})_{\mathbf{2}}, \mathbf{B}_{\mathbf{2}}, \chi), & x \in [L_p, L_q) \\ \vdots & \vdots \\ \Gamma(\mathbf{m}(\boldsymbol{s})_{\boldsymbol{k}}, \mathbf{B}_{\boldsymbol{k}}, \chi), & x \in [L_l, L_n] \end{cases} \tag{S9-75}$$

where $\mathbf{m}(\boldsymbol{s})_{\boldsymbol{k}}$ denotes the collection of magnetic moments (i.e., the magnetization profile) under the kth magnetic field, while p, q, and l represent the indices of the tube segments in different regions.

By comparing Equations (S9-73) and (S9-75), it can be seen that when multiple bends are required, the magnetization profile $\mathbf{m}(\boldsymbol{s})$ designed for a single magnetic field is considerably more complex than the magnetization profiles $\mathbf{m}(\boldsymbol{s})_{\boldsymbol{k}}$ desinged for multiple magnetic fields. Specifically, $|\mathbf{m}(\boldsymbol{s})_{\boldsymbol{k}}| < |\mathbf{m}(\boldsymbol{s})|$ and in some cases,

$\mathbf{m}(s)_k$ is even a subset of $\mathbf{m}(s)$. Furthermore, as the number of tube regions increases, the number of magnetic moments in $\mathbf{m}(s)_k$ becomes increasingly smaller compared to that in $\mathbf{m}(s)$. Therefore, the complexity and design difficulty of $\mathbf{m}(s)_k$ are significantly lower than those of $\mathbf{m}(s)$, indicating that the proposed multi-bend deformation design method greatly reduces the design complexity of the magnetization profile.

When applying the multi-bend deformation design method for magnetizing the tube, the specific magnetization design procedure for a single bend can be found in Supplementary Note 10. Additionally, since this method requires multiple magnetic fields to control different regions of the tube, the magnetic field design scheme described in Section 4.1.3 can be utilized to arrange several magnetic fields appropriately, thereby creating distinct magnetic field regions (see Supplementary Fig. 79B). For a detailed analysis of the magnetic field layout and interference, please refer to Supplementary Note 11.

4.2.2 Design and Optimization Strategies

In this study, the presented magnetization design cases (Supplementary Figs. 2-26) were all developed based on a single magnetic field, covering common modes of operation. For example, we achieved obstacle traversal in a plane using a single bend (Figs. 2c and 2f, Supplementary Videos 4-5), obstacle traversal in a plane via two bends (Fig. 2e, Supplementary Video 4), and obstacle traversal in 3D space through multiple bends (Supplementary Figs. 80-83, Supplementary Video 17). These strategies were implemented by designing various magnetization profiles. To demonstrate the feasibility of achieving multiple bends with multiple magnetic fields, we fabricated two Halbach arrays to generate two uniform magnetic fields arranged at a 45° angle (detailed parameters are provided in Supplementary Figs. 84-85). We manufactured two types of tubes and one rod, and after nesting them, the assembly was placed in the aforementioned magnetic fields for operation. It was observed that, under the first magnetic field, the nested tubes exhibited planar bending, while under the second

magnetic field, the tubes could achieve either planar or spatial bending (Supplementary Video 18).

In the operation demonstrated in Supplementary Video 18, several shortcomings are apparent due to the Halbach array's inability to adjust the magnetic field in real time. First, the tube begins bending before it even enters the Halbach array. This premature bending results from the presence of residual magnetic fields around the periphery of the Halbach array, which cannot be eliminated and thus cause the tube to bend—an effect that is undesirable in practical applications. Second, the video does not incorporate obstacles to showcase the tube's ability to traverse them. Because the Halbach array cannot modulate the magnetic field strength, it does not allow for the gradual increase in the magnetic field—as observed with the magnetic field generated by the VSM (Figs. 2c and 2f, Supplementary Videos 4-5)—that facilitates smooth transitions between different deformation states. To address these shortcomings, future research could explore the following methods. (1) A device could be designed to adjust the diameter of the circular distribution of permanent magnets in the Halbach array, thereby controlling the magnetic field intensity. Our simulations, as shown in Supplementary Fig. 90 (detailed analysis in Supplementary Note 11), indicate that the uniform magnetic field decreases with increasing distance, and when the distribution circle reaches a diameter of 150 mm, the magnetic field is nearly zero. This device would enable real-time control of the magnetic field strength by adjusting the diameter of the magnet distribution circle, and the field direction could be managed by rotating the Halbach array. (2) Instead of employing a Halbach array, the magnetic field can be generated using electromagnetic coils. Similar to our approach with the VSM for control, coils can be designed to produce the desired magnetic field in real time, enabling smooth and continuous adjustments to both its magnitude and direction.

In the multi-bend deformation design described above, the magnetization profile in the first magnetic field (Halbach array) employs Configuration C in 1D magnetization reprogramming (Supplementary Figs. 9-10), while the magnetization profile in the second magnetic field utilizes Configuration G in 1D magnetization reprogramming (Supplementary Fig. 15). This proposed design method transforms the

approach from relying on a single integrated magnetization profile for the entire tube to adopting a segmented, region-based magnetization profile design. The magnetization profile for each region can directly refer to the common operation paradigms we provided (Supplementary Figs. 2-26). This multi-bend deformation design method not only reduces the complexity involved in designing magnetization profiles for intricate deformations but also allows for regional partitioning based on specific requirements, thereby enabling rapid implementation of various deformation designs by leveraging established paradigms.

4.3 High-Curvature Deformation Operation

Based on the analysis in Section 1.1 of this Note, the tube's relative motion resistance $F_{m-total}$, is influenced by the curvature of the tube's deformation under the magnetic field. As the deformation curvature of the tube gradually increases, once $F_{m-total}$ exceeds the tube's critical buckling load F_{cr} and yield force F_y, it will no longer be possible to control the tube's relative motion. In Sections 1, 2, and 3 of this Note, we presented various methods to increase F_{cr} and F_y, while reducing $F_{m-total}$. However, if the tube's deformation curvature increases to a point where $F_{m-total}$ surpasses a critical threshold—rendering any method incapable of satisfying the mechanical requirements for tube motion control (as indicated by Equation (S9-16))—then control over the tube's relative motion will be lost.

For ease of exposition, we summarize the calculation of the relative motion resistance from Section 1.1 using the following equation:

$$F_{m-total} = \Psi(\kappa, \chi) \tag{S9-76}$$

where Ψ represents the mapping function that relates various parameters to the relative motion resistance $F_{m-total}$, κ denotes the curvature of the tube, and χ is the tube's physical and geometrical parameters. We also reference Equation (S9-68) as follows:

$$\kappa = \Gamma(\mathbf{m}, \mathbf{B}, \chi) \tag{S9-77}$$

In Equation (S9-77), the relationships among the parameters were analyzed in Section 1.1 of this Note, indicating that the tube's curvature is positively correlated with $F_{m-total}$. Furthermore, according to the analysis in Supplementary Note 2, under general conditions, both the magnetic moment $\mathbf{m}$ and the magnetic field $\mathbf{B}$ are positively correlated with the curvature κ. Although there may be special cases—such as when switching between specific steady states—where the curvature is not positively correlated with the magnetic moment or magnetic field strength, here we focus on the general scenario and do not address these exceptional cases.

As the tube's curvature increases, the relative motion resistance reaches a critical point beyond which the conditions for relative motion cannot be met. We denote this critical point of the relative motion resistance as $F_{m-total-crit}$. Thus, the condition for the controllability of the tube is

$$F_{m-total} < F_{m-total-crit} \tag{S9-78}$$

Let the curvature corresponding to the critical point be denoted as κ_{crit}. Since there is a positive correlation between curvature and relative motion resistance, the above inequality is equivalently expressed as

$$\kappa < \kappa_{crit} \tag{S9-79}$$

During the process of achieving high-curvature deformations, when the tube's relative motion reaches its critical point and control is lost, the only option is to reduce the tube's relative motion resistance, and then restore control once the adjustment is complete. According to Equation (S9-76), such a reduction is accompanied by a change in the tube's curvature—that is, the tube's shape is altered. If this transient change in shape significantly affects the operation, then temporarily altering the tube's curvature would not be a feasible solution. However, if the impact of the transient deformation is negligible or minor, then a temporary adjustment in curvature is acceptable, with the tube returning to its original shape once control is re-established. Based on Equation (S9-77), a transient reduction in the tube's curvature can be achieved by modulating one of three factors: the magnetic moment, the magnetic field strength, or the tube's

physical and geometrical parameters. In the following sections, we discuss these approaches in detail.

(1) Physical and geometric parameters of the tube

Real-time modulation of the tube's physical or geometric parameters can, to some extent, reduce its curvature. For instance, incorporating stimuli-responsive materials, such as photo-responsive or thermo-responsive compounds, into the tube allows the elastic modulus to be adjusted in real time using external stimuli like light or temperature. This adjustment enables control over the tube's curvature; once the tube's relative motion is re-established, the stimulus can be removed, allowing the tube to revert to its original curvature. Additionally, employing innovative designs to dynamically alter the tube's geometric dimensions or structure, thereby modifying the area moment of inertia, can also serve as a means to control curvature. These approaches present potential avenues for future research; here, we offer these concepts as preliminary ideas without an in-depth discussion.

(2) Magnetization profile of the tube

By reducing the effective magnetic moment of the tube, the force or torque exerted by the magnetic field is decreased, leading to a reduction in the tube's curvature. One approach to modify the effective magnetic moment is through the introduction of an auxiliary tube. For instance, if the relative motion resistance between tube A and tube B under large deformations exceeds the critical threshold—rendering effective magnetic moment adjustment via relative motion unfeasible—the motion of tube C can be utilized. The magnetic moment of tube C can vectorially superimpose onto those of tubes A and B, thereby lowering their effective magnetic moment and enabling the restoration of relative motion control. It should be noted, however, that the introduction of tube C might interfere with other operations, which must be comprehensively considered during the design phase.

(3) External Magnetic Field

Reducing the external magnetic field to decrease the forces or torques acting on the tube is the simplest and most direct approach. In contrast to methods that alter the tube's physical/geometric parameters or modify its magnetization profile, which require

more complex procedures or designs, this method is straightforward. However, it also has certain limitations. For example, it may not be suitable in scenarios involving multiple instruments because adjusting the magnetic field for one instrument may affect the performance of others. Therefore, an optimized design for multiple instruments is necessary to achieve a balance between multi-instrument operation and magnetic field reduction.

In summary, the three schemes for achieving high-curvature deformations essentially rely on temporarily reducing the tube's deformation to lower its relative motion resistance during that time interval and thereby enable control of the tube's relative motion before immediately restoring it to its original state. This approach assumes that such transient deformations have no adverse effect on other operations. Naturally, we aim to avoid reaching the critical level of relative motion resistance in our designs. However, if this cannot be avoided, the methods provided above offer effective alternatives. For example, in this study, we adopted the method of adjusting the external magnetic field. When using Configuration G in 1D magnetization reprogramming to achieve helical deformation (Supplementary Fig. 15, Supplementary Video 1), the tube fabricated with Ecoflex (ECOFLEX™ 0050, KauPo, DE) exhibits low stiffness so that its critical buckling load is lower than the relative motion resistance between tubes. Consequently, during the control of the tube's relative motion, we directly reduced the magnetic field strength to 0 mT. In this manner, control of the tube's relative motion was successfully achieved, after which the magnetic field was restored. For further details, please refer to Supplementary Fig. 15 and Supplementary Video 1.

4.4 Multi-Nested Tube Operation

To reprogram the magnetization profiles, we exploit the relative motion of the tubes. When only a limited variety of magnetization profiles is required, a two-layer tube configuration is sufficient. However, as the number of required deformation types increases, so does the variety of magnetization profiles, which in turn necessitates an increase in the number of tubes. Suppose there are n nested tubes, where the ith tube

has an elastic modulus E_i and an area moment of inertia I_i. The effective bending stiffness of the nested tubes is given by

$$(EI)_e = \sum_{i=1}^{n} E_i I_i$$

(S9-80)

Assuming the nested tube is subjected to a moment M in a magnetic field, the curvature produced according to Euler–Bernoulli beam theory is

$$\kappa = \frac{M}{\sum_{i=1}^{n} E_i I_i}$$

(S9-81)

Thus, when subjected to the same moment, the tube's deformation is directly influenced by its effective stiffness.

When altering the relative positions of the tubes, the number of tubes present in a given region may vary; at one moment, there might be only one tube, while at another, the configuration changes such that the region contains n tubes. For relatively small values of n, the effective stiffness of the nested tubes changes only slightly as the positions vary. For instance, as illustrated in Supplementary Fig. 95A, consider the case of two nested tubes. Suppose that the relative motion of the two tubes occurs entirely within zone A. According to Equation (S9-80), at point A in Supplementary Fig. 95A, initially only tube A is present, so its effective stiffness is $E_1 I_1$. As tube A and tube B undergo relative motion, tube B moves beyond point A, and the effective stiffness then becomes $E_1 I_1 + E_2 I_2$. Similarly, when there are three tubes, the effective stiffness changes from $E_1 I_1$ to $E_1 I_1 + E_2 I_2 + E_3 I_3$, i.e.,

$$(EI)_e = \begin{cases} E_2 I_2, & 1\ tube \\ E_1 I_1 + E_2 I_2, & 2\ tubes \\ E_1 I_1 + E_2 I_2 + E_3 I_3, & 3\ tubes \end{cases} \qquad \text{(S9-82)}$$

The two-layer and three-layer tube nesting configurations presented in this study can effectively achieve the required deformations. However, as the number of tubes n increases, abrupt changes in stiffness may occur in certain regions, which adversely affects the control of tube deformation.

Although Supplementary Figs. 2-26 provides common tube configurations—including two-layer and three-layer tubes—that can be used individually or in combination to satisfy typical operational requirements, the aforementioned design methods may not be adequate in scenarios requiring the nesting of a larger number of tubes. To address this issue, we propose a **zone-based tube nesting design method** as a feasible reference for multi-layer tube nesting designs.

The proposed zone-based tube nesting design method mitigates the issue of excessive stiffness variation during tube operation by partitioning the nested tubes into distinct working zones. As illustrated in Supplementary Fig. 95B, the tubes are divided into several zones, with the number of tubes remaining constant within each zone, and each tube moves exclusively within its designated zone. For example, in Supplementary Fig. 95B, zone 1 comprises only tubes 1-2; zone 2 contains tubes 1-3; zone 3 includes tubes 1-4; and so on. Assuming that zone 1, located at the distal end (i.e., far from the operational end), operates with two tubes, and that a new zone is added for each additional tube, then, due to the nested arrangement, the ith zone will consist of $i+1$ tubes. If the stiffness of the ith tube is denoted by $E_i I_i$, then the maximum effective stiffness of the tubes in the kth zone is given by

$$(EI)_e^k = \sum_{i=1}^{k+1} E_i I_i \tag{S9-83}$$

Thus, in the kth zone, since only the movement of the $(k+1)^{th}$ tube alters the overall stiffness, the maximum stiffness variation in this zone is given by

$$(EI)_{c-max}^k = E_{k+1} I_{k+1} \tag{S9-84}$$

As shown in Equation (S9-84), with the proposed zone-based nesting method, regardless of how many tubes are nested, the stiffness variation in the kth zone is solely determined by the stiffness of the $(k+1)^{th}$ tube. This single-tube-induced stiffness change can be effectively controlled. In contrast, when the zone-based nesting design is not employed and all tubes operate within a single zone, the maximum stiffness variation in the kth zone is given by

$$(EI)_{c-max}^{k} = \sum_{i=2}^{k+1} E_i I_i$$

(S9-85)

Comparing Equations (S9-84) and (S9-85) reveals that the proposed method dramatically reduces the stiffness variation by an amount of $\sum_{i=2}^{k} E_i I_i$. When only a small number of tubes are nested, the maximum stiffness variation differs only slightly between using and not using the proposed method. For example, in a two-tube configuration, both approaches yield the same stiffness variation. In a three-tube configuration, the proposed method results in a maximum stiffness variation of either $E_2 I_2$ or $E_3 I_3$ (depending on which is greater), whereas without the proposed method, the maximum stiffness variation is $E_2 I_2 + E_3 I_3$. As the number of tubes increases, the difference in stiffness changes becomes more pronounced, demonstrating that the zone-based nesting design method can effectively mitigate this issue.

Moreover, as the number of nested tubes increases, corresponding adjustments to the magnetization profiles and magnetic fields become necessary for deformation control. We discuss these modifications separately in terms of single-magnetic field control and multi-magnetic field control.

(1) Single-Magnetic Field Control

When employing single-magnetic field control, all tubes operate under the same magnetic field, meaning that every nested tube is subjected to the same magnetic field strength. Since the effective stiffness of the tubes varies across different zones and progressively increases with the zone number, the design of the magnetization profiles must likewise be adjusted so that each zone receives sufficient force or moment to achieve the desired deformation. Suppose the magnetic moment of the magnetic unit in the jth zone is denoted as $m(j)$ and then we have

$$m(j) = \begin{cases} m_1, & j = 1 \\ m_2, & j = 2 \\ \vdots & \vdots \\ m_n, & j = n \end{cases}$$

(S9-86)

$$m_1 < m_2 < \cdots < m_n \tag{S9-87}$$

$$m(j) = \hbar\left(E_j I_j, \gamma\right) \tag{S9-88}$$

where $\hbar$ represents the mapping function from the tube's effective stiffness (and other related parameters) to the magnetic moment, while γ denotes the tube's dimensions and other related parameters. Since the tube's effective stiffness gradually increases with the number of nested tubes, the design method described above can be employed to ensure that the magnetic moment also increases progressively, thereby mitigating the significant stiffness variations induced by multi-tube nesting.

(2) Multi-Magnetic Field Control

In Section 4.1.3 of this note, we proposed a multi-magnetic field control method that can be integrated with the zone-based tube nesting design. In this approach, a separate magnetic field is applied to each zone, or to every few zones, as illustrated in Supplementary Fig. 96. For instance, magnetic field 1 may be used to control zone 1 or zones 1-2. Suppose that each zone is controlled by a dedicated magnetic field, with the magnetic field strength in the jth zone denoted as $B(j)$; then, we have

$$B(j) = \begin{cases} B_1, & j = 1 \\ B_2, & j = 2 \\ \vdots & \vdots \\ B_n, & j = n \end{cases} \tag{S9-89}$$

$$B_1 < B_2 < \cdots < B_n \tag{S9-90}$$

$$B(j) = h\left(E_j I_j, \gamma\right) \tag{S9-91}$$

Here, h represents the mapping function from the tube's effective stiffness (and other related parameters) to the magnetic field strength. By adopting the aforementioned design—where the magnetic field strength increases gradually—we can ensure that sufficient force or moment is generated in zones with a larger number of tubes to induce the desired deformation. In the above approach, each magnetic field is assigned to one zone; however, in practical applications, one magnetic field may be used to control two or more zones, but typically no more than three, depending on the specific design requirements.

The single-magnetic field and multi-magnetic field control strategies address the design from the perspectives of magnetic moment and magnetic field strength, respectively, thereby ensuring that the forces/moments acting on tubes in different zones vary appropriately. These two methods can be employed individually or in combination, with the choice of adjusting the magnetic moment, the magnetic field, or both simultaneously determined by the particular application to achieve optimal control performance.

To demonstrate the feasibility of the proposed approach, we integrated it with the multi-magnetic field control method described in Section 4.1.3 above. The specific design and experimental setup are illustrated in Supplementary Figs. 84-85. We fabricated Tube A, Tube B, and a rod. Tube A consists of two layers of tubes, as shown in Supplementary Video 18. Specifically, the outermost black tube is directly fixed onto the next outermost red tube. However, in Supplementary Fig. 84, we simplified this arrangement according to its configuration and labeled it as Tube A with only one layer. Therefore, although Supplementary Fig. 84 depicts a three-layer tube configuration, it is actually composed of four layers. We divided the system into two zones: in one zone, tube B and the rod are present, while in the other, tube A, tube B, and the rod coexist. These two zones correspond to the two magnetic field regions generated by the Halbach arrays shown in Supplementary Fig. 85, which in turn relate to the multi-bend deformation operation described in Section 4.2. According to Equation (S9-87), the magnetic unit installed in tube A is designed with a relatively large magnetic moment to induce deformation in tube A, tube B, and the rod. In the Halbach array closer to the operational end, only tube A is magnetized, so that under the resulting forces it drives the embedded tube B and rod to deform; in the Halbach array farther from the operational end, tube A is absent, allowing tube B and the rod to move relative to each other and achieve the desired deformation. The detailed operation is shown in Supplementary Video 18. Since this design combines several methods, additional discussions can be found in Section 4.2.2.

Please note that, in Sections 4.1-4.4 above, we proposed four new methods—namely, a segmented design method, a multi-magnetic field control method, a multi-

bend deformation design method, and a zone-based tube nesting design method—to address specific challenges in long-range operation, multi-bend deformation, high-curvature deformation, and multi-nested tube operation, respectively. In the experimental demonstrations, some designs incorporate multiple methods simultaneously. As a result, certain demonstrations/videos are referenced multiple times, serving as demonstrations or validations for different design strategies.

Supplementary Note 10:

Design of Magnetization Profile

The deformation of soft robots under a magnetic field depends on both the external magnetic field and the intrinsic magnetization profile. We presented common configurations and deformations of 1D, 2D, and 3D structures in Supplementary Figs. 2-26, covering widely used paradigms that can accommodate most application needs. To establish a more general design approach, we provide the following three design methods as references for broader design applications: **Forward Design Method**, **Inverse Design Method**, and **Template-Based Design Method**. Since the deformation of a 1D tube serves as the foundation for 2D and 3D structures, we focus our analysis on the deformation of a 1D soft tube, while the deformation of 2D and 3D structures can be extended based on the 1D case.

1. Forward Design Method

For relatively simple deformation designs, empirical knowledge or an exhaustive search approach can be used. Once the design is completed, theoretical analysis or simulations are employed before fabrication or testing to validate and refine the design, ensuring feasibility. At this stage, the specific magnetization profile of the soft tube is determined, allowing the Forward Design Method to be applied. By utilizing the known magnetization profile, applied magnetic field, and other given parameters, the potential deformations can be predicted.

The detailed computational method is provided in Supplementary Note 2. In this approach, the pseudo-rigid-body model is adopted, approximating the soft tube as a system composed of multiple rigid rods. Gravitational potential energy, magnetic potential energy, and elastic potential energy are considered in formulating the total potential energy function. If additional external factors are present, they can also be incorporated into the analysis. The total potential energy function can be expressed as:

$$V = U_m + U_g + U_e + U_o \tag{S10-1}$$

where U_m represents the magnetic potential energy of the system;

U_g means the gravitational potential energy of the system;

U_e represents the elastic potential energy of the system;

U_o is the potential energy contributed by other possible external factors.

Based on the principle of minimum potential energy, the fmincon function in MATLAB can be used to determine the joint angles θ between rigid rods, thereby obtaining the final deformation of the soft tube. Alternatively, the modeling approach described in the "Materials and Methods" section can be employed, using ABAQUS to simulate the deformation of soft tubes, 2D sheets, or 3D bodies. The Forward Design Method is used to solve the forward problem, where the computation is relatively simpler compared to the Inverse Design Method, and the solution is generally unique.

2. Inverse Design Method

Compared to the Forward Design Method, the Inverse Design Method requires less prior experience from the designer but poses greater mathematical challenges. The following section introduces the approach for determining the magnetization profile of a soft tube using the Inverse Design Method.

When solving the forward problem, the pseudo-rigid-body model is employed, and the principle of minimum potential energy is used to determine the joint angles between rigid rods. A similar approach can be applied to solving the inverse problem. The soft tube is assumed to consist of n rigid rods, each containing a magnetic unit with a variable magnitude and direction. The direction of each magnetic unit is influenced by the position of its corresponding rod. Consequently, the magnetic moment of each unit can be expressed as:

$$\mathbf{m_i} = \mathbf{m_i}(\chi, \theta) \tag{S10-2}$$

Here, χ represents the magnetization parameter of the magnetic units, defined as $\chi = (\chi_1, \dots, \chi_n)$, which are the unknown parameters to be identified. θ denotes the joint angles between the rigid rods, given by $\theta = (\theta_1, \dots, \theta_n)$. Based on the analysis in

Supplementary Note 2, the total magnetic potential energy of the soft tube can be directly obtained as:

$$U_m(\chi,\theta) = -\sum_{i=1}^{m} \mathbf{m}_{\boldsymbol{i}}(\chi,\theta)\cdot\mathbf{B}_{\boldsymbol{i}}$$

(S10-3)

where $\mathbf{B}_{\boldsymbol{i}}$ represents the magnetic field acting on the ith magnetic unit. Similarly, the elastic potential energy and gravitational potential energy of the soft tube can be expressed as:

$$U_e(\theta) = \frac{2n}{L}\sum_{i=1}^{n} E_i I_i \theta_i^2$$

(S10-4)

$$U_g(\theta) = -\frac{1}{2}\sum_{i=1}^{n} m_i^m(\mathbf{p}_{\boldsymbol{i}}^{\boldsymbol{f}} - \mathbf{p}_{\boldsymbol{i}}^{\boldsymbol{i}})\cdot\mathbf{g}$$

(S10-5)

where E_i represents the modulus of elasticity of the ith rod;

I_i is the moment of inertia of the cross-section of the ith rod;

m_i^m represents the weight of the ith magnetic unit;

$\mathbf{p}_{\boldsymbol{i}}^{\boldsymbol{f}}$ means the initial position vectors of the ith rod;

$\mathbf{p}_{\boldsymbol{i}}^{\boldsymbol{i}}$ is the final position vectors of the ith rod;

$\boldsymbol{g}$ is the acceleration due to gravity.

$\mathbf{p}_{\boldsymbol{i}}^{\boldsymbol{i}}$ and $\mathbf{p}_{\boldsymbol{i}}^{\boldsymbol{f}}$ can be determined based on the joint angles between the rigid rods. For detailed calculations, please refer to Supplementary Note 2.

If the soft tube is influenced by additional factors that may affect either the magnetization of the magnetic units or the joint angles between the rigid rods, these effects are denoted as $U_o(\chi,\theta)$. Thus, the total potential energy of the soft tube system can be expressed as:

$$V(\chi,\theta) = U_m(\chi,\theta) + U_g(\theta) + U_e(\theta) + U_o(\chi,\theta) \quad \text{(S10-6)}$$

A specific magnetization parameter χ is first assigned to the equation, and a search is conducted within the θ space to determine $\theta^*(\chi)$, which minimizes the total potential energy. $\theta^*(\chi)$ can be expressed as:

$$\theta^*(\chi) = arg\ \min_{\theta} V(\chi, \theta) \tag{S10-7}$$

By solving the above equation, the magnetization parameter χ is mapped to the equilibrium deformation parameter $\theta^*(\chi)$. In the pseudo-rigid-body model, the deformation of the soft tube is governed by the joint angles θ between the rigid rods. To ensure that the obtained deformation matches the target deformation, functions are defined as:

$$\Omega(\chi) = ||\theta^*(\chi) - \theta^{target}||^2 \tag{S10-8}$$

$$\chi^* = arg\ \min_{\chi} \Omega(\chi) \tag{S10-9}$$

where θ^{target} represents the set of joint angles in the target deformation;
χ^* denotes the magnetization parameter that minimizes $\Omega(\chi)$.

In the above computation, the smaller $\Omega(\chi)$ is, the better. As it approaches zero, the obtained solution $\theta^*(\chi)$ becomes increasingly close to the target value θ^{target}. However, in practical applications, achieving an exact zero is unnecessary as long as the deformation meets the application requirements. A smaller $\Omega(\chi)$ leads to higher deformation control accuracy but significantly increases computational cost. Therefore, instead of minimizing $\Omega(\chi)$ indefinitely, it is sufficient to ensure that it meets a predefined threshold δ, given by:

$$\Omega(\chi) \leq \delta \tag{S10-10}$$

where δ represents the threshold for the error between the actual deformation and the target deformation, ensuring that it meets application requirements.

We set Equation (S10-7) as the inner function while defining Equation (S10-8) as the outer function. A numerical iteration method is used to solve the equations

iteratively. In the inner function, a search is conducted to determine $\theta^*(\chi)$, while in the outer function, $\Omega(\chi)$ is optimized. For each iteration, the computed joint angles are compared with the desired angles, and the distribution of the magnetization profile is adjusted accordingly. After multiple iterations, the joint angles of all rods gradually converge to the expected values, ultimately yielding a magnetization profile that meets the required criteria.

During the optimization process, the magnetization parameter χ may experience abrupt variations, leading to an unsmooth magnetization distribution or numerically extreme solutions. To obtain a feasible solution, regularization can be applied to the outer optimization function. In this case, Equation (S10-8) can be extended as:

$$\Omega_{reg}(\chi) = \Omega(\chi) + \mu\rho(\chi) \tag{S10-11}$$

where μ is the coefficient of the regularization term, and $\rho(\chi)$ represents the regularization function.

The coefficient μ is used to adjust the weight of the regularization term, while $\rho(\chi)$ is introduced to constrain excessive variations in the magnetization parameter χ. The specific form of $\rho(\chi)$ can be determined based on practical requirements. For instance, to prevent excessive fluctuations in magnetization, Tikhonov Regularization can be introduced. By penalizing the sum of the squared magnetization parameters, the optimization process becomes more stable and avoids excessively large numerical values. This can be expressed as:

$$\rho(\chi) = \|\chi\|^2 \tag{S10-12}$$

Another example is that, due to manufacturing constraints, the maximum magnetization magnitude of certain substrate materials may be significantly limited, requiring an upper bound on the magnetization magnitude. This can be enforced using Physical Constraints to restrict its maximum value. The corresponding regularization term can be expressed as:

$$\rho(\chi) = \sum \max(0, |\chi_j| - \chi_{max})^2 \tag{S10-13}$$

Where χ_{max} represents the maximum allowable value of the magnetization parameter. Other regularization methods, such as Total Variation Regularization, can also be introduced to impose different types of constraints on the function.

The bi-level optimization approach described above is relatively simple and easy to implement, making it suitable for problems of smaller scale. For extensive computations, a more efficient approach is to optimize θ and χ simultaneously, avoiding the need for nested iterations. When solving the Inverse Design Method, obtaining a valid solution is not always guaranteed, and the solution may not be unique. The approach outlined above provides a fundamental framework for implementing the Inverse Design Method. However, numerous studies have been dedicated to addressing inverse problems (*73*, *74*), and these methods continue to evolve. Existing advancements in inverse problem-solving techniques can be referenced for further improvements.

3. Template-Based Design Method

3.1 Design Steps

Among the previously discussed design methods, both the Forward Design Method and the Inverse Design Method require accounting for the interactions among various parameters, such as the magnetization profile, magnetic field, elasticity, and gravity, which can be rather cumbersome. A simpler alternative is the Template-Based Design Method, which employs a template to facilitate the design process, thereby directly bypassing the interdependent constraints among the parameters. The specific design steps for the Template-Based Design Method are as follows:

(1) Tube fabrication

Fabricate a tube with the desired diameter d_{des} and length L_{des} according to the specific requirements. The tube should be made from a composite of a soft material and magnetic particles, with the soft material (e.g., PDMS or Ecoflex) serving as the substrate. The amount of magnetic particles can be adjusted based on the required magnetization magnitude; typical mixing ratios and corresponding magnetization

magnitudes are shown in Supplementary Fig. 66. The fabrication methods for the tube (or rod) are illustrated in Supplementary Figs. 67-68.

(2) Template fabrication

Create an appropriate template based on the specific application requirements. For instance, if a circular deformation is desired, design the mold using parameters of the circular shape, including the desired median diameter D_{des}, spanning angle θ_{des}, and tube diameter d_{des} (see Supplementary Fig. 72A).

(3) Magnetization process

Secure the tube within the template and place it in a strong magnetic field for magnetization. The tube is forced to conform to the shape defined by the template (e.g., circular), and during magnetization, the magnetic units within the curved tube align parallel to the magnetic field (see Supplementary Fig. 72B).

(4) Template removal and profile acquisition

Remove the tube from the template to obtain the desired magnetization profile. After the template is removed, the tube, under its own elastic recovery, reverts to a straight configuration while retaining a specific magnetization profile that varies with the axial distance (see Supplementary Fig. 72B).

When the magnetized tube is placed in a uniform magnetic field, it will become a ring (Supplementary Fig. 72C). In this design process, one approach is to mix magnetic particles with the substrate material and subsequently magnetize the composite to obtain the magnetization profile; alternatively, permanent magnets may be employed. Using magnetic particles for magnetization is relatively straightforward, although its magnetization magnitude tends to be lower. For instance, when PDMS is used as the substrate, experimental observations and empirical evidence indicate that a mass ratio of 3:1 (magnetic particles to PDMS) offers a good compromise between material pliability and a relatively high magnetization magnitude. Under these conditions, the average magnetization magnitude is approximately 1.88×10^5A/m（Supplementary Fig. 66）, whereas N50 or N52 permanent magnets can easily achieve a magnetization magnitude on the order of 10^6A/m. If alternative substrate materials, such as the Ecoflex

series, are used, the proportion of magnetic particles that can be incorporated is lower, resulting in a magnetization magnitude significantly below 1.88×10^5A/m.

Replacing magnetic particles with permanent magnets can yield a considerably higher magnetization magnitude, which either enables larger deformations or reduces the requirement for high external magnetic field strengths. However, this approach introduces increased design complexity. When employing permanent magnets instead of magnetic particles, a process similar to that depicted in Supplementary Fig. 72 is followed: a fabricated tube, devoid of magnetic particles, is installed in a template and then magnetized within a magnetic field. The key difference is that the tube must have pre-designated positions for mounting the permanent magnets. In this case, only a small magnetic field (generally below 20 mT) is required to reorient the permanent magnets, and the field is maintained until the material securing the permanent magnets cures.

3.2 Parameter Tuning Methods

This method enables the design of templates with arbitrary deformation shapes according to specific deformation requirements, thereby achieving the desired deformation. For example, in Supplementary Fig. 15, a helical deformation is obtained by employing a cylindrical template featuring spiral grooves. This approach is simple and practical, requiring minimal theoretical or design expertise. However, it is important to note that the deformations achieved by this method are inherently gradual. For instance, if a square template is used in an attempt to produce a square deformation, even though the magnetization directions align with a square configuration, the soft tube will not exhibit a perfect square deformation when subjected to a magnetic field. This discrepancy occurs because the magnetization profile undergoes abrupt changes at the square's corners, while the bending deformation of the soft tube transitions gradually, leading to a mismatch. The primary drawback of this method is the need for post-process tuning. Even if the magnetization profile is gradual, factors such as gravity and the tube's inherent elasticity may cause the resulting deformation to deviate from the template shape, necessitating adjustments based on the specific design. For example, in Supplementary Fig. 15, the template used has a helical diameter of 12 mm and a pitch

of 0.3 mm, yet the deformation achieved with this template is consistently larger than these dimensions. The following outlines tuning strategies, categorized by external influencing factors, using helical deformation as an illustrative example.

(1) Effects of Gravity

As shown in Supplementary Fig. 73, a template with a helical diameter D_{des}, a pitch p_{des}, and a groove cross-sectional diameter d_{des} is used to magnetize the soft tube. Here, D_{des}, p_{des}, and d_{des} represent the desired helical outer diameter, pitch, and groove cross-sectional diameter, respectively. If the soft tube is oriented vertically along the helical axis during operation (as illustrated in Supplementary Fig. 73C), the effect of gravity induces a moment $\mathbf{M}^{\mathrm{g}}$ that causes the helical tube's pitch to change from p_{des} to $p_{des} + \Delta p$. Δp denotes the deviation from the desired pitch and it can be expressed as

$$\Delta p = \omega(\sum \mathbf{M}_i^g) \tag{S10-14}$$

where ω is the function describing the relationship between the soft tube's pitch variation and the bending moment (which depends on the physical properties and geometric characteristics of the soft tube), and $\mathbf{M}_i^{\mathrm{g}}$ is the gravitational moment acting on the ith magnetic unit.

To reduce or eliminate this pitch error Δp, the following three methods can be adopted.

(i) Increasing the magnetic field strength

Enhance the magnetic field from the originally specified value $\mathbf{B}$ to $\mathbf{B} + \Delta\mathbf{B}$ so that the magnetic moment acting on the soft tube is augmented, thereby compensating for the deformation induced by the gravitational moment. For a uniform magnetic field, when the magnetic field strength is increased from its predetermined value, the pitch error is given by:

$$\Delta p = \omega[\sum \mathbf{M}_i^g - \sum(\mathbf{m}_i \times \Delta\mathbf{B})] \tag{S10-15}$$

where $\mathbf{m}_i$ represents the magnetic moment of the ith magnetic unit. As the magnetic field strength increases, the pitch error decreases directly. It is important to note that, in

the above equation, even if the magnetic field-generated moment exceeds the gravitational moment, the pitch error will not become negative. This is because the magnetization profile designed by the Template-Based Design Method progressively approaches the shape defined by the template (Supplementary Fig. 73B) as the magnetic field increases, rather than inverting to a reversed helix.

This method is the simplest and most direct; however, its drawbacks are also evident. Increasing the magnetic field is constrained by the external magnetic field generation system and specific application conditions, so the magnetic field strength cannot be increased indefinitely. Moreover, gravity also exerts a stretching effect on the soft tube along the direction of gravity, which cannot be compensated by the magnetic field. If error tolerances are not particularly stringent or if there are no strict requirements on the magnetic field strength, this method can be employed to achieve the desired deformation in a simple and rapid manner.

(ii) Increasing magnetization magnitude

This approach is analogous to increasing the magnetic field strength. Since the force or moment generated by a magnetic field depends on both the magnetization and the magnetic field, enhancing either parameter can counteract gravitational deformation by introducing a larger force or moment to balance gravity. By increasing the magnetic moment of an individual magnetic unit from $\mathbf{m}_i$ to $\mathbf{m}_i + \Delta\mathbf{m}_i$, the pitch error can be expressed as:

$$\Delta p = \omega[\sum \mathbf{M}_i^g - \sum(\Delta\mathbf{m}_i \times \mathbf{B})] \tag{S10-16}$$

During the fabrication of the soft tube, increasing the magnetic particle content can elevate the magnetization magnitude, as a higher proportion of magnetic particles directly results in greater magnetization magnitude (see Supplementary Fig. 66B). However, it is important to note that a higher magnetic particle content also leads to a significant increase in the tube's weight due to the high density of the particle. This creates a trade-off that must be carefully balanced based on the specific geometric parameters. To address the additional weight introduced by increasing the magnetic particle, a magnetic material with a high magnetization-to-weight ratio is required (i.e.,

a material where the increase in magnetization magnitude outweighs the added weight). Permanent magnets offer an excellent solution in this regard; however, as previously discussed, their use introduces additional design complexity and challenges. Ultimately, the choice between magnetic particles, permanent magnets, or a combination of both should be determined by the specific application requirements.

(iii) Reverse pitch compensation design

In this approach, the template's pitch is intentionally designed to be smaller than the desired pitch—that is, reduced from p_{des} to $p_{des} - \Delta p$. During operation, gravity induces an additional pitch change of p_g. By setting the reverse compensation value Δp equal to p_g, the resulting pitch becomes:

$$p_f = p_{des} + p_g \tag{S10-17}$$

$$p_f = p_{des} - \Delta p + \Delta p = p_{des} \tag{S10-18}$$

As shown by the above equation, the final pitch will be p_{des}, precisely meeting the operational requirements. This design leverages the elastic recovery of the soft tube to counteract the gravitational effect, thereby achieving the desired deformation. Although this method is relatively simple and straightforward, it requires multiple tuning iterations to reliably attain the expected pitch p_{des}.

The three methods described above pertain to the specific tuning procedures when the soft tube is oriented vertically. In practical applications, the soft tube can be positioned arbitrarily, and similar tuning strategies based on the aforementioned principles can be applied accordingly.

(2) Effects of Elasticity

In the Template-Based Design Method, the soft tube is initially deformed into various shapes by the template. Upon removal of the template, the principle of minimum potential energy drives the soft tube to gradually revert toward its original configuration (for instance, if the soft tube is fabricated using the dip coating method, it will naturally tend to straighten). Consequently, the greater the deviation of the designed deformation from the original shape, the higher the associated elastic potential

energy. When the soft tube is subsequently placed in a magnetic field to induce deformation, a corresponding force or moment is required to overcome this elastic resistance.

Taking helical deformation as an example, we fabricated a soft tube via the dip coating method and magnetized it using a template with a designed outer diameter D_{des}, pitch p_{des}, and groove cross-sectional diameter d_{des}. Due to the soft tube's elasticity, both the pitch and the outer diameter will deviate from their designed values, changing from p_{des} to $p_{des} + \Delta p$ and from D_{des} to $D_{des} + \Delta D$, respectively. To compensate for the increased pitch error Δp and outer diameter error ΔD, the same three methods previously discussed for reducing gravitational effects can be employed. Specifically, the pitch error can be reduced by increasing the magnetic field strength, increasing the magnetization magnitude, or implementing a reverse compensation design for the pitch. In terms of addressing the outer diameter error, besides enhancing the magnetic field strength and magnetization magnitude, a reverse compensation strategy analogous to that used for the pitch—namely, a reverse compensation design for the helical diameter—can be applied.

In the reverse compensation design for the helical diameter, the designed helical diameter is reduced from D_{des} to $D_{des} - \Delta D$. Similarly, when the soft tube is placed in a magnetic field, its inherent elasticity causes its diameter to spontaneously increase, thereby compensating for the diameter error ΔD. However, this compensation is not always feasible because, as the helical diameter decreases from D_{des}, the elastic potential energy rises rapidly. Beyond a certain threshold, the elastic potential energy may exceed the compensatory capacity of the magnetic force or moment. For example, in our experiments, a template with an outer diameter of 4 mm, a pitch of 2 mm, and a groove cross-sectional diameter of 1.9 mm was used to magnetize a soft tube fabricated from PDMS (using a 10:1 PDMS formulation as the substrate, with magnetic particle mixed in a 3:1 ratio by weight, yielding a straight tube with a diameter of 1.9 mm). However, even when employing a magnetic field several times stronger than the originally specified value (300 mT), the desired deformation could not be achieved. This failure is attributed to the fact that transforming the tube from its initial

configuration to the preset helical shape requires storing an excessive amount of elastic potential energy, which the magnetic field cannot provide sufficient work to overcome.

In such cases, an alternative approach is to reduce the elastic potential energy. The elastic potential energy U_e is positively correlated with both the elastic modulus E and the strain s, as expressed in the following equation:

$$U_e \propto E \tag{S10-19}$$

$$U_e \propto s^2 \tag{S10-20}$$

Based on these relationships, one feasible approach is to reduce the elastic modulus of the soft tube, thereby lowering its elastic potential energy. An alternative strategy is to reduce the deformation magnitude. By fabricating the soft tube with an initial shape that is helical or approximately helical, the relative deformation during the final actuation will be smaller, thus reducing the required elastic potential energy to overcome, and enabling the desired deformation. However, this approach results in an initial configuration that is helical (or near-helical) rather than straight, so whether it satisfies specific operational requirements must be carefully evaluated through detailed design.

(3) Effects of Other Factors

The previous discussion specifically addressed design strategies to mitigate the effects of gravity and inherent elasticity. In practical applications, additional influencing factors may also be present. If these factors are static, similar compensation strategies—akin to those used for gravity and elasticity—can be incorporated during the design stage. By accounting for their impact on the deformation magnitude in the preliminary design, these static influences can be readily eliminated. Conversely, if the influencing factors are dynamic, pre-design compensation is insufficient. In such cases, it becomes necessary to determine specific parameters based on the dynamic environment, which can be achieved through real-time adjustments of the magnetic field or magnetization.

Several applications presented in this study involve similar dynamic operations, albeit not strictly falling under this category. These examples are mentioned here to illustrate the concept of dynamic compensation. For instance, in the “contact-free object navigation” section, as the soft tube enters and exits a blood vessel, its relative position with respect to the vessel changes. Consequently, the curvature radius of the soft tube must be adjusted in real time to accommodate these positional variations. This was achieved by dynamically altering the magnetic field (Fig. 2f and Supplementary Video 5). Similarly, in the “reprogrammable cilia array” section, the bending amplitude of the cilia needs to be modified to meet operational demands and this adjustment was realized by altering the magnetization of the cilia (Fig. 3d and Supplementary Video 8).

The methods described above for compensating for various influencing factors provide relatively simple and direct tuning strategies that enable the rapid attainment of the desired deformation. However, if higher precision is required, more accurate tuning becomes necessary. In such cases, the optimal approach is to combine the Template-Based Design Method with the Forward Design Method outlined in Section 1, thereby allowing for precise calculations of these influencing factors and achieving the desired deformation with higher accuracy.

4. Discussion

By employing the Forward Design Method, the Inverse Design Method, and the Template-Based Design Method, the magnetization profile required for a specific deformation can be obtained. For different functional requirements, multiple deformations are designed, namely,

$$F_i = g(D_i), \quad i \in \{1,2,3,\dots\} \tag{S10-21}$$

where F_i corresponds to distinct functional requirements, g is the mapping function between functionality and deformation, and D_i represents different deformations. Each deformation can be realized through a unique magnetization profile:

$$D_i = h(MP_i), \quad i \in \{1,2,3,\dots\} \tag{S10-22}$$

where h is the mapping function from the magnetization profile to the resulting deformation, and MP_i denotes different magnetization profiles. In this study, the magnetization profile of the soft tube can be modified in real time to obtain various MP_i. To achieve different deformations D_i by dynamically altering the magnetization profiles MP_i, it is essential to consider how these different profiles can be implemented through the nesting of multiple soft tubes and variations in their positions, as well as the transitions between various deformations. The primary challenge lies in the design of the soft tube, which heavily depends on design experience. This requires the empirical development of diverse magnetization profiles, various configuration schemes, and switching mechanisms to enable transitions among the desired deformations. However, at present, it is not feasible to provide a comprehensive, systematic summary of such design experience. Instead, numerous design cases (Supplementary Figs. 2-26) are presented, covering the current widely used paradigms, which are believed to encompass most practical application scenarios. Each case includes detailed design strategies and experimental demonstrations of specific deformations, thereby serving as both a reference for practical applications and an inspiration for future design endeavors.

Supplementary Note 11:

Analysis of Multi-Magnetic Field Interference

For operations requiring a large working space within a Magnetic Interaction Zone, the conventional approach involves designing large-scale magnetic field generators to achieve the desired magnetic fields. However, such large-scale magnetic field generators significantly increase equipment costs, thus restricting the broader application and promotion of magnetically controlled technologies. To address this issue, we propose a multi-magnetic field control method in Supplementary Note 9, which involves spatially combining multiple small-scale magnetic fields to replace a single large-scale magnetic field for controlling soft robots. When utilizing multiple small-scale magnetic fields to control different regions of soft robots, these smaller fields need to be strategically arranged in space. This spatial arrangement inevitably leads to interference among the fields, with overlapping regions potentially producing undesired magnetic fields at certain locations. Therefore, we conducted simulation analyses on the interference among multiple magnetic fields to provide practical references for implementing the multi-magnetic field control method. In our application involving long-range operations with a large Magnetic Interaction Length (as shown in Supplementary Fig. 85), we employed a Halbach array to generate the required magnetic fields. Accordingly, we selected the Halbach array as our simulation model and carried out simulations using COMSOL Multiphysics software (version 6.2, COMSOL Inc., Sweden).

We selected twelve N52-grade neodymium permanent magnets, each rectangular in shape and measuring 12 mm×12 mm×30 mm, and arranged them in a circular configuration following the Halbach array pattern. Two identical Halbach arrays were employed to generate two magnetic fields, designated as the upper magnetic field ($\mathbf{B}_{up}$) and lower magnetic field ($\mathbf{B}_{down}$), respectively, as depicted in Supplementary Fig. 91A. The angle between $\mathbf{B}_{up}$ and $\mathbf{B}_{down}$ is denoted as φ, and the distance between the end faces of the two Halbach arrays is denoted as L (defined here as the magnetic field spacing). While the lower Halbach array remains fixed, the upper

Halbach array rotates around an axis perpendicular to the plane of the paper, with an origin located at a specific quadrant point on its end face. The angle formed between the end faces of these two Halbach arrays during this rotation is defined as the magnetic field plane angle (α), as illustrated in Supplementary Fig. 93A.

For a single magnetic field, we investigated how the diameter of the magnet distribution circle D influences the magnetic field strength. The diameter was increased incrementally from an initial value of 60 mm, with increments of 5 mm, until the magnetic field intensity in the central region approached approximately 0 mT. Seven sampling points (labeled A-G) and one sampling line (along the z-axis) were selected, with their specific positions illustrated in Supplementary Fig. 90A. As demonstrated in Supplementary Figs. 90B-90C, the magnetic field intensity gradually decreases as the diameter of the magnet distribution circle D increases. When the diameter of the magnet distribution circle D reaches 150 mm, the magnetic field intensity in the central region approaches nearly zero. In Section 4.2.2 of Supplementary Note 9, we discussed the possibility of designing an adjustable device capable of varying the diameter of the magnet distribution circle within the Halbach array, thereby enabling control over the magnets and consequently altering the magnitude of the magnetic field. The simulation results provide theoretical support for this approach. Therefore, future research could employ a similar adjustable device to achieve effective control of the magnetic field strength. Supplementary Fig. 90D presents magnetic field intensity distributions for various magnet distribution circle diameters using magnetic flux lines. It is evident that a smaller distribution circle diameter corresponds to denser magnetic flux lines, thus indicating a stronger magnetic field. Additionally, Supplementary Fig. 90C illustrates the magnetic field's influence on surrounding regions. Along the z-axis, at distances greater than 50 mm from the Halbach array center, the magnetic field intensity effectively reduces to 0 mT. This distance may serve as a reference for the safe separation required to prevent interference from a single Halbach array with non-magnetic regions, and as a valuable parameter for future design considerations.

To explore how varying the magnetic field angle φ affects the magnetic field in different regions, we set φ from 0° to 180° in increments of 30°. The diameter of the magnet distribution circles D in both Halbach arrays was set to 75 mm, and the magnetic field spacing L between the two arrays was fixed at 10 mm. Additionally, the magnetic field plane angle α was set to 0°, meaning the two end faces were parallel. Seven sampling points (points A-G) and one sampling line (along the z-axis) were chosen, with their specific positions detailed in Supplementary Fig. 91A. According to the simulation results (Supplementary Figs. 91C-91D), as φ increased from 0° to 90°, the magnetic field intensity gradually decreased; conversely, as φ increased from 90° to 180°, the intensity progressively increased. When the magnetic field angle reached 90°, the magnetic field intensity in the central region achieved its minimum, with an area exhibiting zero magnetic intensity. As illustrated by the magnetic flux lines in Supplementary Fig. 91E, at φ = 90°, no magnetic flux lines appear in a certain central region, indicating a magnetic field strength of approximately 0 mT. Therefore, this characteristic suggests arranging two magnetic fields spatially such that their angle is close to 90°, minimizing the field intensity in transitional regions between them and effectively reducing interference with non-target control areas. Moreover, as depicted in Supplementary Fig. 91D, regardless of the magnetic field angle, the magnetic intensity drops significantly to a minimal value (approximately 20 mT) at distances greater than 60 mm from the Halbach array center. At distances exceeding 80 mm, the magnetic intensity approaches nearly zero and can thus be considered negligible. This distance can serve as a reference “safe distance” beyond which external regions remain unaffected by the Halbach arrays.

We examined the influence of varying magnetic field spacing L (distance between the two Halbach array end-faces) on the magnetic field across different regions. The magnetic field spacing L was increased incrementally, starting from 0 mm and increasing by increments of 10 mm, until the resulting magnetic field intensity approached approximately 0 mT. The diameter of the magnet distribution circles D for both Halbach arrays was fixed at 75 mm. The magnetic field plane angle α was set to 0°, indicating parallel end faces of the two Halbach arrays. The magnetic

field angle φ was tested at two values: 0° and 90°. Sampling points and a sampling line (along the z-axis) used in the analysis are shown in Supplementary Fig. 92A. As demonstrated in Supplementary Fig. 92B, simulation results indicate that when the magnetic field angle φ is 0°, magnetic field intensity at all points in the central region decreases with increasing magnetic field spacing L, reaching approximately 0 mT for all central points at an L of 120 mm. Regarding magnetic field intensity variation along the z-axis, as shown in Supplementary Fig. 92C, there is only a single peak at smaller magnetic field spacings (e.g., 0 mm or 10 mm), indicating the continuous presence of a magnetic field between the two Halbach arrays; the magnetic field intensity reduces to zero outside the arrays only at distances greater than 70 mm from the center. With increasing magnetic field spacing L, two peaks start to emerge, implying the appearance of a region with reduced magnetic intensity between the arrays. When magnetic field spacing L reaches 110 mm, a magnetic-field-free region appears in the central area. In contrast, as illustrated in Supplementary Fig. 92D, when the magnetic field angle φ is set at 90°, several points in the central region continuously exhibit a near-zero magnetic field intensity, regardless of the magnetic field spacing L. Correspondingly, Supplementary Fig. 92E shows that along the z-axis, all curves consistently exhibit two distinct peaks separated by valleys with magnetic intensities approaching zero (as indicated by the red boxes in Supplementary Fig. 92E), irrespective of changes in L. Therefore, when arranging magnetic fields, the magnetic field spacing L can be suitably increased within functional constraints to reduce interference in the central region. However, this reduction has limitations, and a more effective strategy involves setting the magnetic field angle φ to 90°. Similar to the earlier analysis of magnetic field angle φ on magnetic field, setting φ to 90° maintains the magnetic field intensity at nearly zero in the central region. This characteristic can thus serve as a critical parameter to minimize mutual interference between multiple magnetic fields.

Finally, we investigated the effects of varying the magnetic field plane angle α on the magnetic field in different regions. The diameter of the magnet distribution circles D for both Halbach arrays was set at 75 mm, the magnetic field angle φ was

fixed at 90°, and the magnetic field spacing L was fixed at 10 mm. The magnetic field plane angle α was varied from 0° to 150° in increments of 30°. Six sampling points (points D–I) and four sampling lines (z-axis, lines 1, 2, and 3) were selected, with their locations and parameters shown in Supplementary Figs. 93A-93B. As shown in Supplementary Fig. 93C, for sampling points located on the inner side (points G, H, I) and outer side (points D, E, F) of the Halbach arrays, the magnetic field intensity remains relatively high at smaller magnetic field plane angles α. However, when α exceeds approximately 60° or 90°, a sharp decrease in magnetic intensity occurs. The variation of magnetic intensity along the z-axis (Supplementary Fig. 93D) also follows this pattern, with the intensity profile transitioning from two distinct peaks to a single peak as α increases. Supplementary Fig. 94A presents the magnetic intensity variations along lines 1, 2, and 3. We observed that the magnetic intensity reaches its maximum near the Halbach array end-face plane (i.e., near the rotation origin of the upper Halbach array), due to the proximity of this location to the magnets in both arrays. On line 2, regardless of the changes in the magnetic field plane angle, magnetic intensities remain consistently low on both the inner and outer sides of the Halbach arrays and approach approximately zero beyond a short distance of about 35 mm. On lines 1 and 3, when the magnetic field plane angle α exceeds 90°, magnetic intensities on both sides of the arrays behave similarly to line 2. In contrast, when α is smaller than 90°, the magnetic field intensity is essentially zero on the outer side but remains high on the inner side of the arrays. These simulation results illustrate how different magnetic field plane angles α influence the magnetic field in various spatial regions. In practical applications, selecting a larger magnetic field plane angle α is advisable to reduce the magnetic field's interference in both inner and outer regions. Nevertheless, even at smaller magnetic field plane angles α, it is possible to identify specific regions with relatively low magnetic interference to accommodate particular application requirements.

In summary, we analyzed the effect of the diameter of the magnet distribution circle D on the magnetic field intensity for a single magnetic field. Additionally, we evaluated the influence of magnetic field angle φ, magnetic field spacing L, and

magnetic field plane angle α on the magnetic field intensity across various regions. Although our analysis is not exhaustive—only selected sampling points and sampling lines were studied, rather than all possible regions—the investigated areas are representative of typical application scenarios. Furthermore, the associated parameters and observed trends provided herein can serve as practical references for specific magnetic field configurations. These results are readily applicable to designs where high control precision is not strictly required. For scenarios demanding greater accuracy, more detailed simulation studies can be conducted following the methodologies and insights established in this analysis. Additionally, our simulations focused solely on the interference between two magnetic fields. Due to space constraints in this paper, we did not conduct a detailed analysis of interference among multiple magnetic fields. However, using the methodology outlined in this study, further simulations can be conducted as needed to investigate the interactions of multiple magnetic fields. The multi-magnetic field control method proposed in Supplementary Note 9 is not limited to the use of Halbach arrays; it can also be implemented using electromagnetic coils. Since the long-range operation with a large Magnetic Interaction Length setup in our study utilizes Halbach arrays, our simulations were conducted exclusively on this configuration. Nevertheless, the same approach can be applied to analyze the interference of magnetic fields generated by electromagnetic coils.

Supplementary Note 12:

Detailed Procedures and Discussion for Coordinated Multi-Instrument Operation Experiments

To conserve space in the main text, we present here the detailed procedures and discussion for the three experiments described in the “Coordinated Multi-Instrument Operation” section. The details are as follows:

To demonstrate the feasibility of this method, we constructed two types of integrated soft tubes, referred to as Tube A and Tube B (Supplementary Fig. 55). The inner tube was substituted with a soft rod to facilitate manufacturing and manipulation. We operated directed downward with a strength of 70 mT (Fig. 4b and Supplementary Video 10). To visually demonstrate the independent and controllable changes in the states of both tubes within the same magnetic field, we measured the variations in the radius of curvature and the terminal bending angles (definition in Supplementary Fig. 29) throughout the operation (Fig. 4c). Note that, in their straight state, the radius of curvature is infinite, and the terminal bending angle is 0°. The entire operation process was divided into six stages, outlined as follows: (1) Tube A was moved forward with a straight state under the magnetic field. At this stage, Tube A was non-magnetic because d_{R12} was set to 0, corresponding to 0-3s in Fig. 4b(i) and Zone A in Fig. 4c. (2) The relative positions of the two magnetic units within Tube A were altered by changing d_{R12}, which allowed Tube A to assume various states. This operation corresponds to 3s-10.5s in Fig. 4b(i) and Zone B. (3) While maintaining the state of Tube A, Tube B was advanced or retracted. At this time, Tube A maintained a specific state under the magnetic field with a certain d_{R12} value, while Tube B was in a straight state as a result of d_{R12}= 0, corresponding to 10.5s-13s in Fig. 4b(ii) and Zone C. (4) With Tube A’s state unchanged, the state of Tube B was adjusted by changing d_{R12}, corresponding to 13s-20.3s in Fig. 4b(ii) and Zone D. (5) Tube B was returned to a straight state (d_{R12}= 0) and retracted, corresponding to Zone E. (6) Tube A was returned to a straight state (d_{R12}= 0) and retracted, corresponding to Zone F. This

operation demonstrated the feasibility of manipulating multiple instruments under the same magnetic field. The magnetization profile settings for the instruments are relatively simple, resulting in straightforward deformations. More complex deformations or functionalities can be achieved by employing more complex magnetization designs, such as the helical transformations shown in Configuration G of the 1D magnetization reprogramming.

To further explore the potential application of this proposed method, we established three experimental scenarios based on typical application settings depicted in Fig. 4a. The experimental phantoms were fabricated using 3D printing (Form 3B+, Formlabs, USA) with Elastic 50A as the printing material. The instruments used in these operations are the same as those in Fig. 4b.

The first experimental scenario, as illustrated in Supplementary Fig. 56A, involves numerous tissues between the target and the entry site, which act as obstacles to diagnostic or therapeutic procedures. The target requires the coordinated action of two instruments: one instrument follows path A to the target to complete preliminary treatment tasks; subsequently, a second instrument follows path B to the target to administer adjunctive therapy. This process is shown in Supplementary Video 11. In this video, as the first soft tube traveled along path A, it initially maintained a straight state under a uniform magnetic field (90 mT, directed downwards) due to obstacles within the path. Upon reaching the target location, it transformed into a curved state under the same uniform magnetic field, enabling its tip to reach the target position. To maintain the shape of the first soft tube, the magnetic field was kept constant. Then, the second soft tube, operating under the same uniform magnetic field, traversed the obstacles in a straight state. Near the target location, the second instrument transitioned to a curved state to position its tip to the target. After completing all diagnostic and therapeutic procedures (not shown in the movie), these two soft tubes reverted to a straight state, allowing them to retract through the obstacles.

In the second experimental scenario, two targets were established (Supplementary Fig. 56B); these targets require sequential operations/treatments,

necessitating three instruments, which follow paths A, B, and C to reach targets A and B, respectively. After completing the tasks, the instruments will be retracted. The instruments used are the same as those depicted in Fig. 4b, with the first instrument configured as Tube A and the second and third instruments as Tube B (Supplementary Fig. 55B). Similar to the first experimental scenario, the entire operation took place under a 90 mT downward uniform magnetic field. The three instruments navigated through the obstacles in a straight state and then adjusted their shapes to position their tips to the targets. Finally, they retracted back through the obstacles in a straight state after completing the procedures. Details of the specific operations can be viewed in Supplementary Video 12.

In the third experimental scenario (Fig. 4d), a target was set up, requiring one instrument to perform auxiliary operations near position A along path A, and another instrument to proceed along path B to carry out diagnostic and therapeutic operations at the target location. However, path B is obstructed by barriers, necessitating an additional instrument to clear the pathway. The instruments used in this operation are the same as those in the second experimental scenario, and the entire procedure was conducted under a 90 mT downward uniform magnetic field. Fig. 4e and Supplementary Video 13 illustrate the entire procedure. Before 12.5s, the first soft tube passed through obstacles in a straight state along path A, then adjusted to a curved state to reach position A and maintained that shape. Between 12.5s and 14s, a secondary soft tube entered the obstacles in a straight state, then shifted to a curved state to hook onto the obstacle. Subsequently, the secondary soft tube retracted, pulling the obstacle with it, and maintained this state to clear path B. From 21s to 23s, the main soft tube traveled in a straight state along path B to near the target location, then changed form to reach the target. After completing the diagnostic and therapeutic operations (not shown in the movie), all soft tubes reverted to a straight state and retracted through the entry site.

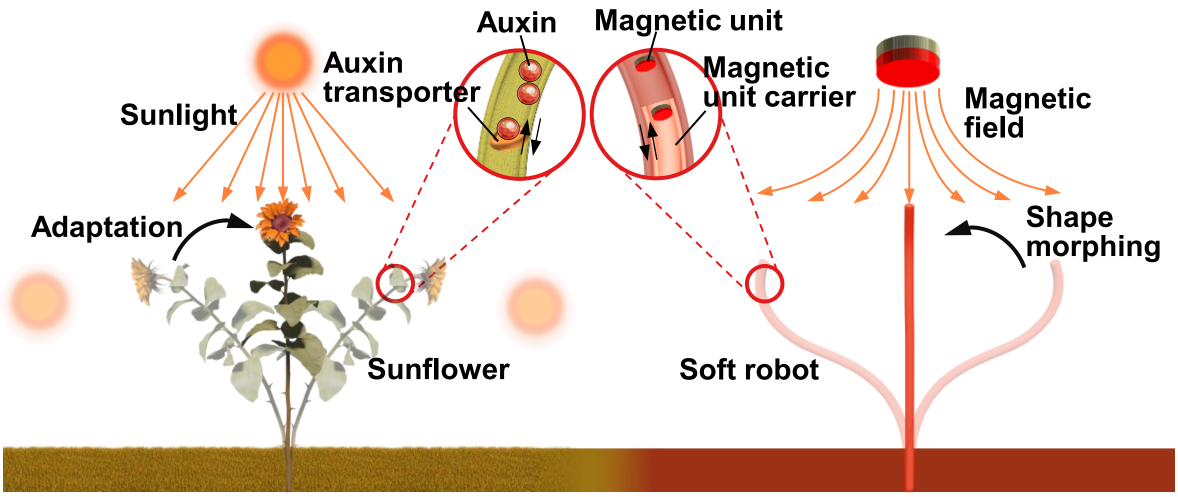

Supplementary Fig. 1. Sunflower-inspired real-time in-situ magnetization reprogramming of magnetic soft robots. Adaptive response of sunflowers mediated by internal auxin redistribution, inspiring a similar mechanism for real-time in-situ magnetization reprogramming. Both systems achieve differential deformation by redistributing their internal elements—auxin in sunflowers and magnetic units in our proposed method. Sunflowers induce auxin transporters in response to illumination to transport auxin, causing variations in auxin distribution, whereas the proposed method induces magnetic unit carriers according to operational requirements to move magnetic units, causing variations in magnetic unit position. Parallel to the auxin transporters in sunflowers, which are internally regulated by biochemical processes, the magnetic unit carriers in the proposed method are controlled by external actuation.

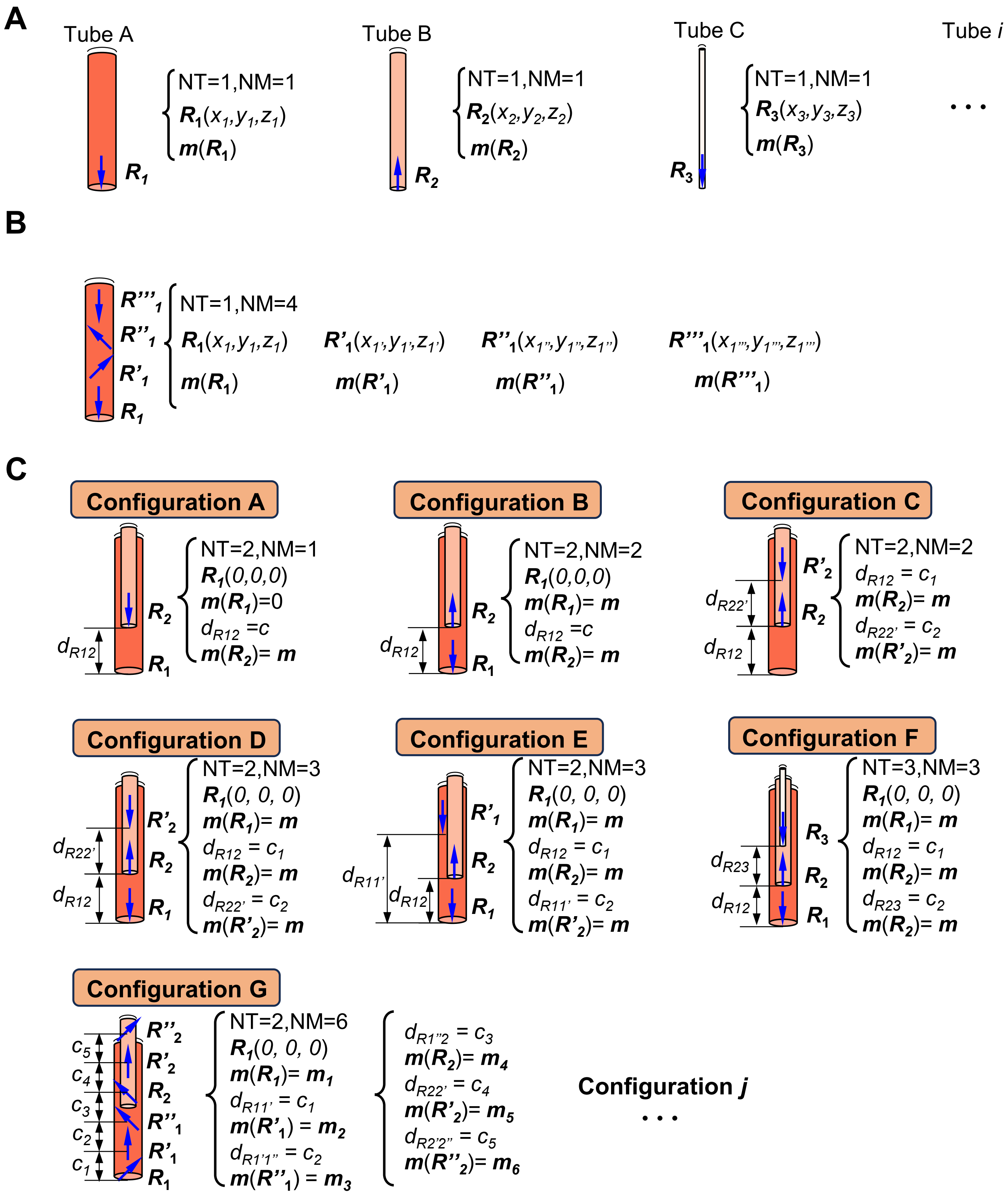

Supplementary Fig. 2. Detailed description and parameterization of one-dimensional (1D) magnetization reprogramming. (**A**) Tubes with varying magnetization profiles and different diameters that can be nested together. Each tube is characterized by parameters ***R***, ***m(R)***, NT, and NM. ***R*** denotes the position of the magnetic unit, ***m(R)*** represents the magnetic moment, NT indicates the number of tubes, and NM signifies the number of magnetic units. (**B**) Magnetization expansion of a single tube and its descriptive parameters. (**C**) Common configurations of 1D magnetization reprogramming. d_{Rij} indicates the distance between two corresponding magnetic units, and c_i represents a constant.

A

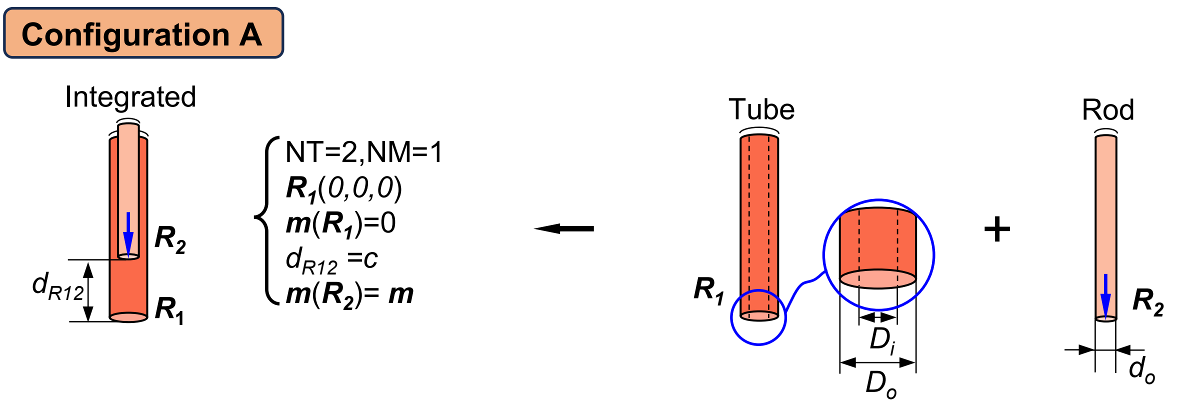

B

Symbol	Value
D_o	2.6 mm
D_i	1.7 mm
d_o	1.4 mm
m	1.02×10^{-3} Am^2
c, d_{R12}	0 mm, 14 mm, 28 mm, 42 mm

C

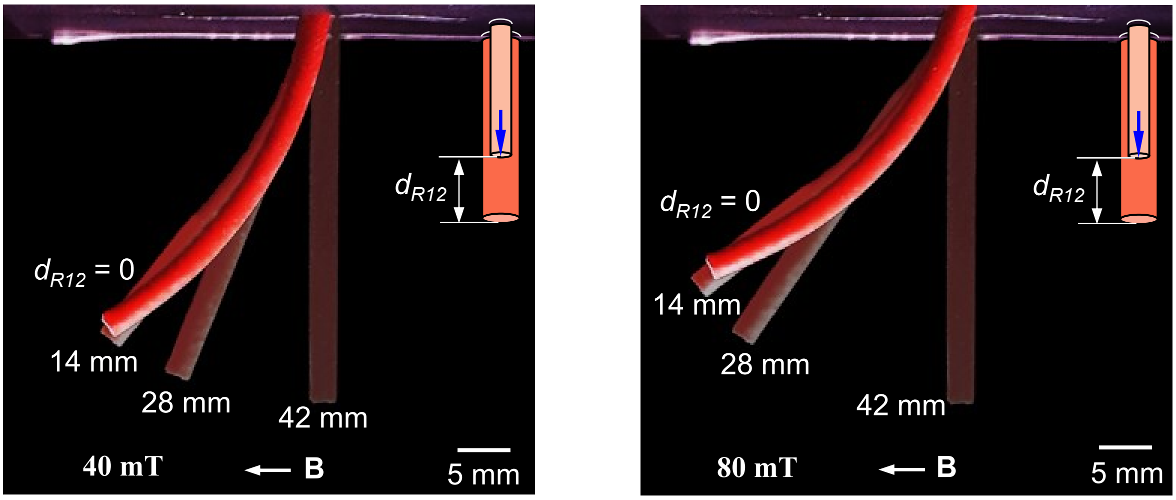

Supplementary Fig. 3. Detailed parameters and shape change characteristics of Configuration A in 1D magnetization reprogramming. (**A-B**) Parameters of Configuration A. (**C**) Deformations of the integrated tube with magnetic units at various positions (d_{R12}=0, 14 mm, 28 mm, 42 mm) under uniform magnetic fields of 40 mT and 80 mT. Deformations under the same magnetic field are consolidated into a single figure.

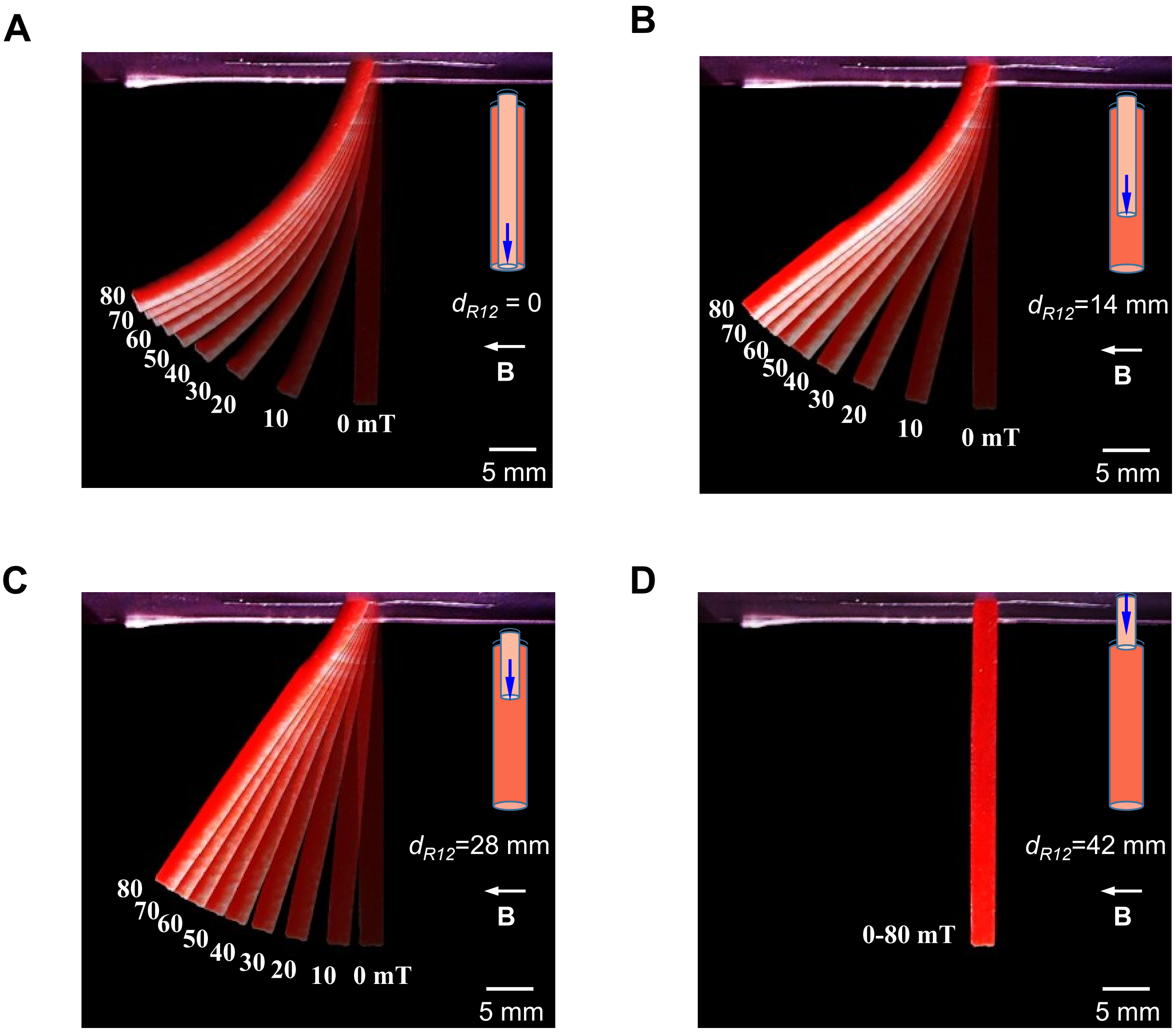

Supplementary Fig. 4. Shape change characteristics of Configuration A in 1D magnetization reprogramming. Deformations of the integrated tube with the same magnetic unit position are consolidated into a single figure, with each figure depicting the magnetic field incrementally increasing from 0 mT to 80 mT in 10 mT increments. These figures illustrate the various deformations with different magnetic unit positions under various magnetic field strengths. (**A**) d_{R12}=0. (**B**) d_{R12}=14 mm. (**C**) d_{R12}=28 mm. (**D**) d_{R12}=42 mm. In (D), the tube does not undergo deformation in response to changes in the magnetic field, as the magnetic unit is located outside the tube. This characteristic allows for selective control over whether the tube is influenced by the magnetic field.

A

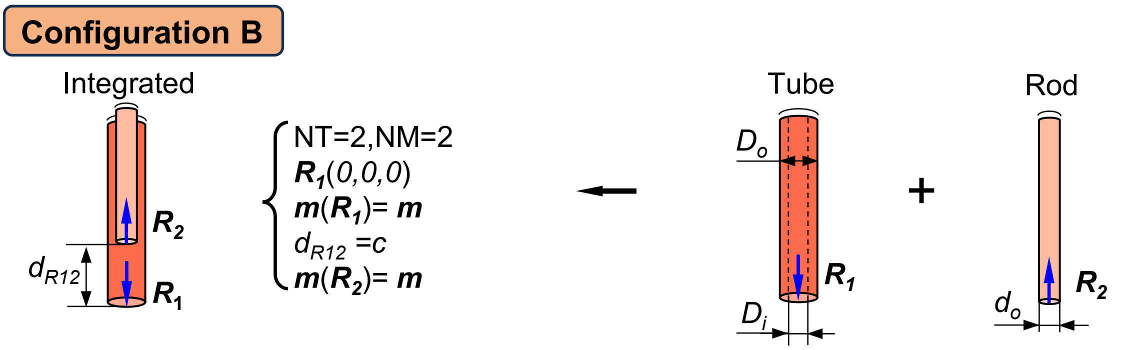

B

Symbol	Value
D_o	2.6 mm
D_i	1.7 mm
d_o	1.4 mm
m	1.02×10^{-3} Am^2
c, d_{R12}	0 mm, 6mm, 12 mm, 18 mm, 24 mm

Supplementary Fig. 5. Detailed parameters of Configuration B in 1D magnetization reprogramming.

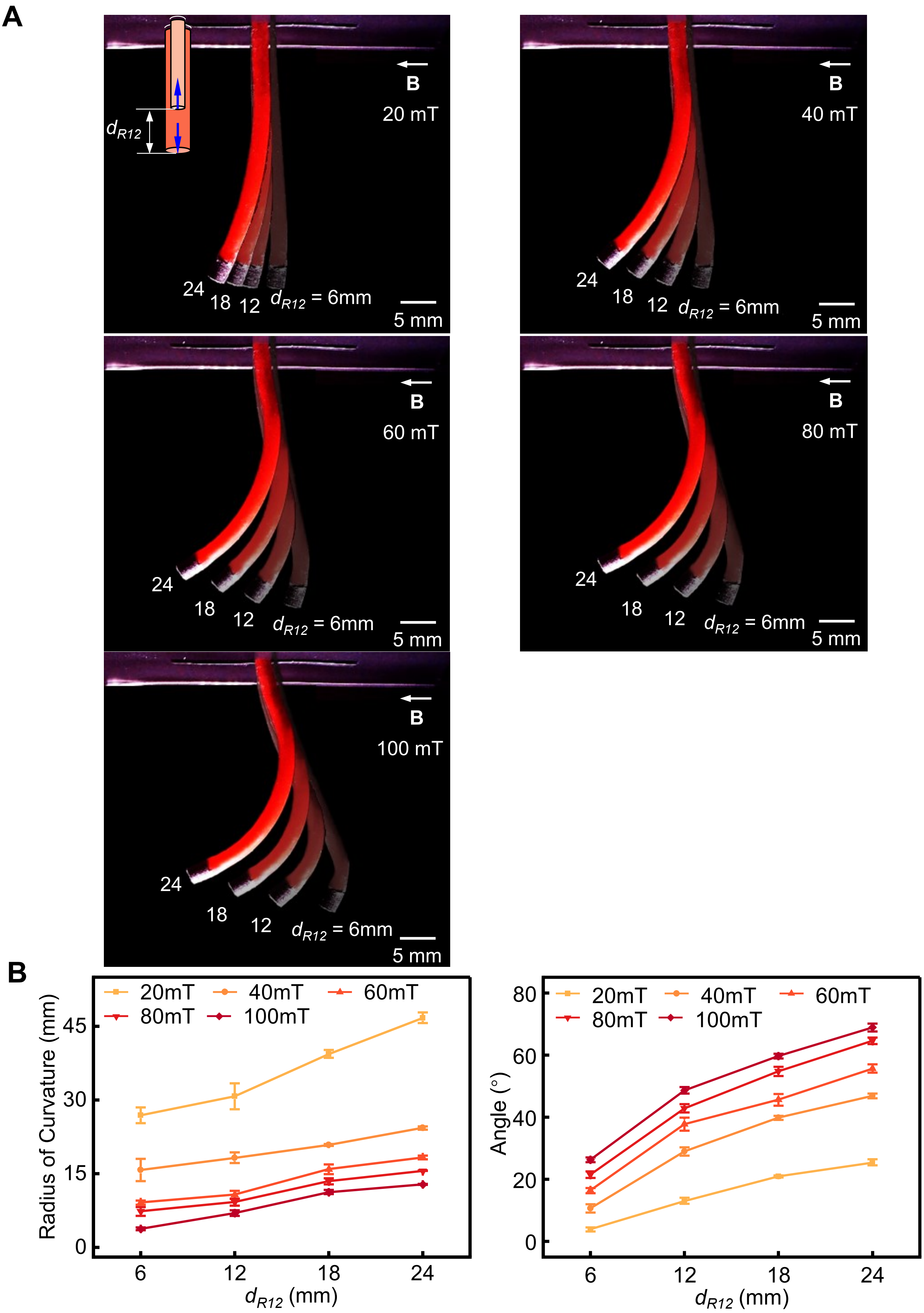

Supplementary Fig. 6. Shape change characteristics of Configuration B in 1D magnetization reprogramming. (**A**) Deformations of the integrated tube with magnetic units at various positions (d_{R12}=6 mm, 12 mm, 18 mm, 24 mm) are consolidated into a single figure. Each figure depicts the deformation under the same uniform magnetic fields, and the magnetic field strength in these five figures incrementally increases from 20 mT to 100 mT in 20 mT increments. (**B**) The shape changes of the integrated tube, characterized by two representative descriptors (radius of curvature and terminal bending angle, Supplementary Fig. 29), are depicted in two figures. Data are presented as mean ± SD (n=3 tests).

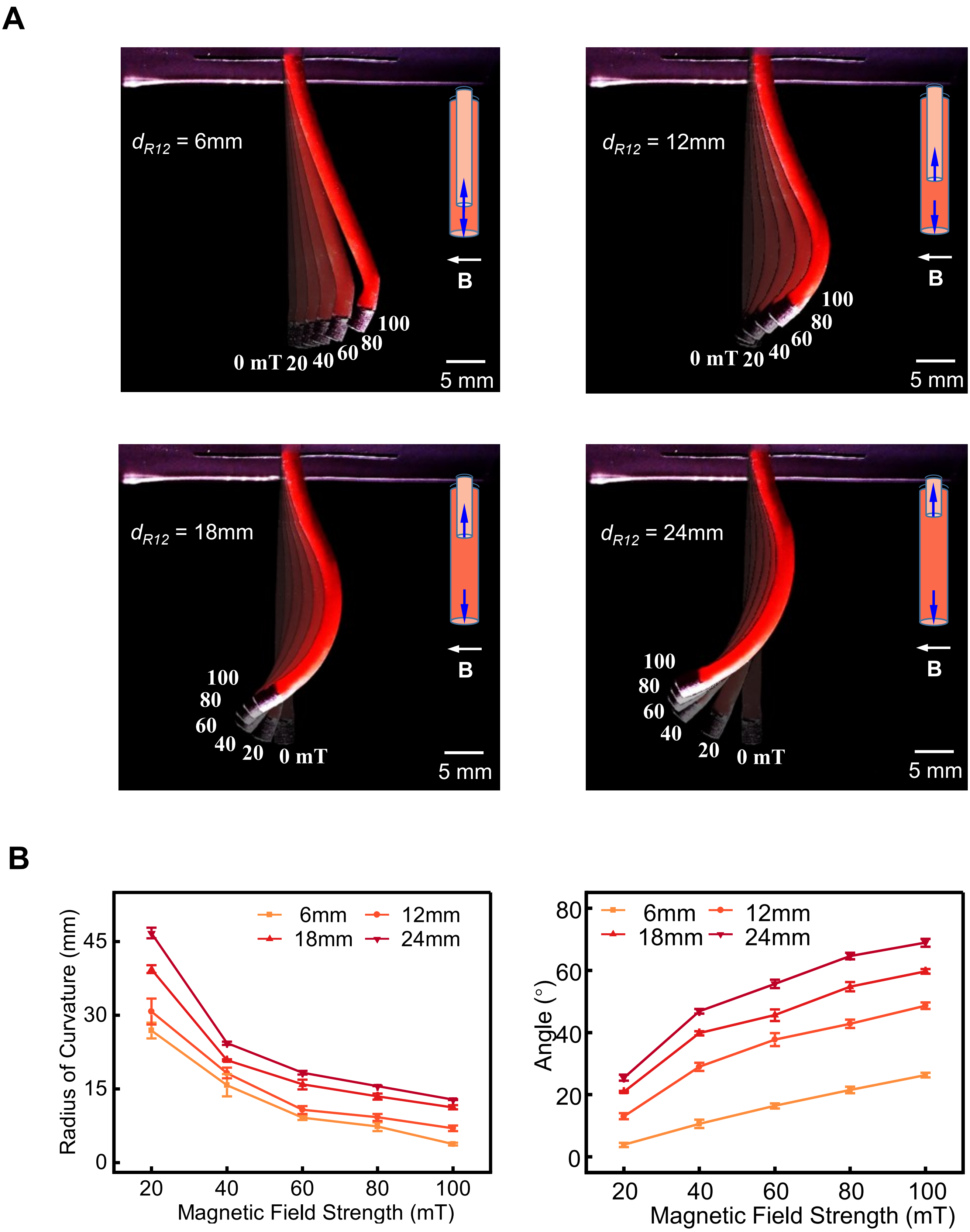

Supplementary Fig. 7. Shape change characteristics of Configuration B in 1D magnetization reprogramming. (**A**) Deformations of the integrated tube with the same magnetic unit position under various magnetic fields are consolidated into a single figure. Each figure depicts the deformation under the magnetic fields incrementally increasing from 0 mT to 100 mT in 20 mT increments. The magnetic positions in these four figures are 6 mm, 12 mm, 18 mm, and 24 mm, respectively. (**B**) The shape changes of the integrated tube are characterized by two representative descriptors (radius of curvature and terminal bending angle, Supplementary Fig. 29), and the magnetic field strength serves as the independent variable on the *x*-axis. Data are presented as mean ± SD (*n*=3 tests).

A

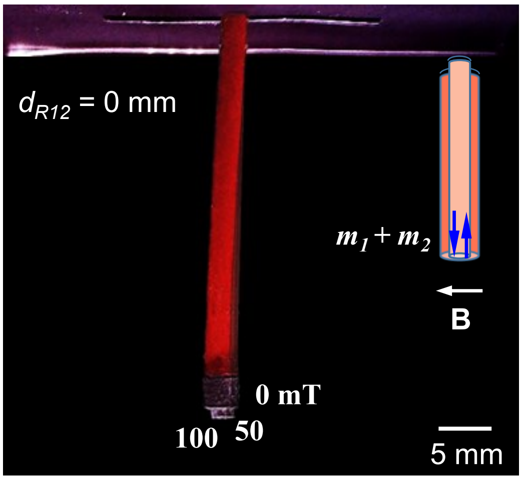

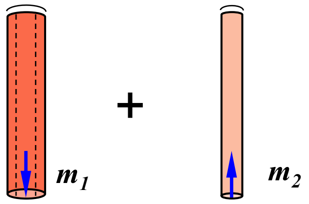

Symbol	Value
m_1	1.02×10^{-3} Am^2
m_2	1.02×10^{-3} Am^2

B

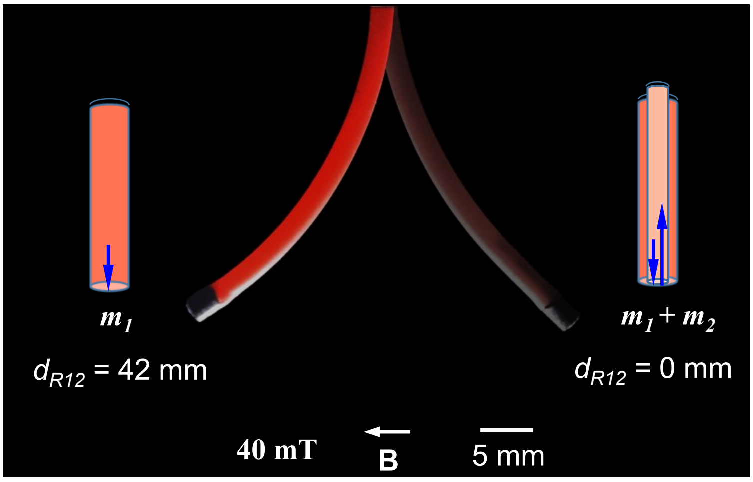

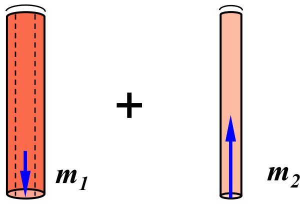

Symbol	Value
m_1	1.02×10^{-3} Am^2
m_2	2.04×10^{-3} Am^2

Supplementary Fig. 8. Representative features of configuration B in 1D magnetization reprogramming. (A) Magnetic neutralization: An integrated tube composed of two magnetic units with equal magnetization magnitudes but opposite directions maintains nearly the same position under varying magnetic fields, which incrementally increase from 0 mT to 100 mT in 50 mT steps. This behavior demonstrates its characteristic of magnetic neutralization. **(B)** Magnetic reversal: An integrated tube, consisting of two magnetic units with one unit having twice the magnetization of the other, both oriented in opposite directions, achieves a total reversal in deformation by adjusting the positions of the magnetic units when subjected to a uniform magnetic field of 40 mT. This behavior exemplifies the characteristic of magnetic reversal.

A

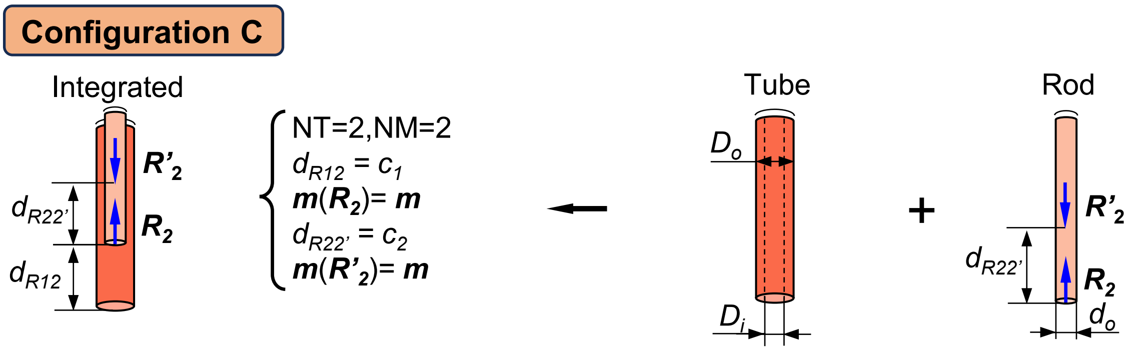

B

Symbol	Value
D_o	2.6 mm
D_i	1.7 mm
d_o	1.4 mm
m	1.02×10^{-3} Am^2
c_1, d_{R12}	0 mm, 5 mm, 10 mm, 15 mm, 20 mm
c_2, $d_{R22'}$	5 mm, 10 mm, 15 mm

Supplementary Fig. 9. Detailed parameters of Configuration C in 1D magnetization reprogramming. In this configuration, the location of the magnetic units is determined by two variables (d_{R12} and $d_{R22'}$), each of which can be set to various values, thus presenting numerous combinational possibilities.

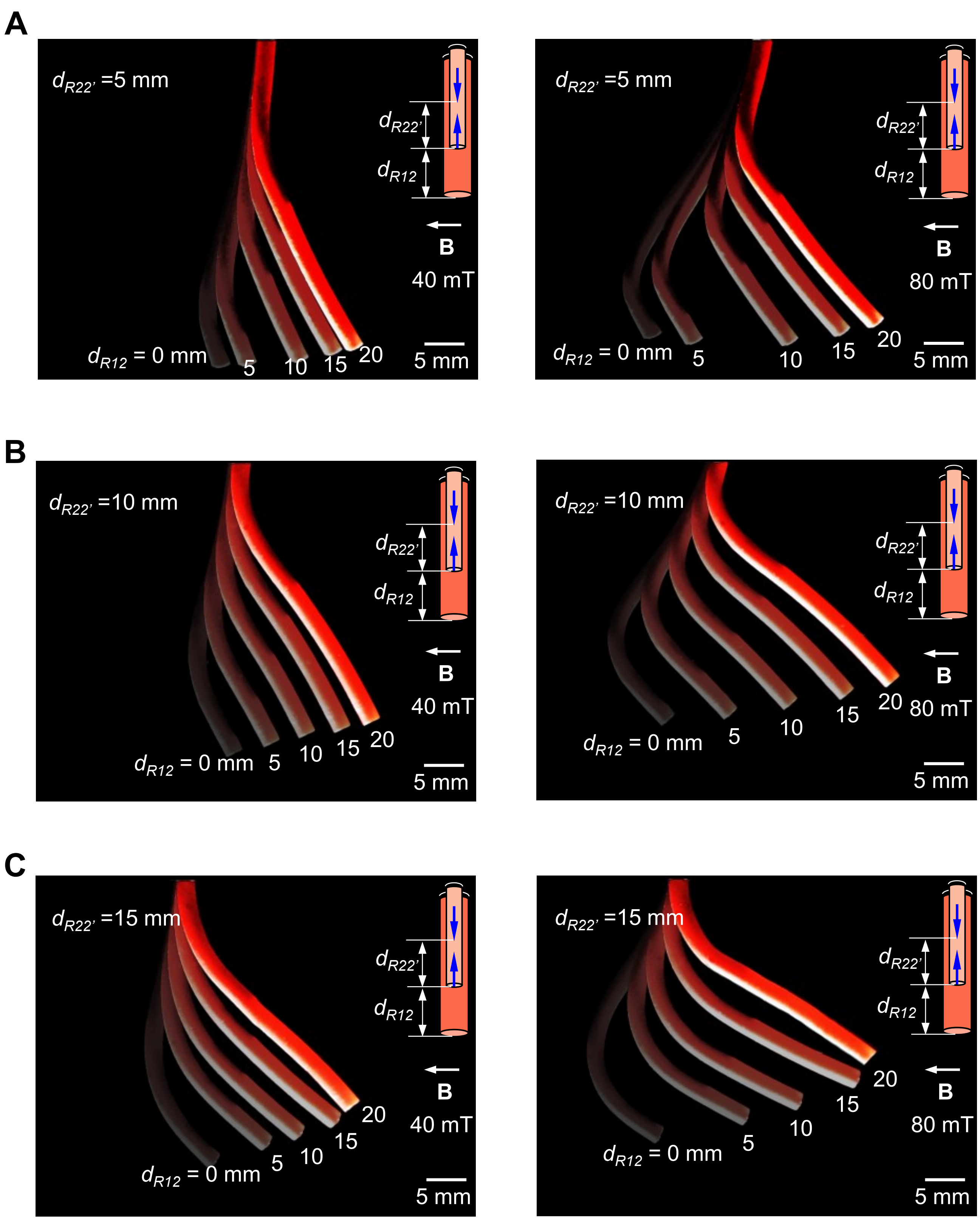

Supplementary Fig. 10. Shape change characteristics of Configuration C in 1D magnetization reprogramming. Parameter $d_{R22'}$ is set to three distinct values, and under each value of $d_{R22'}$, the value of d_{R12} is incrementally increased from 0 to 20 mm in increments of 5 mm to observe the deformation of the integrated tube. Additionally, magnetic field strengths of 40 mT and 80 mT are established as varying conditions. For the same value of $d_{R22'}$ and uniform magnetic field strength, deformations corresponding to five different values of d_{R12} are integrated into a single figure for ease of observation. (**A**) When $d_{R22'}$ is set to 5 mm and d_{R12} assumes various values, the resultant deformations under magnetic fields of 40 mT and 80 mT are observed. (**B**) When $d_{R22'}$ is assigned a value of 10 mm and d_{R12} varies, the corresponding deformations at magnetic field strengths of 40 mT and 80 mT are observed. (**C**) When $d_{R22'}$ is set to 15 mm and d_{R12} assumes various values, the resultant deformations under magnetic fields of 40 mT and 80 mT are obtained.

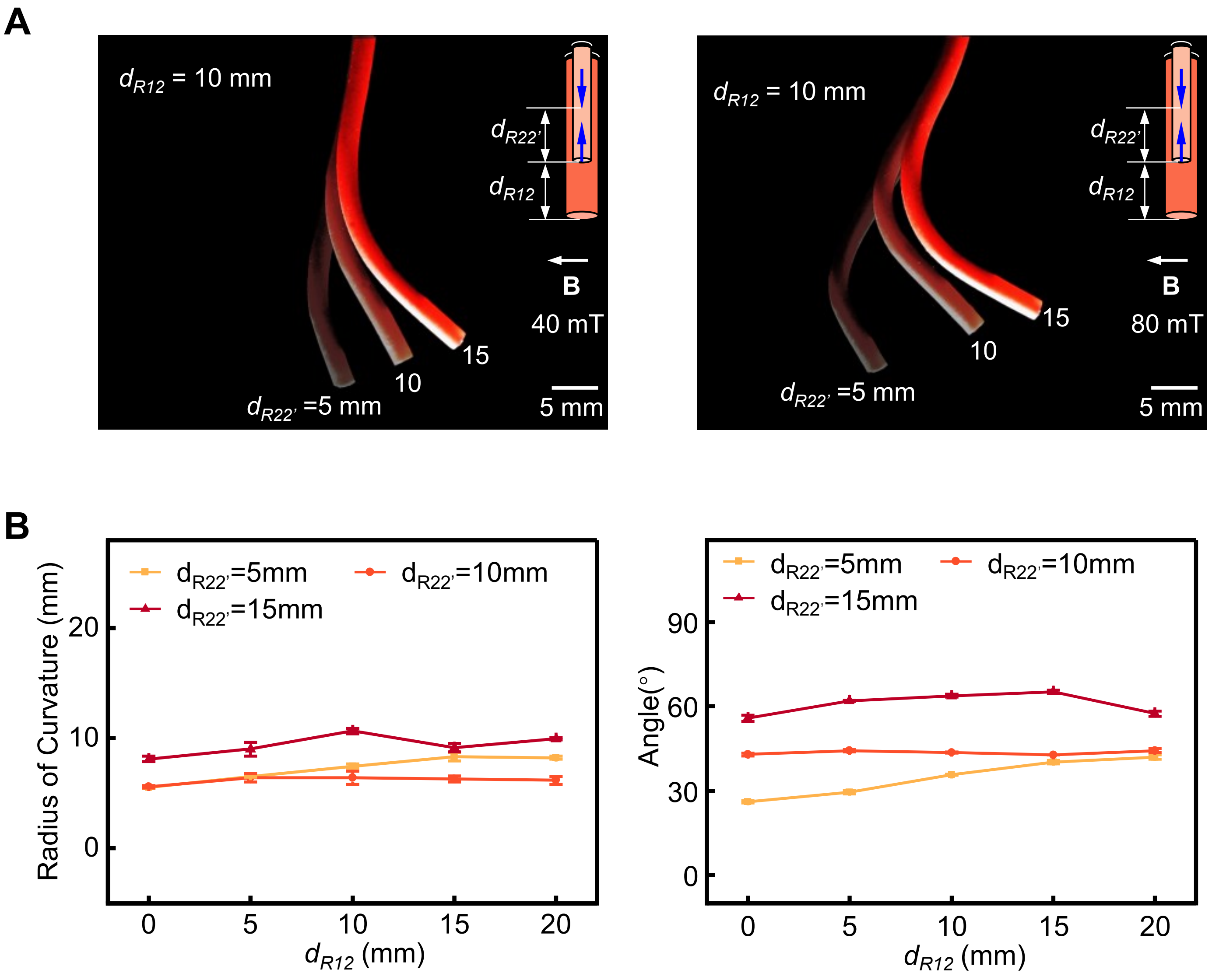

Supplementary Fig. 11. Quantitative description of the deformations generated by Configuration C in 1D magnetization reprogramming. (**A**) Deformation under 40mT and 80mT magnetic fields with d_{R12}=10mm and $d_{R22'}$ =5 mm, 10 mm, 15 mm, 20 mm. The deformations under the same magnetic field conditions are merged into a single figure for easier observation. (**B**) Radius of curvature and terminal bending angle of the integrated tube with different parameters under an 80 mT magnetic field. The trend lines in these two figures indicate that the radius of curvature and terminal bending angle change with variations in $d_{R22'}$ while showing no significant correlation with changes in d_{R12}. Please refer to Supplementary Fig. 29 for the definition of the radius of curvature and terminal bending angle. Data are presented as mean ± SD (n=3 tests).

A

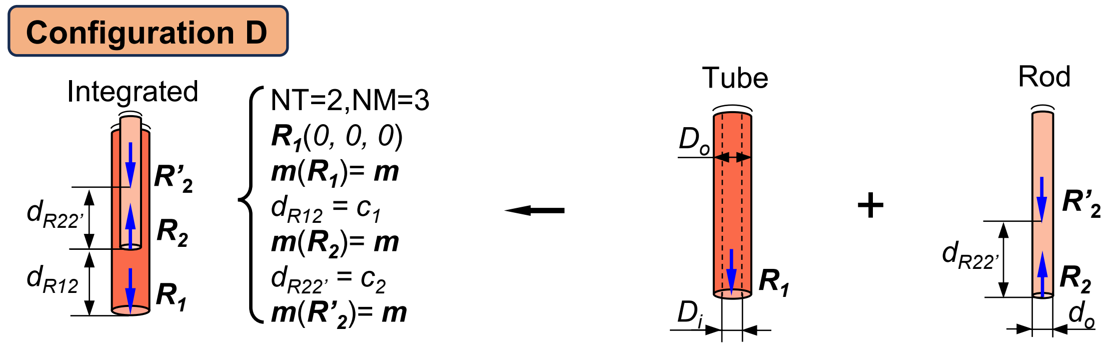

B

Symbol	Value
D_o	2.6 mm
D_i	1.7 mm
d_o	1.4 mm
m	1.02×10^{-3} Am^2
c_1, d_{R12}	0 mm, 4 mm, 8 mm, 12 mm, 16 mm
c_2, $d_{R22'}$	10 mm, 15 mm, 20 mm

C

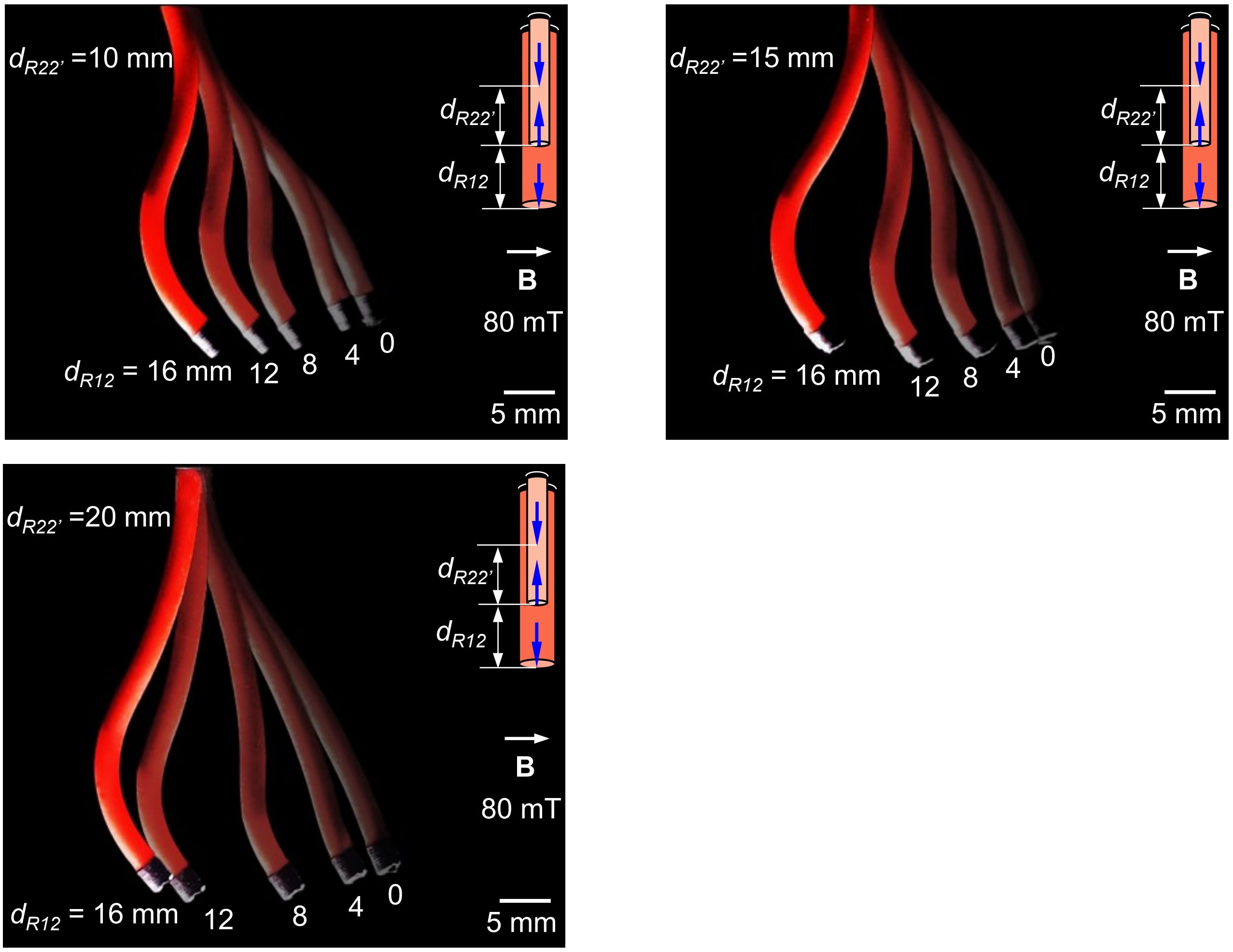

Supplementary Fig. 12. Detailed parameters and shape change characteristics of Configuration D in 1D magnetization reprogramming. (**A**-**B**) Parameters of Configuration D. (**C**) Parameter $d_{R22'}$ is set to three distinct values (10 mm, 15 mm, and 20 mm), and under each value of $d_{R22'}$, the value of d_{R12} is incrementally increased from 0 to 16 mm in increments of 4 mm to observe the deformation of the integrated tube. The magnetic field strength is 80 mT. For the same value of $d_{R22'}$, deformations corresponding to five different values of d_{R12} are integrated into a single figure for ease of observation.

A

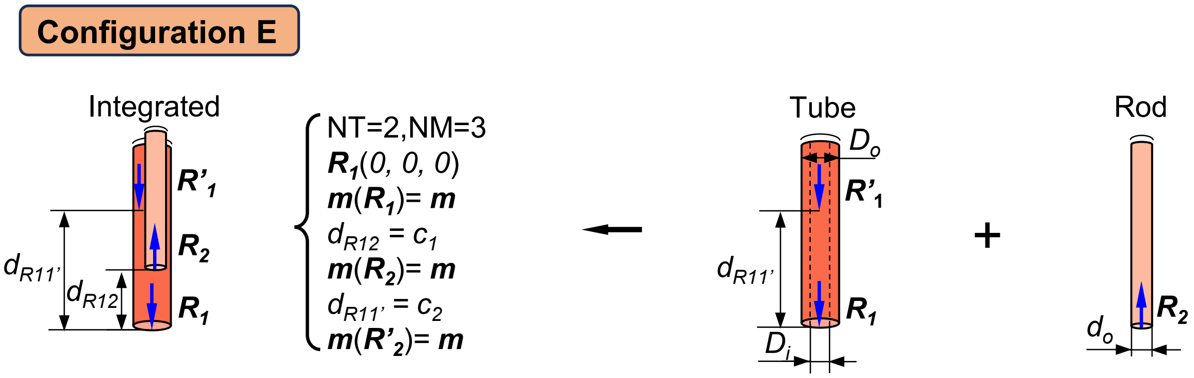

B

Symbol	Value
D_o	2.6 mm
D_i	1.7 mm
d_o	1.4 mm
m	1.02×10^{-3} Am^2
c_1, d_{R12}	0 mm, 9 mm, 18 mm, 27 mm, 36 mm
c_2, $d_{R11'}$	36 mm

C

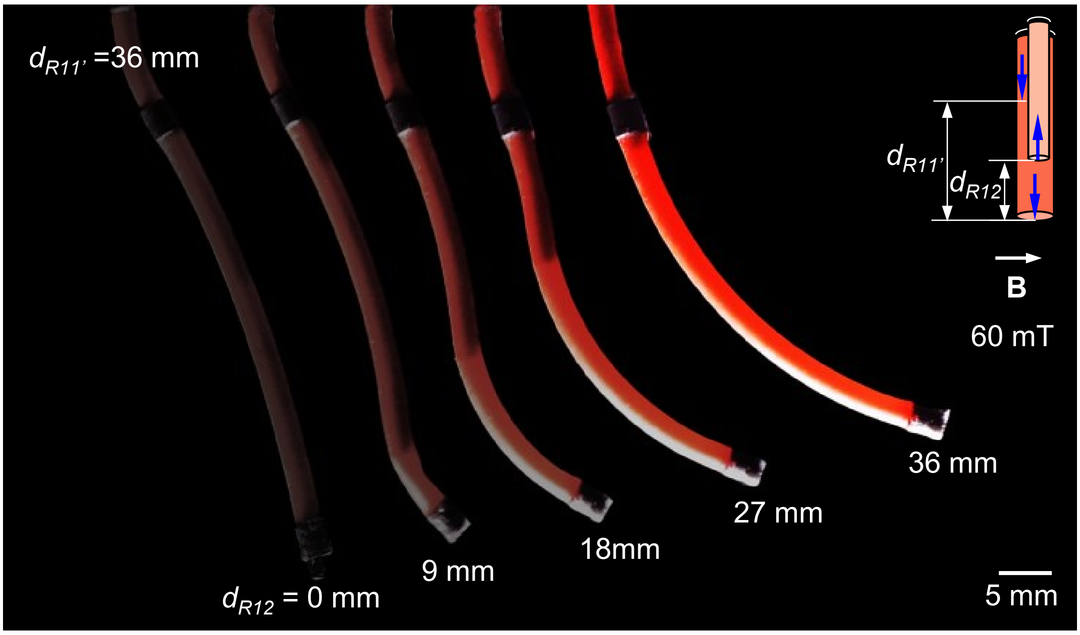

Supplementary Fig. 13. Detailed parameters and shape change characteristics of Configuration E in 1D magnetization reprogramming. (**A-B**) Parameters of Configuration E. (**C**) Parameter $d_{R11'}$ is set to 36 mm, and d_{R12} is incrementally increased from 0 to 36 mm in increments of 9 mm to observe the deformation of the integrated tube. The magnetic field strength is 60 mT.

A

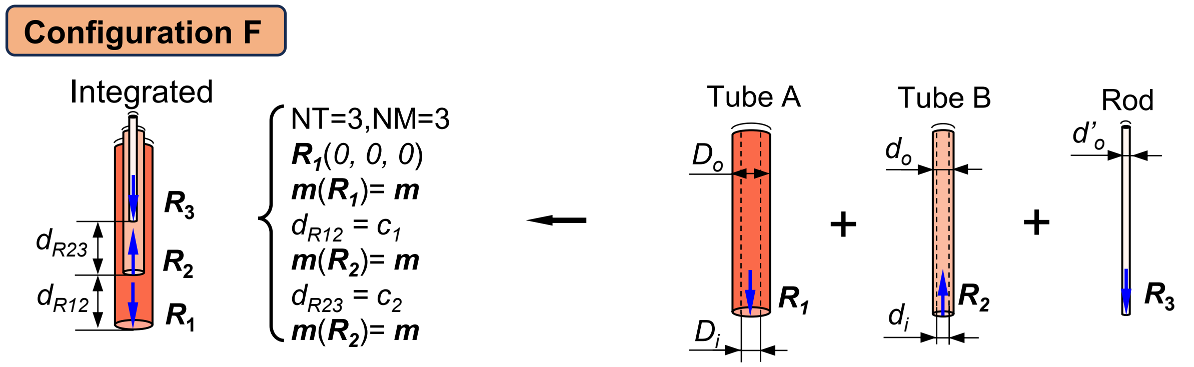

B

Symbol	Value
D_o	2.2 mm
D_i	1.7 mm
d_o	1.5 mm
d_i	1.1 mm
d'_o	0.8 mm
m	4.92×10^{-4} Am^2
c_1, d_{R12}	0 mm, 13 mm, 26mm
c_2, d_{R23}	13 mm, 26mm

C

State	Tubes	Conditions	Magnetic field
A	Tube A + Tube B	d_{R12} = 0	70 mT
B	Tube A + Tube B	d_{R12} = 13 mm	70 mT
C	Tube A + Tube B	d_{R12} = 26 mm	70 mT
D	Tube A + Tube B + Rod	d_{R12} = 13 mm, d_{R23}= 13 mm	70 mT
E	Tube A + Tube B + Rod	d_{R12} = 13 mm, d_{R23}= 26 mm	70 mT

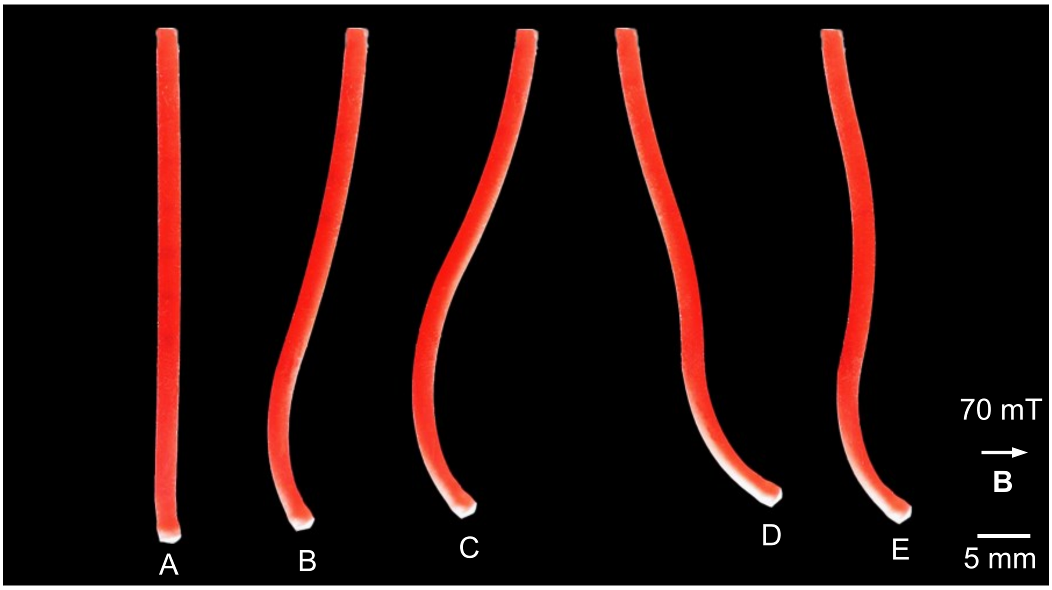

Supplementary Fig. 14. Detailed parameters and shape change characteristics of Configuration F in 1D magnetization reprogramming. (**A-B**) Parameters of Configuration F. (**C**) Owing to the increased diversity in combinations when three tubes are integrated, five common combinations are enumerated in the table. The deformations corresponding to these five combinations were tested under a magnetic field of 70 mT and are illustrated in the figure below.

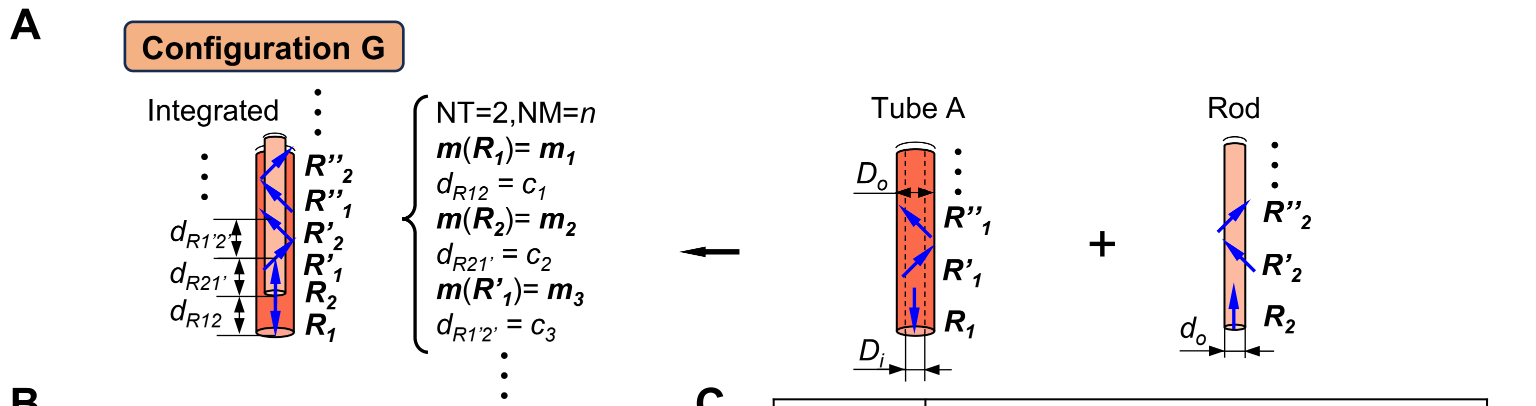

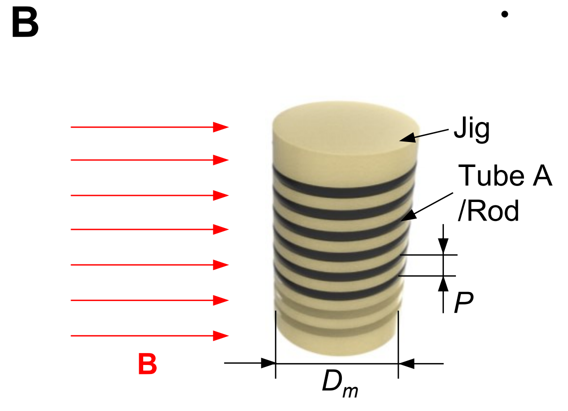

C

Symbol	Value
D_o	1.9 mm
D_i	1.1 mm
d_o	0.7 mm
D_m	12 mm
P	0.3mm
M_{tube}	25894 A/m
M_{rod}	135046 A/m
c_1, d_{R12}	0mm, 5mm, 10mm, 15mm, 20mm

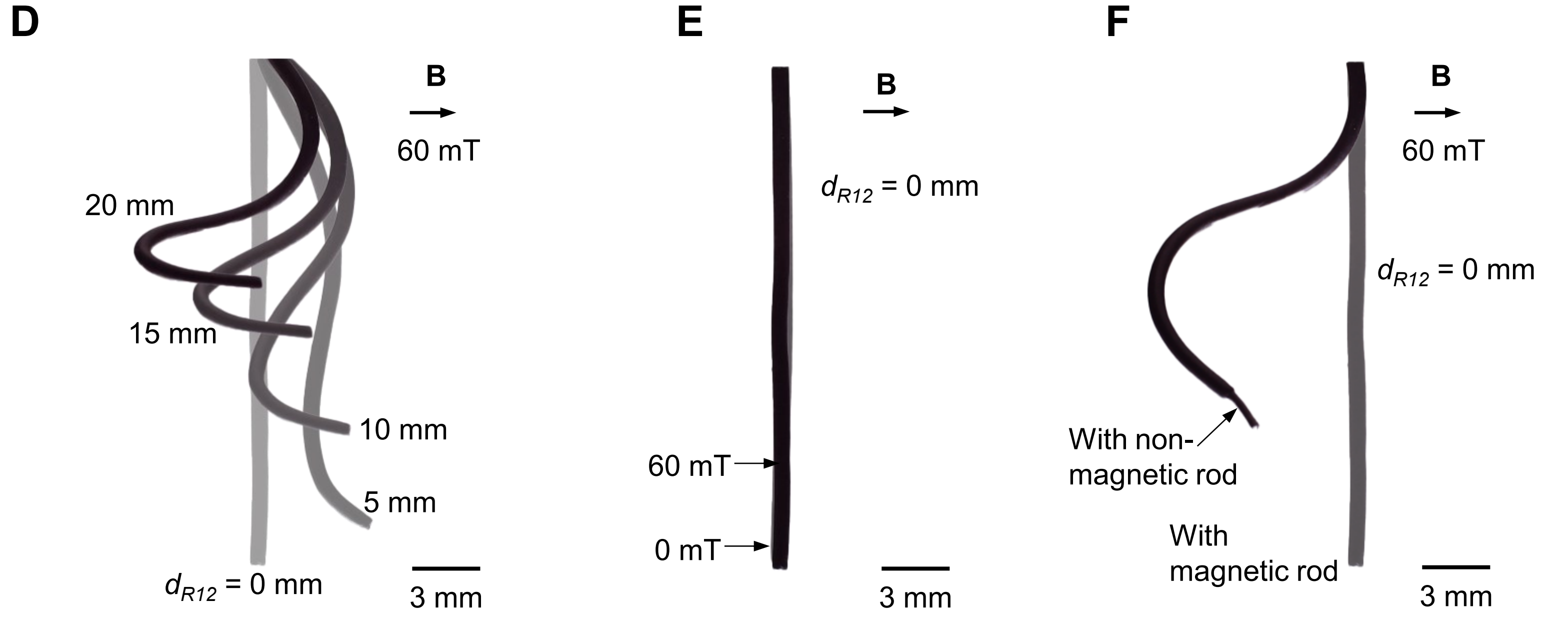

Supplementary Fig. 15. Detailed parameters and shape change characteristics of Configuration G in 1D magnetization reprogramming. (**A**, **B,** and **C**) Details and parameters of Configuration G. In (A), the blue arrows no longer refer to individual magnetic units, but rather indicate that the tube/rod encompasses numerous magnetization directions. The number of magnetic units in this configuration equals the number of magnetic powder particles, which is effectively infinite. In (B), to obtain the helical magnetization profile, the tube and rod were coiled around a jig for magnetization, and they exhibited completely opposite magnetization profiles. (**C**) Design parameters for the tube/rod and the jig used for magnetization. (**D**) Deformation of the integrated tube under various d_{R12}, demonstrating the capability of the integrated tube to switch between linear and helical deformations under the same magnetic field (60 mT). When d_{R12}=0, due to the combination of the rod and tube, magnetic neutralization is achieved, resulting in negligible deformation of the integrated tube in a 60 mT magnetic field. (**E**) To further validate the magnetic neutralization characteristic, a comparative experiment was designed. By applying magnetic fields of 0 mT and 60 mT, it was observed that the integrated tube maintained a nearly linear state in both conditions, with extremely minor differences between them. (**F**) To confirm that this state in (E) was not due to changes in stiffness caused by the inclusion of the internal rod, a control experiment was conducted. An integrated tube containing a magnetic rod remains linear under a magnetic field, whereas one with a non-magnetic rod assumes a helical shape. It was found that the stiffness induced by the rod does not cause the integrated tube to remain linear under magnetic fields. See Supplementary Video 1.

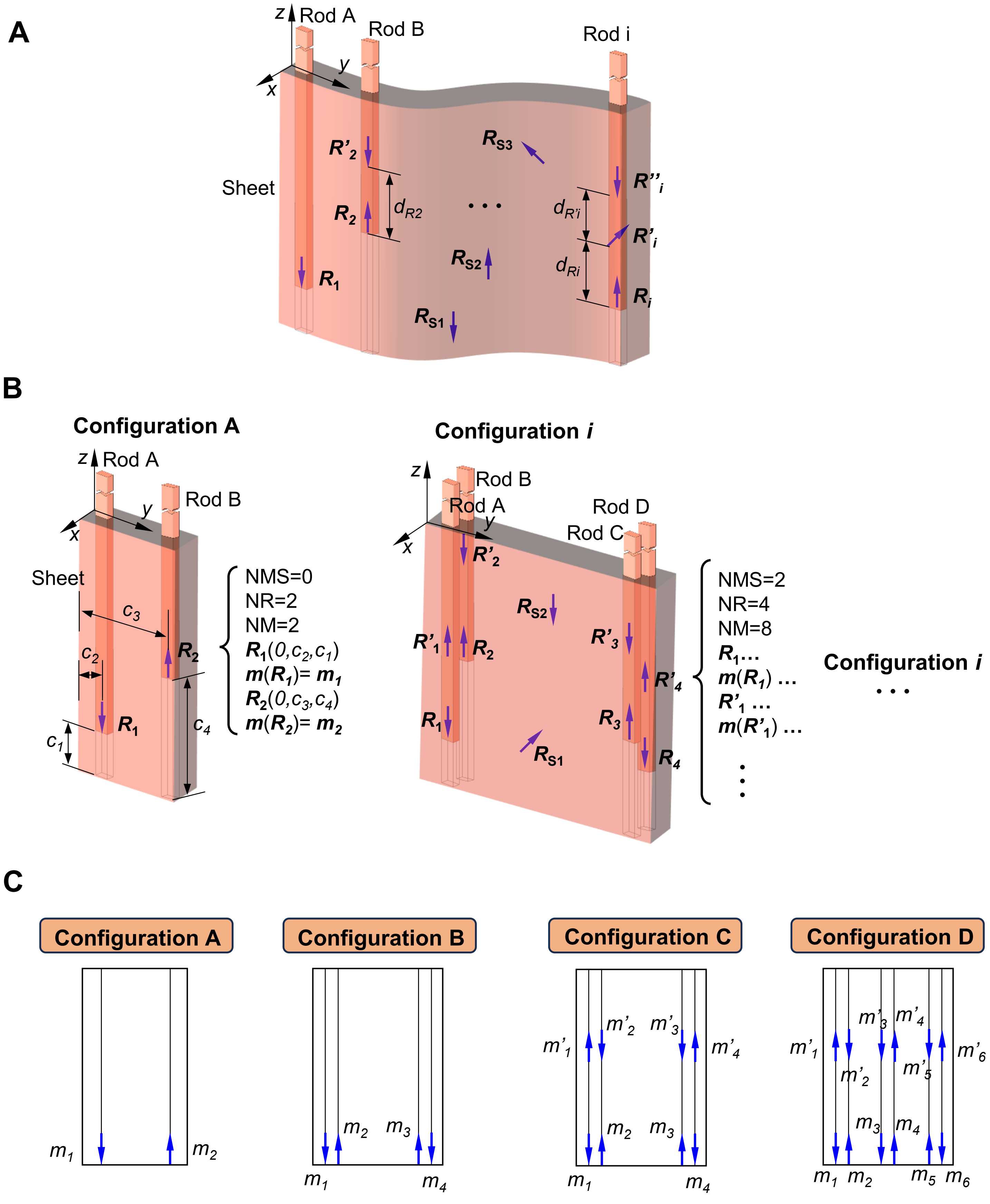

Supplementary Fig. 16. Detailed description and parameterization of two-dimensional (2D) magnetization reprogramming. (**A**) In the sheet, multiple square or round holes can be designed for the rod to pass through. The necessary magnetic units can be mounted to the rod. By adjusting the position of these rods relative to the sheet, changes in the magnetization on the sheet can be achieved. $\boldsymbol{R_i}$ denotes the position of the magnetic unit in the rod, $\boldsymbol{R_{si}}$ denotes the position of the magnetic unit in the sheet, and d_{Ri} indicates the distance between two corresponding magnetic units. (**B**) Common configurations of 2D magnetization reprogramming. NMS signifies the number of magnetic units in the sheet, NR indicates the number of rods, NM means the number of magnetic units in the rod, and c_i represents a constant. (**C**) Four simple configurations were selected and tested; the specific results are presented in the subsequent figures.

A

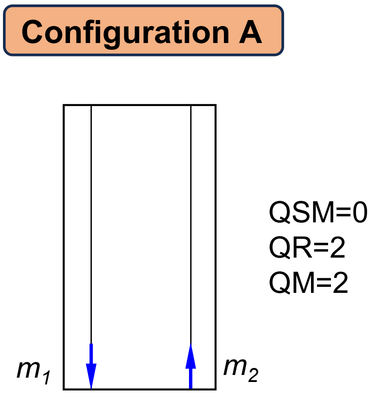

C

Symbol	Value
H	60 mm
W	3 mm
B_0	25 mm
B_1	20.7 mm
B_2	16.4 mm
C_1	1.5 mm
C_2	1 mm
a_0	1.5 mm
b_0	2 mm
L	70 mm
a_1	1.3 mm
b_1	1.8 mm

B

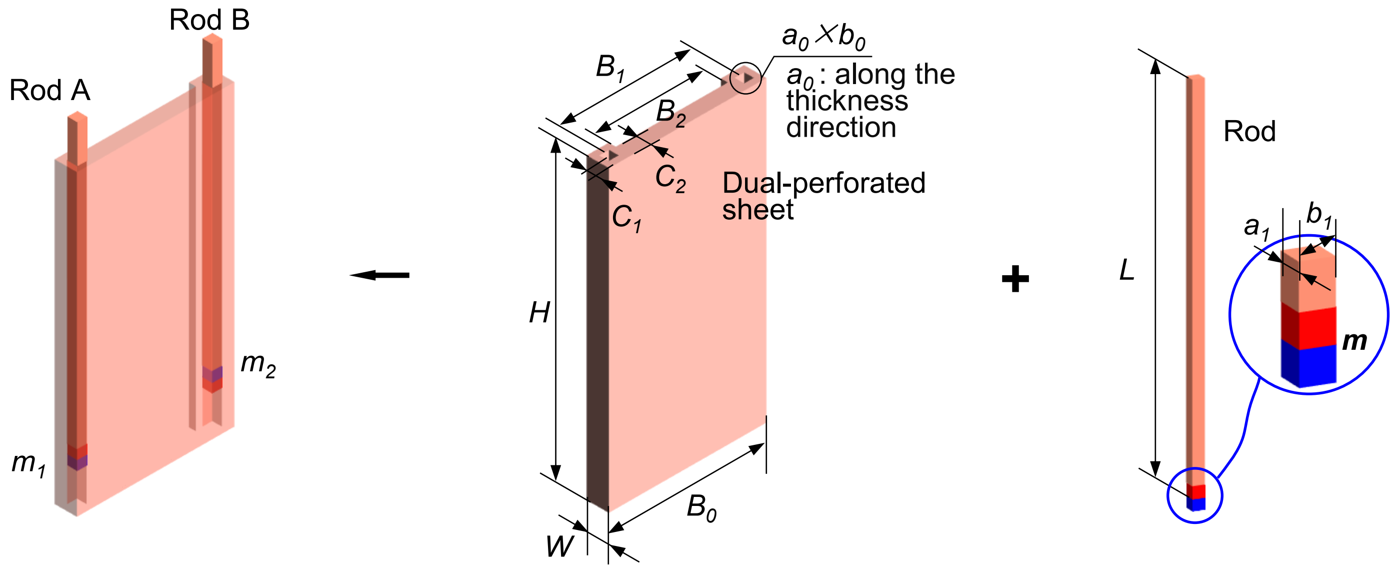

Supplementary Fig. 17. Detailed parameters for Configuration A of 2D magnetization reprogramming in Supplementary Fig. 16.

A

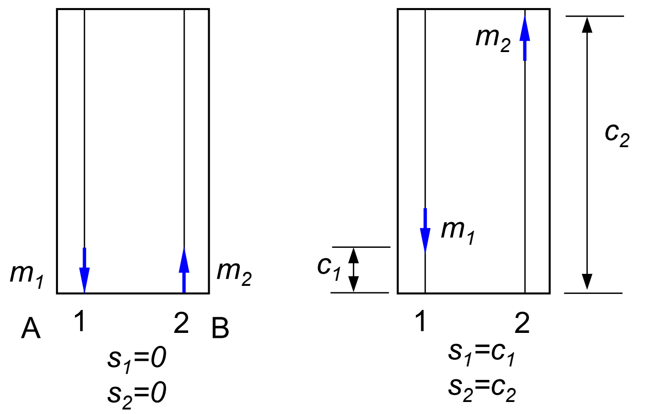

B

Symbol	Value
m_1, m_2	2.21×10^{-3} Am^2
c_1	0 mm, 36 mm
c_2	0 mm, 36 mm

C

Symbol	Value
m_1, m_2	2.21×10^{-3} Am^2
c_1	0 mm, 9 mm, 18 mm, 36 mm
c_2	0 mm

D

E

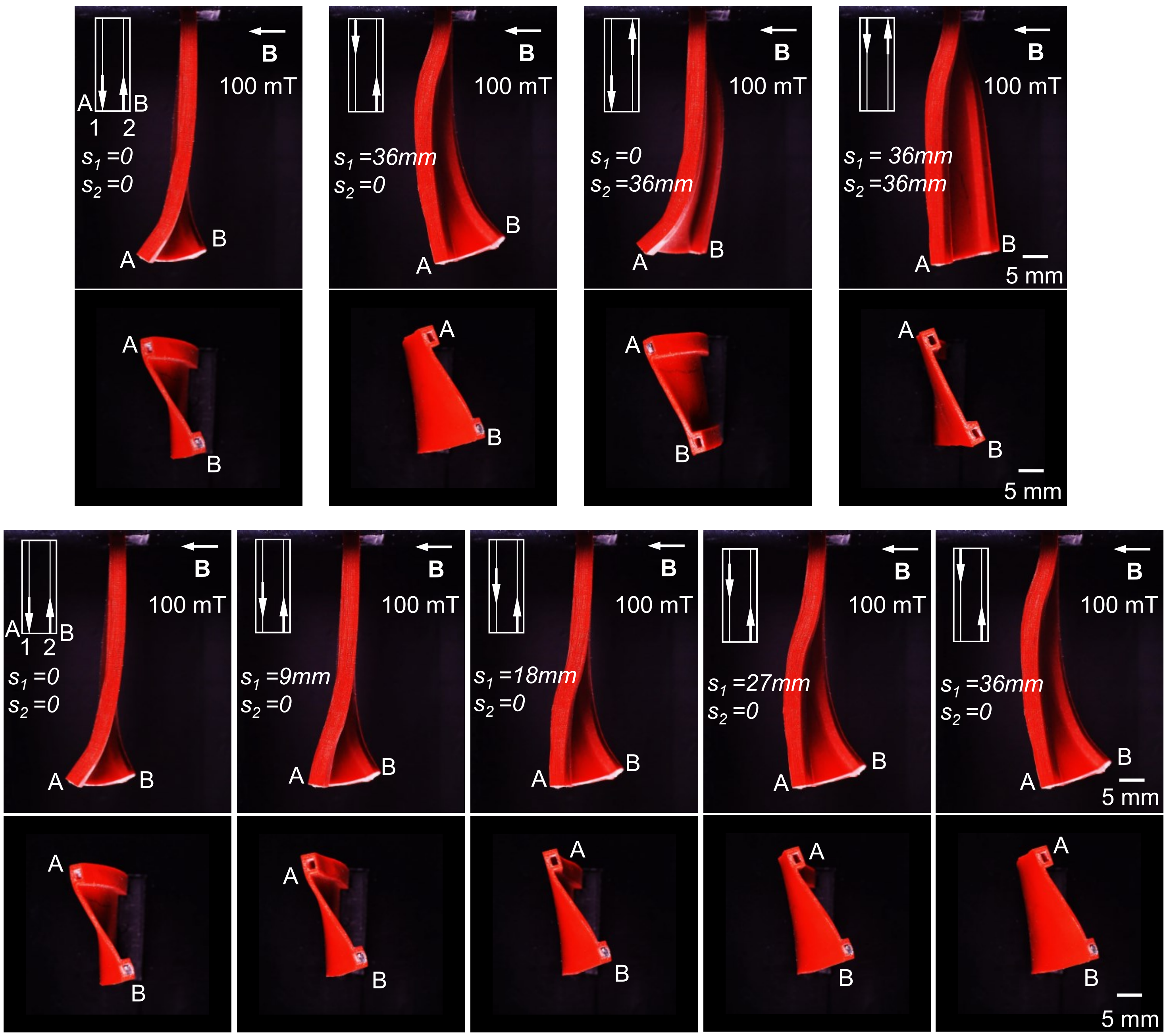

Supplementary Fig. 18. Shape change characteristics of Configuration A in 2D magnetization reprogramming. (**A**) Schematic illustration depicting the positions of the magnetic units. To describe the correspondence between the front and bottom views in the deformation of the sheet, the two lower corners of the sheet are denoted as points A and B. (**B**) Parameter settings for observing deformation at extreme positions: two extreme positions (0 mm and 36 mm) have been selected for each magnetic unit. (**C**) Parameter settings for observing deformation, aimed at controlling the variables: one magnetic unit is fixed (0 mm), while the other is positioned variably. (**D**) Deformations at these extreme positions in (B) with the front view in the first row and the bottom view in the second row. (**E**) Deformations at various positions in (C) with the front view in the first row and the bottom view in the second row.

A

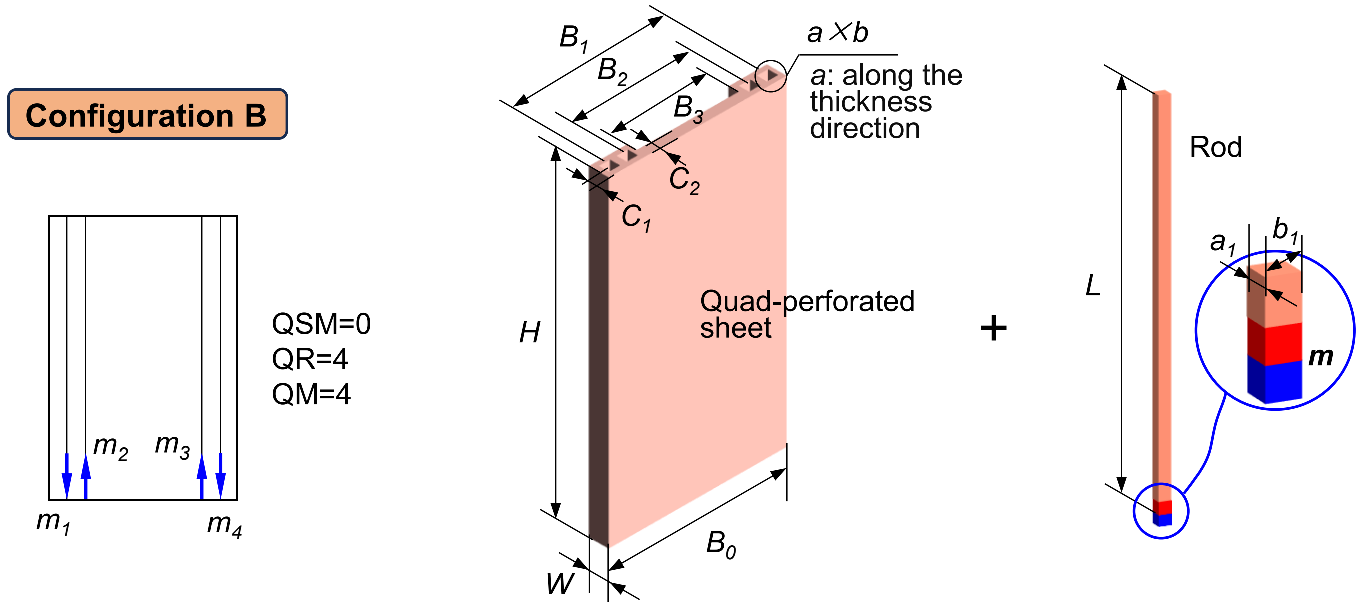

B

Symbol	Value
H	60 mm
W	3 mm
B_0	30 mm
B_1	25.7 mm
B_2	18.9 mm
B_3	14.8 mm
C_1	1.5 mm
C_2	1 mm
a	2 mm
b	2.5 mm
a_1	1.3 mm
b_1	1.8 mm
L	70 mm

Supplementary Fig. 19. Detailed parameters for Configuration B of 2D magnetization reprogramming in Supplementary Fig. 16.

A

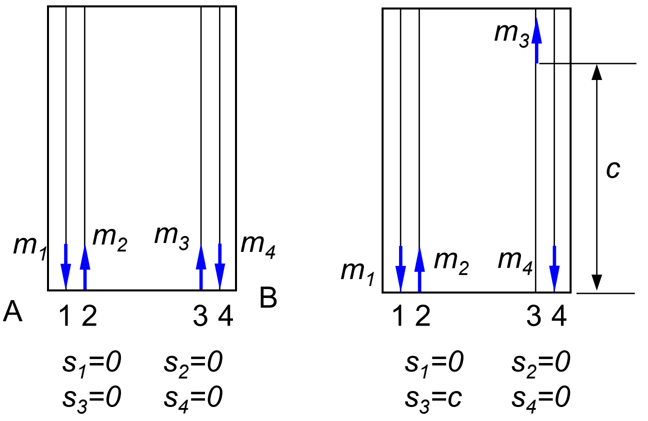

B

Symbol	Value
m_1, m_2, m_3, m_4	1.015×10^{-2} Am^2
c	0 mm, 60 mm

C

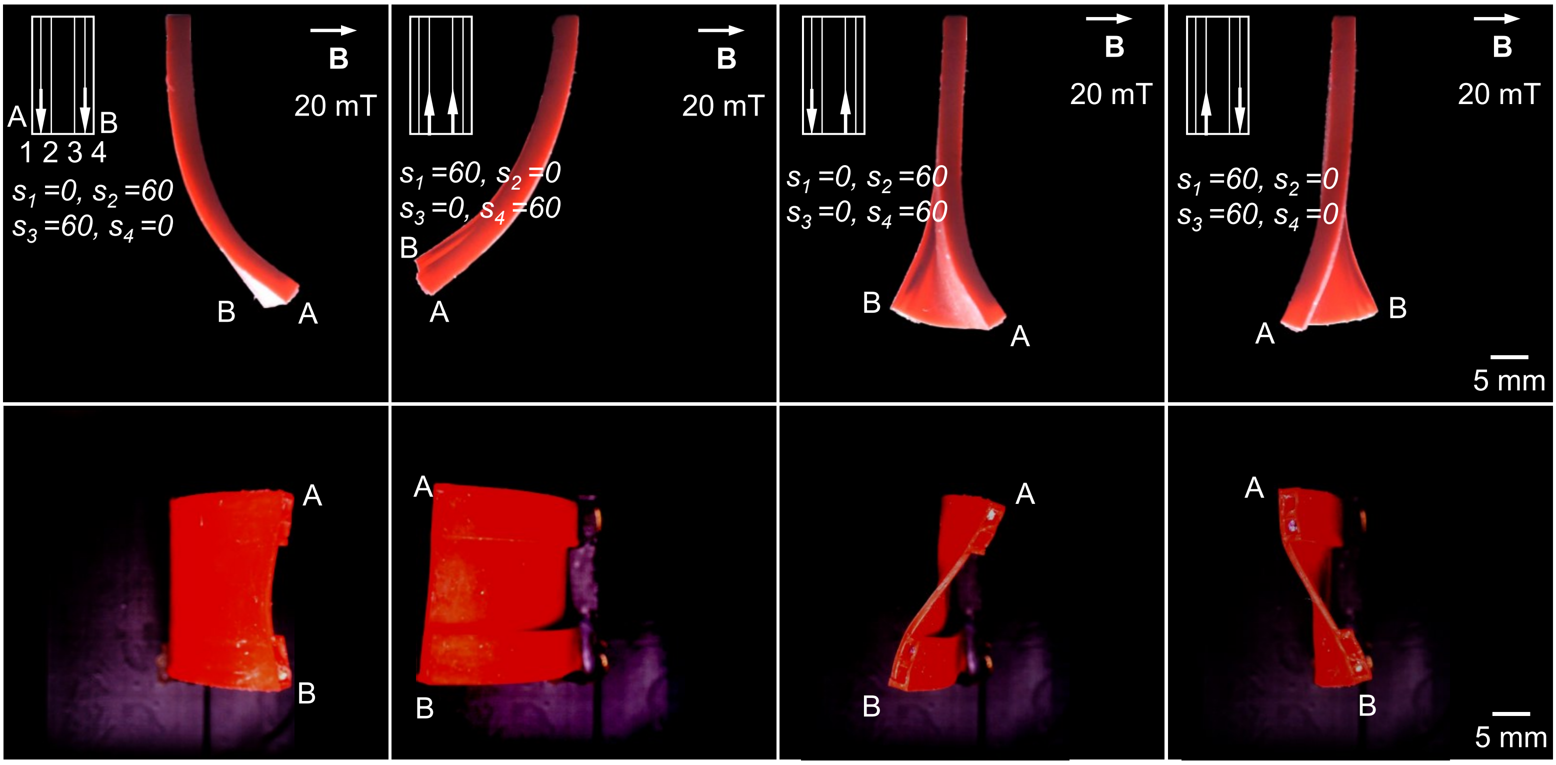

Supplementary Fig. 20. Shape change characteristics of Configuration B in 2D magnetization reprogramming. (**A**) Schematic illustration depicting the positions of the magnetic units. To describe the correspondence between the front and bottom views in the deformation of the sheet, the two lower corners of the sheet are denoted as points A and B. (**B**) Parameter settings for observing deformation at various positions. Note that when c is 60 mm, the magnetic unit is located on the outer side of the sheet. (**C**) Deformations at various positions in (B) with the front view in the first row and the bottom view in the second row.

A

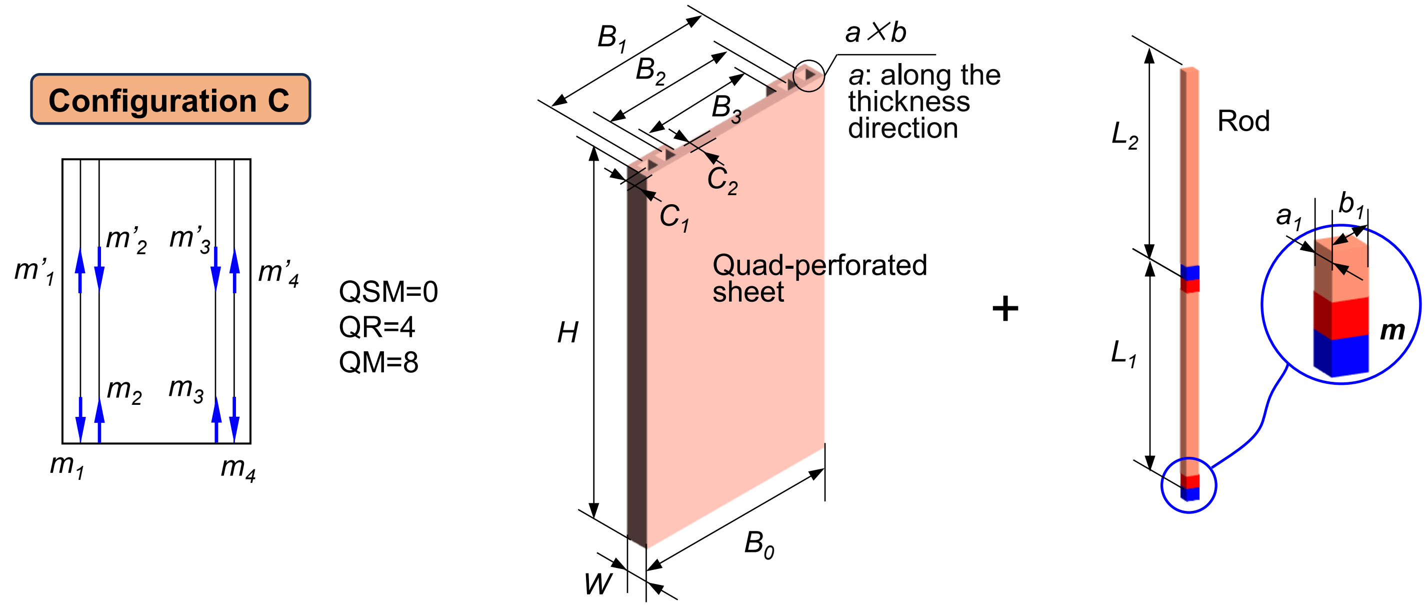

B

Symbol	Value
H	60 mm
W	3 mm
B_0	30 mm
B_1	25.7 mm
B_2	18.9 mm
B_3	14.8 mm
C_1	1.5 mm
C_2	1 mm
a	2 mm
b	2.5 mm
a_1	1.3 mm
b_1	1.8 mm
L_1	30 mm
L_2	40 mm

Supplementary Fig. 21. Detailed parameters for Configuration C of 2D magnetization reprogramming in Supplementary Fig. 16.

A

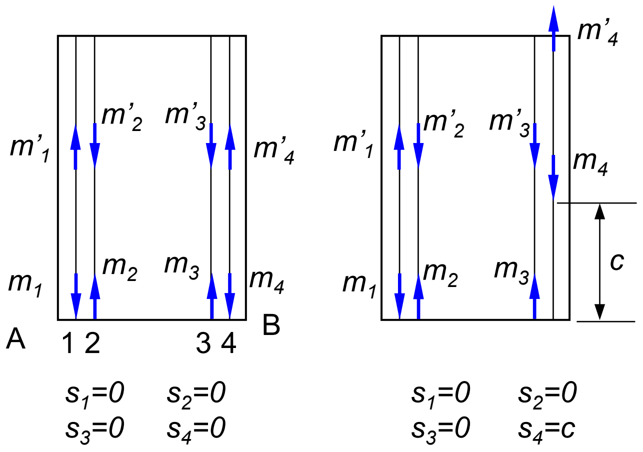

B

Symbol	Value
m_1, m_2, m_3, m_4 m'_1, m'_2, m'_3, m'_4	1.015×10^{-2} Am^2
c	0 mm, 60 mm

C

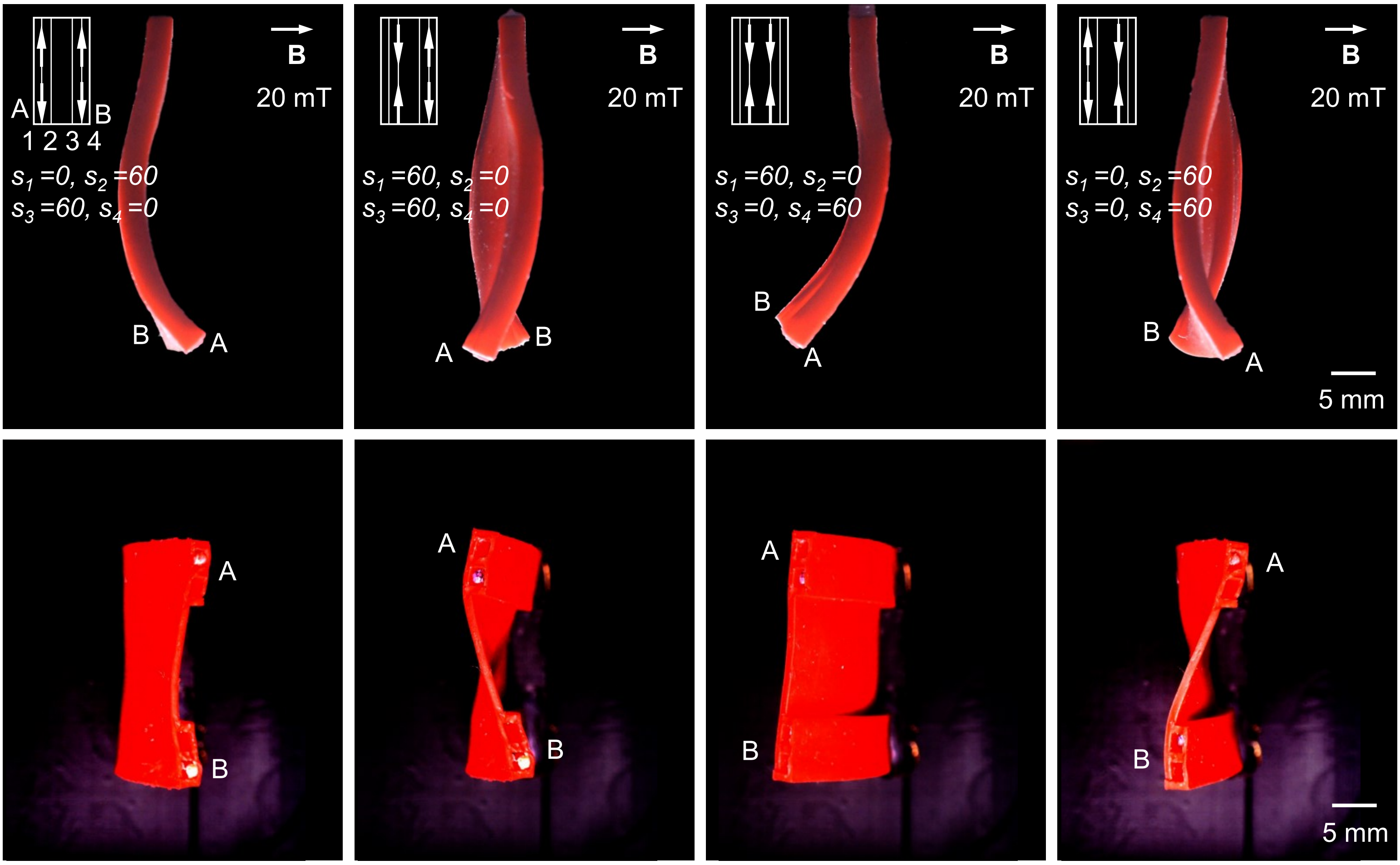

Supplementary Fig. 22. Shape change characteristics of Configuration C in 2D magnetization reprogramming. (**A**) Schematic illustration depicting the positions of the magnetic units. Points A and B are used to designate the two lower corners of the sheet, in order to illustrate the relationship between the front and bottom views during the sheet's deformation. (**B**) Parameter settings for observing deformation at various positions. Note that when c is 60 mm, the magnetic unit is located on the outer side of the sheet. (**C**) Deformations at various positions in (B) with the front view in the first row and the bottom view in the second row.

A

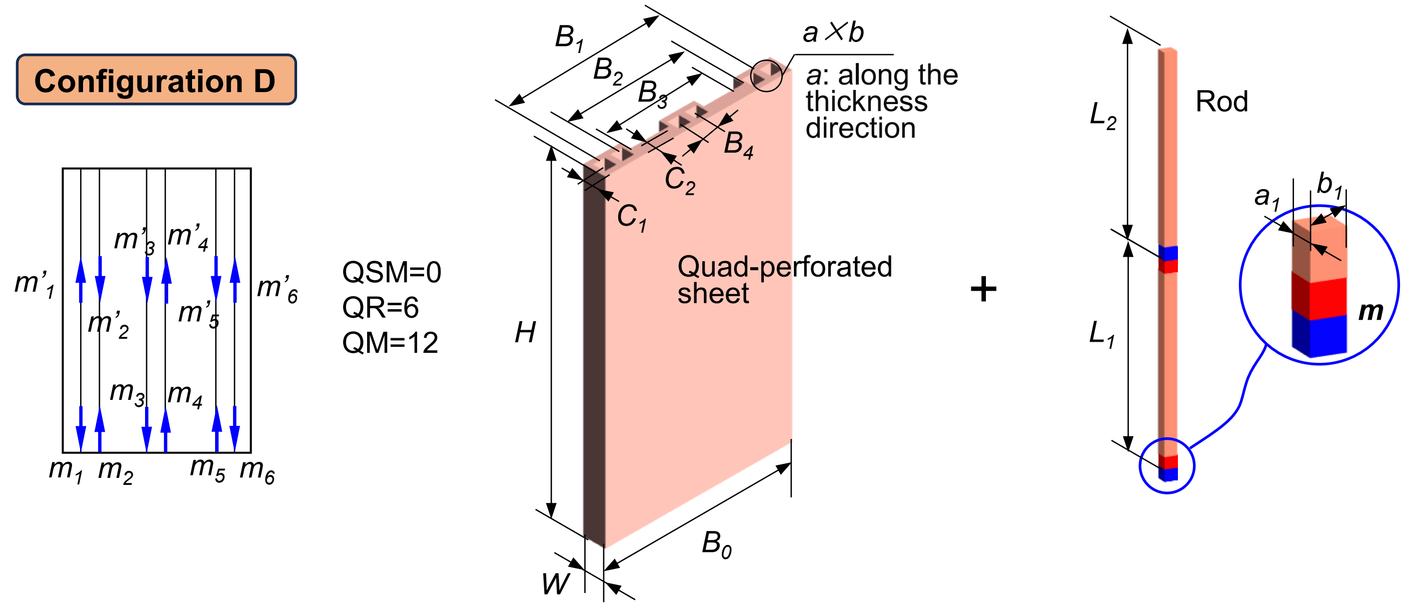

B

Symbol	Value
H	60 mm
W	3 mm
B_0	38 mm
B_1	33.7 mm
B_2	26.9 mm
B_3	22.6 mm
B_4	3.4 mm
C_1	1.5 mm
C_2	1 mm
a	2 mm
b	2.5 mm
a_1	1.3 mm
b_1	1.8 mm
L_1	30 mm
L_2	40 mm

Supplementary Fig. 23. Detailed parameters for Configuration D of 2D magnetization reprogramming in Supplementary Fig. 16.

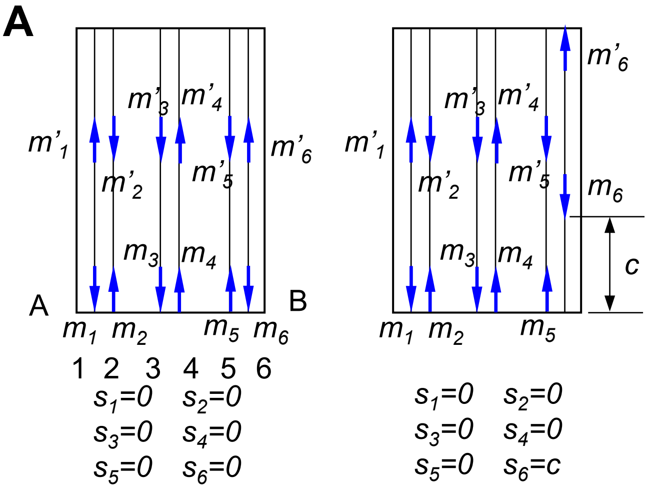

B

Symbol	Value
m_1, m_2, m_3, m_4, m_5, m_6 m'_1, m'_2, m'_3, m'_4, m'_5, m'_6	1.015×10^{-2} Am^2
c	0 mm, 60 mm

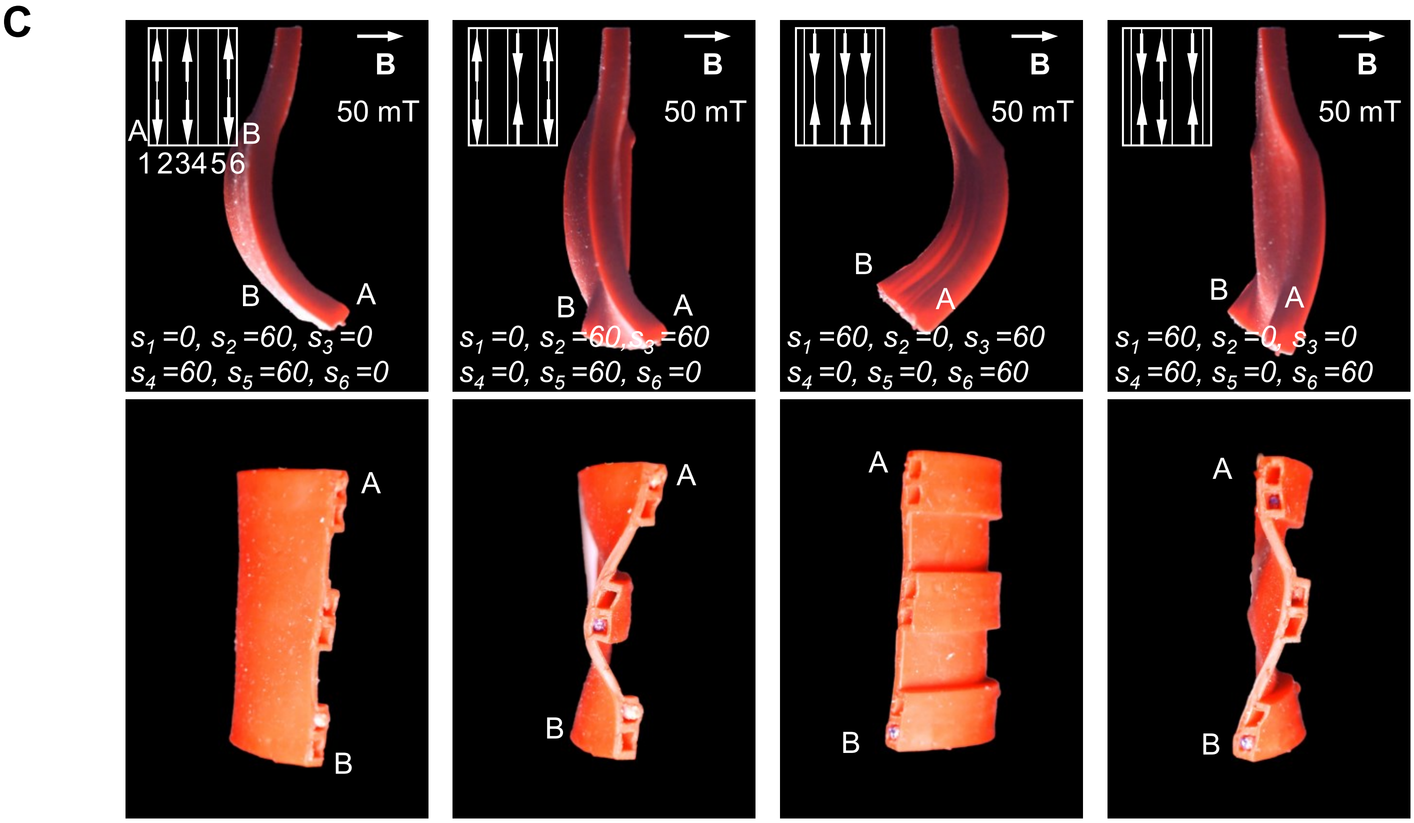

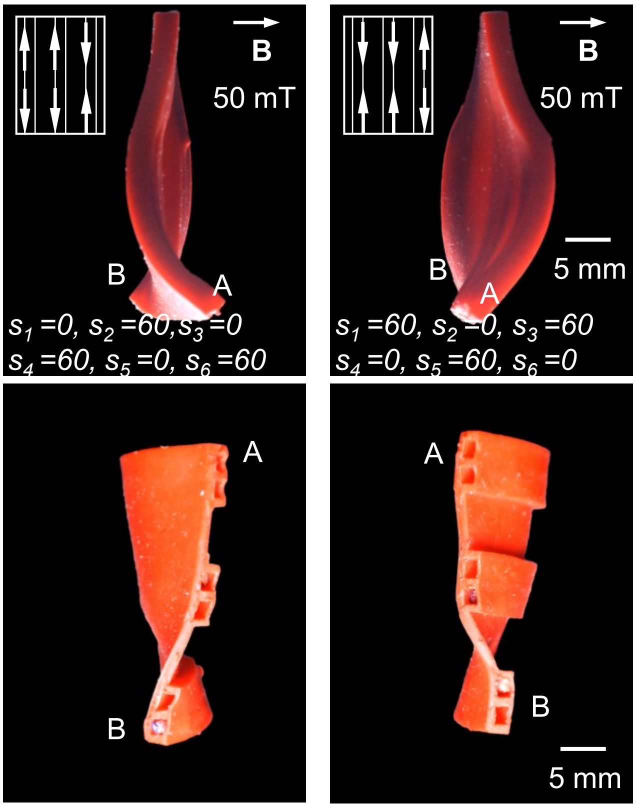

Supplementary Fig. 24. Shape change characteristics of Configuration D in 2D magnetization reprogramming. (**A**) Schematic illustration depicting the positions of the magnetic units. Points A and B are used to designate the two lower corners of the sheet, in order to illustrate the relationship between the front and bottom views during the sheet's deformation. (**B**) Parameter settings for observing deformation at various positions. Note that when c is 60 mm, the magnetic unit is located on the outer side of the sheet. (**C**) Deformations at various positions in (B) with the front view in the first and third rows and the bottom view in the second and fourth rows.

A

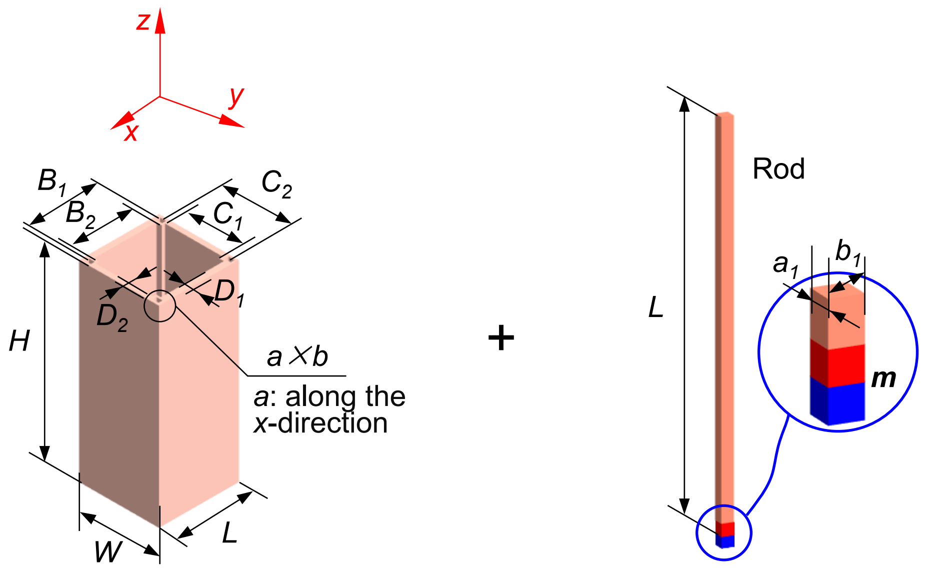

B

Symbol	Value
H	60 mm
W	25 mm
L	25 mm
B_1	20.7 mm
B_2	16.4 mm
C_1	16.4 mm
C_2	19.7 mm
D_1	1 mm
D_2	1 mm
a	1.5 mm
b	2 mm
a_1	1.3 mm
b_1	1.8 mm
L	70 mm

Supplementary Fig. 25. Detailed parameters for a specific configuration of three-dimensional (3D) magnetization reprogramming. This 3D box-shaped soft structure represents an extension of 2D reprogramming within sheets, enabling a broader range of reprogramming possibilities in 3D space.

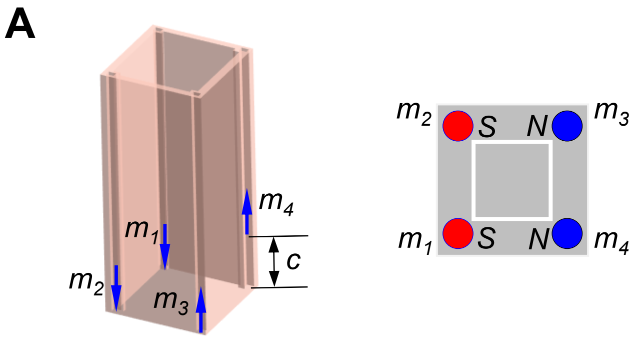

B

Symbol	Value
m_1, m_2, m_3, m_4	1.015×10^{-2} Am^2
c	0 mm, 60 mm

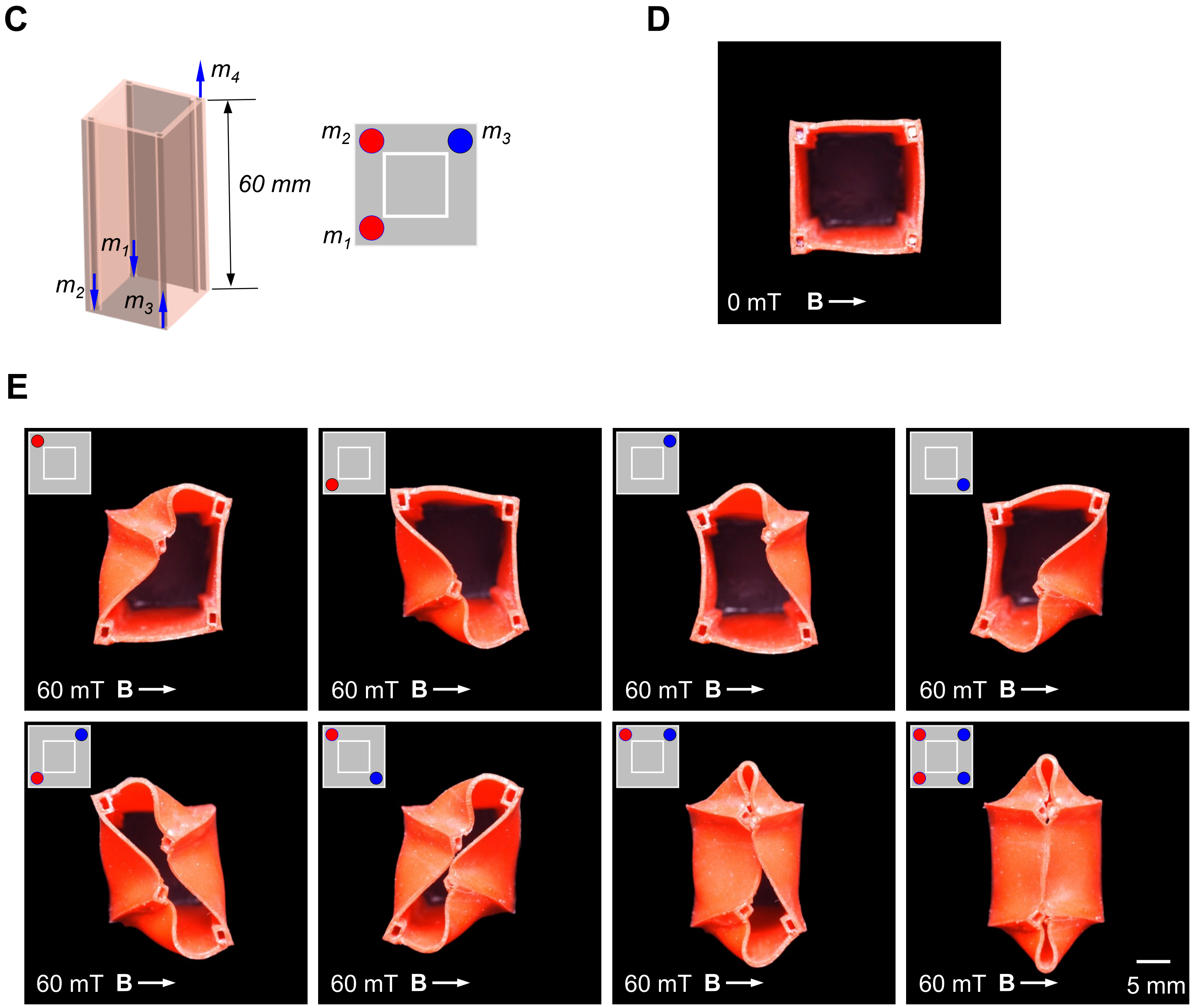

Supplementary Fig. 26. Shape change characteristics of 3D magnetization reprogramming. (**A**) Schematic illustration showing the positions of the magnetic units, where the right-side diagram is a top view of the left-side diagram. In the top view, red circles represent the S poles of the magnetic units, and blue circles indicate the N poles. (**B**) Parameter settings for observing deformation at various positions. Note that when c is 60 mm, the magnetic unit is located on the outer side of the 3D box-shaped soft structure, as shown in (**C**). (**D**) Bottom view of the 3D box-shaped soft structure with no magnetic field applied. (**E**) Bottom view of the deformation of the 3D box-shaped soft structure in different configurations under the same magnetic field.

A

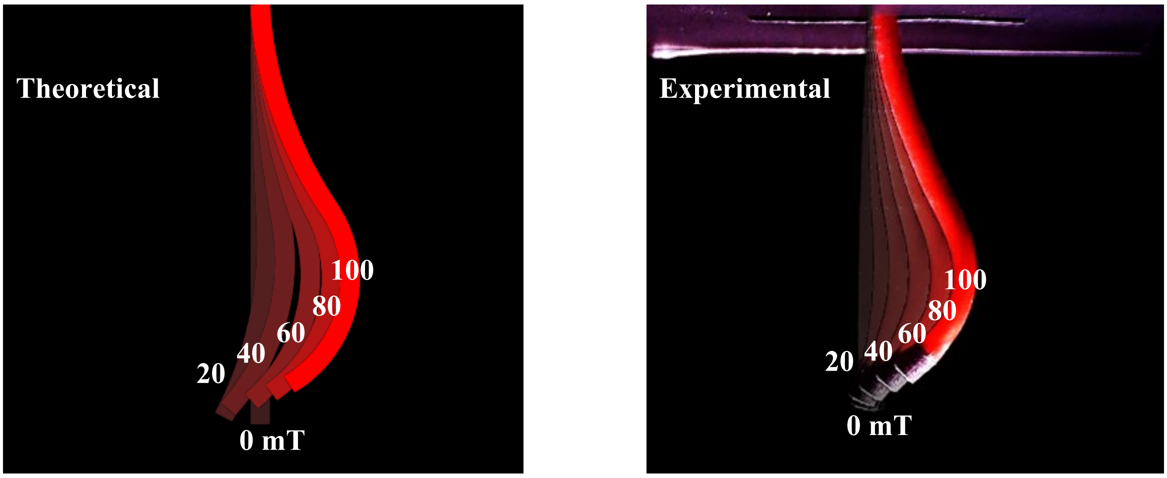

B

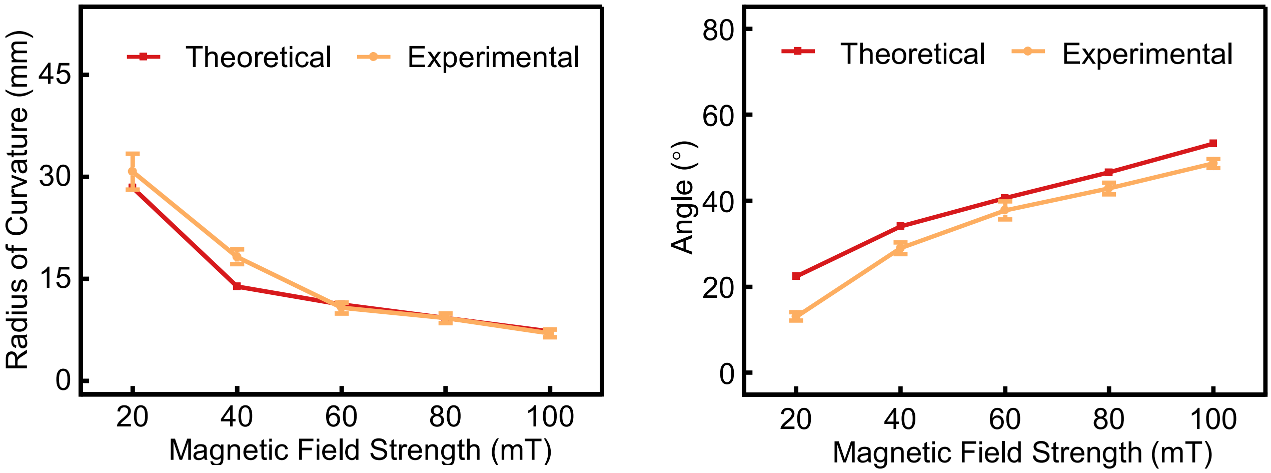

Supplementary Fig. 27. Comparison of theoretical and experimental values for the deformation of soft tubes in 1D magnetization reprogramming. (**A**) The figures present the scenario of Configuration B in 1D magnetization reprogramming with d_{R12} = 12mm under a uniform magnetic field increasing from 0 mT to 100 mT in increments of 20 mT. (**B**) Theoretical and experimental values of soft tube deformation are characterized using two representative descriptors: radius of curvature and terminal bending angle (Supplementary Fig. 29). Experimental data are presented as mean ± SD (n=3 tests).

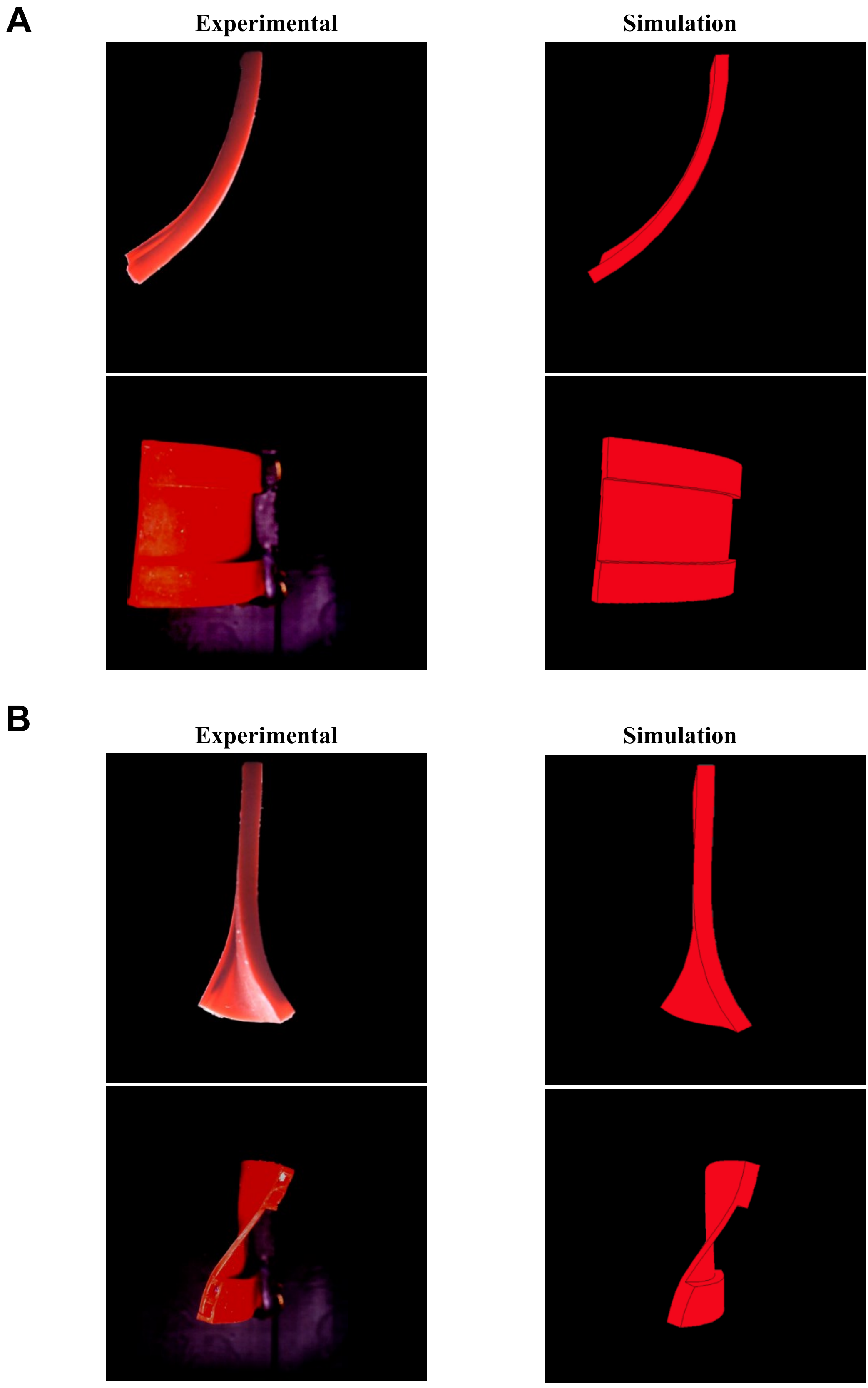

Supplementary Fig. 28. Comparison of experimental and simulation results of Configuration B in 2D magnetization reprogramming. The figures display the deformation of sheets with two different configurations from Supplementary Fig. 20 under a magnetic field of 20 mT directed to the right. The images in the first row represent the front views, while those in the second row are the top views. (**A**) s_1 =60, s_2 =0, s_3 =0, s_4 =60. (**B**) s_1 =0, s_2 =60, s_3 =0, s_4 =60.

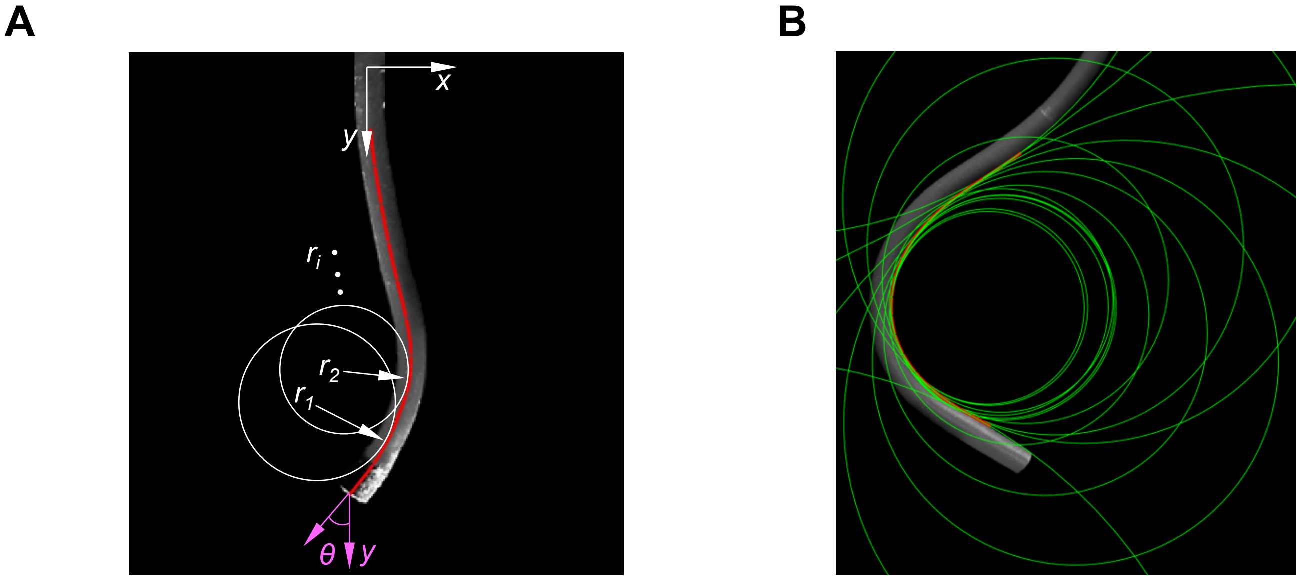

Supplementary Fig. 29. Schematic diagram of quantification of soft tube deformation. (**A**) Parameter schematic. Based on the deformed contour of the soft tube, we identify the characteristic curve (indicated in red in the diagram) and calculate the radius of curvature r_i and the terminal bending angle θ with respect to the *y*-axis. (**B**) Employing MATLAB to obtain the characteristic curve and fit the radius of curvature of the characteristic curve. We utilize circles to approximate the characteristic curve (depicted as green circles and the red dash line in the diagram), selecting the smallest radius from among these circles as the radius of curvature for the characteristic curve. This simplified approach, which involves the radius of curvature and the terminal bending angle, provides a concise description of the deformation extent of the soft tube.

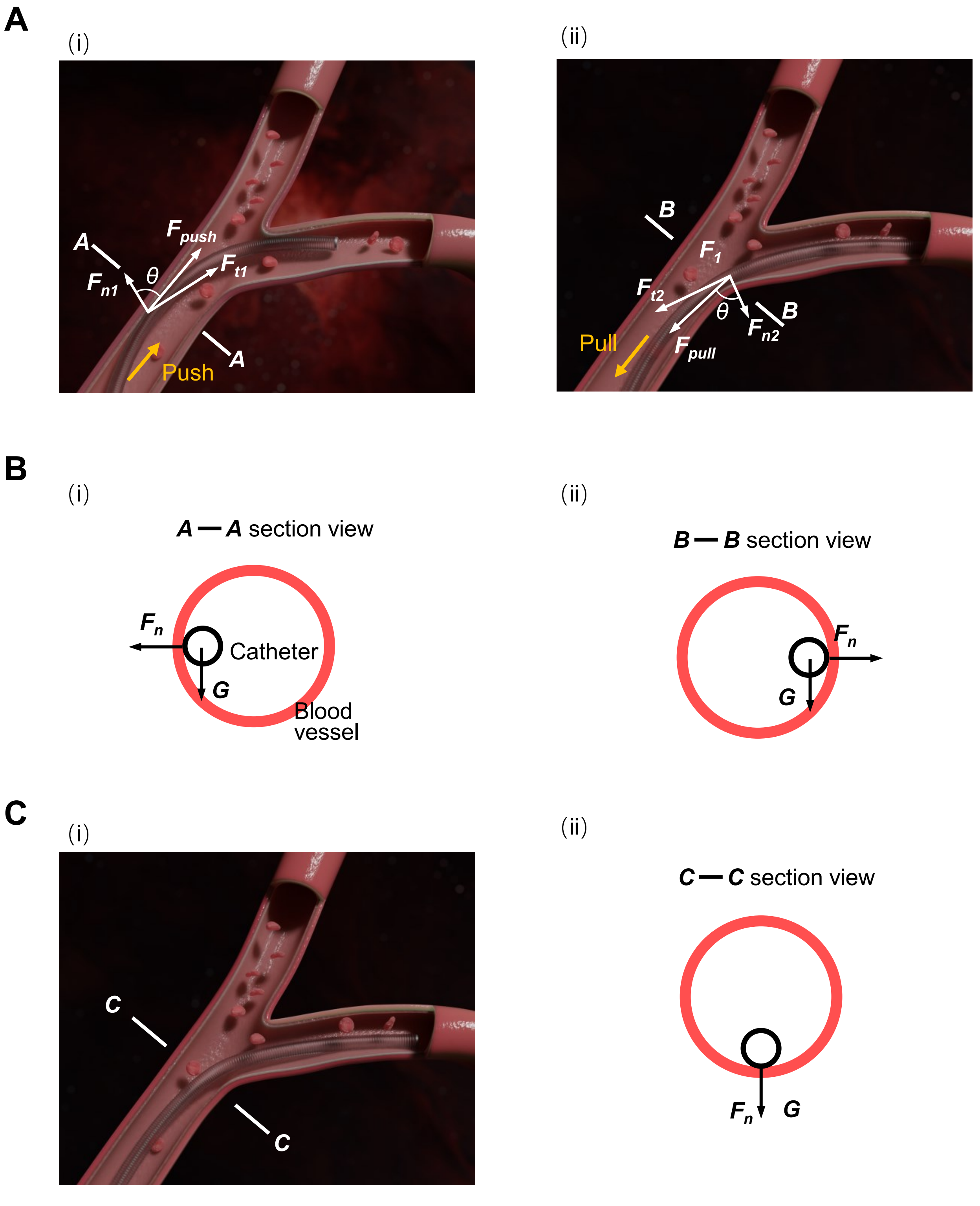

Supplementary Fig. 30. Analysis diagram of a catheter navigating through blood vessels. (**A**) Force analysis diagram of the catheter's contacts with the vessel wall. (i) Pushing the catheter forward. (ii) Pulling the catheter backward. (**B**) Section view at sections A-A and B-B in (A). (**C**) Cross-sectional diagram of the ideal contact state between the catheter and the blood vessel. (i) Sectional view along the plane formed by the main vessel axis and the branch vessel axis. (ii) Section view at section C-C in C(i). In the ideal contact state, the catheter does not contact the sides of the vessel but only has contact with the bottom of the vessel.

A

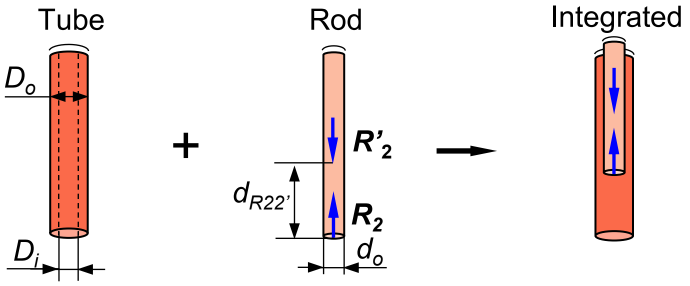

Symbol	Value
D_o	2.6 mm
D_i	1.7 mm
d_o	1.4 mm
$d_{R22'}$	12 mm
m	1.02×10^{-3} Am^2

B

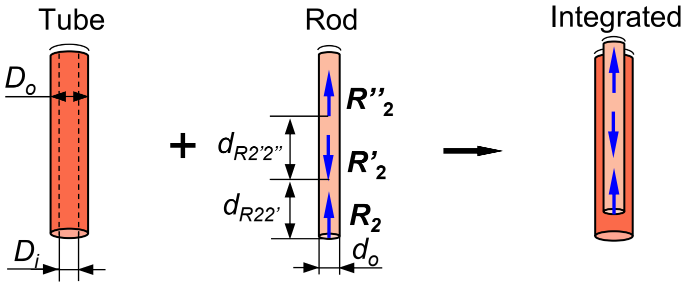

Symbol	Value
D_o	2.6 mm
D_i	1.7 mm
d_o	1.4 mm
$d_{R22'}$,$d_{R2'2''}$	12 mm
m	1.02×10^{-3} Am^2

C

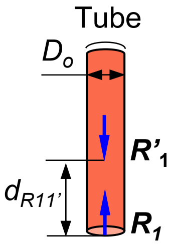

Symbol	Value
D_o	2.6 mm
$d_{R11'}$	12 mm
m	1.02×10^{-3} Am^2

Supplementary Fig. 31. Design parameters of the soft tubes involved in the "Contact-free object navigation" section. (**A**) The soft tube with two magnetic units. (**B**) The soft tube containing three magnetic units. (**C**) Control group, using soft tubes controlled by existing methods.

A

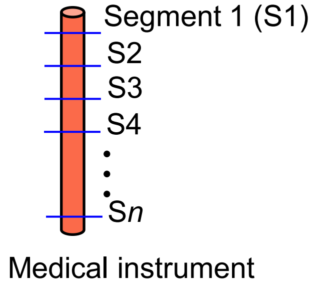

B

	State	
	**0**	**1**
**Deformation State**	Unbent	Bent
**Magnetic Properties**	Non-magnetic	Magnetic

C

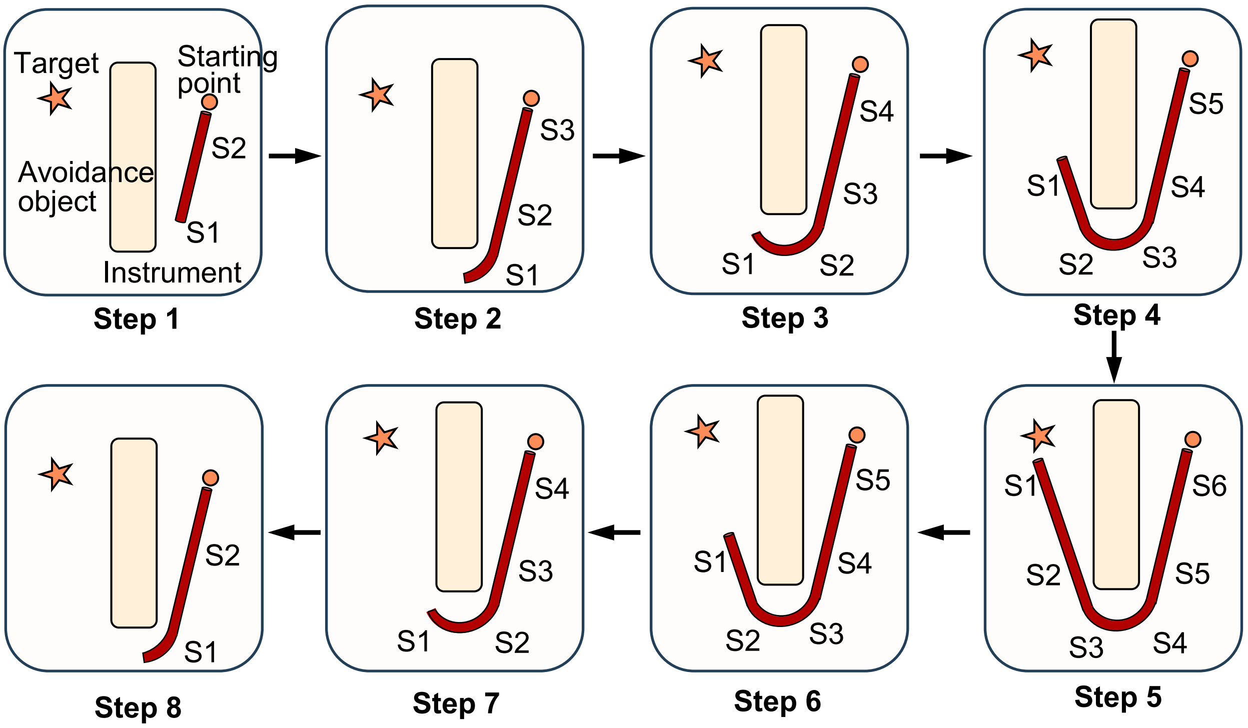

Supplementary Fig. 32. Detailed breakdown of the steps involved in bypassing the avoidance object as depicted in Fig. 2a(iii). (**A**) Segmented diagram of the medical instrument illustrating the magnetization state of different segments. (**B**) Table detailing the state of each segment of the medical instrument. (**C**) Step-by-step breakdown of the medical instrument's bypassing process. The medical instrument advances to reach the target position and then retracts. The magnetization state of the various segments during this process is shown in Supplementary Fig. 33.

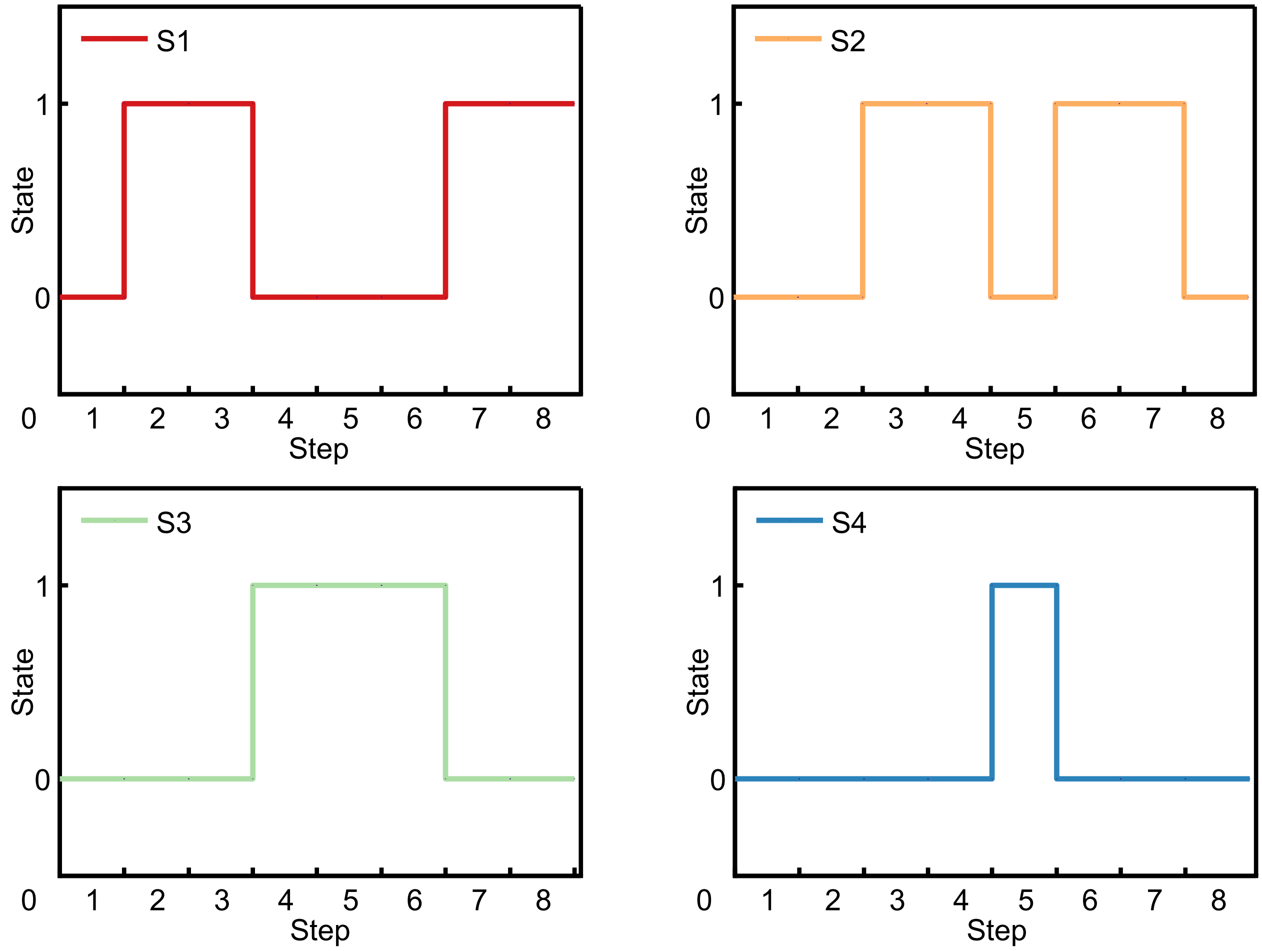

Supplementary Fig. 33. Magnetization state diagram of each segment of the medical instrument during the avoidance object bypassing process as depicted in Fig. 2a(iii) and Supplementary Fig. 32. The segments S1, S2, S3, and S4 correspond to the four sections of the medical instrument as divided in Supplementary Fig. 32. The meanings of the states shown in the diagram can be found in the magnetization state definitions in Supplementary Fig. 32B.

A

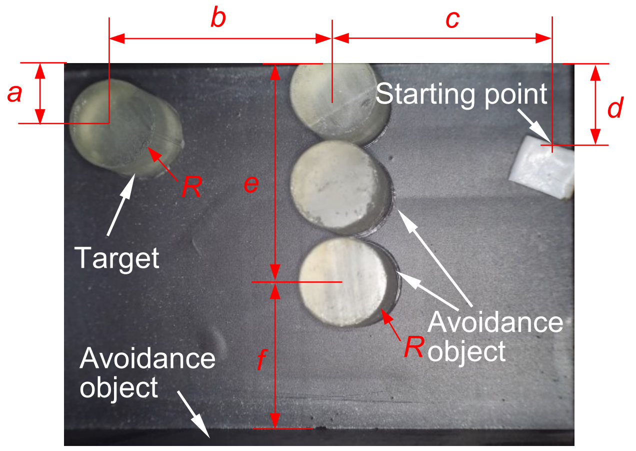

Symbol	Value
a	8.5 mm
b	31 mm
c	31 mm
d	11 mm
e	34 mm
f	19 mm
R	6 mm

B

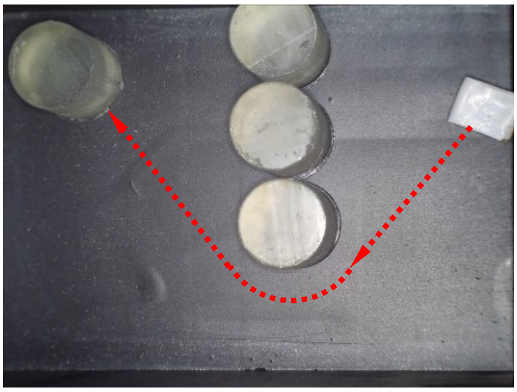

Supplementary Fig. 34. An experimental setup designed based on Fig. 2a(iii). (**A**) Dimensional diagram of the scene layout. (**B**) Path of the soft tube bypassing avoidance objects.

A

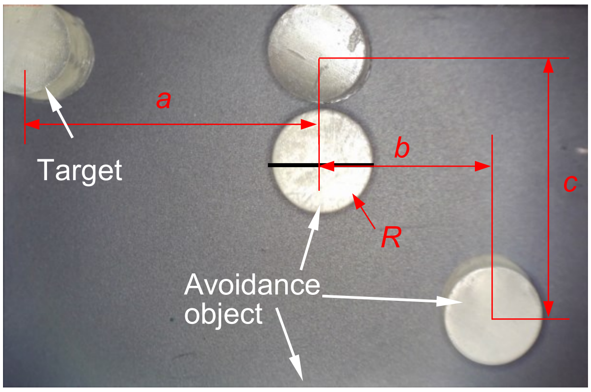

Symbol	Value
a	34 mm
b	19 mm
c	30 mm
R	6 mm

B

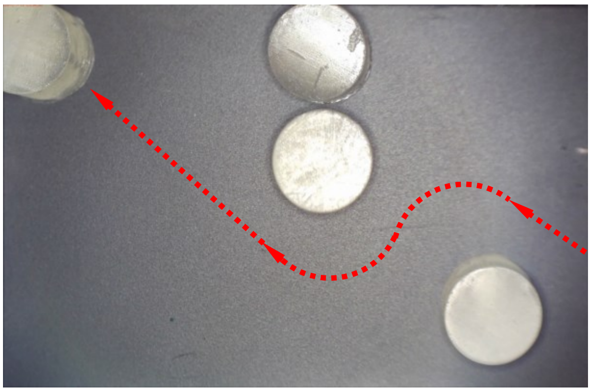

Supplementary Fig. 35. An extended and more complex experimental setup based on Fig. 2a(iii) and Supplementary Fig. 34. (**A**) Dimensional diagram of the scene layout. (**B**) Path of the soft tube bypassing avoidance objects.

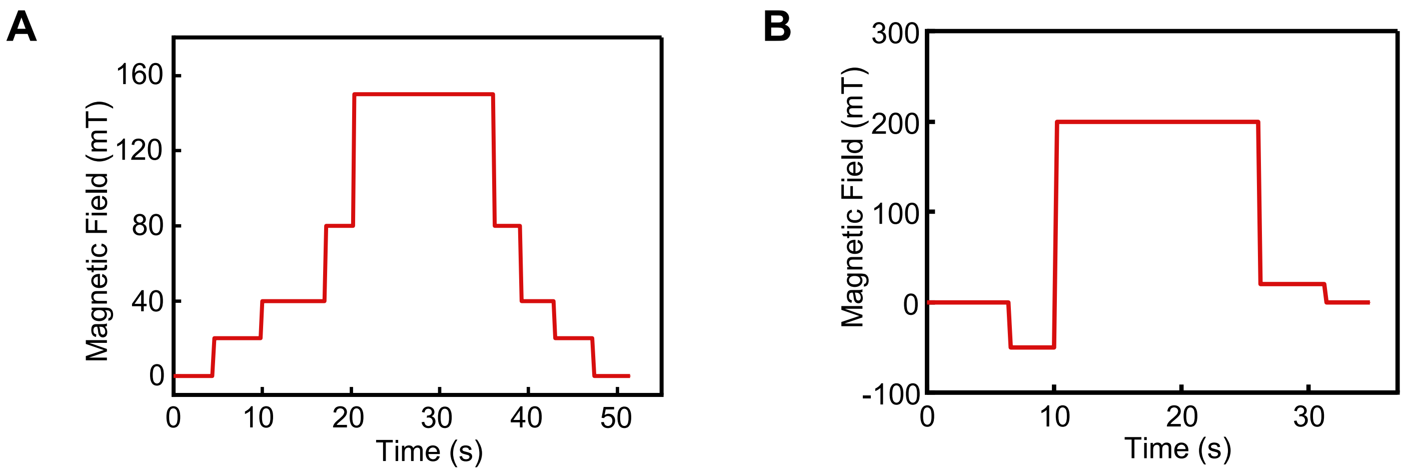

Supplementary Fig. 36. Magnetic field applied as the soft tube bypasses the avoidance objects, corresponding to scenes in Supplementary Fig. 34 and Supplementary Fig. 35. (**A**) Magnetic field for the soft tube bending into one bend in Supplementary Fig. 34. (**B**) Magnetic field for the soft tube bending into two bends in Supplementary Fig. 35.

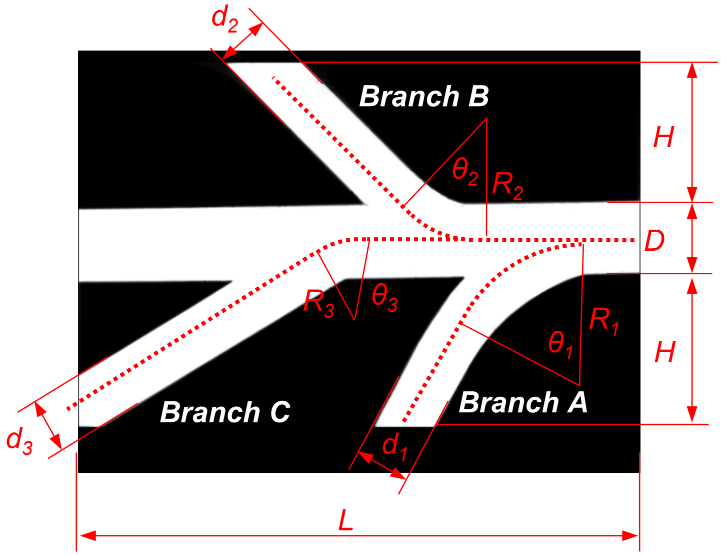

Symbol	Value
L	85 mm
H	20 mm
D	9 mm
d_1	7 mm
d_2	7 mm
d_3	7 mm
R_1	30 mm
R_2	20 mm
R_3	10 mm
θ_1	60°
θ_2	45°
θ_3	30°

Supplementary Fig. 37. A blood vessel model designed based on Fig. 2a(ii). This blood vessel model includes three vascular branches (A, B, and C) to provide different operational paths for the experiment.

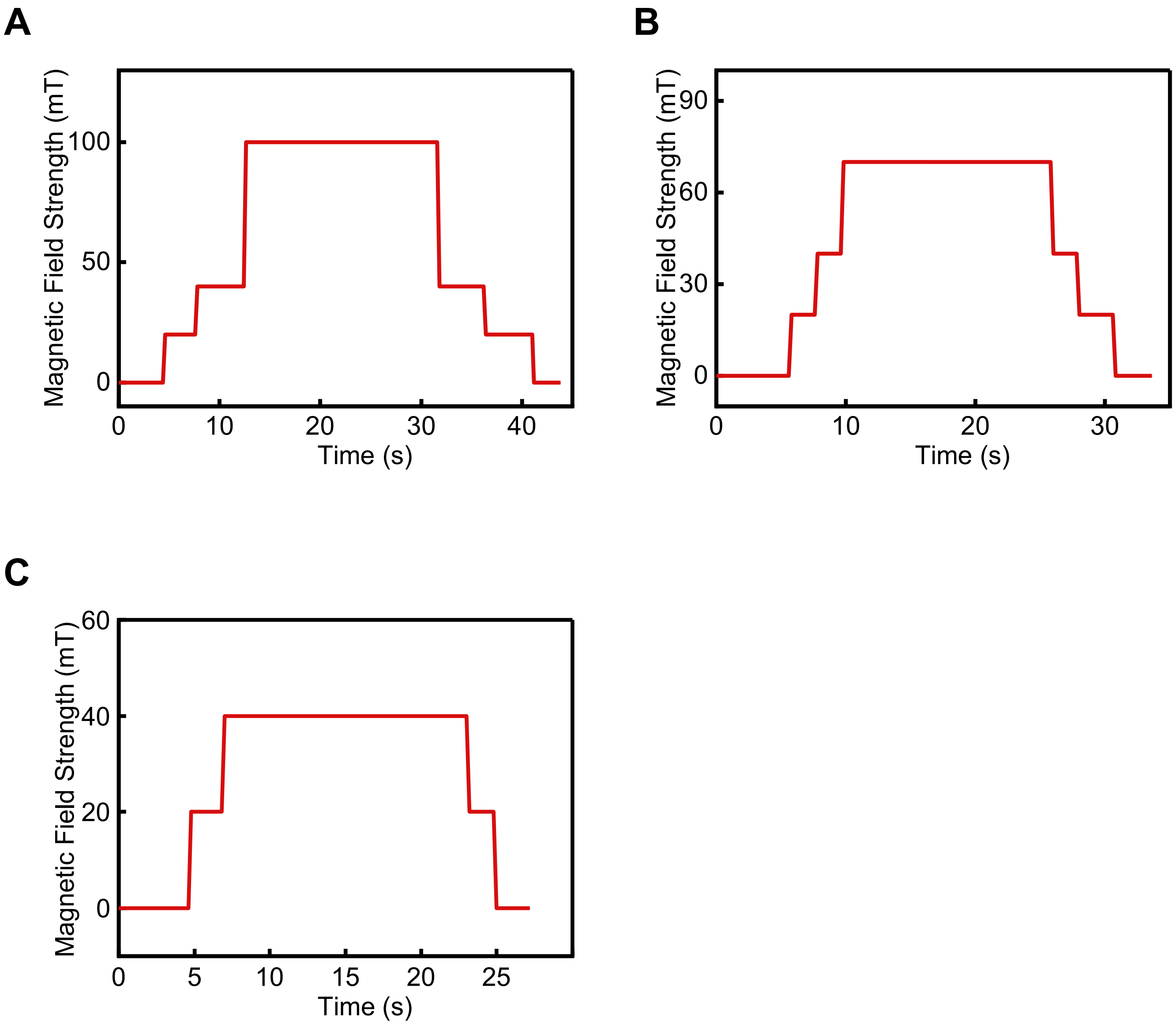

Supplementary Fig. 38. Magnetic field applied as the soft tube passes through different vascular branches. (**A**) Magnetic field for branch A. (**B**) Magnetic field for branch B. (**C**) Magnetic field for branch C.

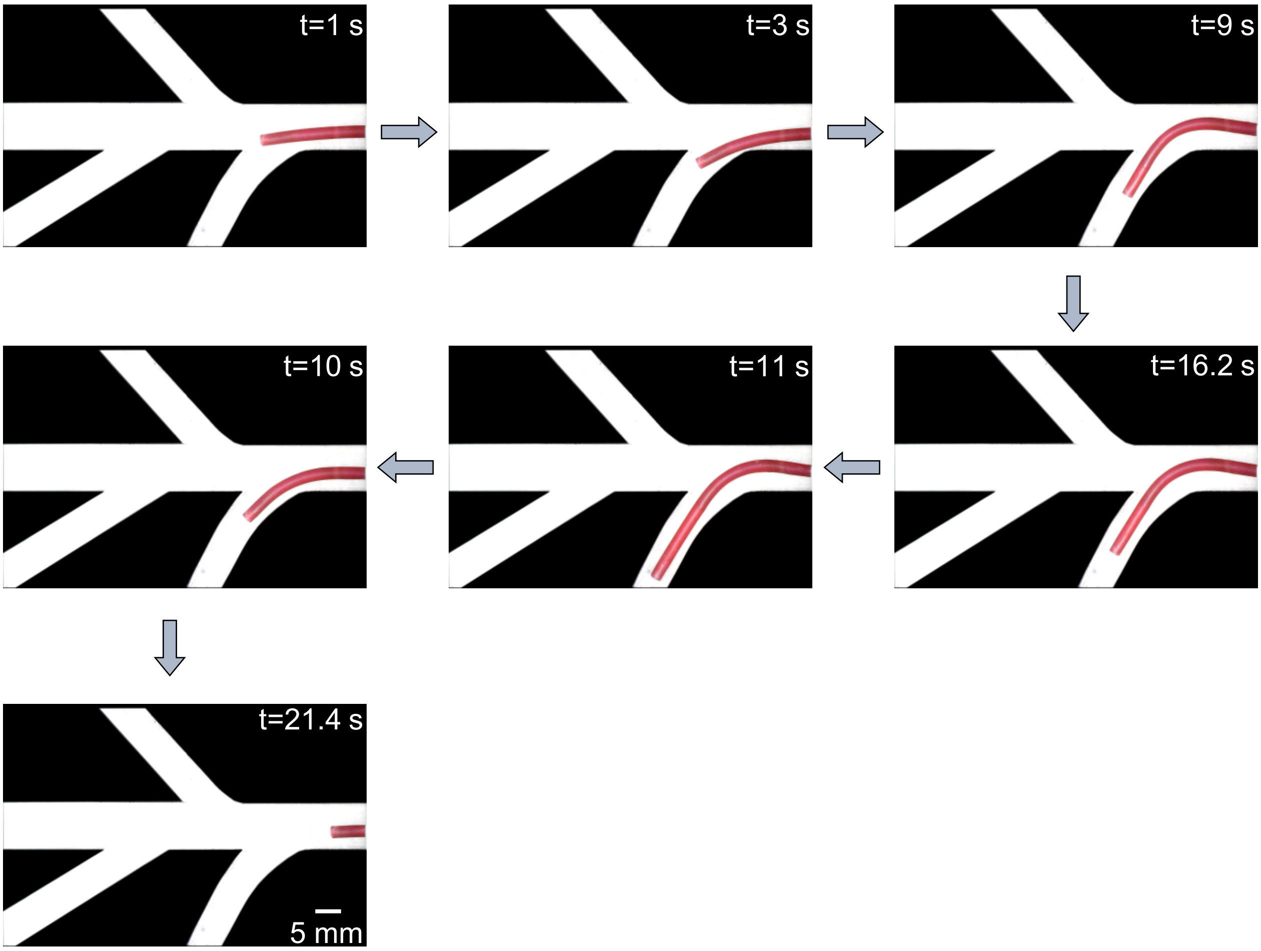

Supplementary Fig. 39. Video snapshots depicting the soft tube navigating through branch A of the blood vessel model without contacts. The snapshots above are taken from Supplementary Video 5.

A

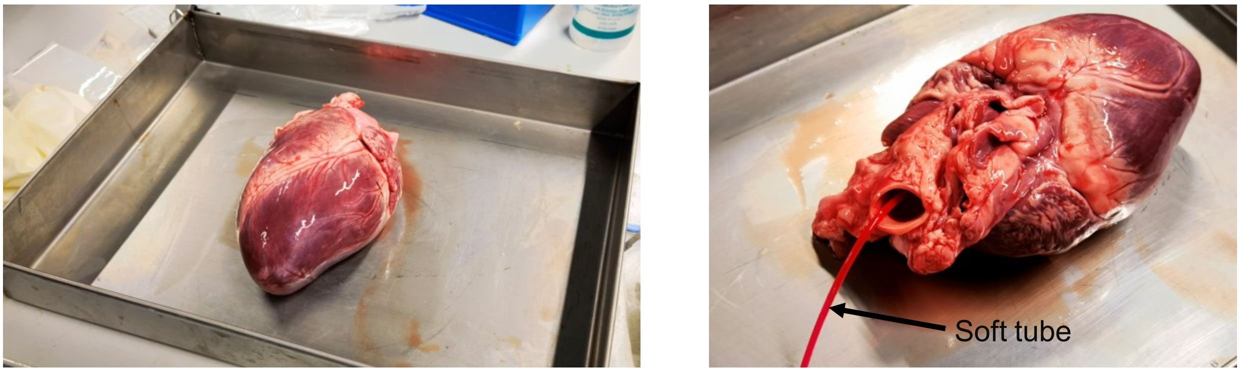

B

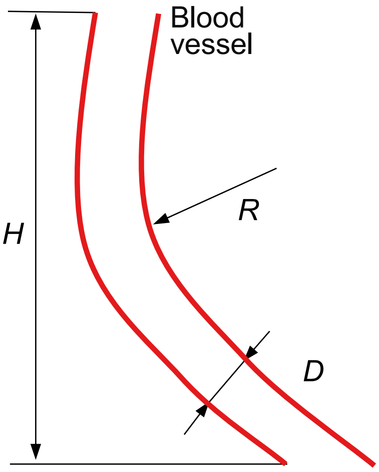

Symbol	Value
R	38 mm
D	14.8 mm
H	44.2 mm

Supplementary Fig. 40. *Ex vivo* experimental setup with porcine vasculature. (**A**) Diagram of the *ex vivo* porcine heart with the soft tube placed within the target vessel. (**B**) Dimensional parameters of the target vessel.

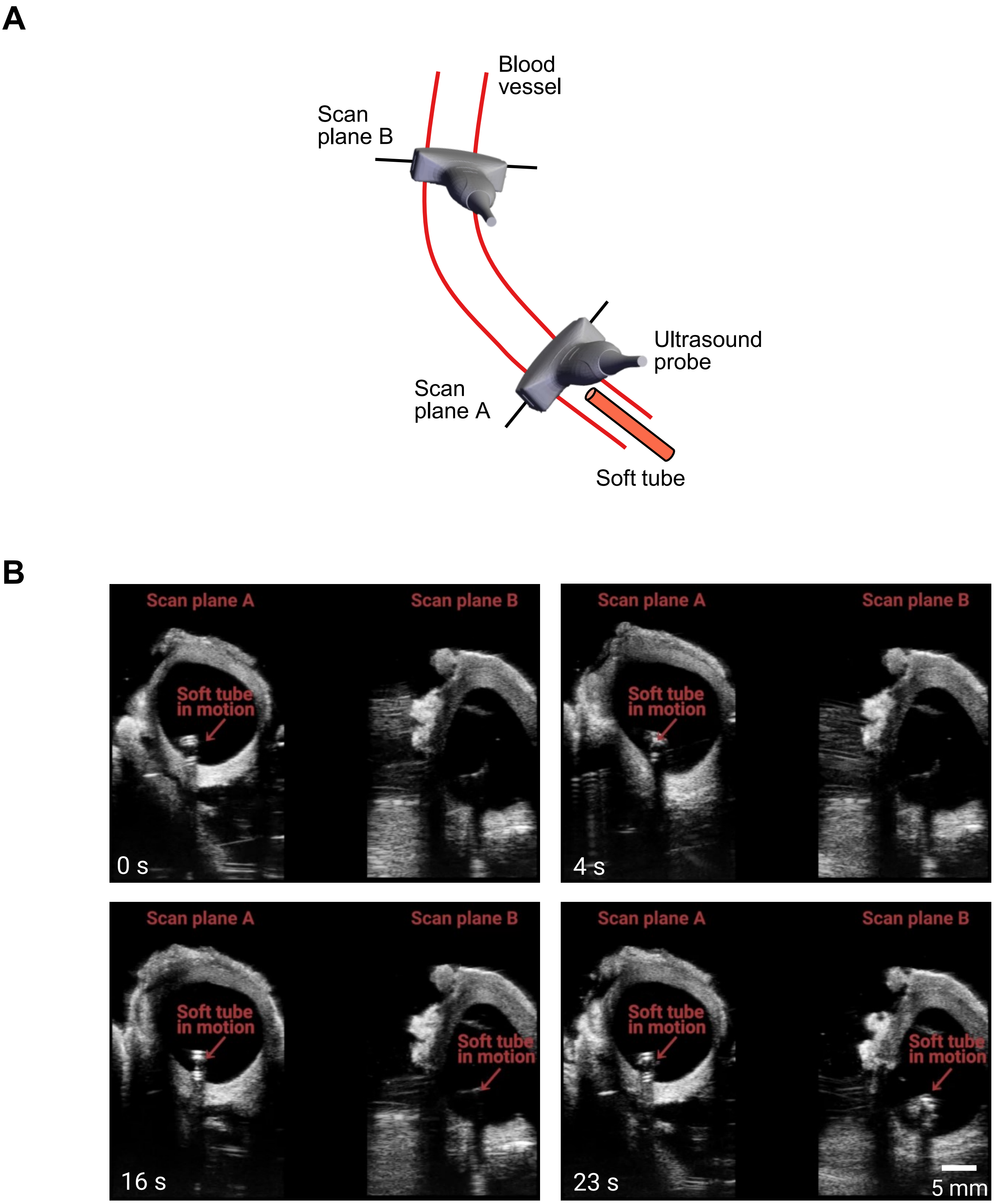

Supplementary Fig. 41. ***Ex vivo*** **experiment.** (**A**) Description of ultrasound probe positions. The ultrasound probe is placed at scan planes A and B, perpendicular to the axis of the vessel, to observe the motion of the soft tube. (**B**) Video snapshots of the ultrasound imaging at scan planes A and B (Supplementary Video 6). Initially, the soft tube appears only in scan plane A. Later, the soft tube is observed in both scan planes A and B, indicating the movement of the soft tube from scan planes A to B as shown in (A). The video snapshots demonstrate that the soft tube does not collide with the vessel walls on either side. The gravity of the soft tube needs to be counteracted by the reaction force of the vessel, resulting in the soft tube contacting the lower side of the vessel. This aligns with the ideal state described in Supplementary Fig. 30C, thus preventing damage to the vessel.

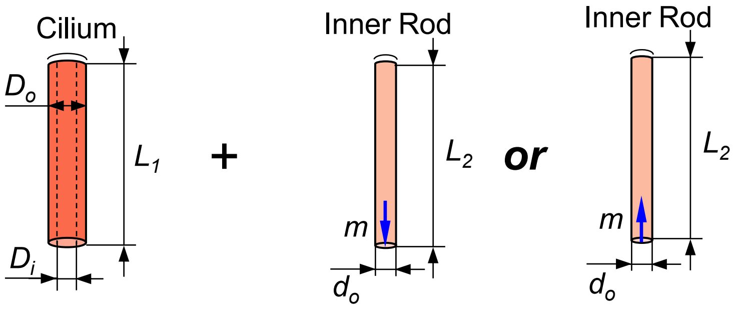

Symbol	Value
D_o	1.4 mm
D_i	0.9 mm
d_o	0.8 mm
L_1	8 mm
L_2	15 mm
m	4.95×10^{-4} Am^2

Supplementary Fig. 42. Parameters of cilia involved in targeted cilia activation in the "Reprogrammable cilia array" section.

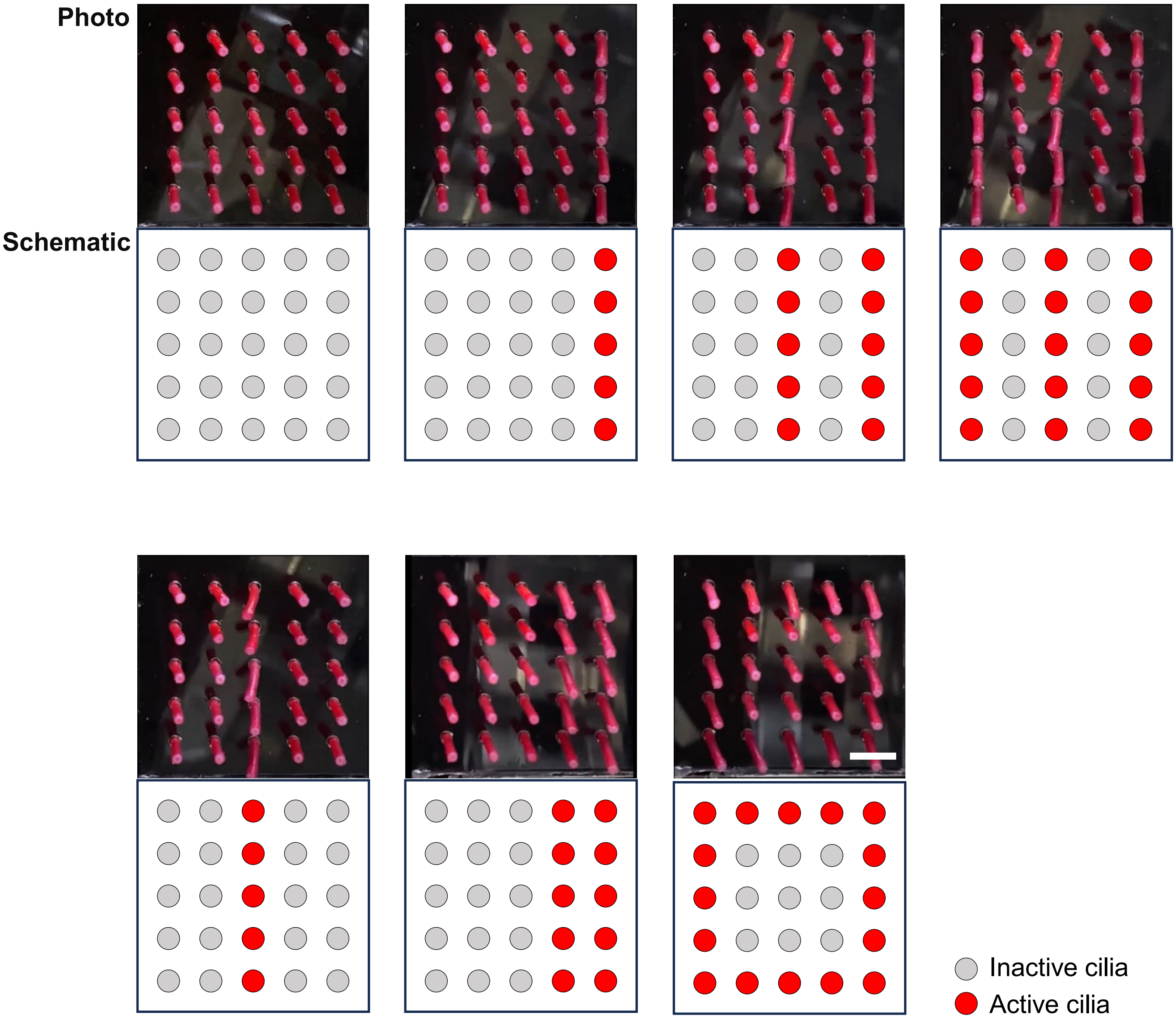

Supplementary Fig. 43. Display of targeted cilia activation. The cilia array in various states is depicted through snapshots derived from the experimental videos (Supplementary Video 7), directly below which are corresponding schematic diagrams. These diagrams illustrate the activation states of the ciliary array, including the inactive state and active state. These figures demonstrate the control of targeted cilia activation in numerous configurations, achievable through the real-time in-situ magnetization reprogramming method proposed in this research. Rotating magnetic field: 45mT. Scale bar: 5mm.

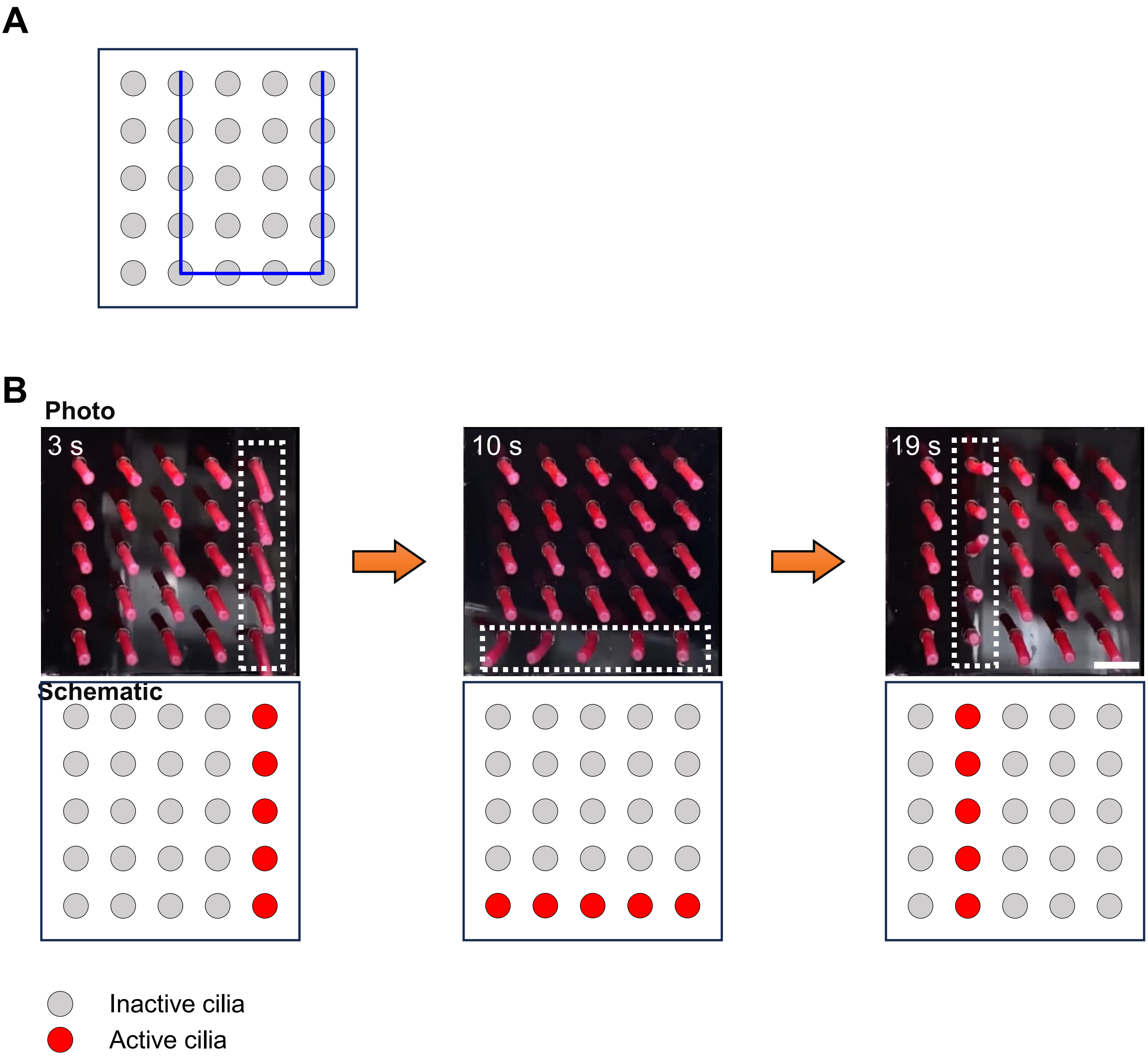

Supplementary Fig. 44. Utilizing targeted cilia activation to potentially achieve path-directed propulsion. (**A**) The desired specific pathway for propulsion (U-shaped pathway). (**B**) To achieve propulsion along a U-shaped pathway, the cilia on the far right column are initially activated, followed by the activation of the cilia on the bottom row. Finally, the cilia on the second column from the left are activated. The cilia array in various states is depicted through snapshots derived from the experimental videos (Supplementary Video 7), directly below which are corresponding schematic diagrams. These diagrams illustrate the activation states of the ciliary array, including the inactive state and active state. Rotating magnetic field: 45mT. Scale bar: 5mm.

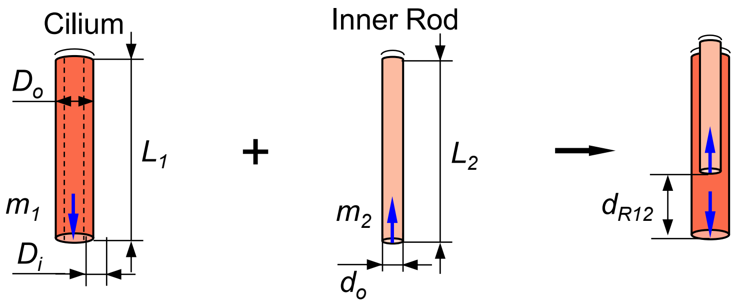

Symbol	Value
D_o	1.4 mm
D_i	0.9 mm
d_o	0.8 mm
L_1	14.5 mm
L_2	20 mm
m_1	4.95×10^{-4} Am^2
m_2	4.95×10^{-4} Am^2

Supplementary Fig. 45. Parameters of cilia involved in ciliary bending amplitude modulation in the "Reprogrammable cilia array" section.

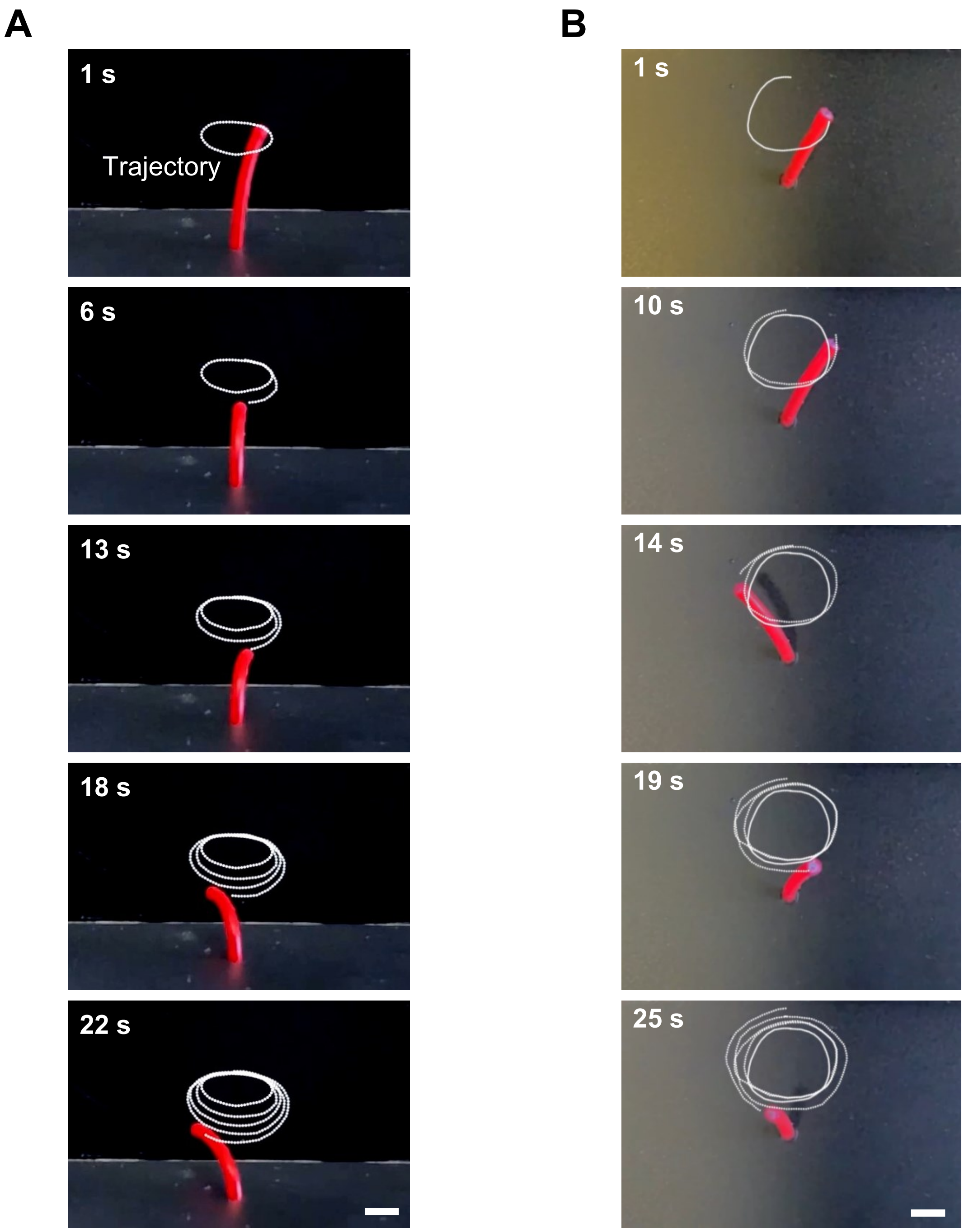

Supplementary Fig. 46. Diagram of various ciliary motion patterns exhibited by cilia at different moments using ciliary bending amplitude modulation under the same rotating magnetic field (45mT). (**A**) Front view. (**B**)Top view. The dashed lines in the diagram represent the trajectory of the midpoint at the end of the cilium. The screenshots above are taken from Supplementary Video 8. Scale bar: 2mm for all panels.

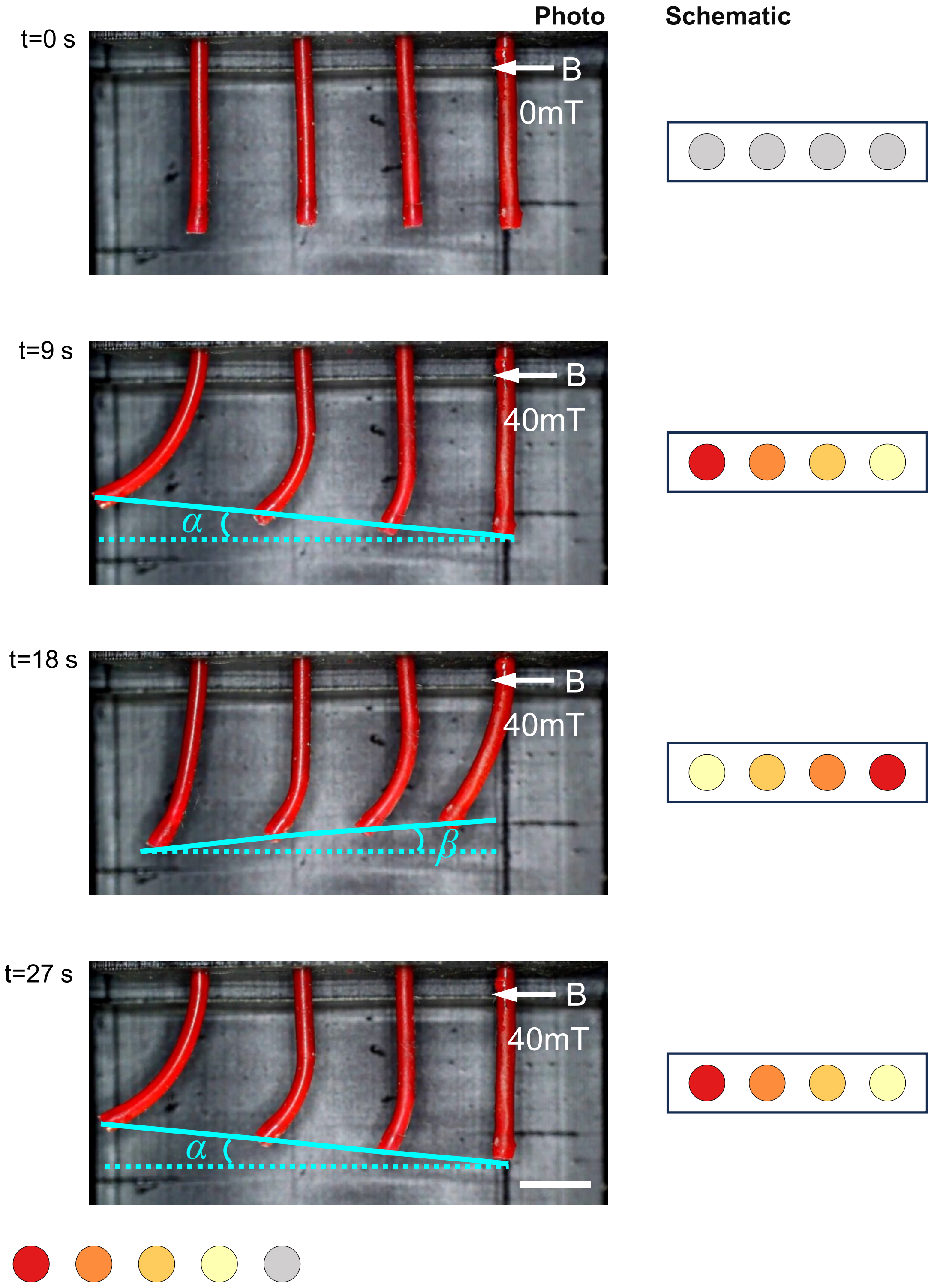

Color intensity indicates the ciliary bending amplitude: lighter to darker shades represent a range from low to high bending amplitude.

Supplementary Fig. 47. Screenshots of ciliary bending amplitude modulation achieved in a 1x4 cilia array under the same uniform magnetic field. The variation in bending amplitude across different cilia results in the tilting angles α and β at the tips of the cilia. To clearly depict the changes in bending amplitude, corresponding schematics are displayed on the right side of the screenshot. The gradient of bending amplitude from left to right is gradual and can also be adjusted as needed to achieve the desired bending amplitudes. The screenshots above are taken from Supplementary Video 8. Scale bar: 5mm.

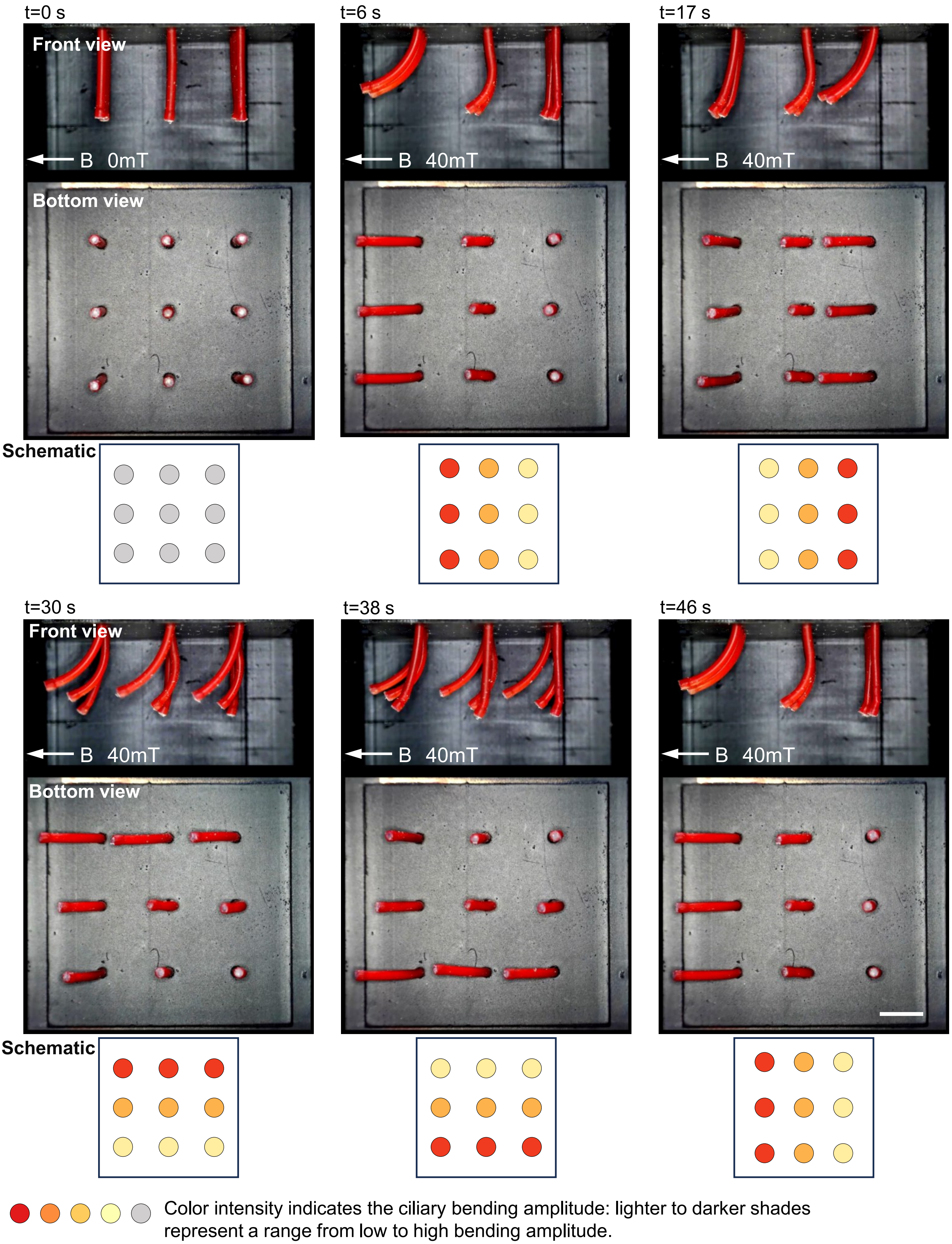

Color intensity indicates the ciliary bending amplitude: lighter to darker shades represent a range from low to high bending amplitude.

Supplementary Fig. 48. Screenshot of ciliary bending amplitude modulation achieved within a 3x3 cilia array under the same uniform magnetic field. To clearly illustrate the variations in bending amplitude, corresponding schematics are displayed at the bottom of the screenshot. The illustration shows some of the achievable patterns, although any pattern that meets specific requirements can be obtained. The screenshots above are taken from Supplementary Video 8. Scale bar: 5mm.

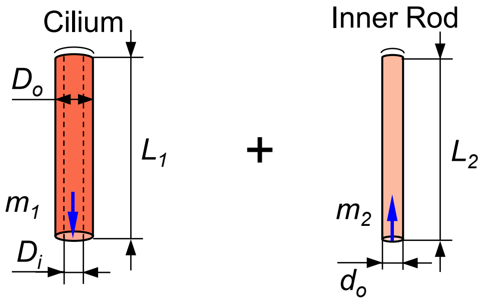

Symbol	Value
D_o	1.4 mm
D_i	0.9 mm
d_o	0.8 mm
L_1	10 mm
L_2	14 mm
m_1	9.9×10^{-4} Am^2
m_2	4.95×10^{-4} Am^2

Supplementary Fig. 49. Parameters of cilia involved in ciliary phase modulation in the "Reprogrammable cilia array" section.

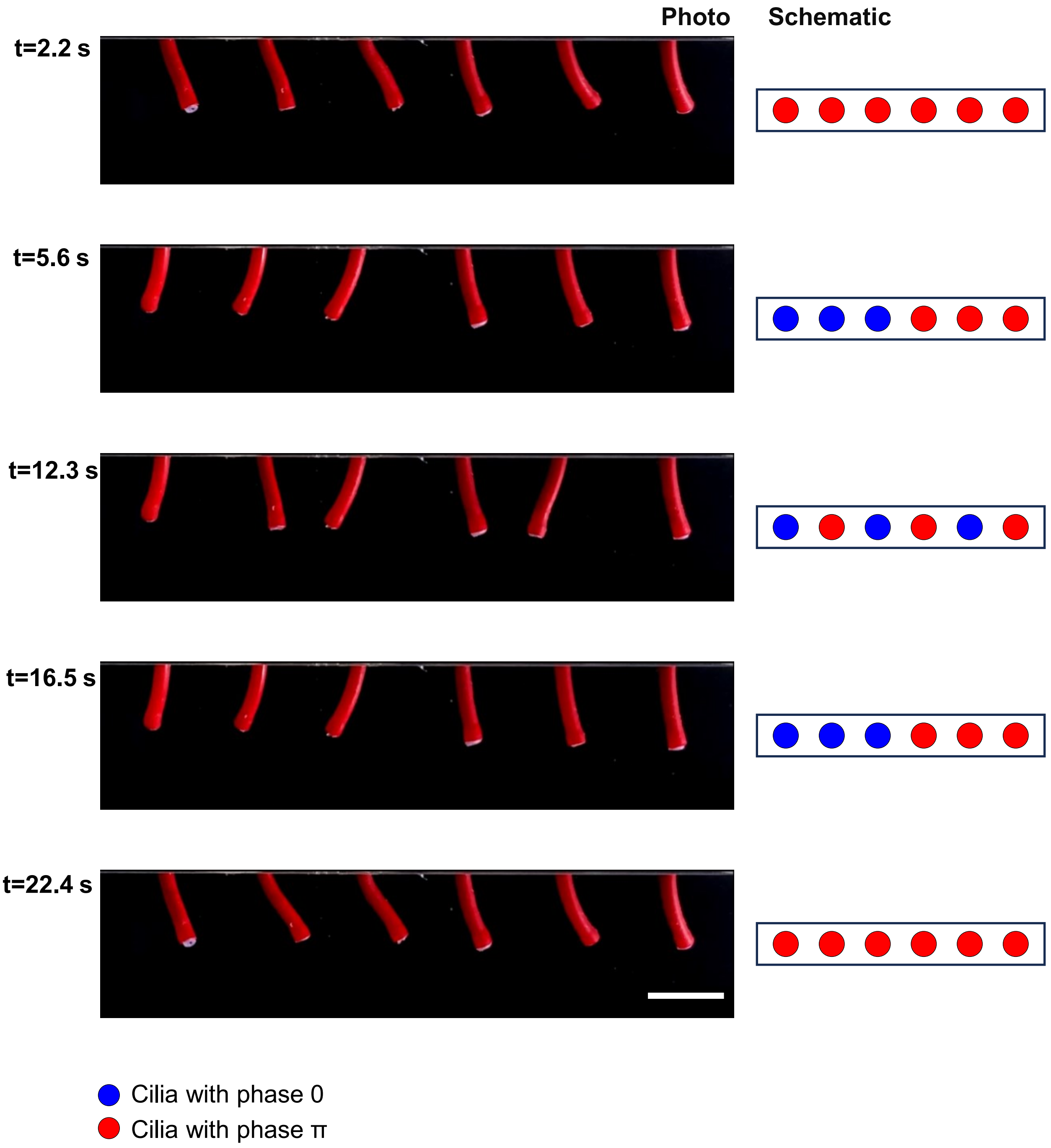

Supplementary Fig. 50. Screenshots of ciliary phase modulation demonstrated using a 1x6 cilia array under the same rotating magnetic field (45mT). To clearly depict the changes in phase, corresponding schematics are displayed on the right side of the screenshots. The screenshots above are taken from Supplementary Video 9. Scale bar: 5mm.

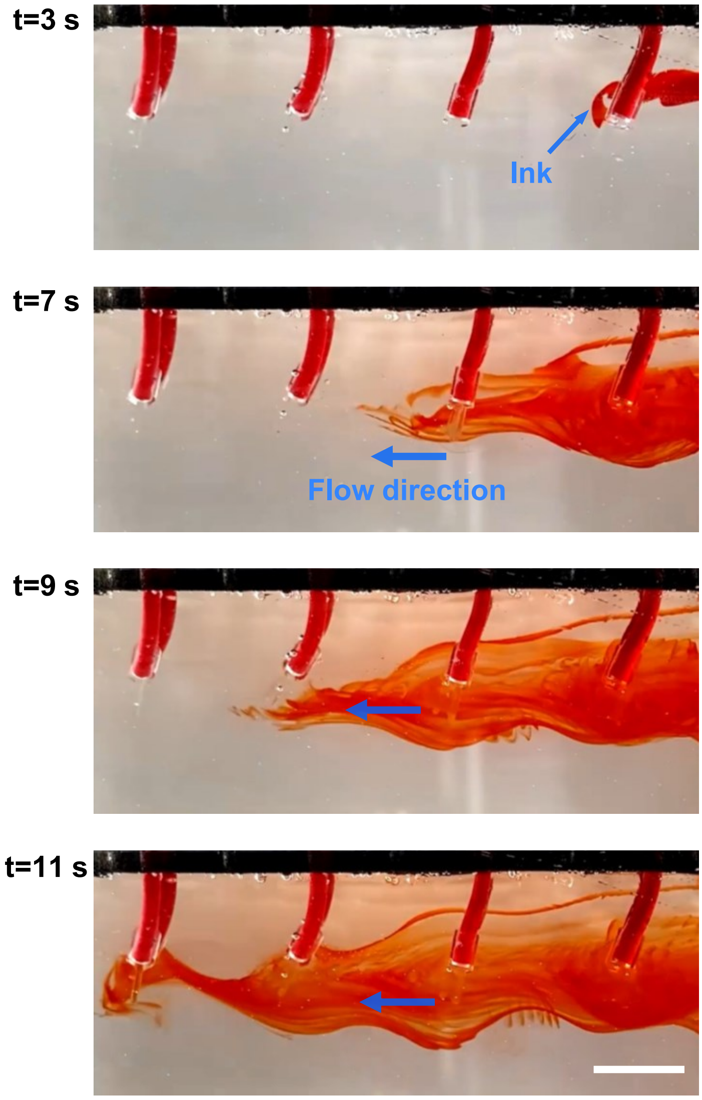

Supplementary Fig. 51. Screenshots of fluid being driven to the left using a 2x4 cilia array under the rotating magnetic field (45mT). All cilia in the array have the same phase. The screenshots above are taken from Supplementary Video 9. Scale bar: 5mm.

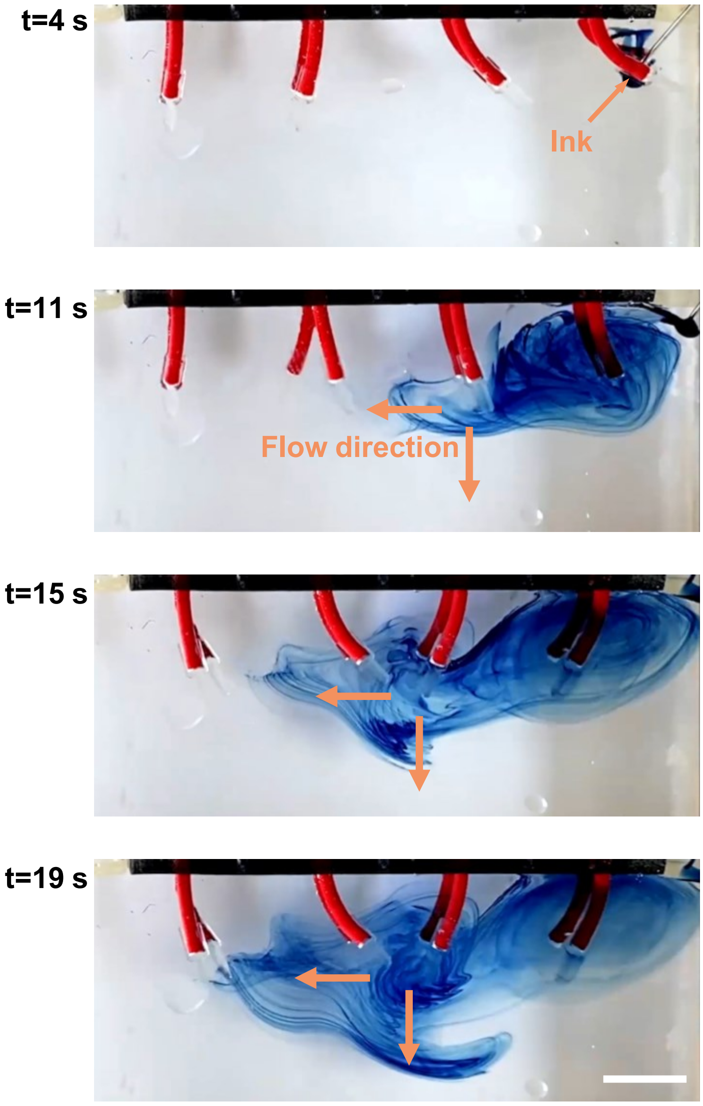

Supplementary Fig. 52. Screenshots of fluid being driven downward and leftward using a 2x4 cilia array under the same rotating magnetic field (45mT). The left two columns of cilia have a phase difference of π compared to the right two columns of cilia. The screenshots above are taken from Supplementary Video 9. Scale bar: 5mm.

A

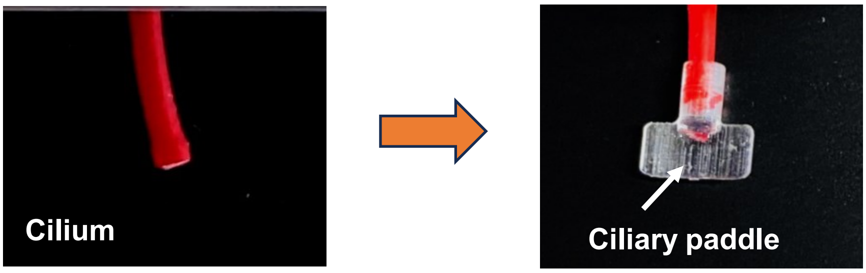

B

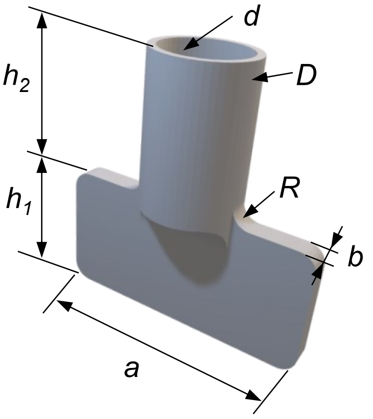

Symbol	Value
a	7.2 mm
b	0.6 mm
h_1	3.7 mm
h_2	4 mm
D	2.5 mm
d	1.6 mm
R	0.5 mm

Supplementary Fig. 53. Detail diagram of a cilium with a paddle at its tip. (**A**) Diagram of a cilium and a cilium with a paddle installed at its tip. To facilitate observation of the cilia's effect on fluid dynamics, paddles have been installed at the tips of the cilia. These paddles enhance the driving effect of the cilia without altering their operating mode. (**B**) Design parameters of the paddle.

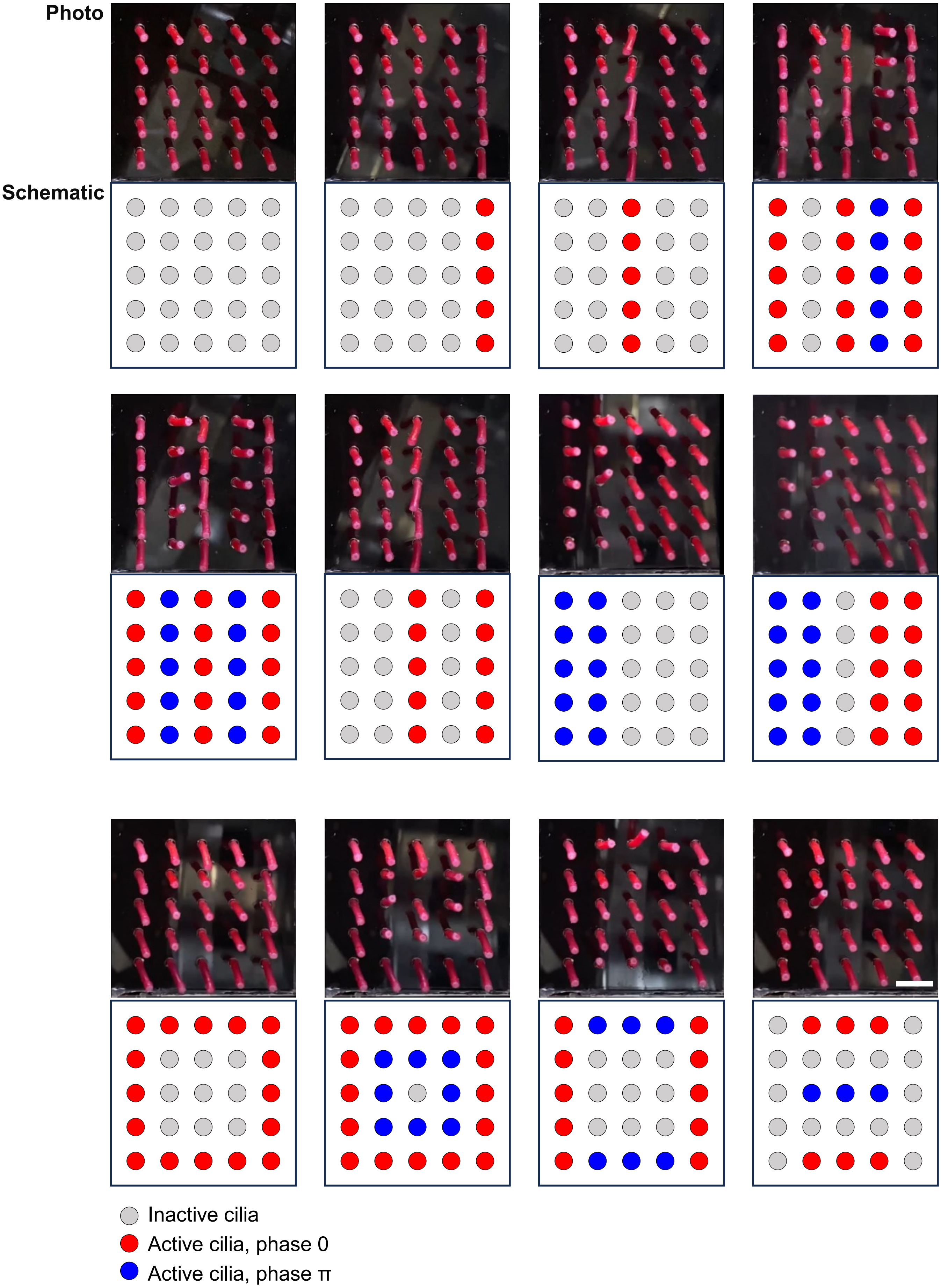

Supplementary Fig. 54. Demonstration of the driving combining targeted cilia activation and ciliary phase modulation. The cilia array in various states is depicted through snapshots derived from the experimental videos (Supplementary Video 7), directly below which are corresponding schematic diagrams. These diagrams illustrate the activation states of the ciliary array, including the inactive state, active state with phase 0, and active state with phase π. These diverse patterns demonstrate the capability to achieve more complex driving patterns through the combination of targeted cilia activation and ciliary phase modulation. Scale bar: 5mm.

A

B

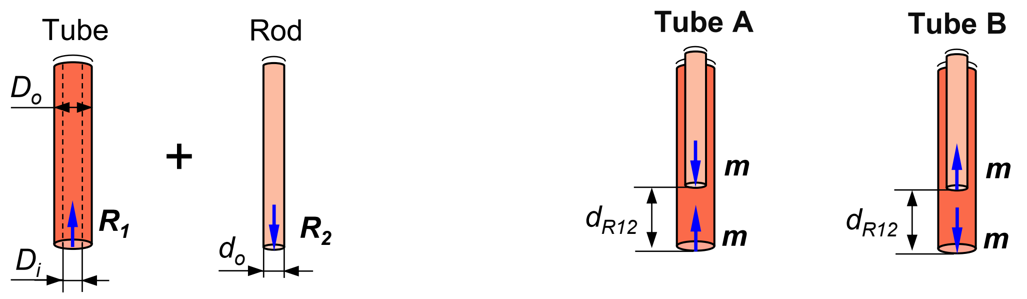

C

Symbol	Value
D_o	2.6 mm
D_i	1.7 mm
d_o	1.4 mm
m	1.02×10^{-3} Am^2

Supplementary Fig. 55. Parameters of soft tubes used in the "Coordinated multi-instrument operation" section. (**A**) Diagram of the involved tubes and rods. (**B**) Two types of integrated tubes. (**C**) Design parameters corresponding to (A-B) above.

A   B

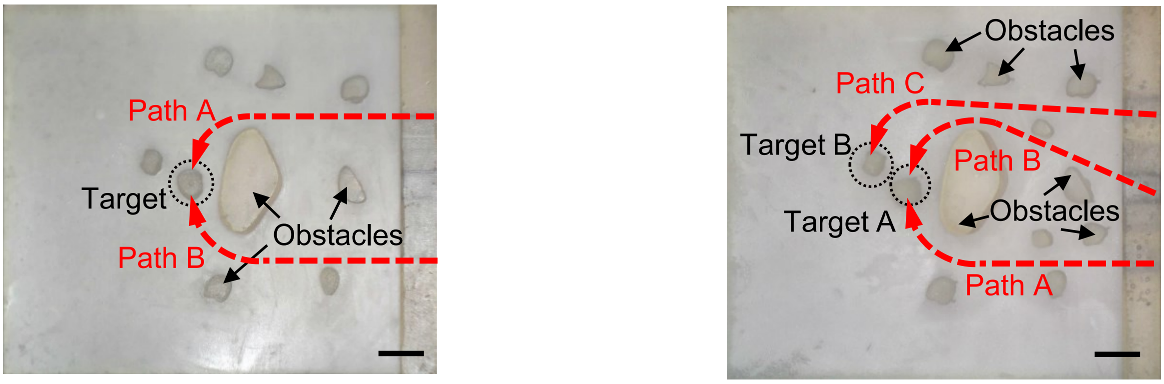

Supplementary Fig. 56. Illustration of the task setup for the application scenario in the "Coordinated multi-instrument operation" section. (**A**) The first application scenario. (**B**) The second application scenario. Scale bar: 5mm for all panels.

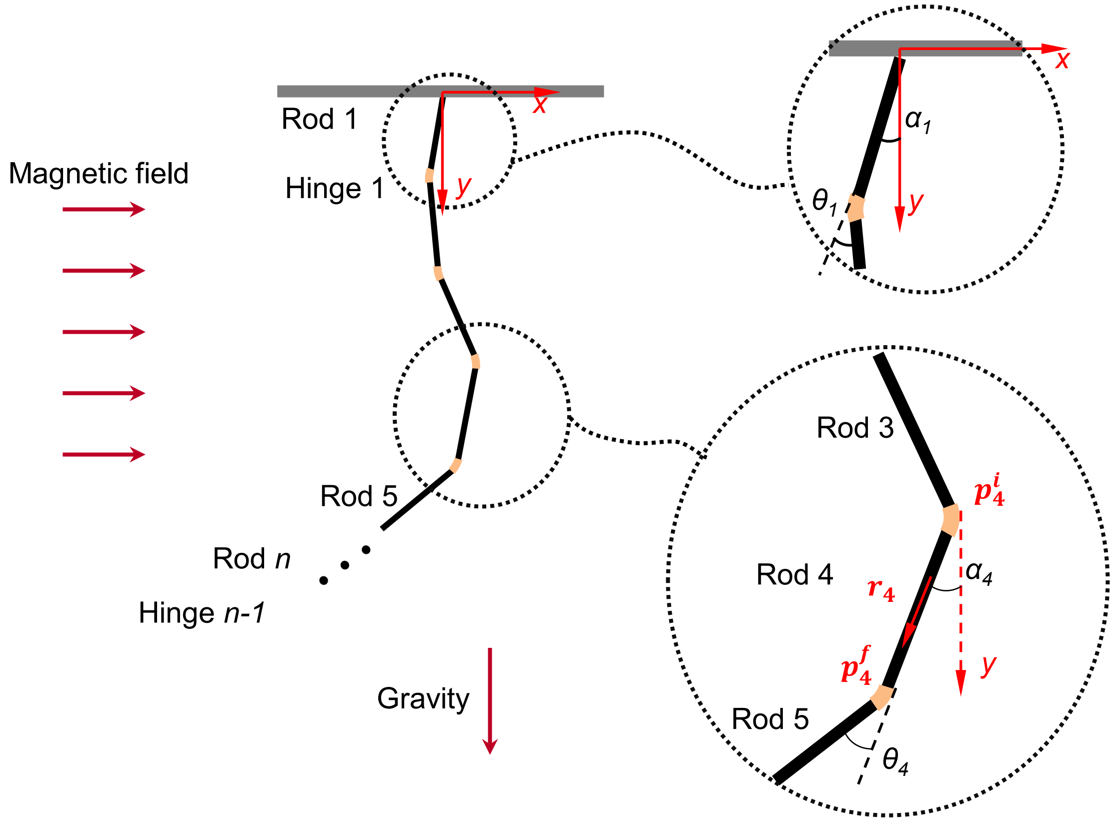

Supplementary Fig. 57. Analysis diagram of soft tube/rod modeling. Based on the pseudo-rigid body model, the soft tube is equivalently represented as a series of connected rods and hinges. This model is vertically suspended, with its top end fixed, and is subjected to the combined effects of a magnetic field and gravity.

A

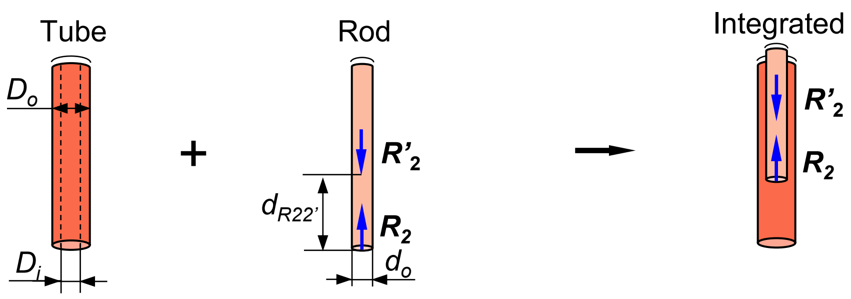

B

Symbol	Value
D_o	2.6 mm
D_i	1.7 mm
d_o	1.4 mm
m	1.02×10^{-3} Am^2
$d_{R22'}$	6 mm, 12 mm, 18 mm, 24 mm
Material	PDMS 3:1, PDMS 5:1, PDMS 10:1, PDMS 15:1
Magnetic field	30 mT, 60 mT, 90 mT, 120 mT, 150 mT

C

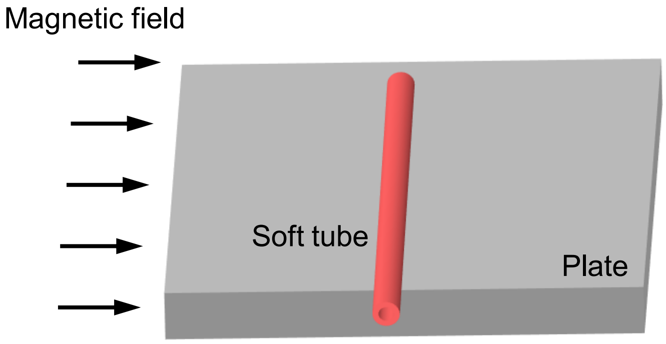

Supplementary Fig. 58. Experimental setup diagram for analysis of soft tube deformation. (**A**) Configuration diagram of the soft tube used in the experiment. (**B**) Manufacturing and experimental parameters for the soft tube involved in experiments. (**C**) Diagram illustrating the placement of the soft tube relative to the direction of the applied magnetic field.

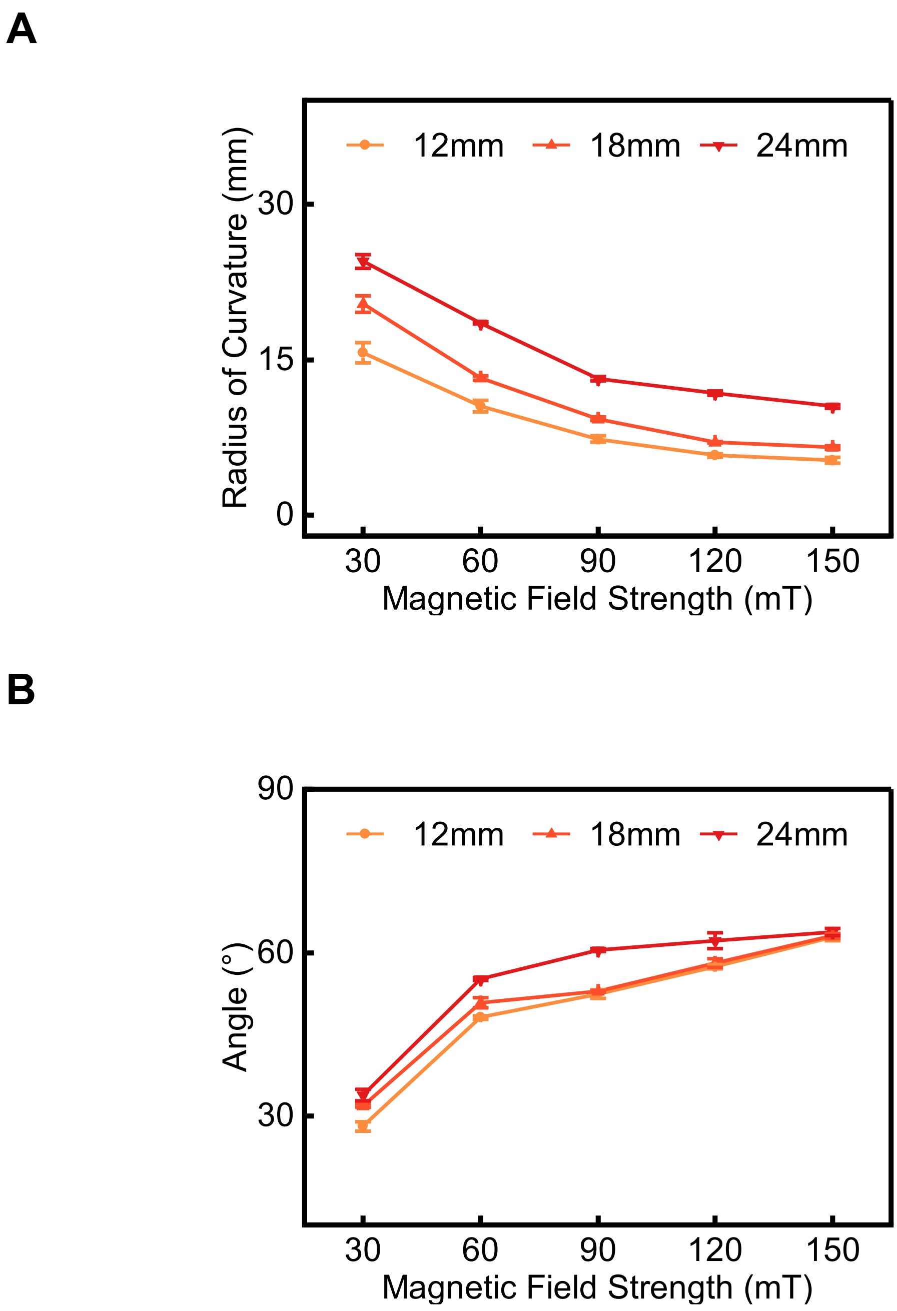

Supplementary Fig. 59. The impact of magnetic field strength on the deformation of soft tubes. (**A**) Radius of curvature. (**B**) Terminal bending angle. The radius of curvature and terminal bending angle are used to describe the deformation of soft tubes. For specific definitions, see Supplementary Fig. 29. Data are presented as mean ± SD (*n*=3 tests).

A

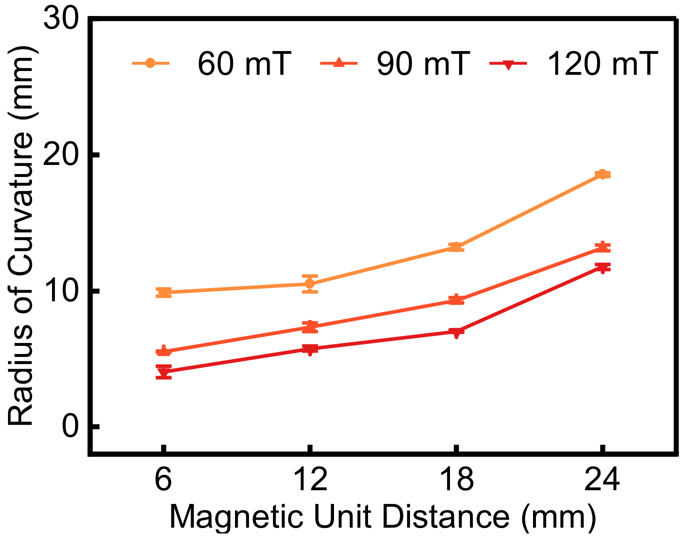

B

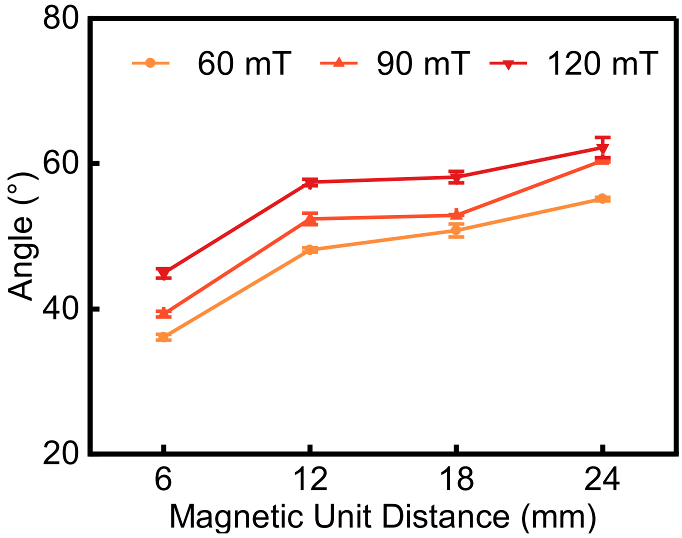

Supplementary Fig. 60. The impact of magnetic unit distance on the deformation of soft tubes. (**A**) Radius of curvature. (**B**) Terminal bending angle. The radius of curvature and terminal bending angle are used to describe the deformation of soft tubes. For specific definitions, see Supplementary Fig. 29. Data are presented as mean ± SD (*n*=3 tests).

A

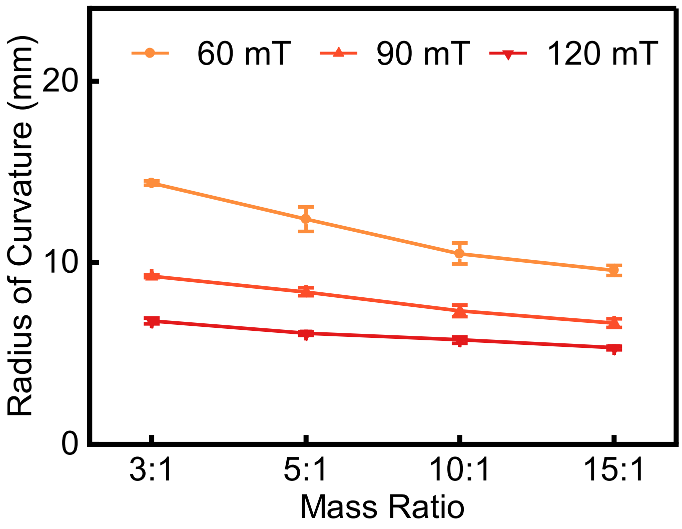

B

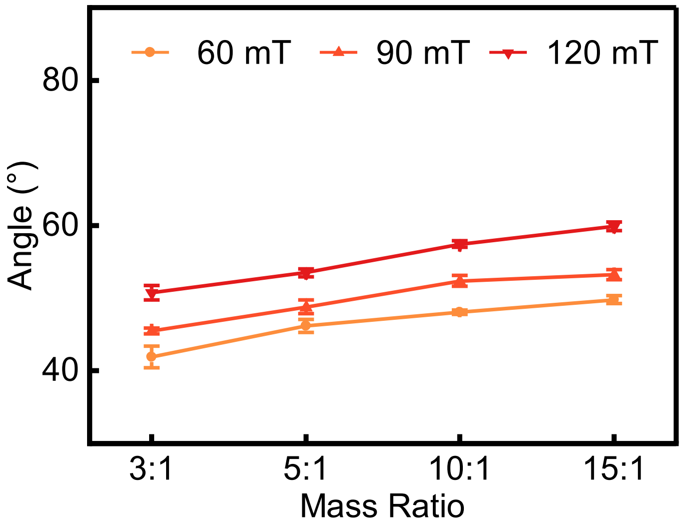

Supplementary Fig. 61. The impact of elastic modulus on the deformation of soft tubes. (**A**) Radius of curvature. (**B**) Terminal bending angle. We utilized Polydimethylsiloxane (PDMS) as the manufacturing material and varied the base-to-curing agent mass ratio at 3:1, 5:1, 10:1, and 15:1 (i.e., values of the *x*-axis above) to achieve soft tubes with different elastic moduli. The radius of curvature and terminal bending angle are used to describe the deformation of soft tubes. For specific definitions, see Supplementary Fig. 29. Data are presented as mean ± SD (*n*=3 tests).

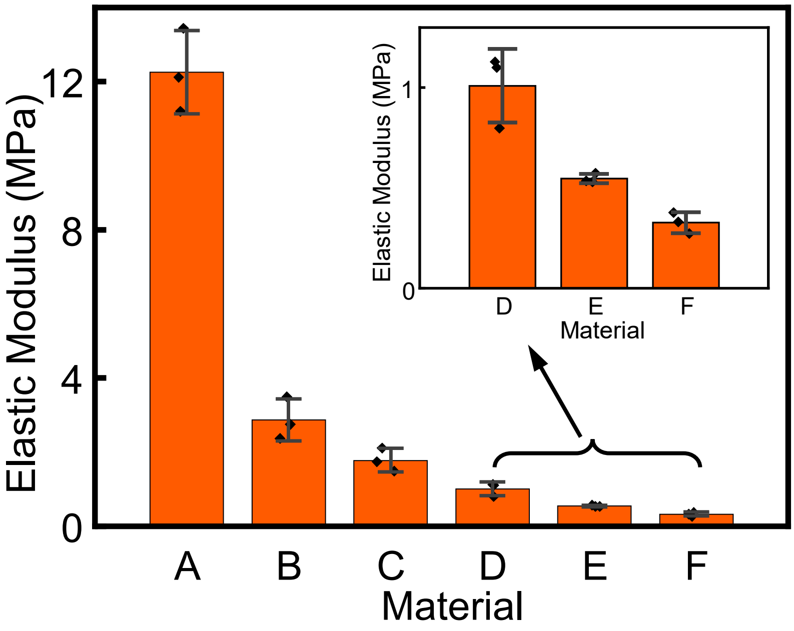

Symbol	Materials
A	PDMS with a base-to-curing agent ratio (B:C ratio) of 10:1 + Magnetic particle m_{PDMS} : $m_{Magnetic\ particle}$=3:1
B	PDMS, B:C ratio 3:1
C	PDMS, B:C ratio 5:1
D	PDMS, B:C ratio 10:1
E	PDMS, B:C ratio 15:1
F	PDMS, B:C ratio 20:1

Supplementary Fig. 62. Elastic modulus of different materials. PDMS: polydimethylsiloxane. Tensile tests were conducted using a Universal Testing System (Model 5942, INSTRON, USA). The tensile test for each ratio was repeated three times to calculate the mean and standard deviation (*n*=3 tests). Bars represent mean values; error bars denote the standard deviation.

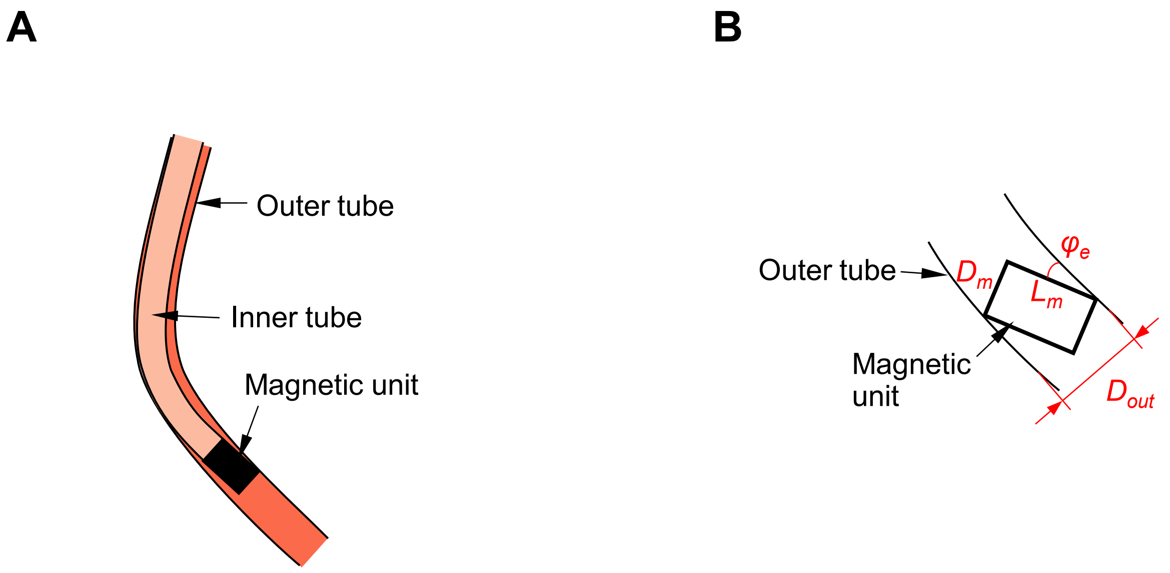

Supplementary Fig. 63. Diagram for error analysis of magnetic unit position control. (**A**) Diagram describing the position of the inner tube within the outer tube. (**B**) Illustration of the position of the magnetic unit in the outer tube under extreme conditions. This scenario is analyzed to calculate the angle error of the magnetic unit caused by the gap between the outer tube and the inner tube in extreme cases.

A

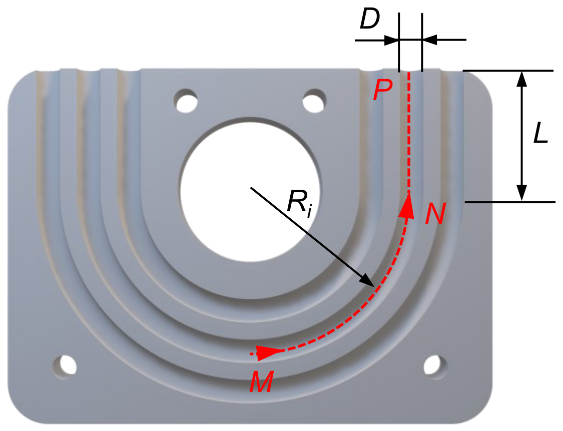

Symbol	Value
R_i	2 mm, 5 mm, 10 mm, 15 mm, 20 mm, ∞
L	15 mm
D	2.9 mm

B

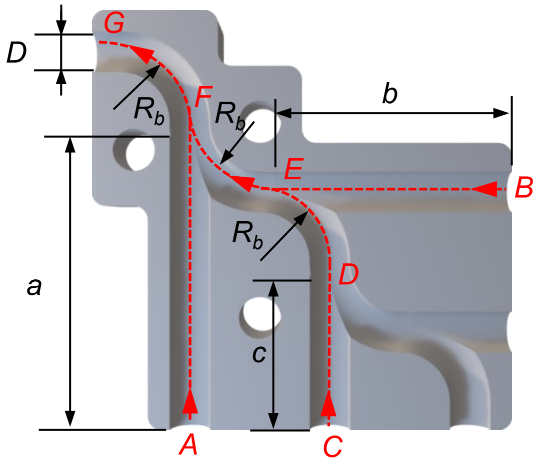

Symbol	Value
R_b	10 mm
a	22 mm
b	18 mm
c	12 mm
D	2.9 mm

Supplementary Fig. 64. Experimental setup for assessing parameters influencing inner tube operation. (**A**) Setup for experiments assessing the impact of the radius of curvature. (**B**) Setup for experiments evaluating the effect of the number of bends.

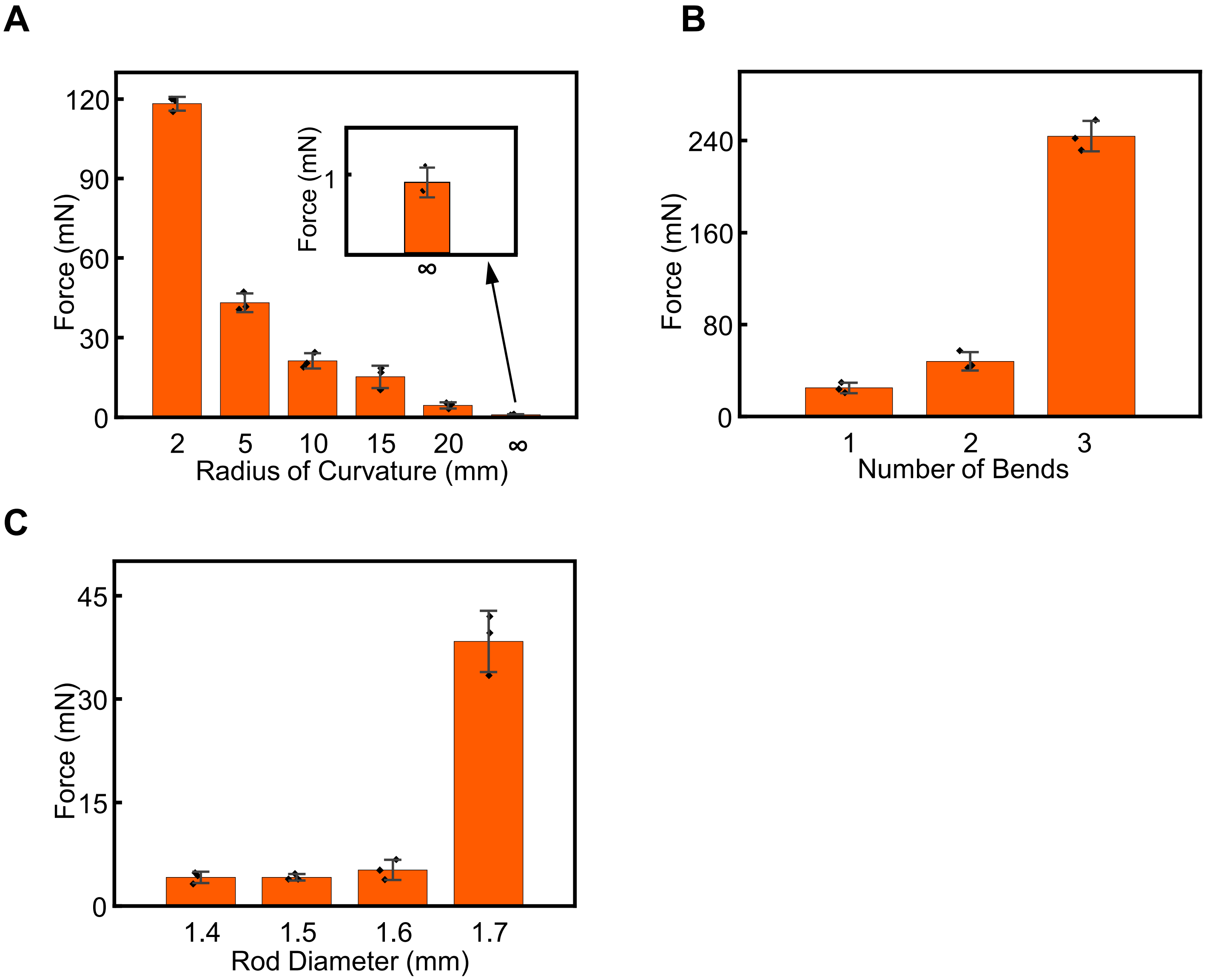

Supplementary Fig. 65. Effect of various parameters on the relative motion resistance involved in manipulating tubes. (**A**) Different radii of curvature. (**B**) Different numbers of bends. (**C**) Different rod diameters. Each type of operation was repeated 3 times (*n*=3 tests). Bars represent mean values; error bars denote the standard deviation.

A

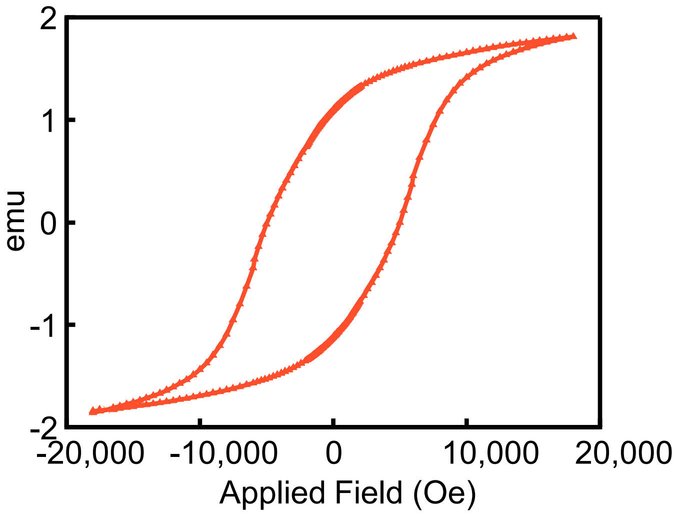

B

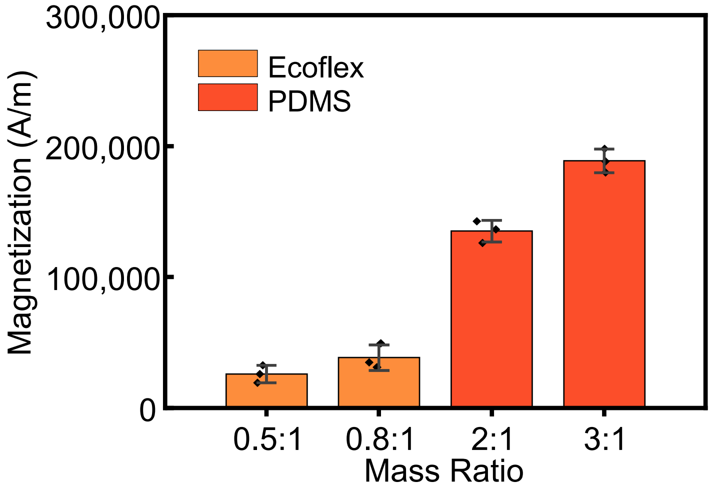

Supplementary Fig. 66. Magnetic characterization of different materials. (**A**) Magnetic hysteresis characteristics. This diagram is continuously obtained by a vibrating sample magnetometer (VSM) under varying magnetic fields. The sample is made from Ecoflex 50, mixed with magnetic particles, having a mass ratio of magnetic particles to Ecoflex 50 at 0.5:1. The volume of this sample is 38.2 mm³. This diagram facilitates the determination of the sample's remanence, thereby providing a foundation for calculating the magnetization magnitude in the subsequent figure. (**B**) Magnetization magnitude. The mass ratios represented here are the mass of the magnetic particle to the mass of the soft material (namely Ecoflex and PDMS). For these measurements, Ecoflex refers to the Ecoflex 50 series, and the mass ratio of the PDMS base-to-curing agent is 10:1. Each data point is derived from a singular measurement across three samples (n=3 tests). Bars represent mean values; error bars denote the standard deviation.

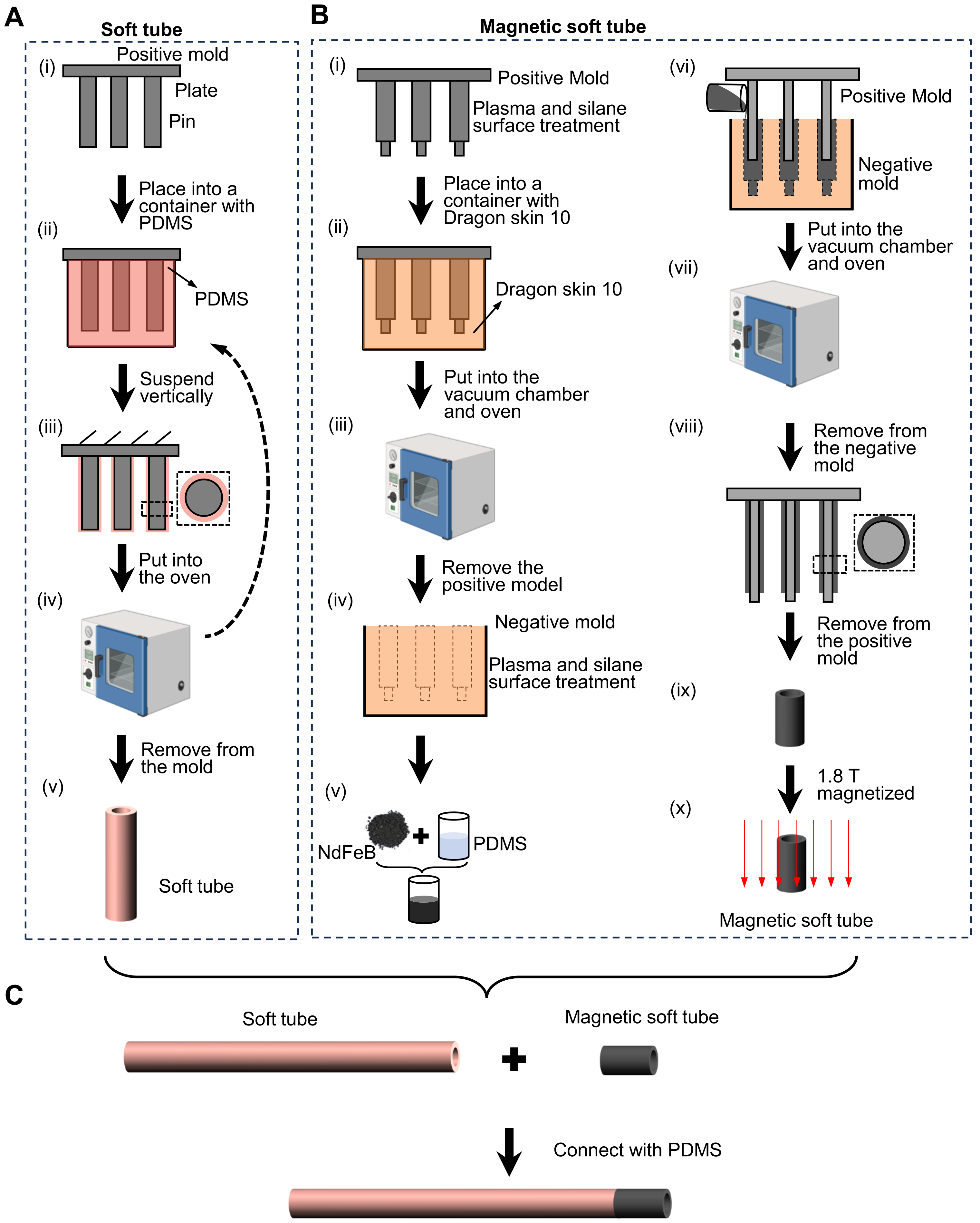

Supplementary Fig. 67. Fabrication process of tubes. (**A**) Fabrication process for soft tubes. PDMS: Polydimethylsiloxane. In this process, the mass ratio of base to curing agent in PDMS can be adjusted according to requirements. Steps (ii) to (iv) can be repeated to achieve soft tubes with varying wall thicknesses. (**B**) Fabrication process for magnetic soft tubes. NdFeB: Neodymium Iron Boron. (**C**) Soft tubes and magnetic soft tubes can be connected together using PDMS. After connection, the assembly requires curing in an oven to solidify the PDMS. The oven icons above are provided by BioRender.com and used with permission [75].

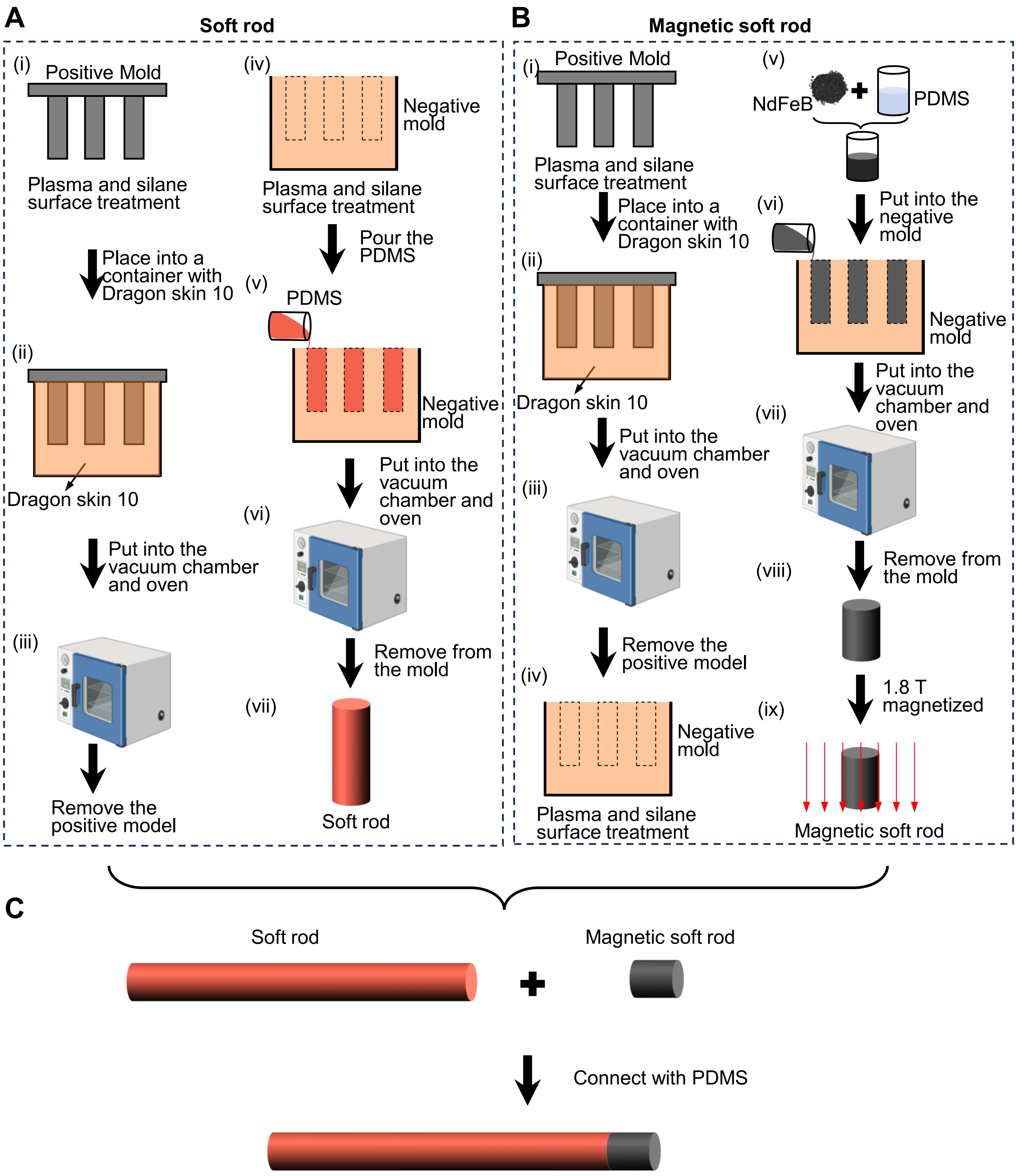

Supplementary Fig. 68. Fabrication process of rods. (**A**) Fabrication process for soft rods. PDMS: Polydimethylsiloxane. In this process, the mass ratio of base to curing agent in PDMS can be adjusted according to requirements. (**B**) Fabrication process for magnetic soft rods. NdFeB: Neodymium Iron Boron. (**C**) Soft rods and magnetic soft rods can be connected together using PDMS. After connection, the assembly requires curing in an oven to solidify the PDMS. The oven icons above are provided by BioRender.com and used with permission [75].

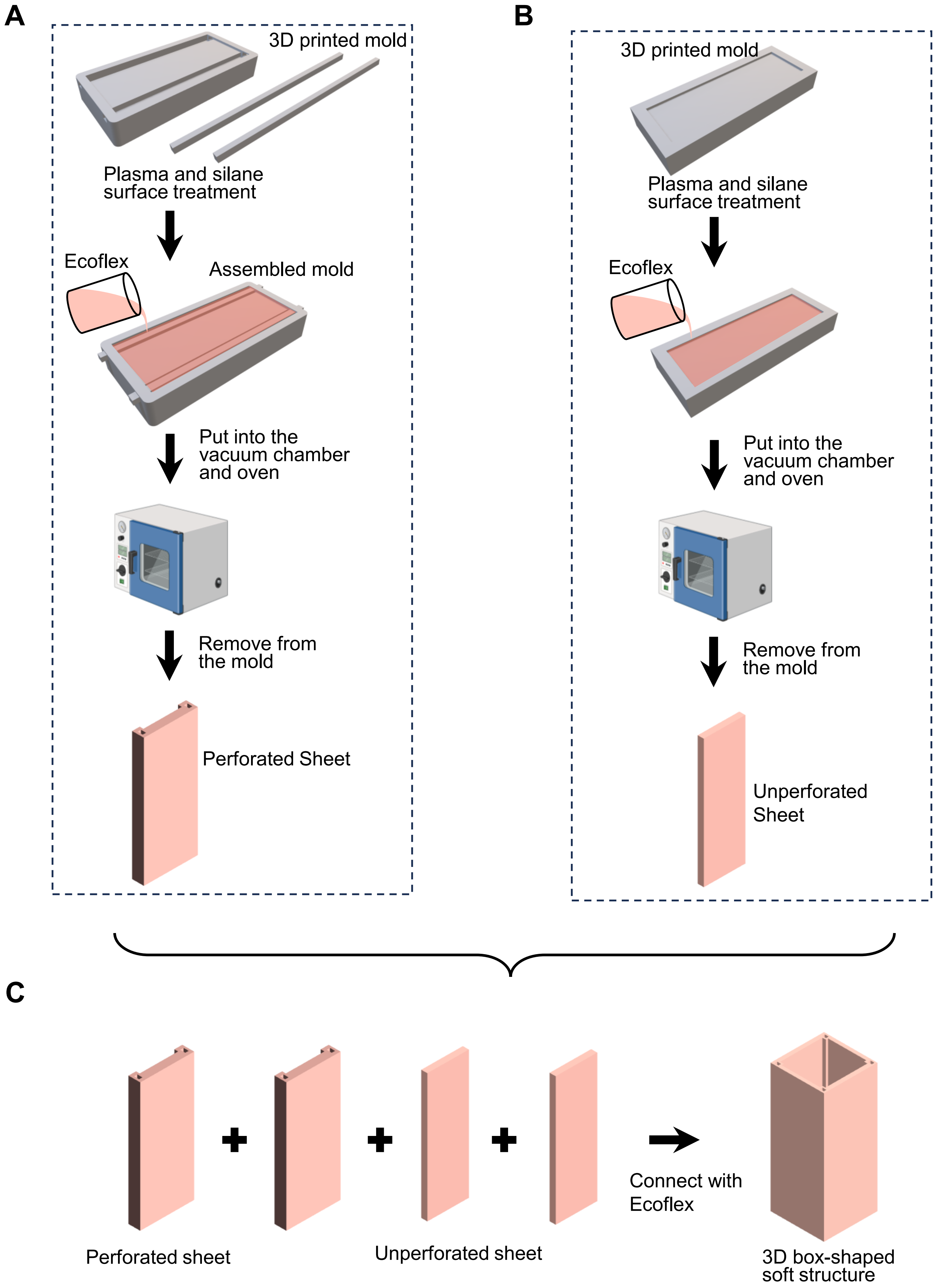

Supplementary Fig. 69. Fabrication process of 2D sheets and 3D box-shaped soft structures. (**A**) Fabrication process for perforated sheet. (**B**) Fabrication process for unperforated sheet. (**C**) Fabrication process for 3D box-shaped soft structure. The oven icons above are provided by BioRender.com and used with permission [75].

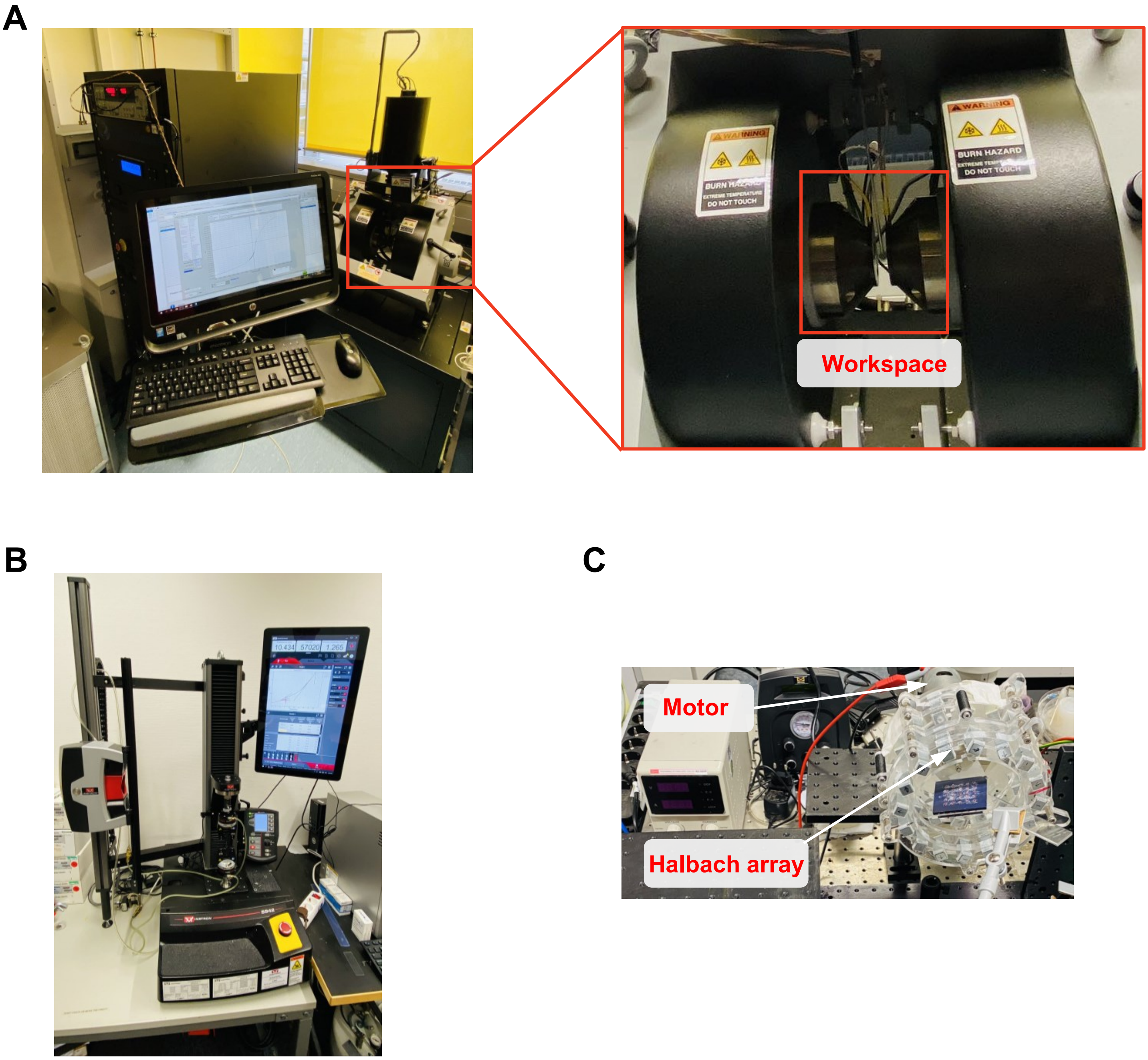

Supplementary Fig. 70. Experimental platforms. (**A**) Vibrating sample magnetometer: utilized for magnetizing materials and generating uniform magnetic fields for control of soft robots. (**B**) Universal testing system: employed for conducting tensile strength tests and measuring friction forces. (**C**) Halbach array: designed to generate rotating magnetic fields.

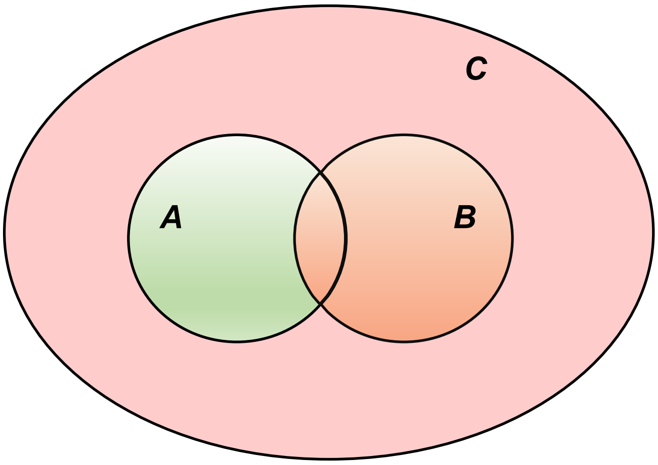

A: Existing method (only changing the external magnetic field)
B: Proposed method + Static magnetic field
C: Proposed method + Tunable magnetic field

Supplementary Fig. 71. Force-torque space generated by different methods. We theoretically compared the force-torque spaces generated by existing methods, the proposed method under a static magnetic field, and the proposed method under a tunable magnetic field. Detailed theoretical derivations are provided in Supplementary Note 8.

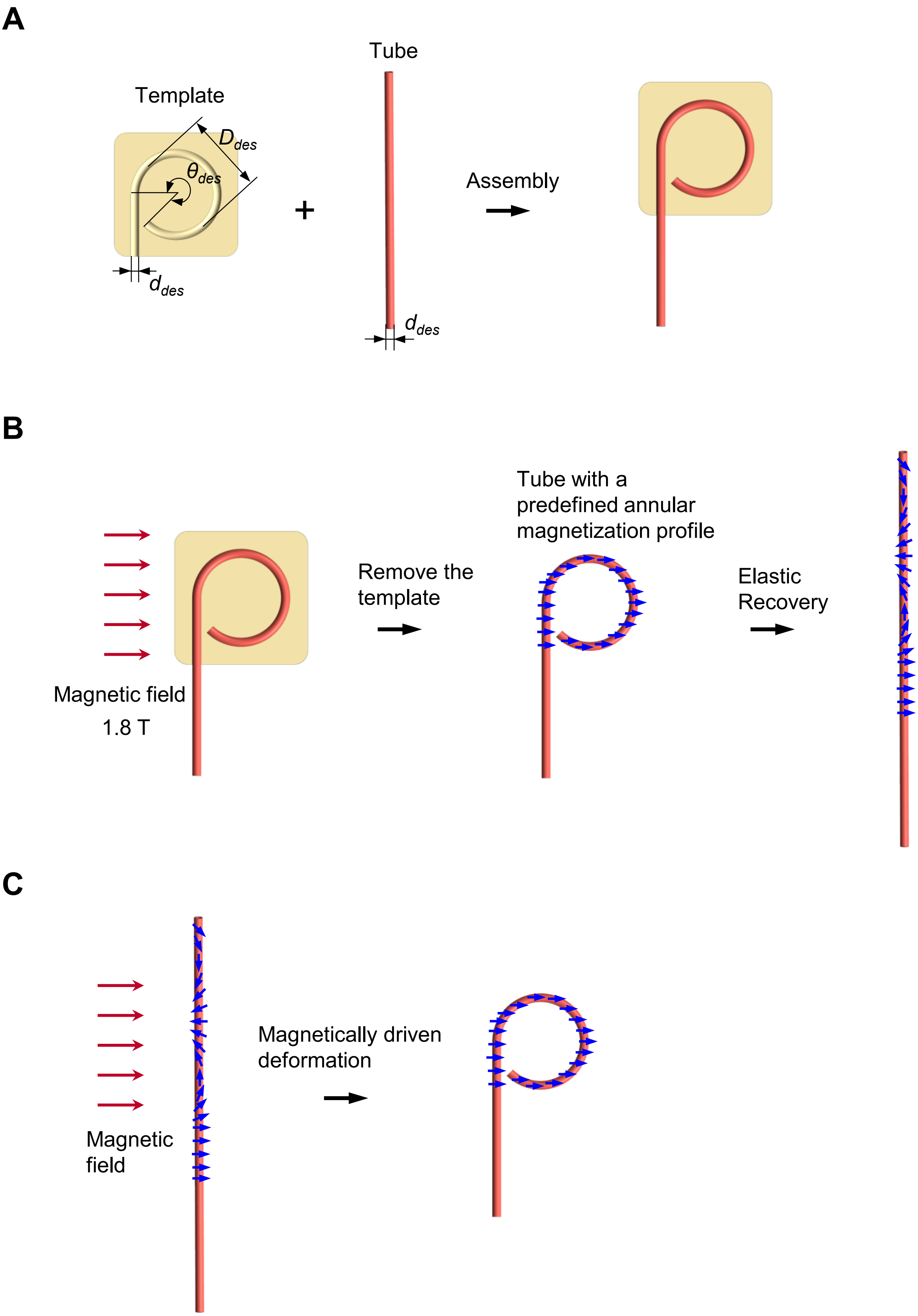

Supplementary Fig. 72. Template-based magnetization profile design method. (**A**) According to the requirements, templates and tubes with specific parameters are fabricated, and the tube is mounted onto the template to fix its shape. (**B**) The tube assembled with the template is then placed in a strong magnetic field for magnetization; after magnetization, the template is removed to yield a tube with a specific magnetization profile. (**C**) The tube with the specific magnetization profile is placed in a uniform magnetic field, where it deforms in a manner similar to the template.

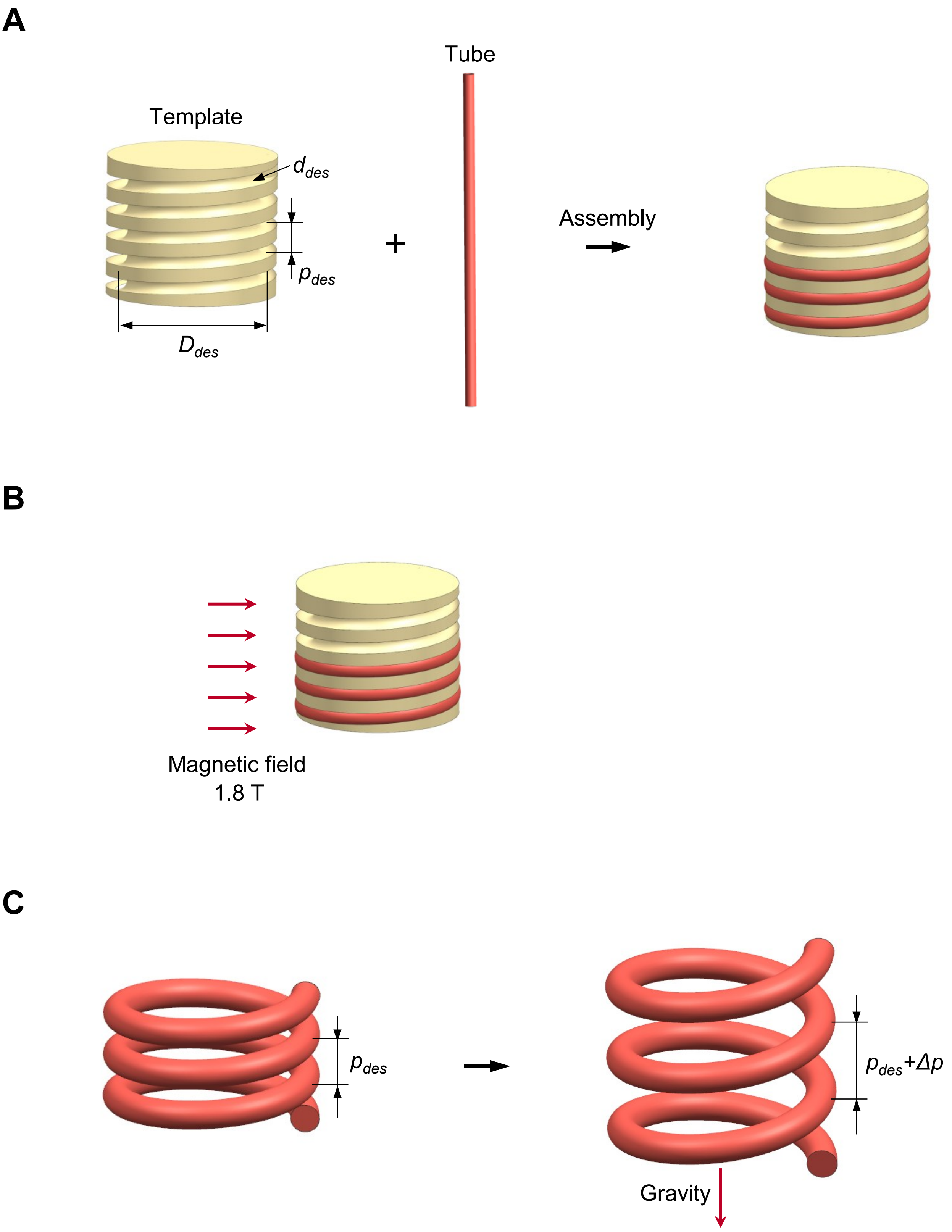

Supplementary Fig. 73. Magnetization and analysis of helical tubes. (**A**) The helical magnetization profile of the tube is obtained using a template. A template and tube with specific parameters are first fabricated, and the tube is wound and mounted onto the template. (**B**) The tube, assembled on the template, is then placed in a strong magnetic field for magnetization to achieve the desired magnetization profile. (**C**) Under the influence of gravity, the helical tube exhibits an increase in pitch.

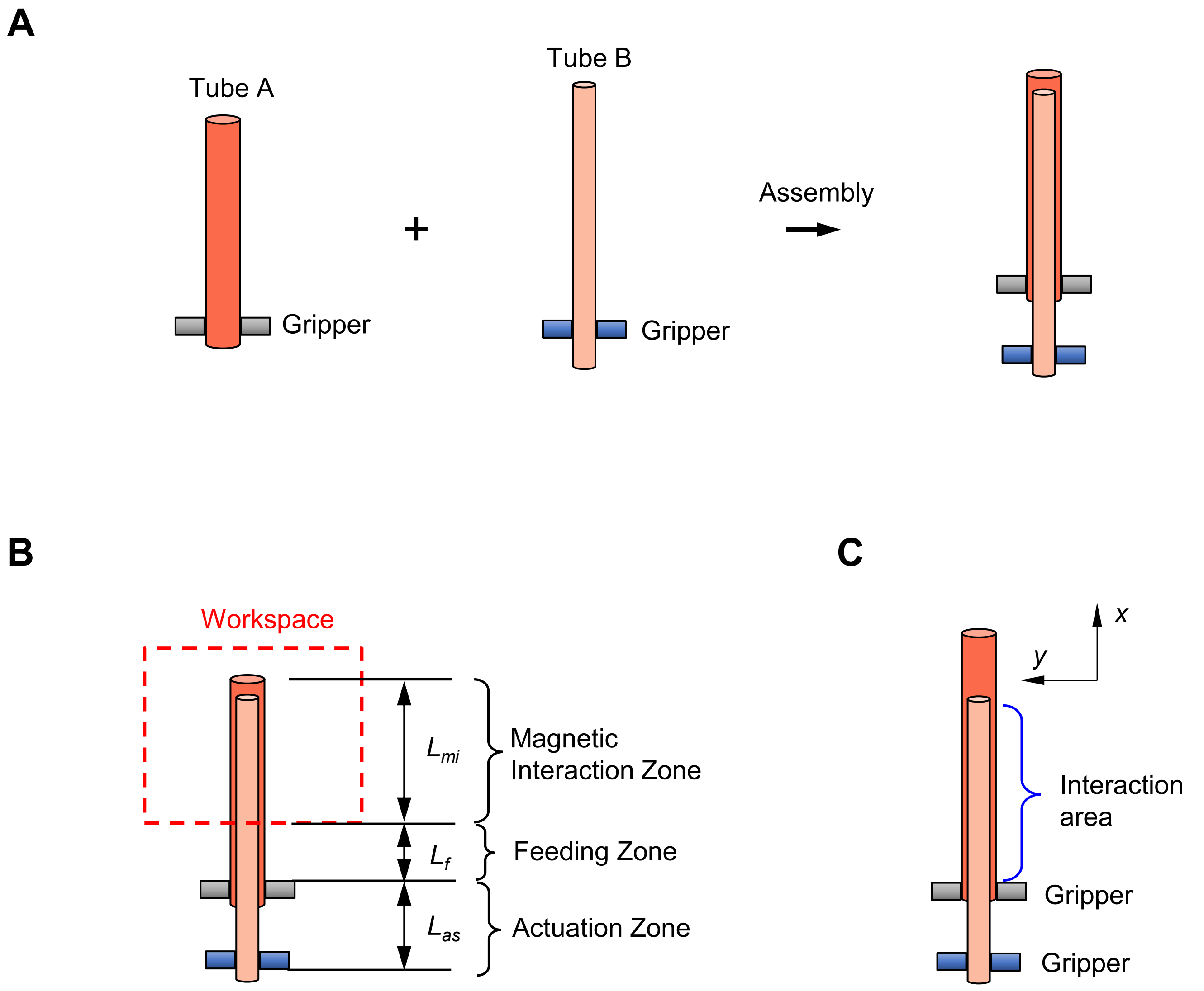

Supplementary Fig. 74. Operation of tubes and their working areas. (**A**) Each tube is manipulated by a gripper at its end. Two tubes are assembled together, and the gripper manipulation enables relative movement between them. (**B**) The operation zone is divided based on the tubes' functions into the Magnetic Interaction Zone, Feeding Zone, and Actuation Zone. (**C**) The interaction area between the two tubes involves both tensile and compressive forces.

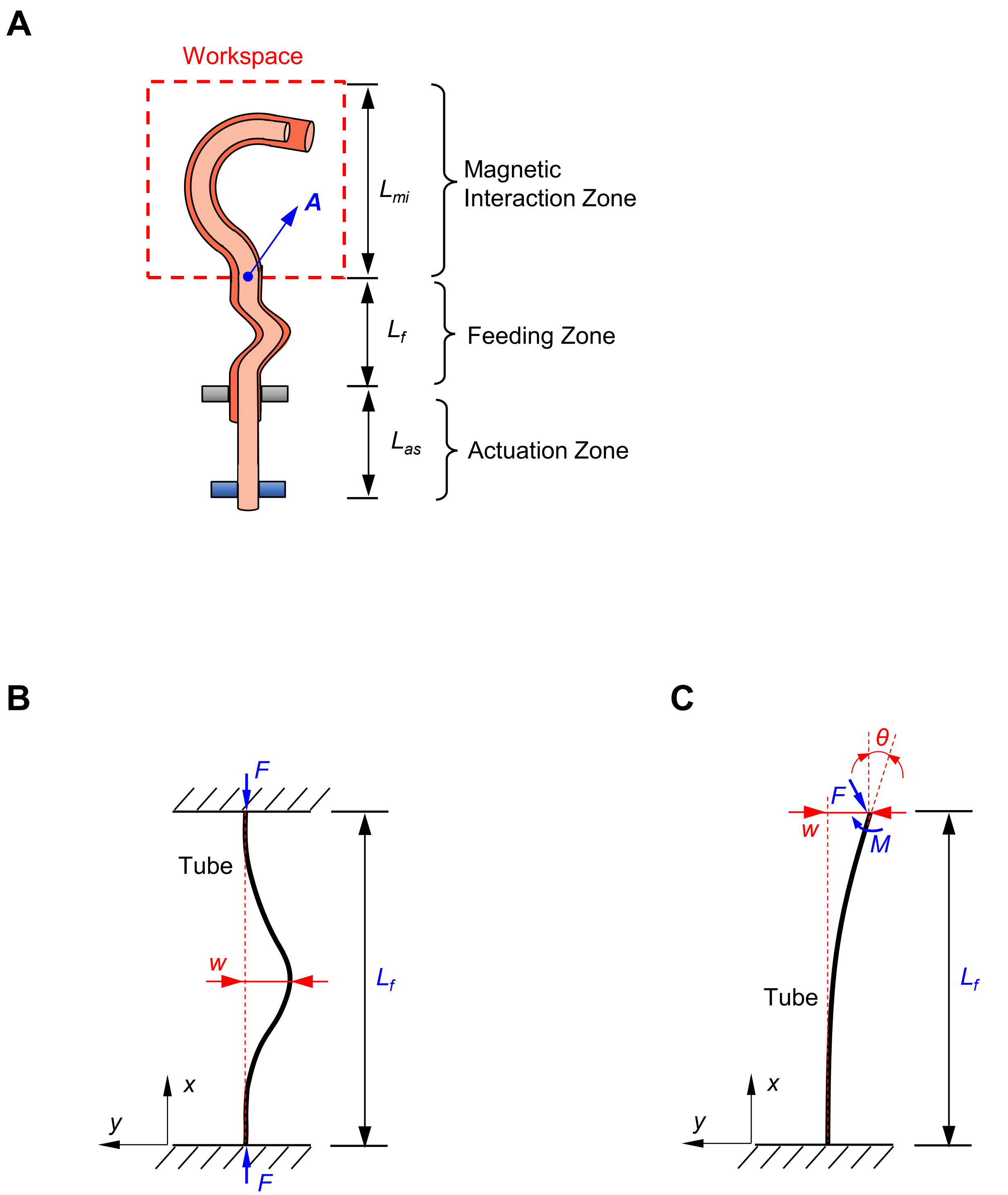

Supplementary Fig. 75. Force analysis of the tube in the Feeding Zone. (**A**) The tube in the Feeding Zone undergoes bending deformation under applied forces. Point A is located at the boundary (interface) between the Feeding Zone and the Magnetic Interaction Zone. (**B**) Compressive rod stability model. In this model, both ends of the tube are fixed, and it exhibits bending deformation under compression. This model is employed to analyze the stability issues of the tube under compression during the relative movement between two tubes. (**C**) Cantilever beam model. One end of the beam is fixed, while the other end is subjected to both a force and a moment, leading to deflection. This model is used to analyze the deformation behavior of two nested tubes under load within the Feeding Zone.

A

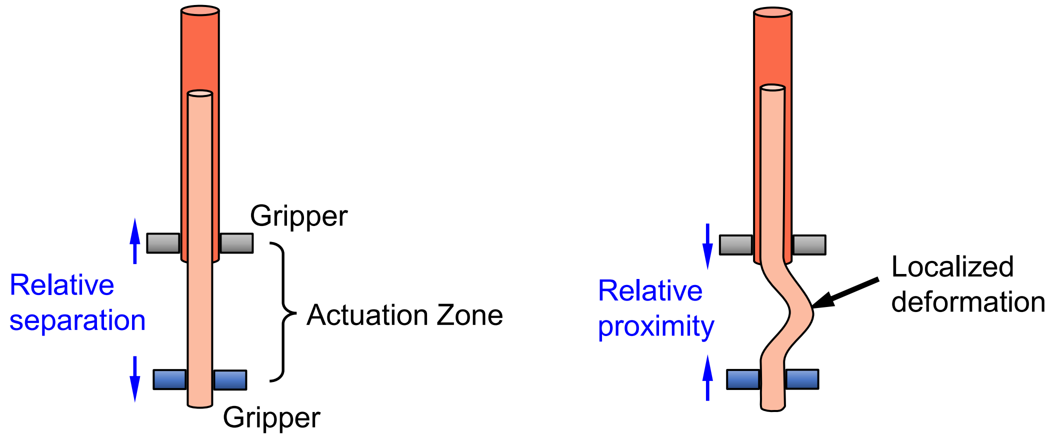

B

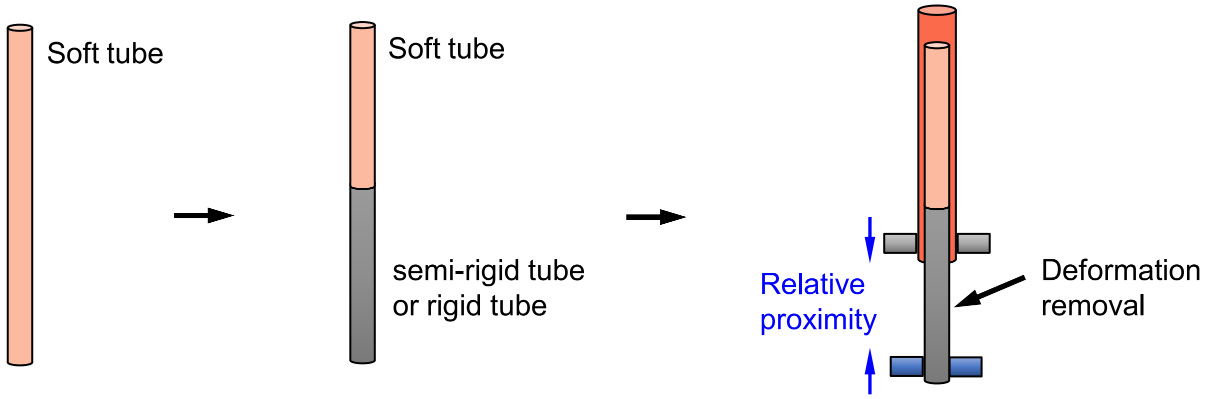

Supplementary Fig. 76. Deformation of the tube in the Actuation Zone and proposed solution. (**A**) Two grippers independently control the two tubes, facilitating their relative motion, namely relative separation and relative proximity. Due to the inherent characteristics of the tensile and compressive members, the tube is prone to bending deformation during relative proximity. (**B**) To mitigate this issue, the inner tube is designed in segments with varying stiffness. The portion of the tube that is required to deform under the magnetic field retains the soft tube design, whereas the segment located in the Actuation Zone is constructed with higher stiffness—employing either a semi-rigid or rigid tube—to prevent bending deformation.

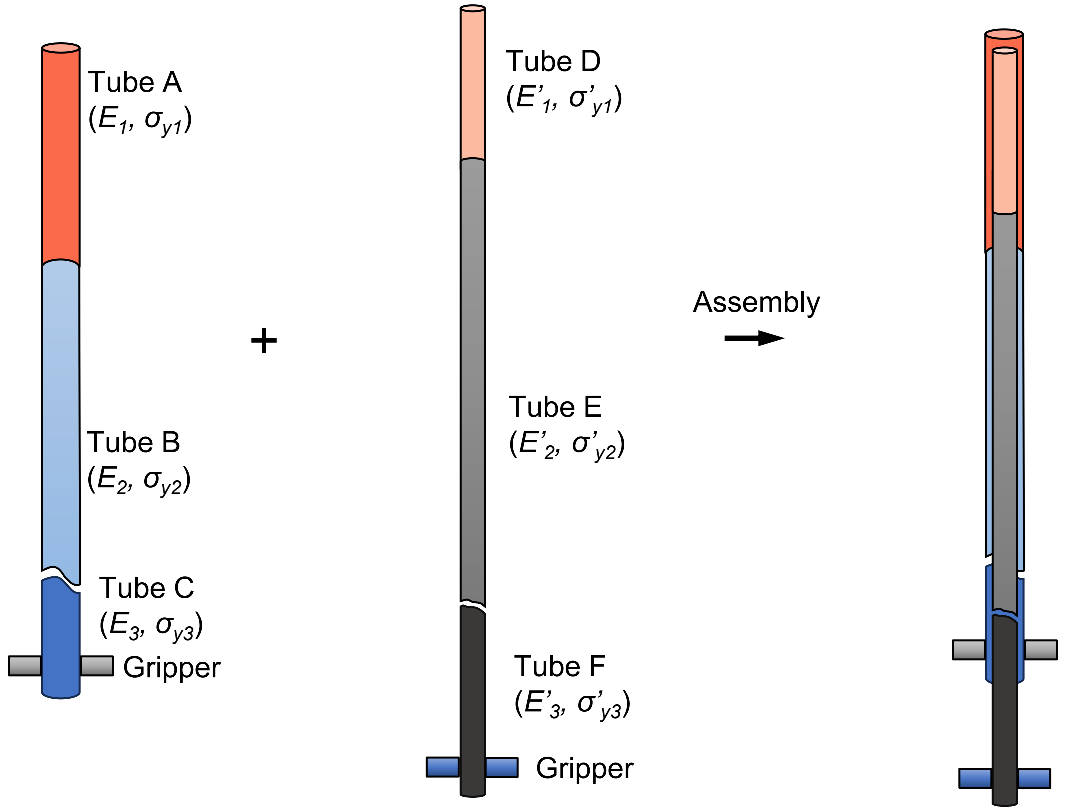

Supplementary Fig. 77. Tube design utilizing the segmented design method. Both the outer and inner tubes are divided into three segments, each featuring distinct elastic moduli and yield strengths that progressively increase from the distal end toward the gripper end. The three segmented sections are secured together to form a composite tube, which is then nested for assembly.

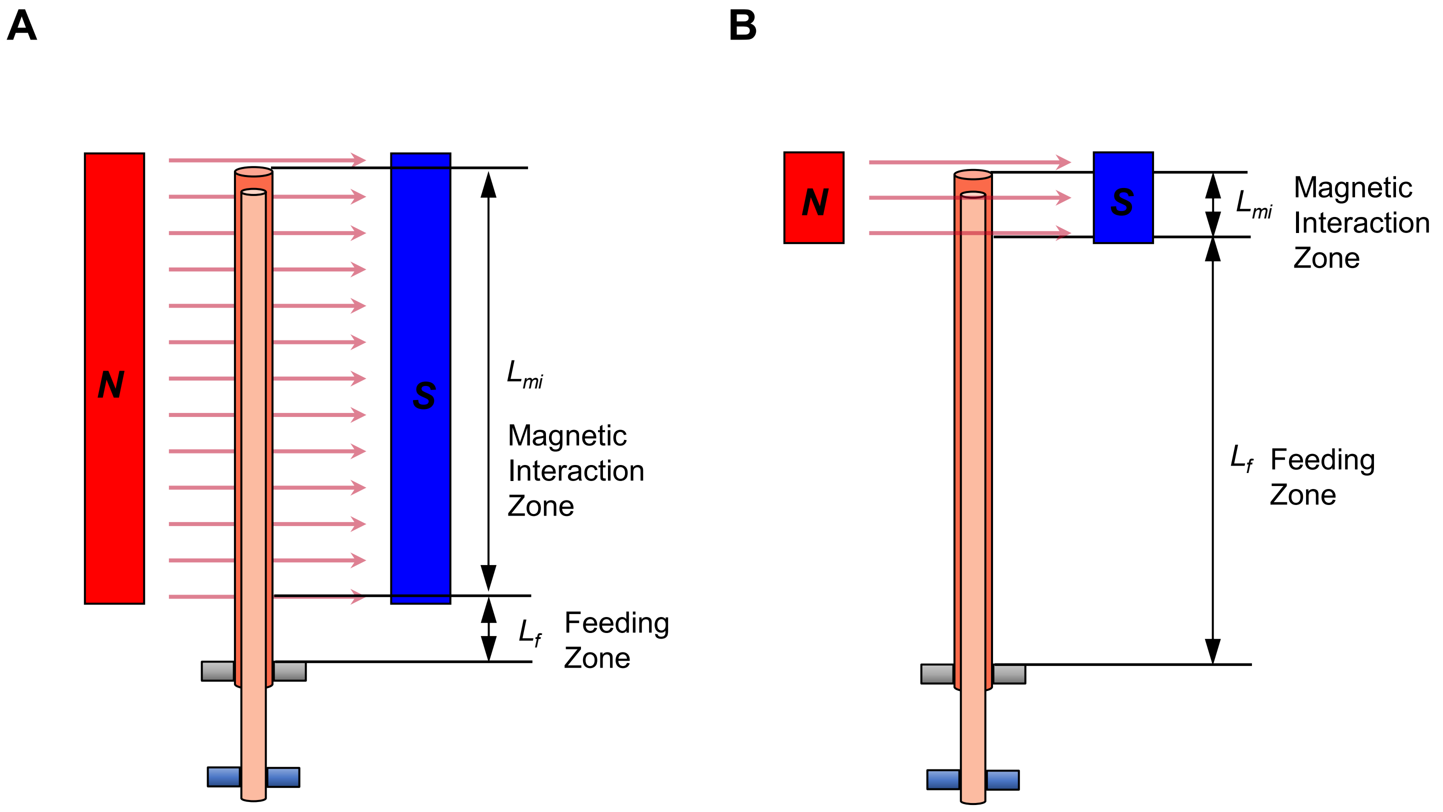

Supplementary Fig. 78. Different modes of long-range operation. (**A**) Long-range operation with a large Magnetic Interaction Length. This operating mode features an extensive magnetic field region—that is, a relatively large Magnetic Interaction Zone paired with a comparatively small Feeding Zone. Accordingly, it is characterized by a large Magnetic Interaction Length and a small Feeding Length. (**B**) Long-range operation with a large Feeding Length. This operating mode, in contrast to the previous one, features a small Magnetic Interaction Zone paired with a larger Feeding Zone.

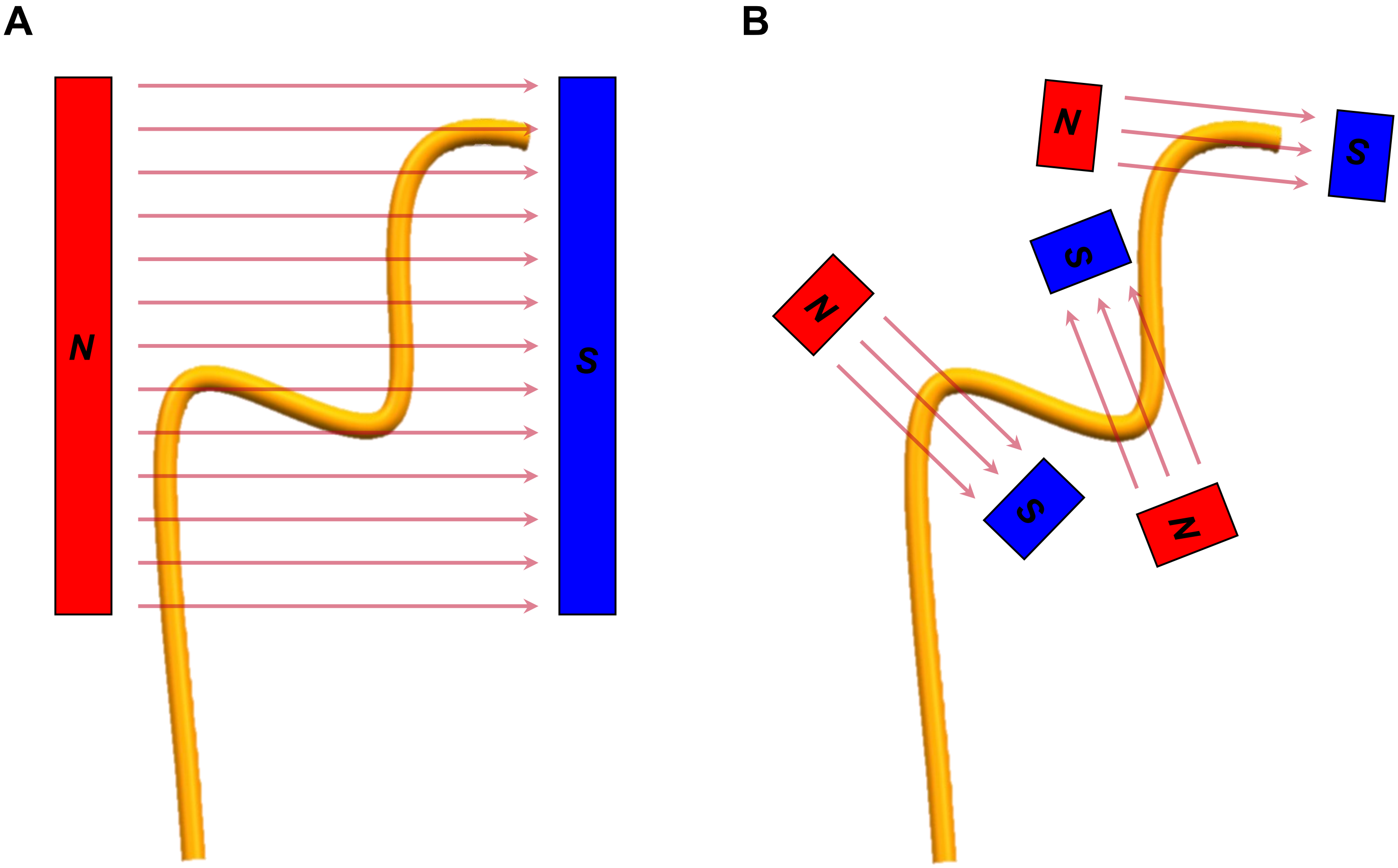

Supplementary Fig. 79. Operation with a large Magnetic Interaction Zone and multi-magnetic field control method. (**A**) Operation with a large Magnetic Interaction Zone. When the tube needs to operate within an extensive magnetic field region, a large-scale magnetic field generation device is used to create a magnetic field that covers the tube's entire workspace. (**B**) Proposed multi-magnetic field control method. By superimposing multiple small-scale magnetic fields in space, different regions of the tube can be independently controlled, thereby substituting for a large-scale magnetic field.

A

B

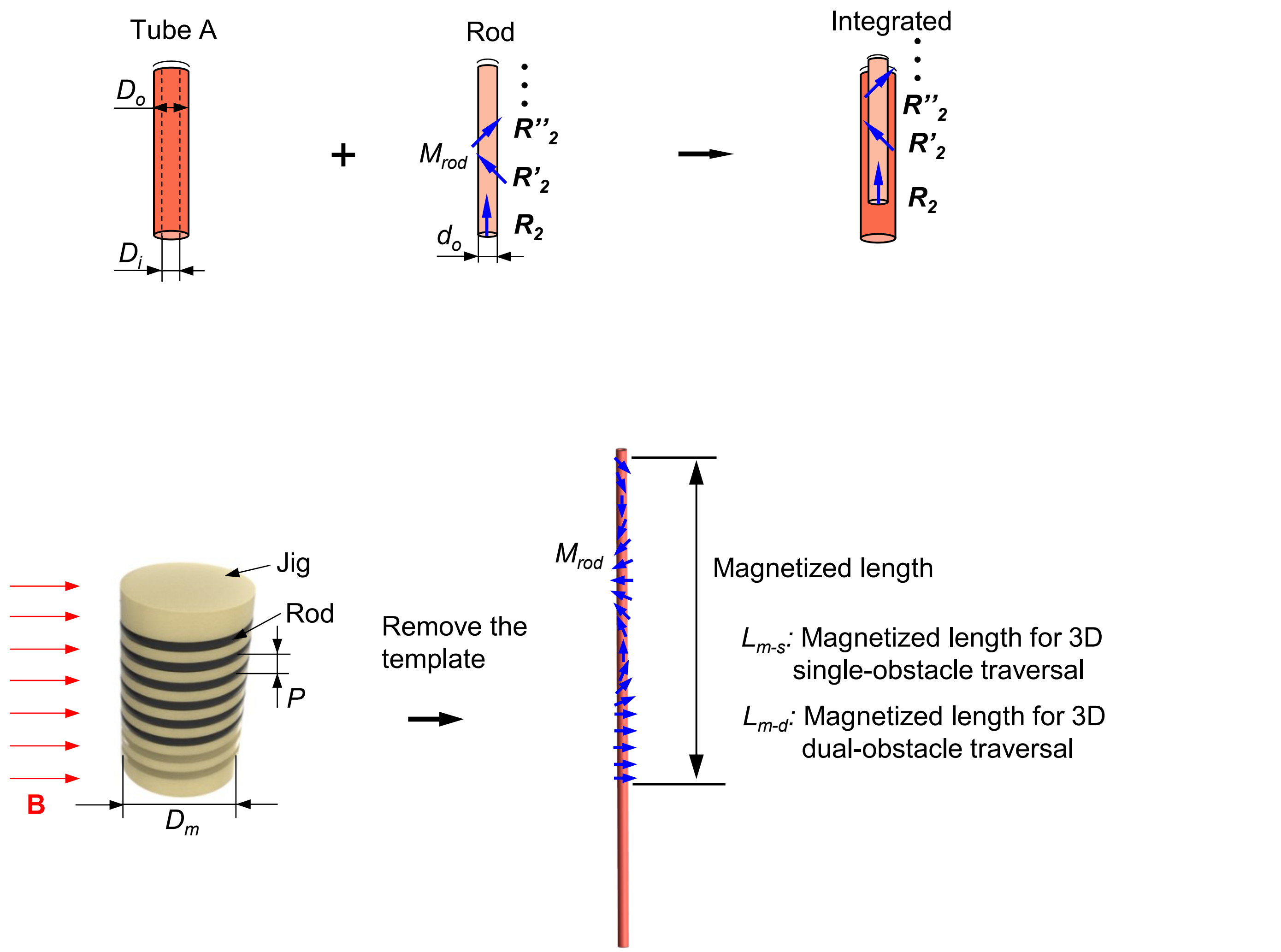

C

Symbol	Value
D_o	2.6 mm
D_i	1.7 mm
d_o	1.4 mm
D_m	19 mm
P	3 mm
M_{rod}	188716 A/m
$L_{m\text{-}s}$	50 mm
$L_{m\text{-}d}$	68 mm

Supplementary Fig. 80. Tube design parameters for the 3D navigation operation demonstration. We present two types of 3D operations, namely, "3D single-obstacle traversal under a single magnetic field" and "3D dual-obstacle traversal under a single magnetic field". Although the designs for both operations are similar, they differ in the magnetized length. The variation in magnetized length, when processed using a template, results in distinct magnetization profiles, which in turn lead to different deformations. (**A**) Tube configuration. (**B**) Magnetization method. (**C**) Specific parameter values.

A

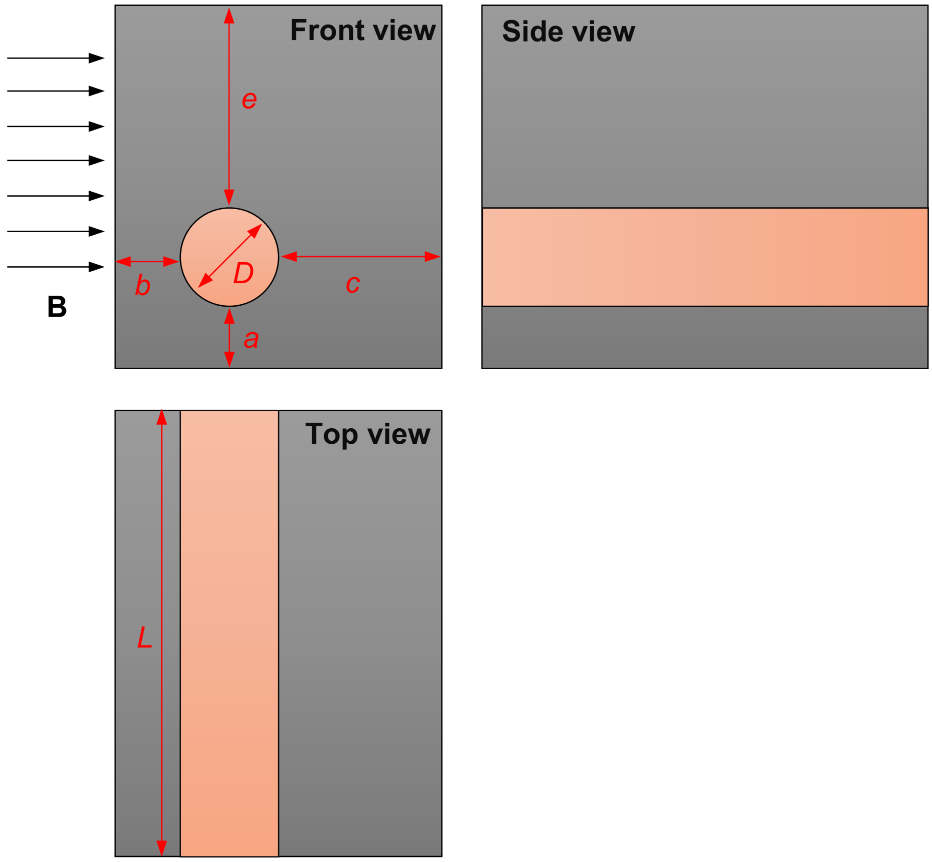

B

Symbol	Value
D	14 mm
a	11 mm
b	13 mm
c	25 mm
e	35 mm
L	80 mm

Supplementary Fig. 81. Experimental setup for 3D single-obstacle traversal. (**A**) Three-view illustration of the setup. A single obstacle is involved and the applied magnetic field is unidirectional, oriented from left to right in the front view. (**B**) Specific parameter values.

A

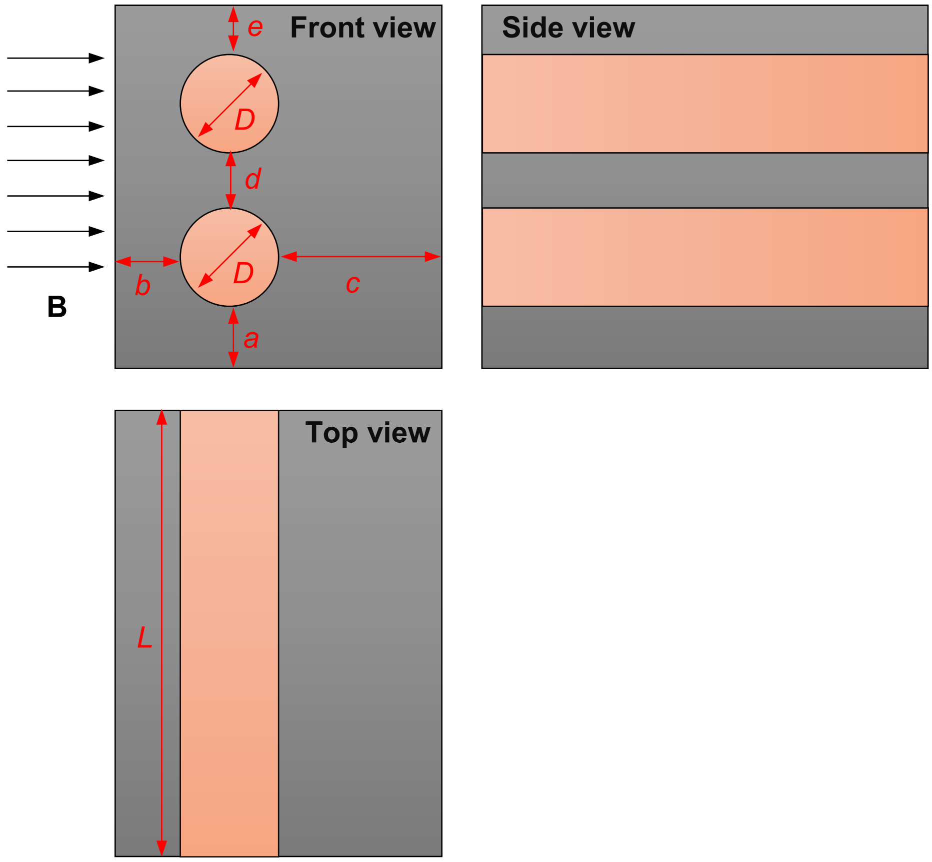

B

Symbol	Value
D	14 mm
a	13 mm
b	13 mm
c	25 mm
d	13 mm
e	6 mm
L	80 mm

Supplementary Fig. 82. Experimental setup for 3D dual-obstacle traversal. (**A**) Three-view illustration of the setup. Two obstacles are involved and the applied magnetic field is unidirectional, oriented from left to right in the front view. (**B**) Specific parameter values.

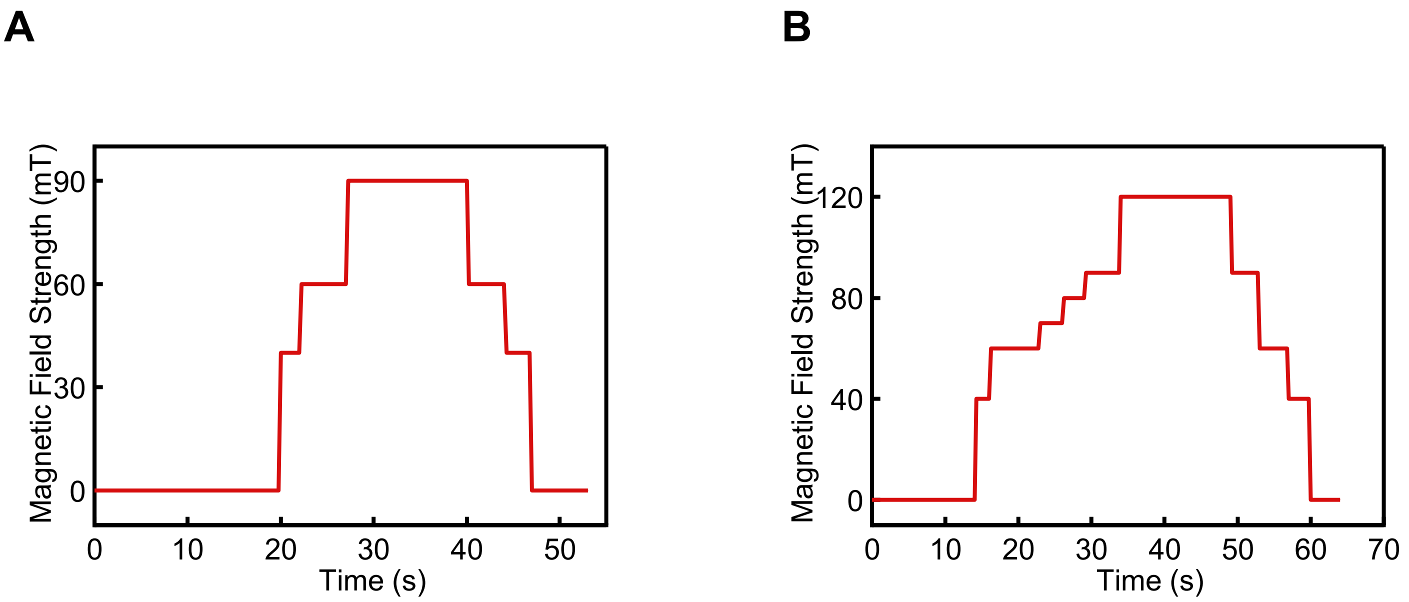

Supplementary Fig. 83. Magnetic field applied in the 3D navigation demonstration in Supplementary Figs. 81-82. During the operation, the direction of the magnetic field remained constant, while its magnitude was gradually varied to facilitate smooth tube deformation. (**A**) 3D single-obstacle traversal. (**B**) 3D dual-obstacle traversal.

A

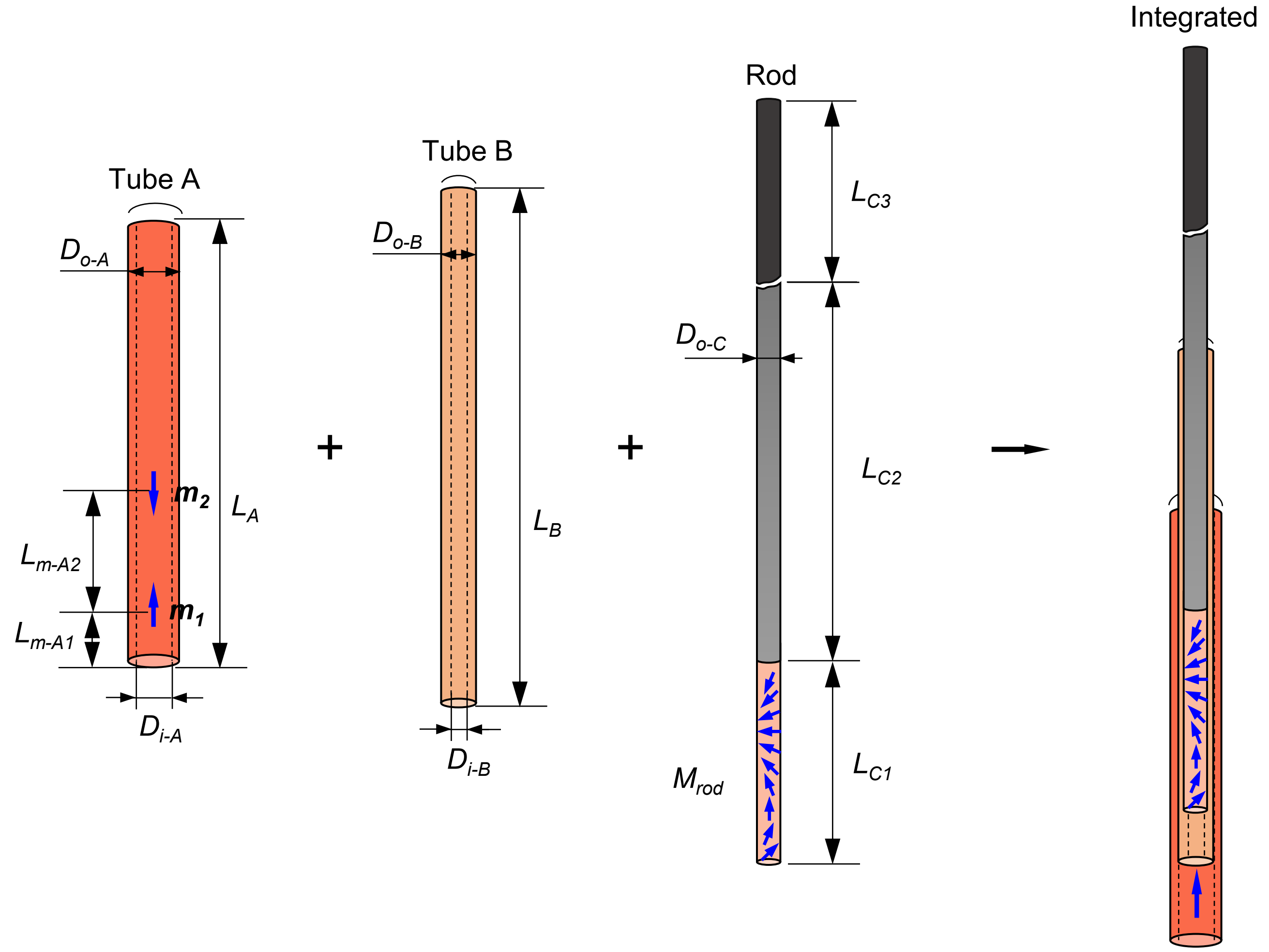

B

Symbol	Value
$D_{o\text{-}A}$	4.8 mm
$D_{i\text{-}A}$	3.8 mm
L_A	155 mm
$L_{m\text{-}A1}$	52 mm
$L_{m\text{-}A2}$	47 mm
$D_{o\text{-}B}$	2.8 mm
$D_{i\text{-}B}$	2 mm
L_B	315 mm
$D_{o\text{-}C}$	1.4 mm
L_{C1}	53 mm
L_{C2}	300 mm
L_{C3}	85 mm
m_1	2.4×10^{-2} Am^2
m_2	2.4×10^{-2} Am^2
M_{rod}	188716 A/m

Supplementary Fig. 84. Design parameters of the tube involved in long-range operation with a large Magnetic Interaction Length. (**A**) Tube configuration. The magnetization profile at the rod's tip is identical to the tube's magnetization profile for the 3D single-obstacle traversal shown in Supplementary Fig. 80. (**B**) Specific parameter values.

A

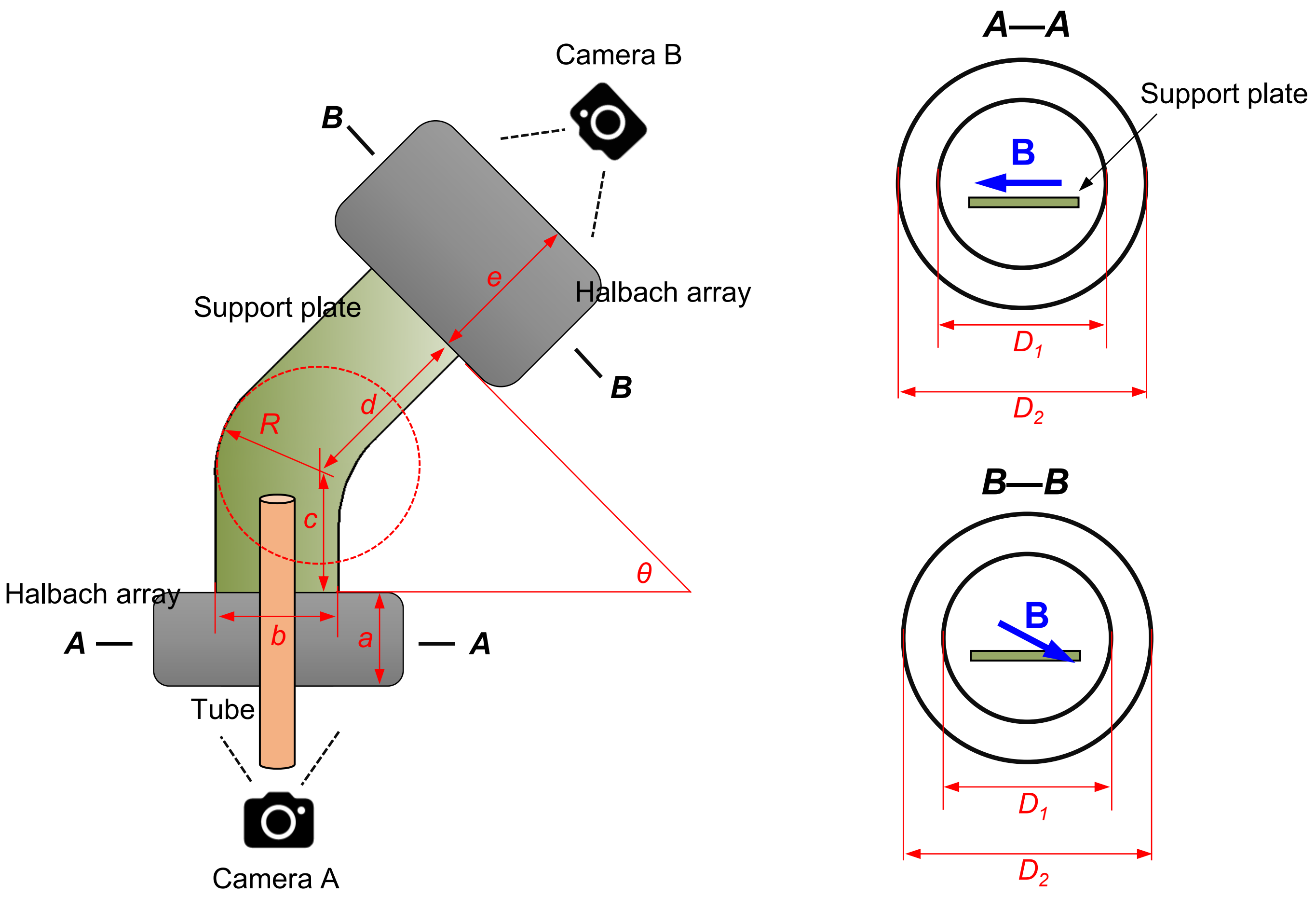

B

Symbol	Value
D_1	110 mm
D_2	170 mm
R	70 mm
θ	45°
a	40 mm
b	70 mm
c	89 mm
d	91 mm
e	102 mm
B	45 mT

Supplementary Fig. 85. Experimental setup for long-range operation with a large Magnetic Interaction Length. (**A**) Top view schematic and cross-sectional view of Halbach arrays. The figure illustrates an experimental platform composed of two Halbach arrays arranged at 45°, creating dual magnetic fields. A support plate is positioned in the intermediate region between the two Halbach arrays to provide support for the tube. Cameras are installed on the exterior sides of the Halbach arrays to record the tube's real-time operation, with the tube entering the Halbach array near Camera A. (**B**) Specific parameter values.

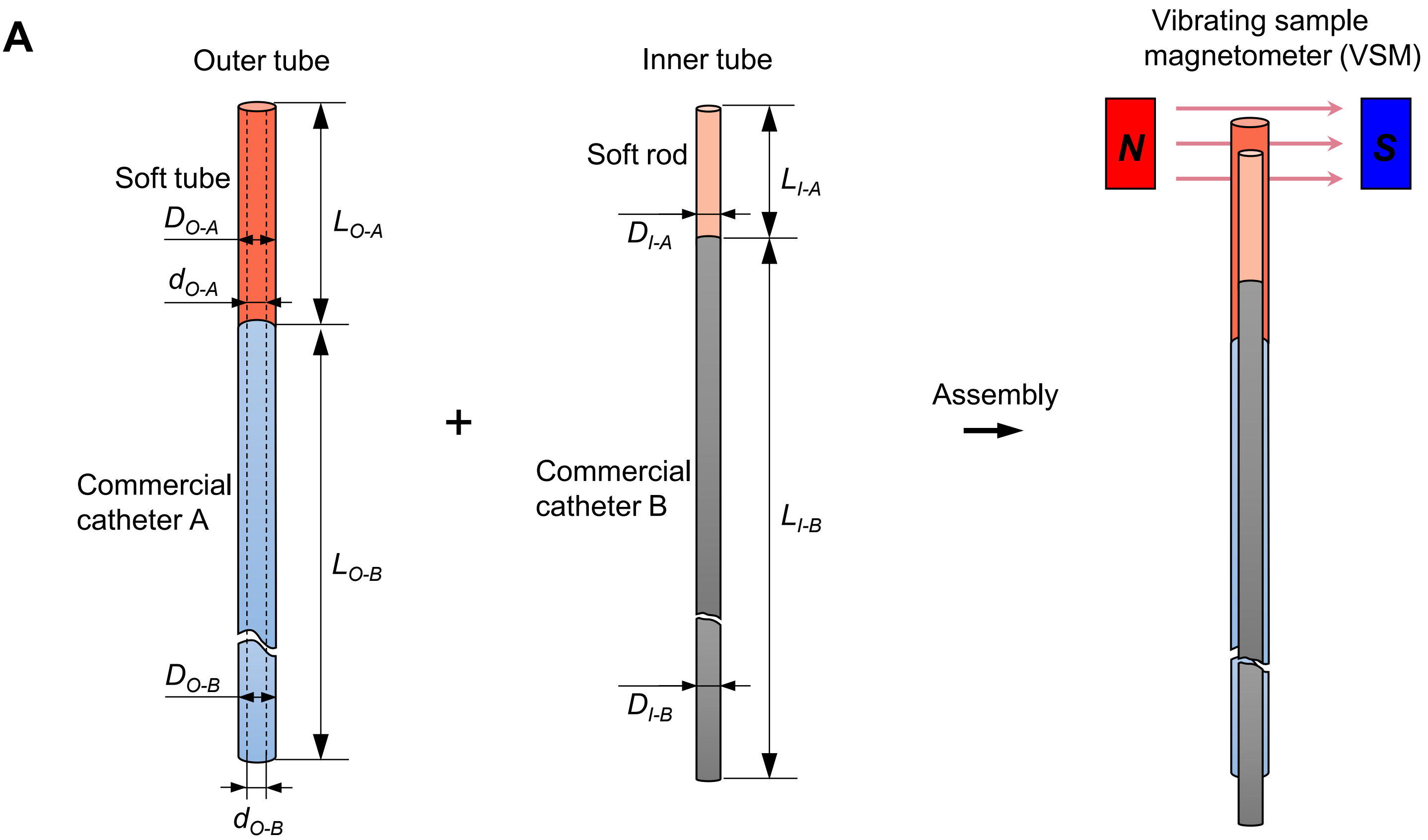

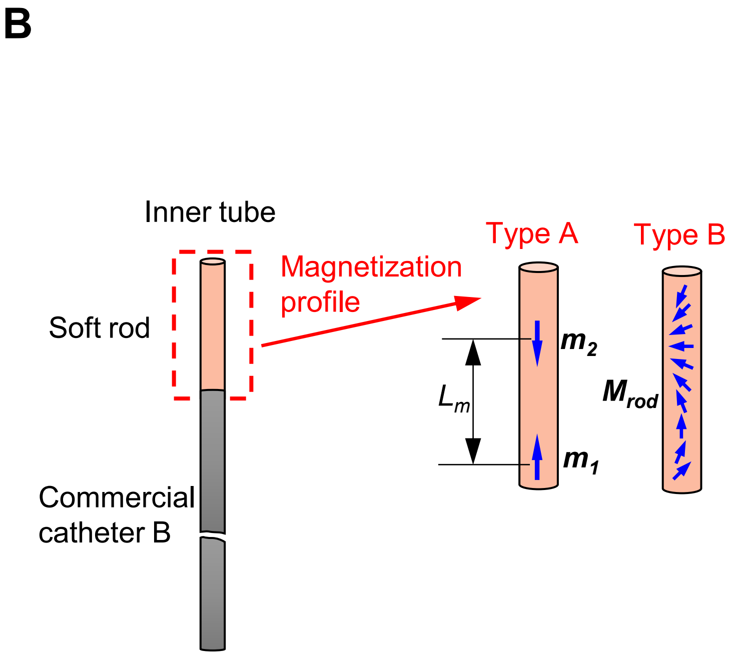

C

Symbol	Value
$D_{O\text{-}A}$	2.8 mm
$D_{O\text{-}B}$	2.7 mm
$d_{O\text{-}A}$	2 mm
$d_{O\text{-}B}$	2.28 mm
$L_{O\text{-}A}$	140 mm
$L_{O\text{-}B}$	1100 mm
$D_{I\text{-}A}$	1.4 mm
$D_{I\text{-}B}$	2.2 mm
$L_{I\text{-}A}$	140 mm
$L_{I\text{-}B}$	1100 mm
L_m	12 mm
m_1	1.02×10^{-3} Am^2
m_2	1.02×10^{-3} Am^2
M_{rod}	188716 A/m

Supplementary Fig. 86. Design parameters of the tube involved in long-range operation with a large Feeding Length. (**A**) Tube configuration. The outer tube is fabricated by connecting a commercial catheter with a soft tube, while the inner tube is assembled by linking a commercial catheter with a soft rod. Commercial catheters A and B are sourced from a catheter kit (WAIN-FBK-6SD110, ASAHI INTECC Co. LTD., JP). The vibrating sample magnetometer (VSM) is used to generate magnetic fields. (**B**) Two different magnetization profiles of the soft rod. Type A exhibits deformation confined to a plane, whereas Type B enables 3D deformation. The magnetization profile of Type B is identical to the one shown in Supplementary Fig. 80 for the 3D single-obstacle traversal. (**C**) Specific parameter values.

A

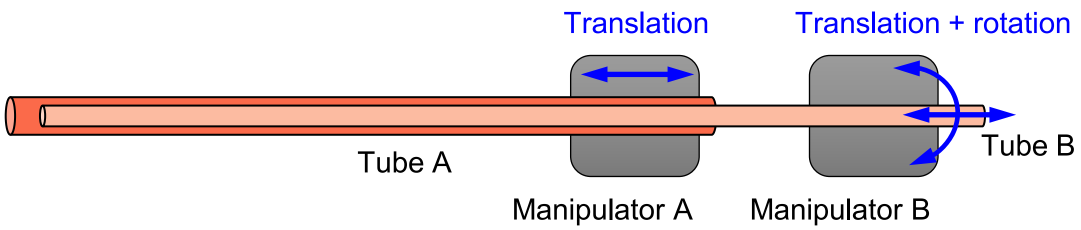

B

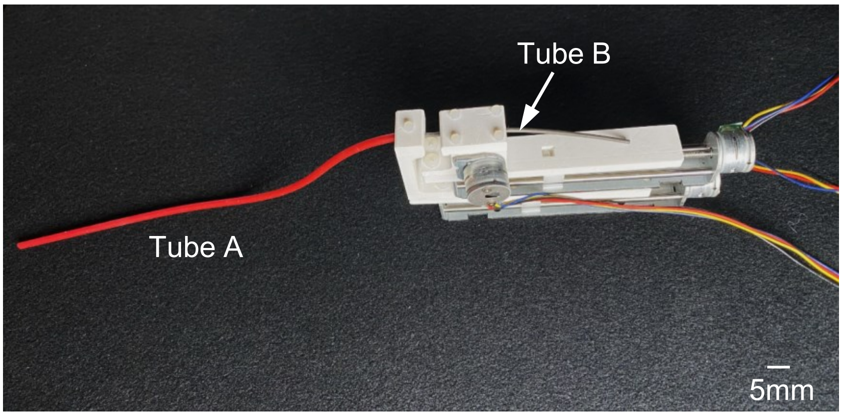

Supplementary Fig. 87. Schematic diagram and photograph of the tube operating mechanism. (**A**) Schematic diagram. The schematic diagram illustrates the tube operating mechanism, which utilizes two manipulators—manipulator A and manipulator B—to control the outer tube (Tube A) and the inner tube (Tube B), respectively. Manipulator A is designed to perform translational motion, whereas manipulator B is capable of both translational and rotational motions. Naturally, changes in the tube configuration may lead to variations in the number of tubes and their motion modes, thereby necessitating corresponding modifications to the manipulators. (**B**) Photograph.

A

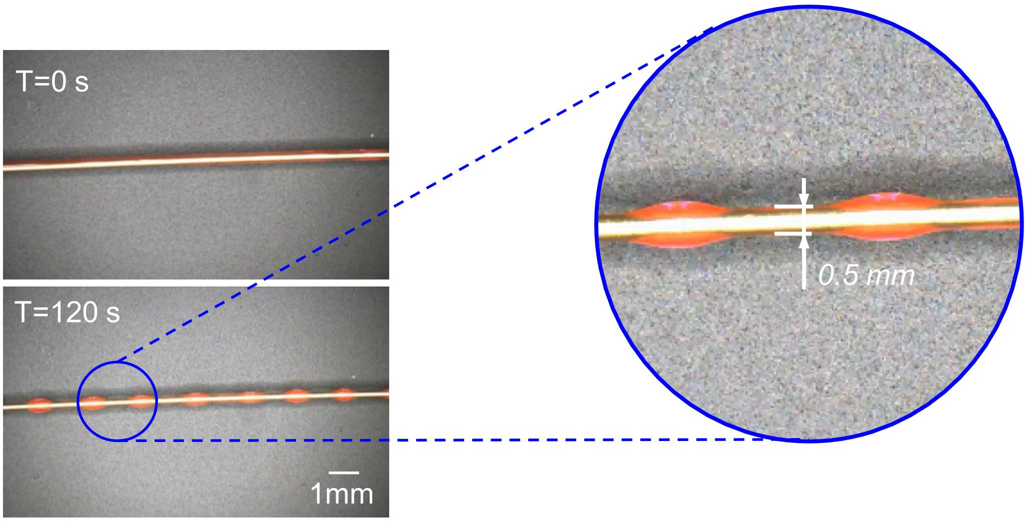

B

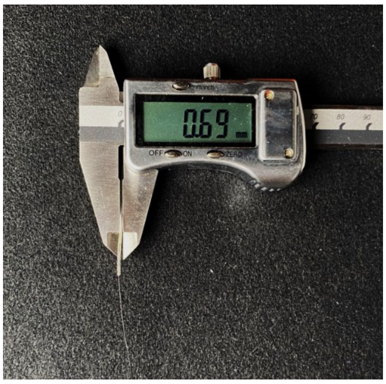

C

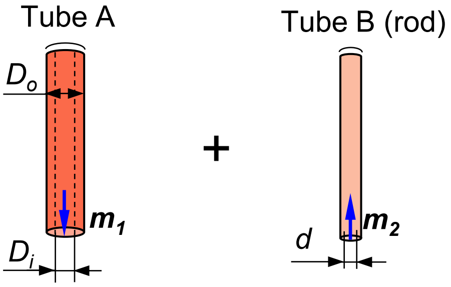

Symbol	Value
D_o	0.7 mm
D_i	0.4 mm
d	0.3 mm
m_1, m_2	2.5×10^{-4} Am^2

Supplementary Fig. 88. Problems encountered during miniature tube fabrication and photographs with parameters of the fabricated tubes. (**A**) Illustration of problems encountered in fabricating miniature tubes using the dip-coating method. To fabricate small-scale tubes, copper wires with a diameter of 0.5 mm were employed. Given that the diameter of these copper wires is significantly smaller than those used previously, surface tension dominates at this scale. Consequently, the PDMS coated onto the copper wires forms spherical droplets under the influence of surface tension, making it impossible to fabricate tubes with inner diameters smaller than 0.5 mm using the dip-coating method. (**B**) Tubes re-fabricated from commercial tubing. Commercially available tubes (06420-01, Cole-Parmer, US) were heated, melted, and stretched to fabricate tubes at a smaller scale. (**C**) Configuration and parameters for the re-fabricated tube.

A

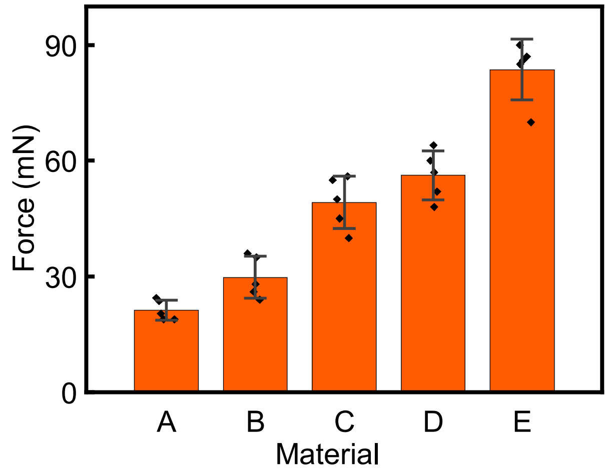

Symbol	Materials
A	PDMS, Base-to-curing agent ratio (B:C ratio): 20:1
B	PDMS, B:C ratio 15:1
C	PDMS, B:C ratio 10:1
D	PDMS, B:C ratio 3:1
E	PDMS(B:C ratio10:1) + Magnetic particle $m_{PDMS} : m_{Magnetic\ particle}$=3:1

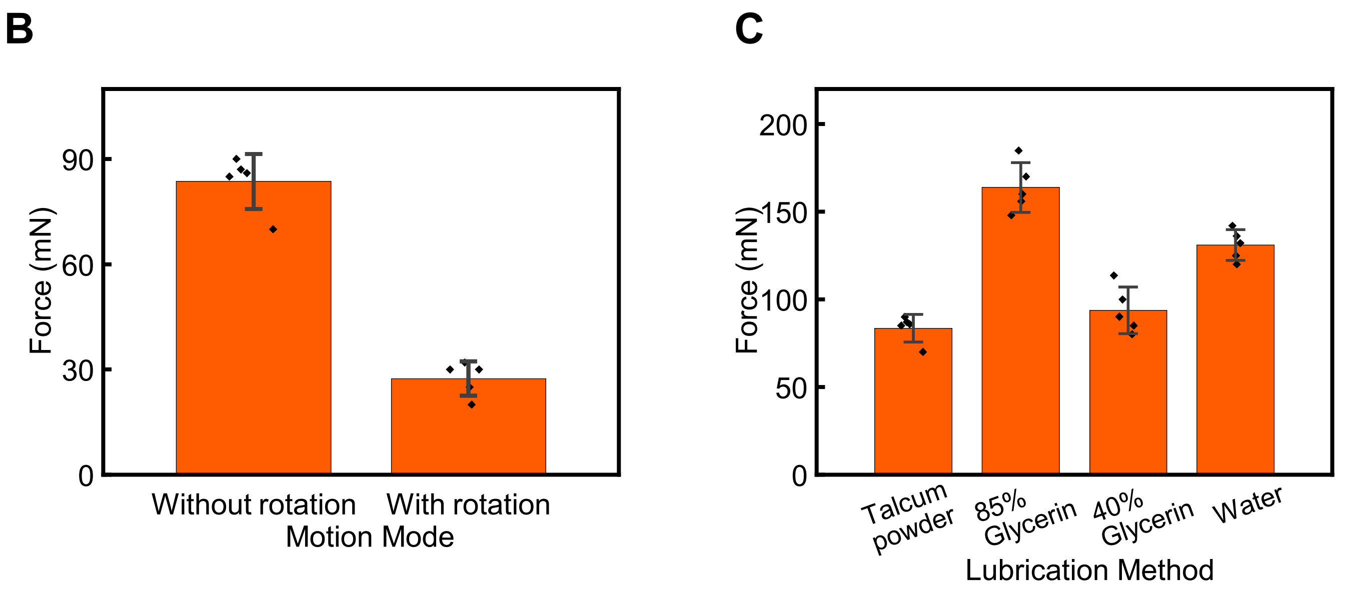

Supplementary Fig. 89. Influence of various parameters on the relative motion resistance involved in manipulating tubes. (**A**) Effect of different materials on relative motion resistance. (**B**) Effect of different motion modes on relative motion resistance. (**C**) Effect of different lubrication methods on relative motion resistance. All experiments were conducted using the setup shown in Supplementary Fig. 64A, with a chosen bend curvature radius of 10 mm. Each type of operation was repeated 5 times (*n*=5). Bars represent mean values; error bars denote the standard deviation.

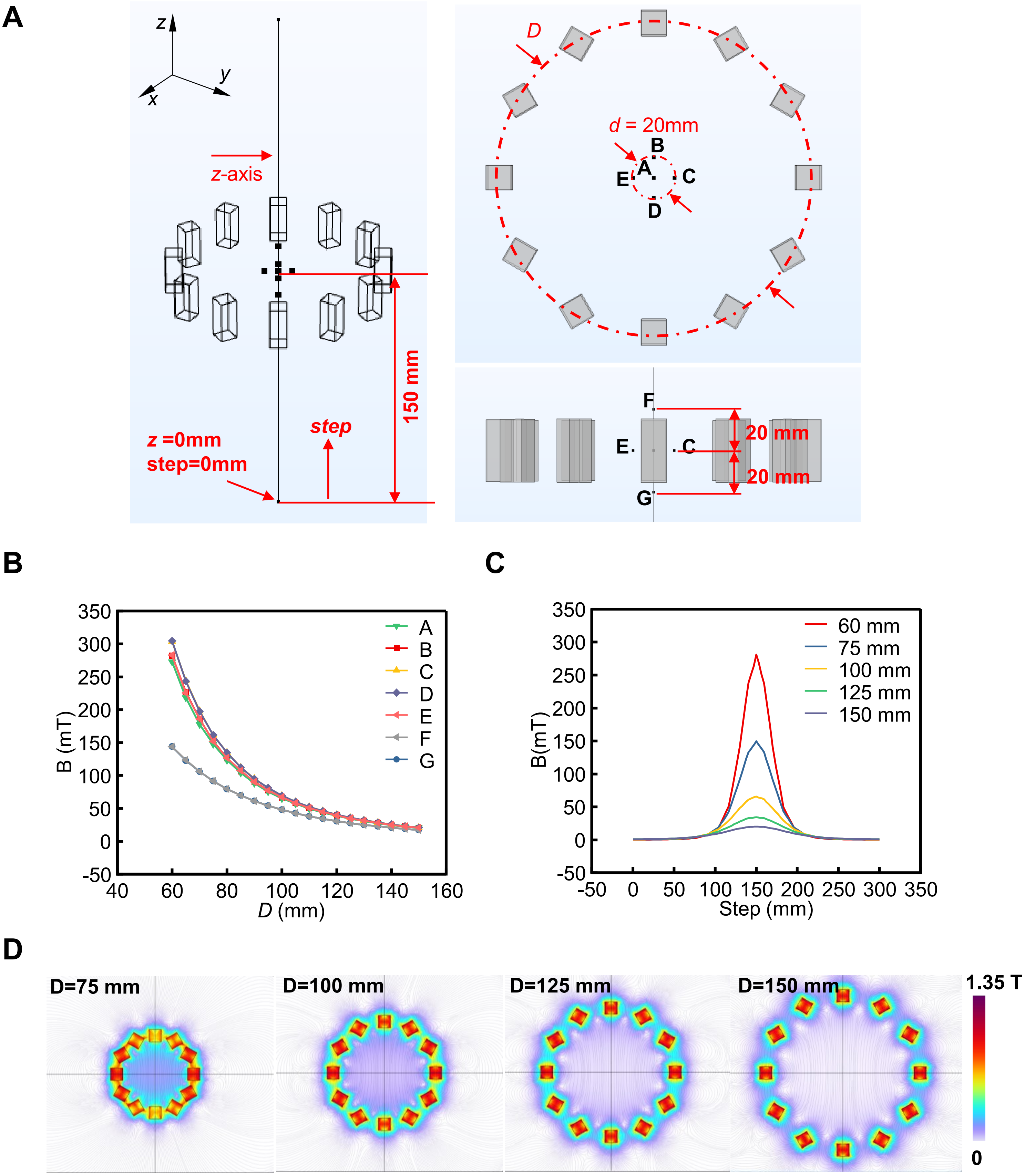

Supplementary Fig. 90. Influence of diameter of the magnet distribution circle on magnetic field intensity in different regions. (**A**) Schematic diagram showing dimensions of the Halbach array. The illustration includes specific dimensions of the Halbach array and the positions of sampling points. (**B**) Variation of magnetic field intensity at different sampling points with respect to the diameters of the magnet distribution circle in the Halbach array. (**C**) Variation of magnetic field intensity along the *z*-axis with changing diameters of the magnet distribution circle in the Halbach array. The term "step" represents the sampling interval achieved by moving upward along the *z*-axis in (A). (**D**) Distribution of magnetic field intensity on the *z* = 150 mm plane.

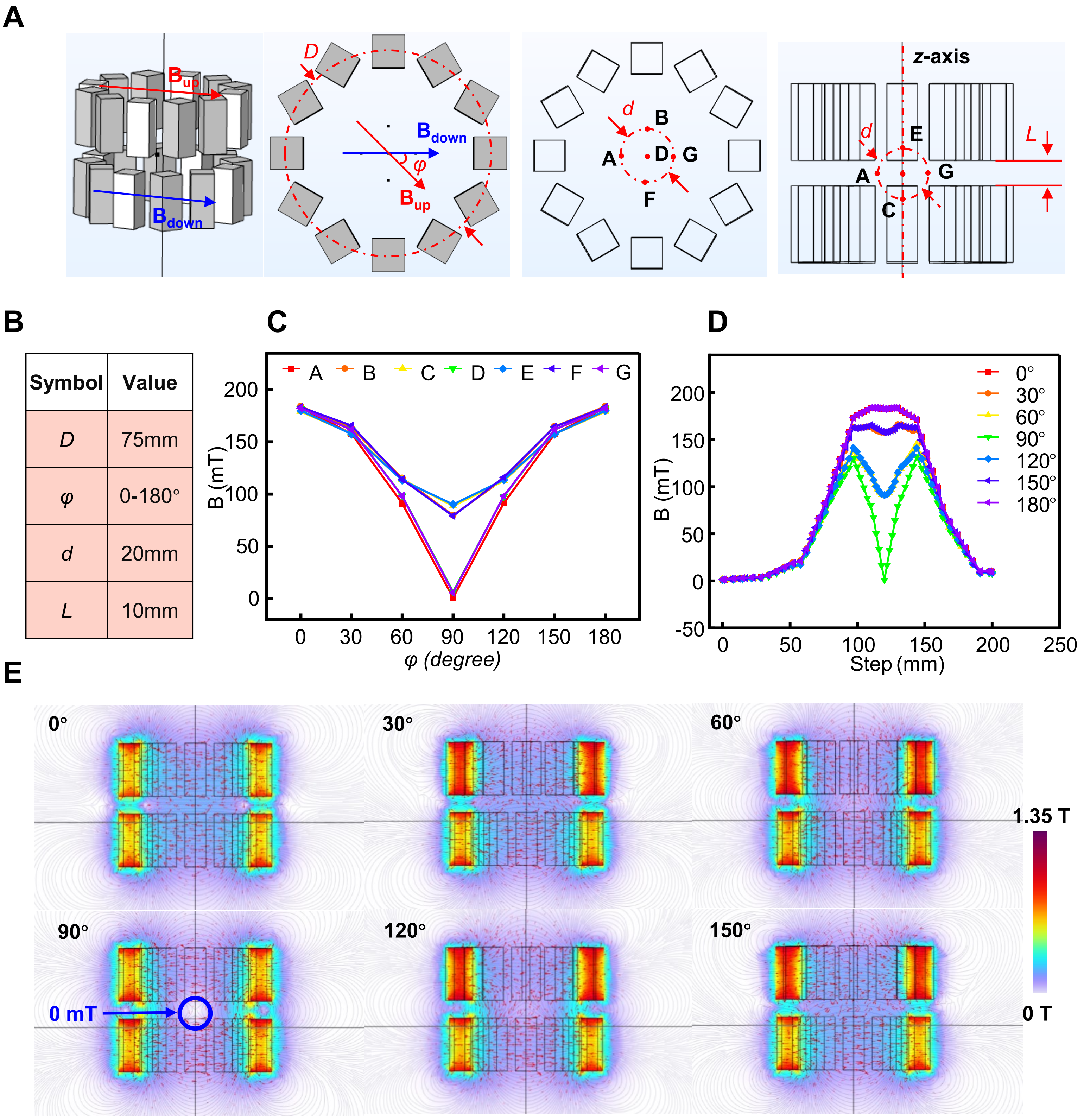

Supplementary Fig. 91. Impact of the magnetic field angle on the magnetic field in different regions. (**A**) Schematic of the two Halbach arrays and the sampling points. Two magnetic fields are generated by the two Halbach arrays, whose end faces are parallel. The magnetic field generated by the upper Halbach array is denoted as $\mathbf{B}_{up}$, and that of the lower Halbach array as $\mathbf{B}_{down}$. φ represents the angle between $\mathbf{B}_{up}$ and $\mathbf{B}_{down}$. (**B**) Values of the parameters in (A). (**C**) Magnetic field intensities at various sampling points under different magnetic field angles. (**D**) Magnetic field intensities along the *z*-axis under different magnetic field angles. Here, "step" refers to the sampling interval achieved by moving upward along the *z*-axis in Supplementary Fig. 90A. In Supplementary Fig. 90A, the starting point for the step is located 150 mm downward along the central axis from the center of the lower Halbach array, and the displacement used in this setup is 120 mm. (**E**) Magnetic field distribution under different magnetic field angles.

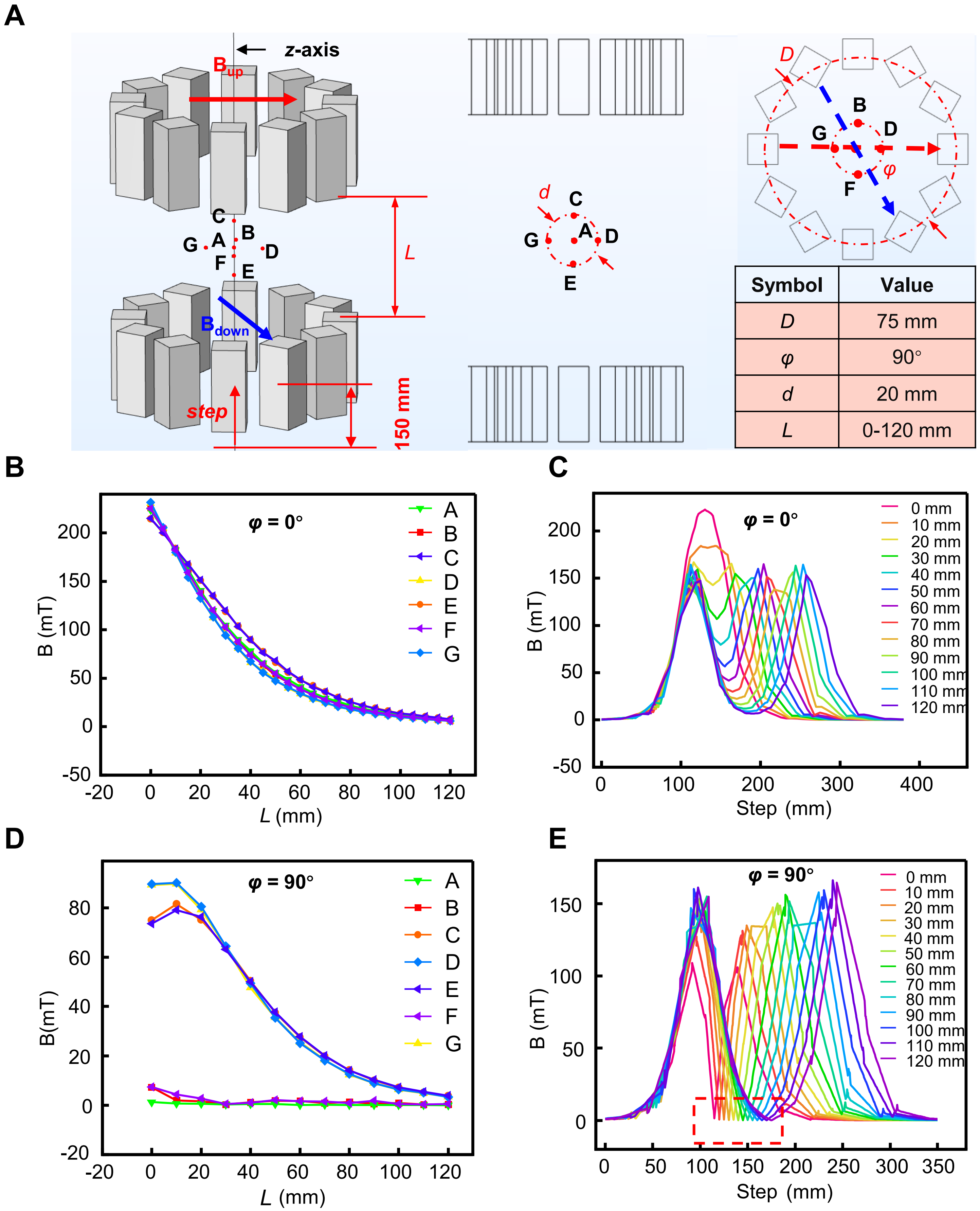

Supplementary Fig. 92. Impact of magnetic field spacing on the magnetic field in different regions. (**A**) Schematic of the arrangement of two Halbach arrays and the positions of the sampling points. Two magnetic fields are generated by the two Halbach arrays, whose end faces are parallel. *L* represents the distance between the end faces of the two Halbach arrays (magnetic field spacing). *φ* represents the angle between $\mathbf{B}_{up}$ and $\mathbf{B}_{down}$. (**B**) Magnetic field intensities at various sampling points when *φ* is 0°. (**C**) Magnetic field intensities along the *z*-axis when *φ* is 0°. Here, “step” denotes the sampling interval achieved by moving upward along the *z*-axis in (A). (**D**) Magnetic field intensities at various sampling points when *φ* is 90°. (**E**) Magnetic field intensities along the z-axis when *φ* is 90°.

A

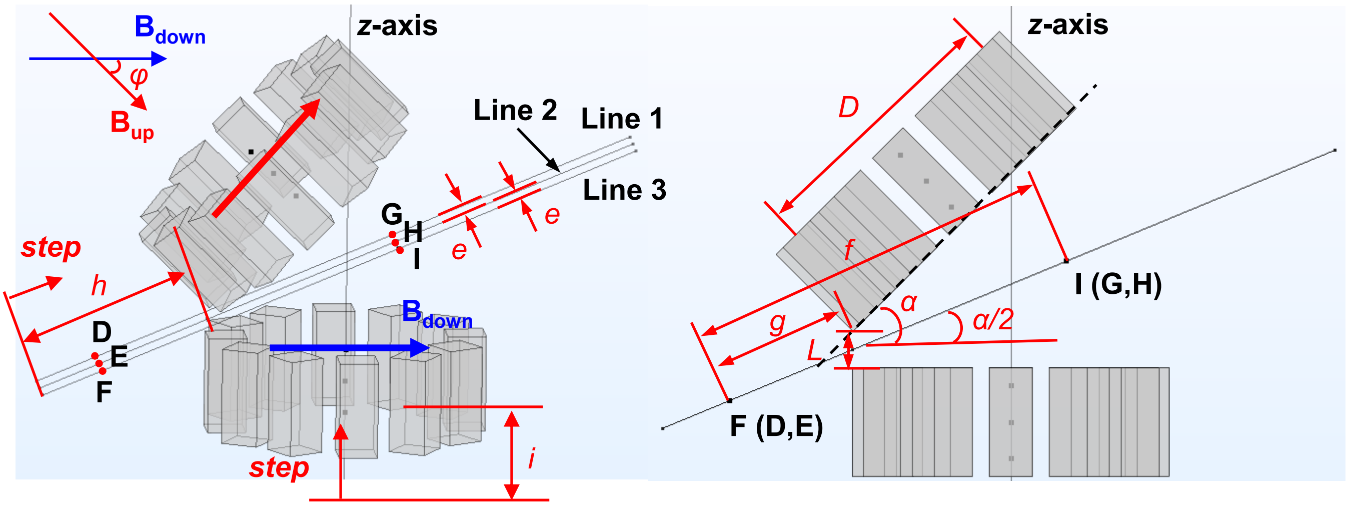

B

Symbol	Value
D	75 mm
φ	90°
d	20 mm
L	10 mm
e	10 mm
f	100 mm
g	36.5 mm
h	56.5 mm
i	100 mm

C

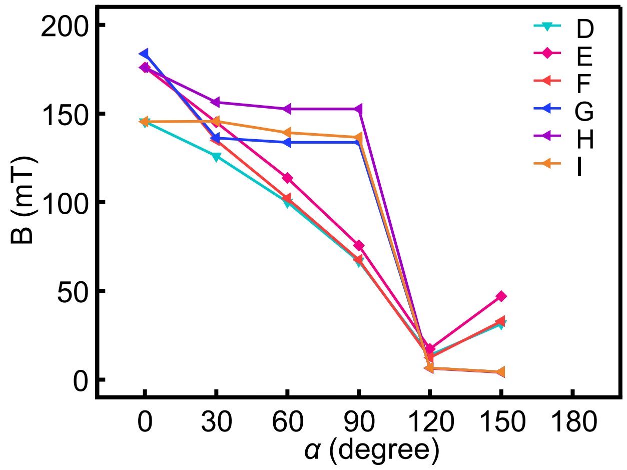

D

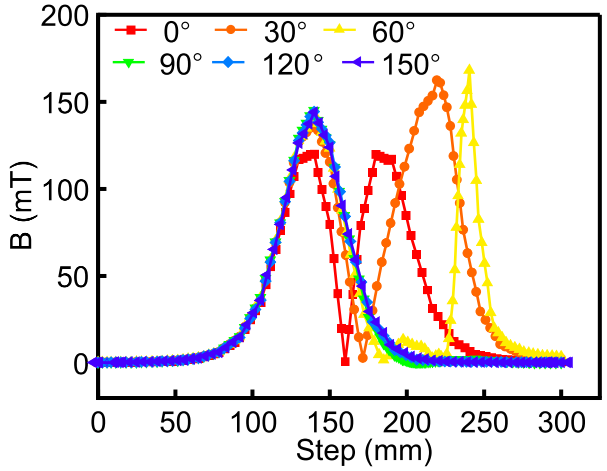

Supplementary Fig. 93. Impact of the magnetic field plane angle on the magnetic field in different regions. (**A**) Schematic showing the arrangement of the two Halbach arrays and the positions of the sampling points. Two magnetic fields are generated by the Halbach arrays, with the angle between their magnetic field planes (i.e., the end faces of the arrays) being *α*. (**B**) Values of the relevant parameters in (A). (**C**) Magnetic field intensities at various sampling points under different magnetic field plane angles. (**D**) Magnetic field intensities along the *z*-axis under different magnetic field plane angles. Here, "step" refers to the sampling interval achieved by moving upward along the *z*-axis in (A).

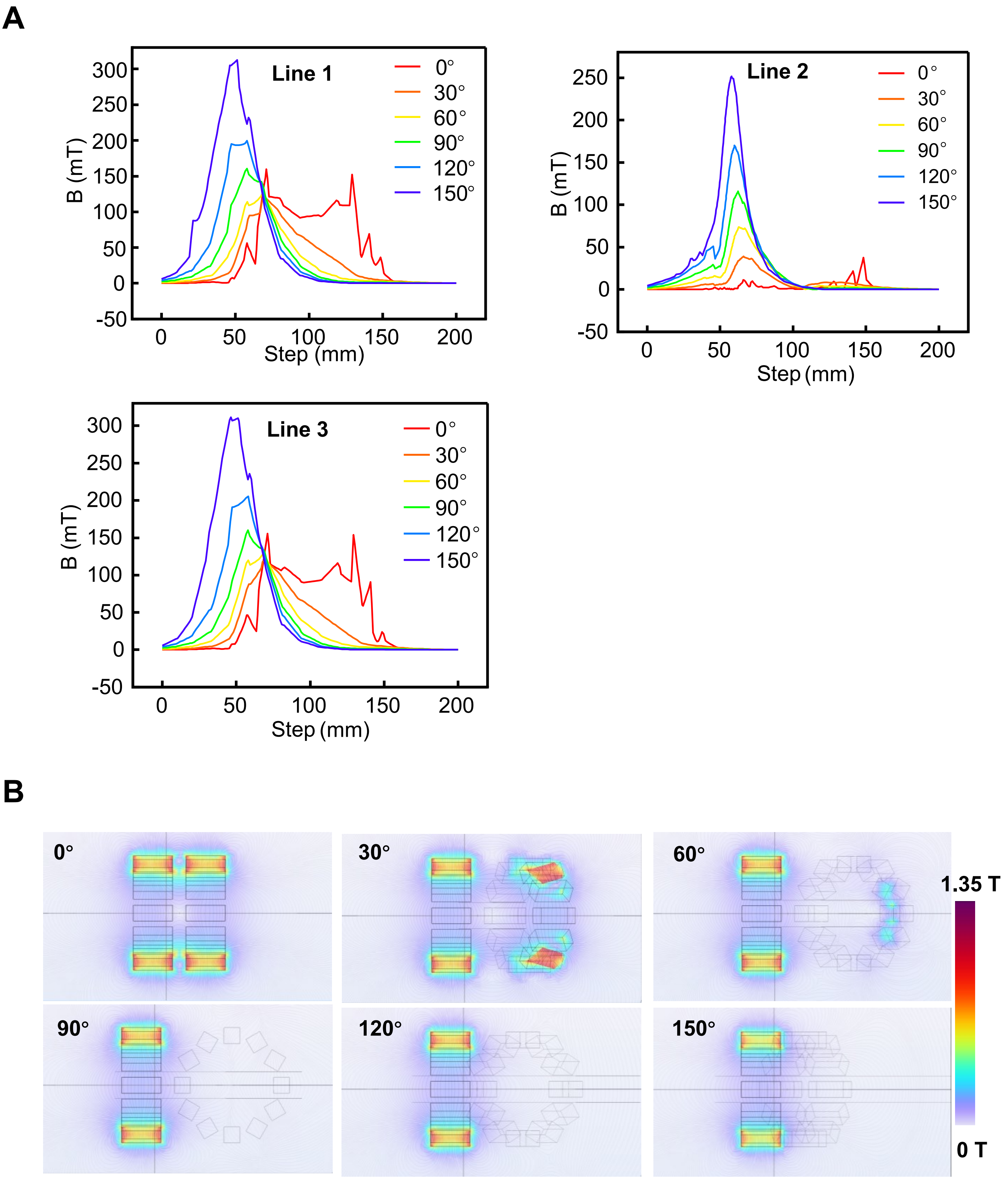

Supplementary Fig. 94. Continuation Figure: Impact of the magnetic field plane angle on the magnetic field in different regions. (**A**) Magnetic field intensities along Lines 1, 2, and 3 in Supplementary Fig. 93A. Here, “step” denotes the sampling interval achieved by moving along the axis of Lines 1, 2, and 3 in Supplementary Fig. 93A. (**B**) Magnetic field distribution under different magnetic field plane angles.

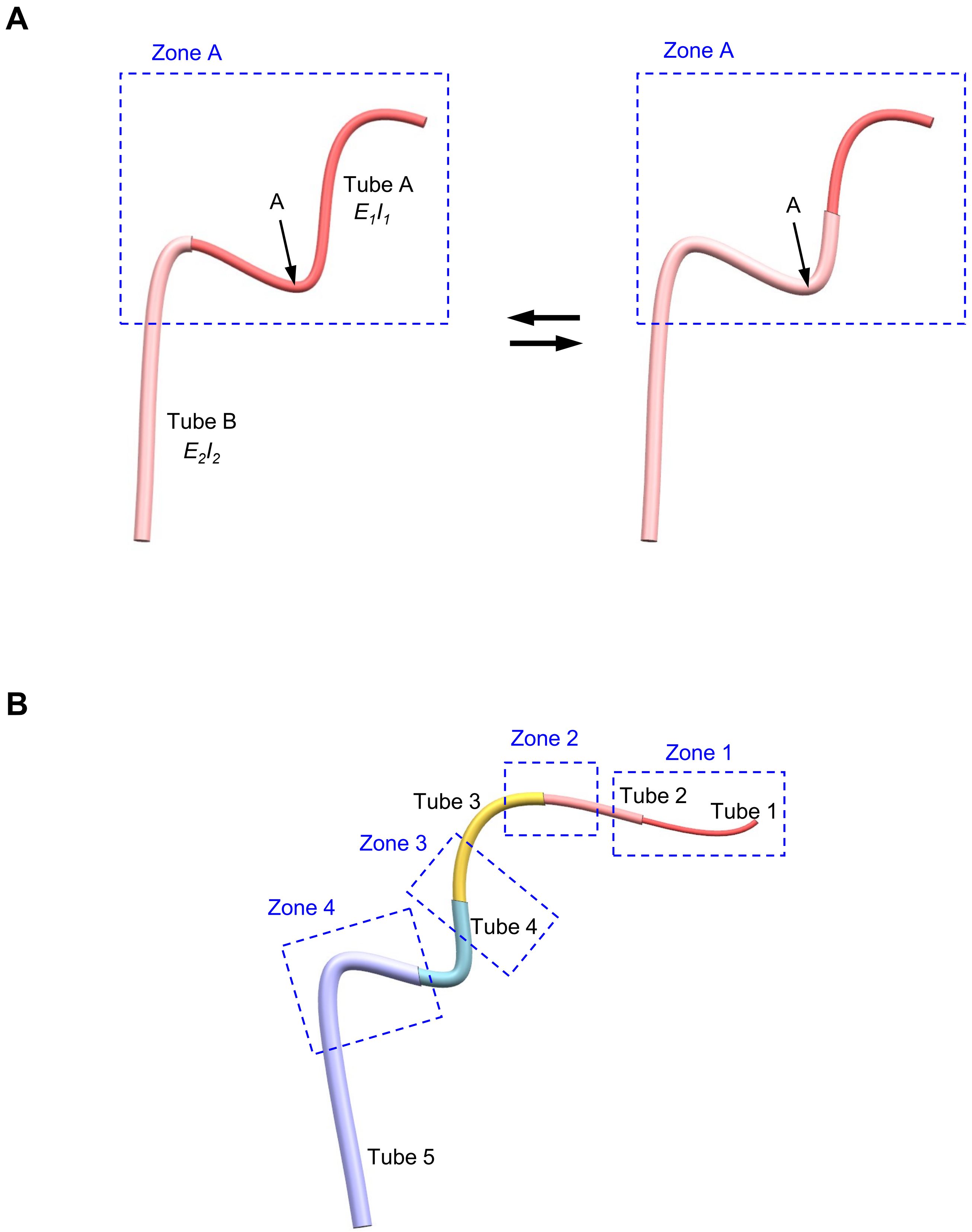

Supplementary Fig. 95. Demonstration of stiffness variation in the multi-nested tube and proposed zone-based tube nesting design method. (**A**) Stiffness variation in the multi-nested tube. In zone A, as the position of tube B changes, point A transitions from containing only tube A to containing both tube A and B, thereby altering the stiffness at that point. As the number of tubes increases, this stiffness variation becomes increasingly pronounced. (**B**) Proposed zone-based tube nesting design method. The nested tubes are partitioned into zones so that each tube moves only within its designated zone. As a result, each zone contains a fixed number of tubes, effectively reducing the overall stiffness variation.

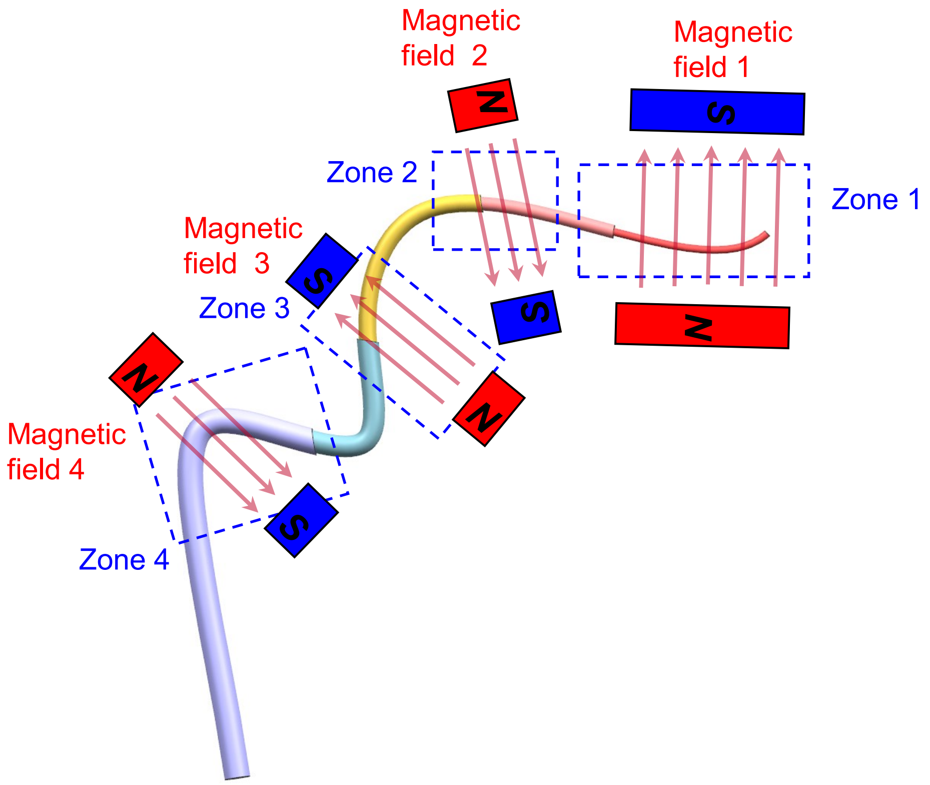

Supplementary Fig. 96. Combined scheme for the zone-based tube nesting design method and the multi-magnetic field control method. By assigning distinct magnetic fields to different zones, this integrated approach facilitates the design of both the tube's magnetization profile and the external magnetic fields, thereby achieving the desired deformation.

Legends for Supplementary Videos 1 to 21

The experimental videos associated with this study correspond to the sections of the paper as follows.

Real-time in-situ magnetization reprogramming

Video 1, Video 2

Contact-free object navigation

Video 3, Video 4, Video 5, Video 6

Reprogrammable cilia array

Video 7, Video 8, Video 9

Coordinated multi-instrument operation

Video 10, Video 11, Video 12, Video 13

Shape adaptable soft grippers

Video 14, Video 15

Other supplementary videos

Video 16, Video 17, Video 18, Video 19, Video 20, Video 21

Supplementary Video 1: 1D magnetization reprogramming

To demonstrate 1D magnetization reprogramming, we designed various soft tubes in Configurations A–G and evaluated their ability to undergo a range of deformations under a constant external magnetic field. Each deformation was achieved by adjusting its intrinsic magnetization profile, rather than by varying the external magnetic field. In Configuration G, we performed a control experiment to confirm that the tube's linear response in the magnetic field was not caused by stiffness changes from the internal rod. Moreover, two properties, magnetic neutralization and magnetic reversal, are also showcased in the movie.

Supplementary Video 2: 2D and 3D magnetization reprogramming

To more thoroughly demonstrate the proposed method, we selected four Configurations (A-D) in 2D magnetization reprogramming and included one configuration in 3D magnetization reprogramming. Their capacity to undergo a variety of deformations under a constant external magnetic field was then evaluated. The 2D magnetization reprogramming uses 2D sheets as carriers, while the 3D magnetization reprogramming employs 3D box-shaped structures as carriers.

Supplementary Video 3: Application I-1- Comparison of operating characteristics

To demonstrate the differences in operating characteristics between the proposed and the existing methods, we conducted experiments in a uniform magnetic field. With the existing method, the bent sections of the soft tube moved along with the tube itself. In contrast, using the proposed method, whether forming with two or three bends, the displacement of the bent sections of the soft tube remained independent of the motion of the soft tube.

Supplementary Video 4: Application I-2- Avoidance object bypassing

We established an experimental setup using soft tubes fabricated by both the existing and the proposed methods to observe potential contact with avoidance objects. We discovered that the proposed method, which involves forming with a bend and maintaining its position, successfully avoided any contact with the avoidance objects, an improvement not achieved by the existing methods. Moreover, we implemented a more complex scenario involving multiple avoidance objects, requiring the soft tube to form and maintain two bends to navigate through.

Supplementary Video 5: Application I-3- Experiments in vascular models

To validate the potential application of the proposed method in vascular interventional surgery, we constructed a vascular model featuring three different branches, each at a unique angle to the main vessel. We observed the performance of both existing and proposed methods in guiding a soft tube from the main vessel into these branches.

Supplementary Video 6: Application I-4- Ex vivo experiment

To further demonstrate the potential application of the proposed method in vascular interventional surgery, we conducted experiments using porcine heart vessels. The soft tube was placed within the vessels, and we advanced it while employing ultrasound imaging to observe and record the tube's movements and its contact with the vessel walls. We found that the soft tube could avoid contact with the sides of the vessels and only made contact with the vessel's bottom, thus achieving the ideal scenario.

Supplementary Video 7: Application II-1- Targeted cilia activation

We first demonstrated the activation of a single cilium, exploring the fundamental functionality of cilia. We then illustrated targeted cilia activation within a 5×5 cilia array, showcasing selective activation over a multiciliary system. Additionally, we explored potential path-directed propulsion, highlighting how directed movement could be achieved. Lastly, we integrated the targeted cilia activation with the ciliary phase modulation, demonstrating advanced techniques for coordinating ciliary movement to achieve optimized performance.

Supplementary Video 8: Application II-2- Ciliary bending amplitude modulation

We initially demonstrated bending amplitude modulation of a single cilium, showing that the cilium was able to achieve five distinct motion patterns under a constant-amplitude rotating magnetic field. Subsequently, to vividly demonstrate the variations in bending amplitude, we opted for a uniform magnetic field and conducted experiments within both 1×4 and 3×3 cilia arrays, respectively. These videos showcase the potential of cilia to achieve different motion patterns through the ciliary bending amplitude modulation.

Supplementary Video 9: Application II-3- Ciliary phase modulation

This video provides a comprehensive overview of our experiments on phase modulation in cilia arrays and its effects on fluid dynamics. Initially, we demonstrated

phase modulation in two cilia, followed by experiments involving six cilia, illustrating that the proposed method enables real-time, in-situ phase modulation of the cilia. Subsequently, we explored the effects of different phases on fluid propulsion. Ultimately, by adjusting the phase of the cilia in real time, we successfully altered the direction of the fluid drive.

Supplementary Video 10: Application III-1- Independent control of multiple soft tubes under the same magnetic field

To demonstrate independent control of multiple soft tubes under the same magnetic field, we achieved separate deformation control of two soft tubes within a uniform magnetic field. Initially, we controlled the deformation of a single soft tube. We then maintained the deformation of this tube while changing the deformation of a second soft tube. Subsequently, we retracted the second tube, returned to controlling the first, and finally retracted the first soft tube. These operations effectively demonstrated that the proposed method could achieve independent control of multiple soft tubes under the same magnetic field.

Supplementary Video 11: Application III-2- Coordinated operation of two soft tubes

We established a simple operational scenario where one soft tube advanced in a straight state before circumventing an obstacle in a curved state, and maintained this state. Subsequently, another soft tube advanced in a straight state and then maneuvered around the opposite side of the obstacle in a curved state, collaboratively completing the operation with the first tube. After completing the task, both tubes retracted in a straight state. All operations were conducted under a uniform magnetic field.

Supplementary Video 12: Application III-3- Coordinated operation of three soft tubes

We established a more intricate operational scenario, featuring an increased number of obstacles and requiring additional soft tubes. All operations required the

soft tubes to transition between straight and curved states within the same uniform magnetic field, navigating through obstacles to reach designated targets. After completing the task, the tubes similarly switched between straight and curved states to navigate back through the obstacles in a retraction phase.

Supplementary Video 13: Application III-4- Coordinated operation of three soft tubes in more complex tasks

We established an operational scenario more complex than that in Supplementary Video 12, involving two soft tubes sequentially navigating through obstacles to reach and operate at the target locations. One of the pathways was completely obstructed, rendering it impassable by mere changes in the soft tube's state. This required the deployment of a third soft tube tasked with appropriately repositioning the obstacle to enable passage. This operation further demonstrated the capability of the proposed method to achieve coordinated multi-instrument operations.

Supplementary Video 14: Application IV-1- Various bending modes of shape-conforming soft grippers

We illustrated how shape-conforming soft grippers can achieve multiple bending modes under a uniform magnetic field by adjusting their magnetization profiles. These bending modes are similar to the movements of a human hand, enabling the selective bending of "fingers" based on specific needs. For more intuitive presentation, schematic diagrams were provided alongside each experimental video.

Supplementary Video 15: Application IV-2- Demonstration of grasping objects with different shapes

We positioned two shape-conforming soft grippers side by side, creating a setup akin to a pair of human hands. This arrangement allows us to dictate the state of each "finger" (whether bent or straight), facilitating the grasp of objects with various shapes. We created objects in three distinct shapes to showcase the versatility of the soft grippers. By altering their magnetization profiles, we successfully demonstrated the capability to grasp these varied objects in a uniform magnetic field.

Supplementary Video 16: 3D operation under a fixed magnetic field

To demonstrate the 3D manipulation capabilities of the proposed method under a fixed magnetic field, we generated the magnetic field using a Halbach array, which maintains constant magnitude and direction. Simultaneously, we utilized a rod featuring a helical magnetization profile at its tip, in conjunction with an outer tube. We observed the 3D manipulation of the tube under a fixed magnetic field.

Supplementary Video 17: 3D Navigation operation demonstration

To demonstrate the 3D navigation ability of the proposed method, we controlled the tube to bypass various obstacles under a single magnetic field. Two different obstacle configurations were set up—one with a single obstacle and another with two obstacles—both of which require 3D navigation to overcome. Accordingly, we configured the tube with two distinct magnetization profiles. Under a constant magnetic field direction, the field was gradually ramped up. As the field intensity was incrementally increased or decreased, the tube underwent smooth 3D deformation and successfully surmounted both obstacle configurations.

Supplementary Video 18: Long-range operation with a large Magnetic Interaction Length

This video validates our proposed "Long-Range Operation with a Large Magnetic Interaction Length" mode. Two Halbach arrays were employed to generate dual magnetic fields, spatially configured to form an extensive Magnetic Interaction Zone. Initially, we showed that the tube can maintain a fixed operating mode at its tip, where its 3D deformation pattern remains constant. Subsequently, we illustrated that the tube can also adopt a variable operating mode at its tip, enabling real-time adjustment of its 3D deformation pattern. In the aforementioned operations, the tube's operational length reached 305 mm.

Supplementary Video 19: Long-range operation with a large Feeding Length

In this video, we demonstrate a mode we term "Long-range Operation with a

Large Feeding Length.” Utilizing the proposed segmented design method, the tube was constructed in segments. The tube integrates a commercial catheter with a soft tube to accommodate operations within a large Feeding Zone. By manipulating the tube to traverse the Feeding Zone and enter the magnetic field region, the desired deformations are achieved. We demonstrated that at the tube’s tip, both 2D and 3D deformations—as well as variable operating modes—can be realized. In this operation, the tube’s effective operational length reached 1240 mm.

Supplementary Video 20: 1D magnetization reprogramming in miniature structures

To demonstrate that the proposed method is applicable for small-scale operations, we fabricated a tube with an outer diameter of 0.7 mm, configured according to Configurations B in 1D magnetization reprogramming. The video shows that the tube’s magnetization profile can be altered using the proposed method to achieve different deformation modes. To rule out the influence of the inner rod on the deformation, we fabricated a non-magnetic rod and conducted a control experiment. The comparison revealed that the deformation exhibited by the tube with a non-magnetic rod is completely different from that of the tube with a magnetic rod.

Supplementary Video 21: Surface tension effects in tube fabrication

We employed the dip coating method to fabricate tubes in this study. To demonstrate the defects associated with fabricating small-scale tubes using this approach, we recorded the flow behavior of polydimethylsiloxane (PDMS) on the surface during the fabrication process. A 0.5 mm diameter copper wire was used as the carrier, on which PDMS with a base-to-curing agent mass ratio of 10:1 was uniformly applied. At this scale, surface tension dominates, causing the PDMS to gradually coalesce into spherical droplets. This phenomenon renders the method unsuitable for fabricating small-scale tubes.

References

1. W. Hu, G. Z. Lum, M. Mastrangeli, M. Sitti, Small-scale soft-bodied robot with multimodal locomotion. *Nature* **554**, 81–85 (2018).

2. J. Zhang, Z. Ren, W. Hu, R. H. Soon, I. C. Yasa, Z. Liu, M. Sitti, Voxelated three-dimensional miniature magnetic soft machines via multimaterial heterogeneous assembly. *Sci. Robot.* **6**, eabf0112 (2021).

3. Y. Alapan, A. C. Karacakol, S. N. Guzelhan, I. Isik, M. Sitti, Reprogrammable shape morphing of magnetic soft machines. *Sci. Adv.* **6**, eabc6414 (2020).

4. X. Kuang, S. Wu, Q. Ze, L. Yue, Y. Jin, S. M. Montgomery, F. Yang, H. J. Qi, R. Zhao, Magnetic Dynamic Polymers for Modular Assembling and Reconfigurable Morphing Architectures. *Adv. Mater.* **33**, 2102113 (2021).

5. H. Wen, Y. Sun, R. Liu, L. Li, Q. Cao, Reprogrammable magnetization pattern and shape morphing of phase-change magnetic soft composites. *Compos. Commun.* **40**, 101618 (2023).

6. H. Song, H. Lee, J. Lee, J. K. Choe, S. Lee, J. Y. Yi, S. Park, J.-W. Yoo, M. S. Kwon, J. Kim, Reprogrammable Ferromagnetic Domains for Reconfigurable Soft Magnetic Actuators. *Nano Lett.* **20**, 5185–5192 (2020).

7. Q. Ze, X. Kuang, S. Wu, J. Wong, S. M. Montgomery, R. Zhang, J. M. Kovitz, F. Yang, H. J. Qi, R. Zhao, Magnetic Shape Memory Polymers with Integrated Multifunctional Shape Manipulation. *Adv. Mater.* **32**, 1906657 (2020).

8. H. Deng, K. Sattari, Y. Xie, P. Liao, Z. Yan, J. Lin, Laser reprogramming magnetic anisotropy in soft composites for reconfigurable 3D shaping. *Nat. Commun.* **11**, 6325 (2020).

9. K. Ni, Q. Peng, E. Gao, K. Wang, Q. Shao, H. Huang, L. Xue, Z. Wang, Core–Shell Magnetic Micropillars for Reprogrammable Actuation. *ACS Nano* **15**, 4747–4758 (2021).

10. J. Cui, T.-Y. Huang, Z. Luo, P. Testa, H. Gu, X.-Z. Chen, B. J. Nelson, L. J. Heyderman, Nanomagnetic encoding of shape-morphing micromachines. *Nature* **575**, 164–168 (2019).

11. M. R. Clary, S. N. Cantu, J. A.-C. Liu, E. E. Evans, J. B. Tracy, Magnetic Reprogramming of Self-Assembled Hard-Magnetic Cilia. *Adv. Mater. Technol.* **9**, 2302243 (2024).

12. H. Rafii-Tari, C. J. Payne, G.-Z. Yang, Current and Emerging Robot-Assisted Endovascular Catheterization Technologies: A Review. *Ann. Biomed. Eng.* **42**,

697–715 (2014).

13. M. J. Mack, Minimally Invasive and Robotic Surgery. *J. Am. Med. Assoc.* **285**, 568 (2001).

14. M. Runciman, A. Darzi, G. P. Mylonas, Soft Robotics in Minimally Invasive Surgery. *Soft Robot.* **6**, 423–443 (2019).

15. J. Jayender, R. V. Patel, S. Nikumb, Robot-assisted Active Catheter Insertion: Algorithms and Experiments. *Int. J. Rob. Res.* **28**, 1101–1117 (2009).

16. H.-J. Cha, H.-S. Yoon, K. Y. Jung, B.-J. Yi, S. Lee, J. Y. Won, "A robotic system for percutaneous coronary intervention equipped with a steerable catheter and force feedback function" in *IEEE/RSJ International Conference on Intelligent Robots and Systems (IROS)* (IEEE, 2016), pp. 1151–1156.

17. X. Bao, S. Guo, N. Xiao, Y. Li, C. Yang, Y. Jiang, A cooperation of catheters and guidewires-based novel remote-controlled vascular interventional robot. *Biomed. Microdevices* **20**, 20 (2018).

18. X. Bao, S. Guo, N. Xiao, Y. Li, C. Yang, R. Shen, J. Cui, Y. Jiang, X. Liu, K. Liu, Operation evaluation in-human of a novel remote-controlled vascular interventional robot. *Biomed. Microdevices* **20**, 34 (2018).

19. X. Bao, S. Guo, Y. Guo, C. Yang, L. Shi, Y. Li, Y. Jiang, Multilevel Operation Strategy of a Vascular Interventional Robot System for Surgical Safety in Teleoperation. *IEEE Trans. Robot.* **38**, 2238–2250 (2022).

20. X. Bao, S. Guo, C. Yang, L. Zheng, Haptic Interface With Force and Torque Feedback for Robot-Assisted Endovascular Catheterization. *IEEE/ASME Trans. Mechatron.*, 1–15 (2023).

21. Y.-H. Kim, Y.-J. Park, H. In, C. W. Jeong, K.-J. Cho, Design Concept of Hybrid Instrument for Laparoscopic Surgery and Its Verification Using Scale Model Test. *IEEE/ASME Trans. Mechatron.*, 1–1 (2015).

22. G. A. Fontanelli, M. Selvaggio, L. R. Buonocore, F. Ficuciello, L. Villani, B. Siciliano, A New Laparoscopic Tool With In-Hand Rolling Capabilities for Needle Reorientation. *IEEE Robot. Autom. Lett.* **3**, 2354–2361 (2018).

23. H. Liu, M. Selvaggio, P. Ferrentino, R. Moccia, S. Pirozzi, U. Bracale, F. Ficuciello, The MUSHA Hand II: A Multi-Functional Hand for Robot-Assisted Laparoscopic Surgery. *IEEE/ASME Trans. Mechatron.*, 1–1 (2020).

24. K.-W. Kwok, K. Hung Tsoi, V. Vitiello, J. Clark, G. C. T. Chow, W. Luk, G.-Z. Yang, Dimensionality Reduction in Controlling Articulated Snake Robot for Endoscopy Under Dynamic Active Constraints. *IEEE Trans. Robot.* **29**, 15–31

(2013).

25. J. M. Prendergast, G. A. Formosa, M. J. Fulton, C. R. Heckman, M. E. Rentschler, A Real-Time State Dependent Region Estimator for Autonomous Endoscope Navigation. *IEEE Trans. Robot.* **37**, 918–934 (2021).

26. W. Wei, R. E. Goldman, H. F. Fine, S. Chang, N. Simaan, Performance Evaluation for Multi-arm Manipulation of Hollow Suspended Organs. *IEEE Trans. Robot.* **25**, 147–157 (2009).

27. A. Ebrahimi, M. G. Urias, N. Patel, R. H. Taylor, P. Gehlbach, I. Iordachita, Adaptive Control Improves Sclera Force Safety in Robot-Assisted Eye Surgery: A Clinical Study. *IEEE Trans. Biomed. Eng.* **68**, 3356–3365 (2021).

28. H. G. Shin, I. Park, K. Kim, H. K. Kim, W. K. Chung, Corneal Suturing Robot Capable of Producing Sutures With Desired Shape for Corneal Transplantation Surgery. *IEEE Trans. Robot.* **37**, 304–312 (2021).

29. D. Kundrat, G. Dagnino, T. M. Y. Kwok, M. E. M. K. Abdelaziz, W. Chi, A. Nguyen, C. Riga, G.-Z. Yang, An MR-Safe Endovascular Robotic Platform: Design, Control, and Ex-Vivo Evaluation. *IEEE Trans. Biomed. Eng.* **68**, 3110–3121 (2021).

30. F. Bechet, K. Ogawa, E. Sariyildiz, K. Ohnishi, Electrohydraulic Transmission System for Minimally Invasive Robotics. *IEEE Trans. Ind. Electron.* **62**, 7643–7654 (2015).

31. M. P. Armacost, J. Adair, T. Munger, R. R. Viswanathan, F. M. Creighton, D. T. Curd, R. Sehra, Accurate and Reproducible Target Navigation with the Stereotaxis Niobe® Magnetic Navigation System. *J. Cardiovasc. Electr.* **18** (2007).

32. Y. Kim, G. A. Parada, S. Liu, X. Zhao, Ferromagnetic soft continuum robots. *Sci. Robot.* **4**, eaax7329 (2019).

33. F. Qi, F. Ju, D. Bai, Y. Wang, B. Chen, Kinematic analysis and navigation method of a cable-driven continuum robot used for minimally invasive surgery. *Int. J. Med. Robot. Comput. Assist. Surg.* **15**, e2007 (2019).

34. H. Rafii-Tari, C. V. Riga, C. J. Payne, M. S. Hamady, N. J. W. Cheshire, C. D. Bicknell, G.-Z. Yang, Reducing contact forces in the arch and supra-aortic vessels using the Magellan robot. *J. Vasc. Surg.* **64**, 1422–1432 (2016).

35. J. Jayender, R. V. Patel, S. Nikumb, "Robot-assisted catheter insertion using hybrid impedance control" in *IEEE International Conference on Robotics and Automation (ICRA)* (IEEE, Orlando, FL, USA, 2006), pp. 607–612.

36. ASAHI INTECC CO., LTD, Core proprietary technologies, *ASAHI INTECC*

MEDICAL PRODUCTS. https://medical.asahi-intecc.com/en/technologies.

37. Y. Cao, Z. Yang, B. Hao, X. Wang, M. Cai, Z. Qi, B. Sun, Q. Wang, L. Zhang, Magnetic Continuum Robot with Intraoperative Magnetic Moment Programming. *Soft Robot.* **10**, 1209–1223 (2023).

38. P. Yang, L. Mao, C. Tian, X. Meng, H. Xie, A Cooperative and Multifunctional Magnetic Continuum Robot for Noninteractive Access, Dexterous Navigation, and Versatile Manipulation. *Adv. Funct. Mater.* **35**, 2412543 (2025).

39. L. Mao, P. Yang, C. Tian, X. Shen, F. Wang, H. Zhang, X. Meng, H. Xie, Magnetic steering continuum robot for transluminal procedures with programmable shape and functionalities. *Nat. Commun.* **15**, 3759 (2024).

40. R. Faubel, C. Westendorf, E. Bodenschatz, G. Eichele, Cilia-based flow network in the brain ventricles. *Science* **353**, 176–178 (2016).

41. M. A. Sleigh, J. R. Blake, N. Liron, The Propulsion of Mucus by Cilia. *Am. Rev. Respir. Dis.* **137**, 726–741 (1988).

42. R. A. Lyons, E. Saridogan, O. Djahanbakhch, The reproductive significance of human Fallopian tube cilia. *Hum. Reprod. Update* **12**, 363–372 (2006).

43. J. R. Blake, M. A. Sleigh, Mechanics of Ciliary Locomotion. *Biol. Rev.* **49**, 85–125 (1974).

44. W. Gilpin, V. N. Prakash, M. Prakash, Vortex arrays and ciliary tangles underlie the feeding–swimming trade-off in starfish larvae. *Nat. Phys.* **13**, 380–386 (2017).

45. M. G. Stafford-Smith, R. F. G. Ormond, Sediment-rejection mechanisms of 42 species of Australian scleractinian corals. *Mar. Freshwater. Res.* **43**, 683–705 (1992).

46. X. Zhang, J. Guo, X. Fu, D. Zhang, Y. Zhao, Tailoring Flexible Arrays for Artificial Cilia Actuators. *Adv. Intell. Syst.* **3**, 2000225 (2021).

47. T. ul Islam, Y. Wang, I. Aggarwal, Z. Cui, H. E. Amirabadi, H. Garg, R. Kooi, B. B. Venkataramanachar, T. Wang, S. Zhang, P. R. Onck, J. M. J. den Toonder, Microscopic artificial cilia – a review. *Lab Chip* **22**, 1650–1679 (2022).

48. S. Peerlinck, E. Milana, E. De Smet, M. De Volder, D. Reynaerts, B. Gorissen, Artificial Cilia–Bridging the Gap with Nature. *Adv. Funct. Mater.* **33**, 2300856 (2023).

49. W. Wang, Q. Liu, I. Tanasijevic, M. F. Reynolds, A. J. Cortese, M. Z. Miskin, M. C. Cao, D. A. Muller, A. C. Molnar, E. Lauga, P. L. McEuen, I. Cohen, Cilia metasurfaces for electronically programmable microfluidic manipulation. *Nature*

605, 681–686 (2022).

50. Z. Ren, M. Zhang, S. Song, Z. Liu, C. Hong, T. Wang, X. Dong, W. Hu, M. Sitti, Soft-robotic ciliated epidermis for reconfigurable coordinated fluid manipulation. *Sci. Adv.* **8**, eabq2345 (2022).

51. S. Li, M. M. Lerch, J. T. Waters, B. Deng, R. S. Martens, Y. Yao, D. Y. Kim, K. Bertoldi, A. Grinthal, A. C. Balazs, J. Aizenberg, Self-regulated non-reciprocal motions in single-material microstructures. *Nature* **605**, 76–83 (2022).

52. J. Han, X. Dong, Z. Yin, S. Zhang, M. Li, Z. Zheng, M. C. Ugurlu, W. Jiang, H. Liu, M. Sitti, Actuation-enhanced multifunctional sensing and information recognition by magnetic artificial cilia arrays. *Proc. Natl. Acad. Sci.* **120**, e2308301120 (2023).

53. S. Zhang, X. Hu, M. Li, U. Bozuyuk, R. Zhang, E. Suadiye, J. Han, F. Wang, P. Onck, M. Sitti, 3D-printed micrometer-scale wireless magnetic cilia with metachronal programmability. *Sci. Adv.* **9**, eadf9462 (2023).

54. X. Dong, G. Z. Lum, W. Hu, R. Zhang, Z. Ren, P. R. Onck, M. Sitti, Bioinspired cilia arrays with programmable nonreciprocal motion and metachronal coordination. *Sci. Adv.* **6**, eabc9323 (2020).

55. Z. Cui, Y. Wang, S. Zhang, T. Wang, J. M. J. den Toonder, Miniaturized metachronal magnetic artificial cilia. *Proc. Natl. Acad. Sci.* **120**, e2304519120 (2023).

56. C. Freschi, V. Ferrari, F. Melfi, M. Ferrari, F. Mosca, A. Cuschieri, Technical review of the da Vinci surgical telemanipulator. *Int. J. Med. Robot. Comput. Assist. Surg.* **9**, 396–406 (2013).

57. Y. Kim, E. Genevriere, P. Harker, J. Choe, M. Balicki, R. W. Regenhardt, J. E. Vranic, A. A. Dmytriw, A. B. Patel, X. Zhao, Telerobotic neurovascular interventions with magnetic manipulation. *Sci. Robot.* **7**, eabg9907 (2022).

58. E. Diller, J. Giltinan, M. Sitti, Independent control of multiple magnetic microrobots in three dimensions. *Int. J. Rob. Res.* **32**, 614–631 (2013).

59. F. Chen, W. Xu, H. Zhang, Y. Wang, J. Cao, M. Y. Wang, H. Ren, J. Zhu, Y. F. Zhang, Topology Optimized Design, Fabrication, and Characterization of a Soft Cable-Driven Gripper. *IEEE Robot. Autom. Lett.* **3**, 2463–2470 (2018).

60. Y. Liu, Q. Bi, Y. Li, “Development of a Bio-inspired Soft Robotic Gripper based on Tensegrity Structures” in *IEEE/RSJ International Conference on Intelligent Robots and Systems (IROS)* (2021), pp. 7398–7403.

61. Z. Xiang, L. Hongwei, D. Bingxiao, C. Lu, C. Xiaoqian, H. Yiyong, “Design and

experimental validation of a cable-driven continuum manipulator and soft gripper" in *IEEE International Conference on Robotics and Biomimetics (ROBIO)* (2019), pp. 1965–1968.

62. Y. Li, Y. Chen, Y. Li, Pre-Charged Pneumatic Soft Gripper With Closed-Loop Control. *IEEE Robot. Autom. Lett.* **4**, 1402–1408 (2019).

63. Z. Zhang, X. Ni, H. Wu, M. Sun, G. Bao, H. Wu, S. Jiang, Pneumatically Actuated Soft Gripper with Bistable Structures. *Soft Robot.* **9**, 57–71 (2022).

64. R. Deimel, O. Brock, A novel type of compliant and underactuated robotic hand for dexterous grasping. *Int. J. Rob. Res.* **35**, 161–185 (2016).

65. W. Thongking, A. Wiranata, A. Minaminosono, Z. Mao, S. Maeda, Soft Robotic Gripper Based on Multi-Layers of Dielectric Elastomer Actuators. *J. Robot. Mechatron.* **33**, 968–974 (2021).

66. G.-K. Lau, K.-R. Heng, A. S. Ahmed, M. Shrestha, Dielectric elastomer fingers for versatile grasping and nimble pinching. *Appl. Phys. Lett.* **110**, 182906 (2017).

67. S. Shian, K. Bertoldi, D. R. Clarke, Dielectric Elastomer Based "Grippers" for Soft Robotics. *Adv. Mater.* **27**, 6814–6819 (2015).

68. W. Wang, S.-H. Ahn, Shape Memory Alloy-Based Soft Gripper with Variable Stiffness for Compliant and Effective Grasping. *Soft Robot.* **4**, 379–389 (2017).

69. Y. She, C. Li, J. Cleary, H.-J. Su, Design and Fabrication of a Soft Robotic Hand With Embedded Actuators and Sensors. *J. Mechanisms Robotics.* **7** (2015).

70. H. Rodrigue, W. Wang, D.-R. Kim, S.-H. Ahn, Curved shape memory alloy-based soft actuators and application to soft gripper. *Compos. Struct.* **176**, 398–406 (2017).

71. Y. Thakur, D. W. Holdsworth, M. Drangova, Characterization of Catheter Dynamics During Percutaneous Transluminal Catheter Procedures. *IEEE Trans. Biomed. Eng.* **56**, 2140–2143 (2009).

72. H. Rafii-Tari, C. J. Payne, C. Riga, C. Bicknell, S.-L. Lee, G.-Z. Yang, "Assessment of Navigation Cues with Proximal Force Sensing during Endovascular Catheterization" in *Medical Image Computing and Computer-Assisted Intervention (MICCAI)*, N. Ayache, H. Delingette, P. Golland, K. Mori, Eds. (Springer, Berlin, Heidelberg, 2012), pp. 560–567.

73. A. C. Karacakol, "Design, Simulation, and Programming of Magnetic Soft Robots," thesis, Carnegie Mellon University (2023).

74. C. Wang, Z. Zhao, X. S. Zhang, Inverse design of magneto-active metasurfaces

and robots: Theory, computation, and experimental validation. Comput. Methods Appl. Mech. Eng. 413, 116065 (2023).

75. F. Wang, Created in BioRender. https://BioRender.com/812z0ds (2025).
